# Nickel(II)-Catalyzed Formal [3+2] Cycloadditions between Indoles and Donor–Acceptor Cyclopropanes

**DOI:** 10.3390/molecules29071604

**Published:** 2024-04-03

**Authors:** Víctor Quezada, Mariña Castroagudín, Felipe Verdugo, Sergio Ortiz, Guillermo Zaragoza, Fabiane M. Nachtigall, Francisco A. A. Reis, Alejandro Castro-Alvarez, Leonardo S. Santos, Ronald Nelson

**Affiliations:** 1 Departamento de Química, Facultad de Ciencias, Universidad Católica del Norte, Avda. Angamos 0610, Antofagasta 1270709, Chile; victor.quezada@ucn.cl (V.Q.); marina.castroagudin@ce.ucn.cl (M.C.); 2 Departamento de Química Orgánica, Facultad de Ciencias Químicas, Universidad de Concepción, Edmundo Larenas 129, Concepción 4070371, Chile; felipeverdugo@udec.cl; 3 UMR 7200 Laboratoire d’Innovation Thérapeutique, CNRS, Strasbourg Drug Discovery and Development Institute (IMS), Université de Strasbourg, 67400 Illkirch-Graffenstaden, France; ortizaguirre@unistra.fr; 4 Unidade de Difracción de Raios X, RIAIDT, Universidade de Santiago de Compostela, Campus VIDA, 15782 Santiago de Compostela, Spain; g.zaragoza@usc.es; 5 Instituto de Ciencias Aplicadas, Universidad Autónoma de Chile, Talca 3467987, Chile; fabiane.manke@uautonoma.cl; 6 Laboratory of Asymmetric Synthesis, Chemistry Institute of Natural Resources, Universidad de Talca, Talca 3460000, Chile; francisco.aragao@utalca.cl; 7 Departamento de Ciencias Preclínicas, Facultad de Medicina, Universidad de La Frontera, Temuco 4811230, Chile

**Keywords:** nickel catalyzed, [3+2] cycloaddition, donor–acceptor cyclopropanes, indoles, cyclopenta[*b*]indoles

## Abstract

This article describes the development of a nickel-catalyzed regio- and diastereoselective formal [3+2] cycloaddition between *N*-substituted indoles and donor–acceptor cyclopropanes to synthesize cyclopenta[*b*]indoles. Optimized reaction conditions provide the desired nitrogen-containing cycloadducts in up to 93% yield and *dr* 8.6:1 with complete regioselectivity. The substrate scope showed high tolerance to various substituted indoles and cyclopropanes, resulting in the synthesis of six new cyclopenta[*b*]indoles and the isolation of five derivatives previously reported in the literature. In addition, a mechanistic proposal for the reaction was studied through online reaction monitoring by ESI-MS, allowing for the identification of the reactive intermediates in the Ni(II) catalyzed process. X-ray crystallography confirmed the structure and relative endo stereochemistry of the products. This method enables the fast and efficient construction of fused indolines from readily accessible starting materials.

## 1. Introduction

**Figure 1:**
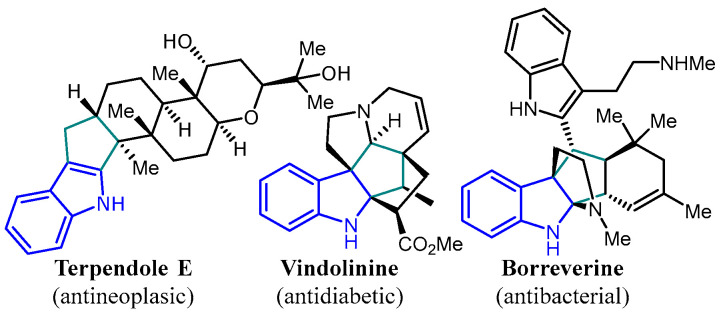
Cyclopenta[*b*]indoles in natural products.

Fused nitrogen heterocycles, such as indoles and indolines, are ubiquitous in natural products, pharmaceuticals, and bioactive compounds [1,2,3,4]. Notably, derivatives like cyclopenta[*b*]indoles, a type of C2,C3-fused indoline, are prevalent scaffolds in a plethora of biologically active alkaloids [1,2,3,4,5,6]. For example, terpendole E is an anticarcinogen inhibitor of cellular mitosis [7,8], while vindolinine, an antidiabetic compound isolated from Catharanthus roseus [9,10], and borreverine are strongly active against Gram-positive bacteria (Figure 1) [11,12].

Due to the interesting biological properties and structural complexity of these indolic compounds, their synthesis stands out as a prominent area of interest in the field of synthetic chemistry. The reported methods include Nazarov cyclization [13,14], addition-cyclization strategies [15,16], Dieckmann condensation [17], variations in Fischer indole synthesis [18,19,20], organocatalysis [21,22], tin-promoted cycloadditions [23], aluminum-promoted intramolecular imino–ene reactions [24], palladium-catalyzed cross-couplings [25,26,27], and cycloadditions catalyzed by Rh [28,29], Ir [30,31], or Au complexes [24,32,33,34]. However, many of these strategies lack atom economy, rely on the use of precious metal catalysts, or involve relatively elaborate substrates.

In this context, intermolecular [3+2] cycloadditions have emerged as powerful for the construction of five-membered rings in a single synthetic step. Significant contributions to this field have been made through catalytic reactions of vinyldiazo compounds [29,35], azomethine ylides [36], and trimethylenemethane (TMM) [37] and methodologies employing donor–acceptor cyclopropanes [20]. This latter strategy is particularly appealing because it would provide a straightforward and atom-economical strategy for gaining access to cyclopenta[*b*]indole cores [38,39,40,41,42,43,44].

**Scheme 1:**
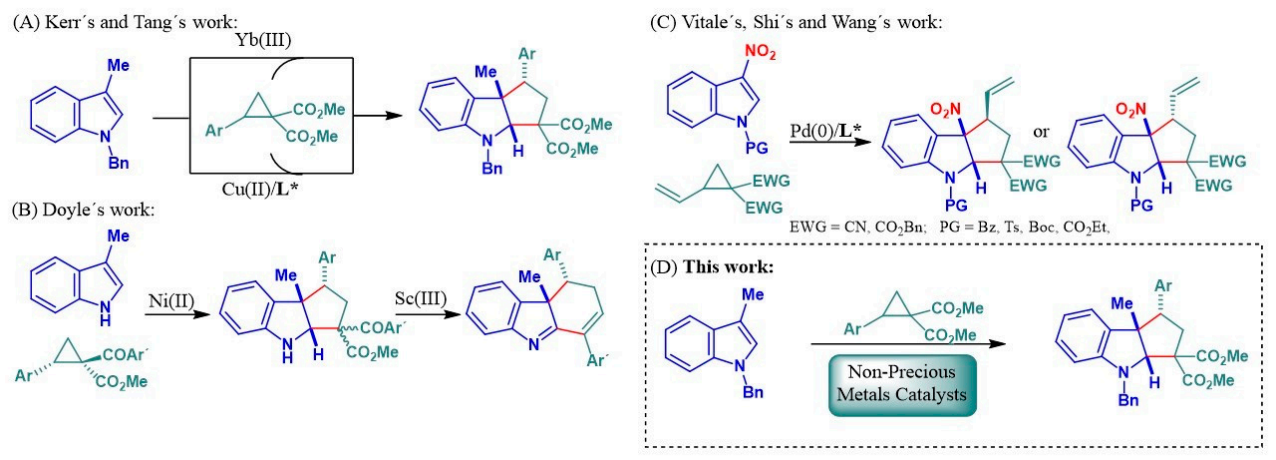
[3+2] cycloadditions of 3-methylindole and 3-nitroindole with donor–acceptor cyclopropanes. L* = Chiral ligand.

In this regard, the Kerr group pioneered the synthesis of this skeleton through a [3+2] cycloaddition of donor–acceptor cyclopropanes and 3-methylindoles as a two-carbon synthon catalyzed by Yb(OTf)_3_ (Scheme 1A) [45,46,47]. Subsequently, in 2013, Xie and Tang reported the use of Cu(II) catalysts (Scheme 1A), in combination with BOX-type chiral ligands, for an enantioselective version of this cyclization [48]. Recently, during the development of this project, Doyle’s group reported the use of 3-alkylindoles as an interesting strategy for the construction of carbocyclic systems fused to indoles via a Ni(II) catalyzed stereoretentive [3+2] cycloaddition, followed by a one-pot Sc(III)-catalyzed decarboxylative ring expansion to afford the corresponding cyclopenta[*b*]indoles and dihydro-3*H*-carbazoles (Scheme 1B) [49]. In the initial stage catalyzed by Ni(II), the authors describe cyclopenta[*b*]indole formation via [3+2] cycloaddition with good retention of the chirality of the starting product, but with a low diastereoselectivity, the inconvenience of which is elegantly overcome in a subsequent rearrangement/decarboxylation stage catalyzed by Sc(OTf)_3_ [49]. On the other hand, other research groups have explored the use of 3-nitroindoles as more reactive species in [3+2] cycloadditions, using vinylcyclopropanes (VCPs) in Pd(0)-catalyzed processes to obtain cyclopenta[*b*]indole systems (Scheme 1C) [50,51,52,53]. Following a similar strategy, but employing phenylaziridines or oxiranyl-dicarboxylates instead of VCPs, other authors have described the efficient synthesis of pyrrolo[2,3-*b*]indole and furo[3,4-*b*]indole scaffolds [54,55,56]. These cyclopropanes can be activated by a variety of Lewis acids, including those based on non-precious metals that are highly desirable for their abundance and cost-effectiveness [57,58,59,60].

In this context, and despite the precedents for the use of non-precious metals to access cyclopenta[*b*]indole skeletons from skatole derivatives, this field is still underdeveloped. Therefore, starting from the general concept of opening metal-catalyzed cyclopropane derivatives, the main objective of this work is the application of non-precious-metal-based catalysts in the diastereoselective synthesis of cyclopenta[*b*]indole-type skeletons in a single synthetic stage by opening donor–acceptor cyclopropanes and guiding their subsequent formal [3+2] cycloaddition with C3-substituted indoles (Scheme 1D).

## 2. Results and Discussion

We started our study by employing different Lewis acids based on non-precious metals, using *N*-benzylskatole (**1a**) and cyclopropane **2a** as model substrates. As an initial control experiment, we attempted to reproduce the reported reaction conditions when employing Cu(OTf)_2_ as a catalyst [48], affording the desired cycloadduct **3aa** at a 79% yield and a diastereoismeric ratio (*dr*) of 5:1, consistent with the literature reports (Table 1, entry 1) [48]. Next, the reactivity of different metal salts was evaluated. The use of Co(ClO_4_)_2_·6H_2_O led to 42% **3aa** with a modest increase in diastereoselectivity (*dr* = 6:1, Table 1, entry 2). On the other hand, Zn(ClO_4_)_2_·6H_2_O showed a similar enhancement of diastereoselectivity (*dr* = 6.4:1) but with a significant decrease in yield (28%, Table 1, entry 3). Interestingly, the use of Ni(ClO_4_)_2_·6H_2_O as the catalyst led to **3aa** in an excellent 95% yield and with a 6:1 ratio of isomers (Table 1, entry 4). This diastereomeric ratio is considerably higher than the 1:1 ratio described by the Doyle group under similar reaction conditions [49]. Furthermore, the use of Ni(II) salts, such as Ni(ClO_4_)_2_·6H_2_O, in cycloaddition reactions of donor–acceptor cyclopropanes has been previously described in the literature [56,61,62,63], which supports the observed results. Other metal salts of Mn^2+^, Fe^2+^, Fe^3+^, and Al^3+^ were also evaluated (see Table S1 in the Supplementary Materials), but they were inefficient in obtaining cyclopenta[*b*]indole **3aa**. Based on these results, we continued our study by evaluating different Ni^2+^ salts. When using a catalytic load of 10 mol%, Ni(ClO_4_)_2_·6H_2_O showed a significantly slower conversion, although it led to a 48% yield (Table 1, entry 5). On the other hand, when Ni(OAc)_2_·4H_2_O was employed, a negligible conversion and reaction yield were observed after 24 h (Table 1, entry 6). Other salts such as NiSO_4_·6H_2_O, Ni(NO_3_)_2_·6H_2_O, or NiCl_2_ were evaluated, providing the desired product in modest yields and incomplete conversions (Table 1, entries 7 to 9). Notably, the use of Ni(OTf)_2_ (Table 1, entry 10) resulted in a significant increase in the yield of the product, reaching 78%, with a diastereomeric ratio of 5:1. At this point, we evaluated the influence of different solvents on the reaction. The use of DCM led to a decrease in yield (47%) with a *dr* = 7.7:1 (Table 1, entry 11), while with CHCl_3_, it provided only a 25% yield with an important decrease in diastereoselectivity (Table 1, entry 12). Given the reports of Tang and Doyle [48,49], we also evaluated the use of toluene as a solvent, observing a poor 29% yield and no diastereoselectivity (Table 1, entry 13). Acetonitrile and ethyl acetate were also evaluated, although the starting material was fully recovered in both cases (Table 1, entries 14 and 15). To consult the other solvents evaluated, see Table S2 in the Supplementary Materials.

Having identified Ni(OTf)_2_ as the most suitable promoter of the [3+2] cycloaddition, we initially evaluated different substituted indoles. Unfortunately, we observed the formation of the desired products with low yields and diastereoselectivities (Table S3 in the Supplementary Materials). For this reason, we decided to assess the influence of different ligands commonly employed in this type of reaction, including bidentate nitrogenous ligands, bisphosphines, and *N,N*’-dioxides (Table 2) [56,64,65]. We started by evaluating nitrogen- and phosphorous-based bidentate ligands such as Bipy, Phen, dppe, d^F^ppe, L_2_-PrPr_2_, and L_3_-PrPr_2_ (Table 2, entries 1 to 6), but unfortunately, none of these catalysts improved the results observed with Ni(OTf)_2_ (see Table 1, entry 10). On this basis, we decided to reevaluate Ni(ClO_4_)_2_·6H_2_O, considering that it proved to be a competent catalyst (Table 1, entry 4) and taking advantage of the labile nature of the perchlorate ion to facilitate the formation of cationic metal–ligand complexes [66]. With the use of bidentate ligands such as Phen or dppe, the formation of compound **3aa** was observed with low yields (Table 2, entries 7 and 8). In contrast, bisphosphines such as dfppe and dppBz afforded the product in modest 43% and 53% yields, respectively (Table 2, entries 9 and 10), while with dppf, no reactivity was observed (Table 2, entry 11). Gratifyingly, the evaluation of rac-BINAP [67,68] resulted in a significant acceleration of the reaction, providing the desired **3aa** product in a 73% yield with a diastereoisomeric ratio of 6:1 after 5 h at room temperature (Table 2, entry 12). For more details on ligand optimization, see Table S4 in the Supplementary Materials. The effect of temperature was also evaluated, with us observing that at both 50 and 80 °C, there was a decrease in the isolated yield of **3aa**, possibly due to the degradation of the starting materials, along with a decrease in the diastereoisomeric ratio (Table 2, entries 13 and 14).


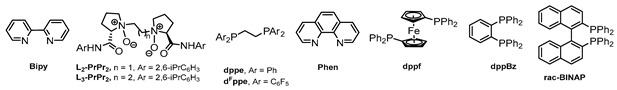
 The results listed in Table 2 suggest that the rigidity and bite angle of bidentate phosphines might be crucial factors in the system’s reactivity. Ligands such as dppe (flexible ligand) and dppBz (rigid ligand) [69], with approximately 90° angles, exhibited a reduced yield and a significant increase in reaction time compared to the BINAP version, which features a greater bite angle (approximately 93°) than the previously mentioned ligands [70]. Ligands with larger angles, such as dppf (99°) [71], also fail to provide ideal conditions for this [3+2] cycloaddition. We primarily evaluated a possible enantioselective version of the cycloaddition using chiral bisphosphines such as (*R*)-BINAP, (*R*)-DM-BINAP, and (*S*)-DTBM-DEGPHOS. Among these, only the BINAP derivatives effectively catalyzed the reaction, yielding up to 81%, but not exceeding 40% ee in any case (see details in Table S5 in the Supplementary Materials). Further studies to develop a highly enantioselective version are under development.

**Scheme 2:**
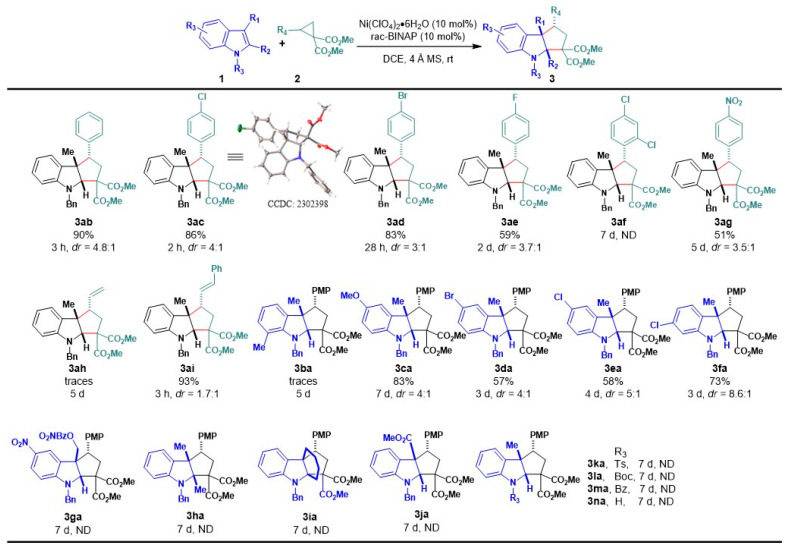
Scope of the reaction ^1, 2, 3^. ^1^ Reaction conditions: Indole **1** (1.0 eq.) and cyclopropane **2** (1.2 eq.), was treated with Ni(ClO_4_)_2_·6H_2_O (10 mol%) and rac-BINAP (10 mol%) in DCE (0.1 M) at rt and time (h). ^2^ Isolated yield. ^3^ Calculated from the ^1^H NMR. h = hours, d = days, ND = not detected.

Then, with optimized conditions in hand, we investigated the scope of the reaction by varying the substitution patterns of both substrates (Scheme 2). Regarding cyclopropanes, diverse functional groups on the aryl ring, such as OMe, H, Cl, Br, F, and NO_2_, were well-tolerated, affording the corresponding products with yields ranging from 51% to 90%, although with lower diastereomer selectivity (**3aa**–**3ag**). In particular, electron–donor groups led to shorter reaction times (2 to 3 h), while strongly attracting substituents like the nitro group significantly extended the reaction time (5 days). However, the use of a cyclopropane bearing a 2,4-dichloro-substituted arene did not lead to the corresponding cycloadduct **3af**, with us instead recovering the starting material. This result may be explained by steric hindrance having occurred between cyclopropane and the catalyst, rather than there being an electronic effect. Importantly, we also evaluated a vinylcyclopropane (VCP), a less stabilized 1,3-dipole system, widely used with palladium catalysis. After 5 days of reaction, only traces of the product **3ah** were observed by mass spectrometry. Inspired by these results, we decided to evaluate a styrylcyclopropane derivative, a more resonance-stabilized VCP. Gratifyingly, this substrate provided cyclopenta[*b*]indole **3ai** at a 93% yield with a *dr* of 1.7:1. Regarding the effect of the indolic ring, substitutions such as OMe, Cl, or Br in positions 5 and 6 were well-tolerated, affording the desired products with good yields and diastereoisomeric ratios of between 8.6:1 to 4:1, albeit with longer reaction times (**3ca**–**3fa**). In contrast, introducing a nitro group in C5, disubstituted C2,C3, or an electron-withdrawing group in the C3 position of the indole did not lead to the desired tricyclic compounds (**3ga**–**3ja**). Similarly, it was observed that the *N*-protection of the indole ring with attractor groups such as Ts, Boc, or Bz did not lead to the formation of the desired skeleton (**3ka**–**3ma**). These results are consistent with a decreased nucleophilicity in the indole ring due to the electronic effects of the abovementioned substituents. On the other hand, incorporating a methyl group at the C7 position of the ring did not provide the desired product (**3ba**), resulting in a complex mixture of products in all cases. This result, along with the case of double substitution in the phenyl ring of cyclopropane (**3af**), indicated that this cycloaddition is also sensitive to steric factors in both reaction components. Finally, we evaluated sktole (deprotected *N*) in the reaction, but the formation of **3na** was not observed and the starting material was recovered. This observation indicated that this Ni(II)-based catalytic system differs from the reaction described by Doyle’s group [49]. The structure of all reaction products was completely characterized by spectroscopic and spectrometric techniques. Additionally, the structure and relative *endo* stereochemistry could be confirmed by X-ray analysis of the **3ac** crystal (CCDC: 2302398) [72].

**Scheme 3:**
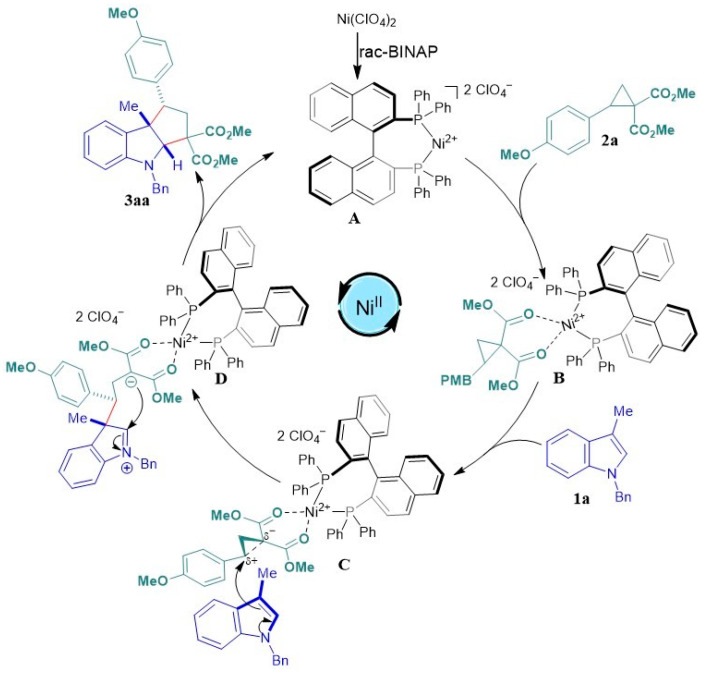
Proposed mechanism for formal [3+2] cycloaddition.

Based on the experimental results and previous reports of [3+2] cycloadditions between donor–acceptor cyclopropanes and indoles [48,49,55], a plausible stepwise mechanism is proposed (Scheme 3). Based on the crystal structure of the cationic BINAP/Ni^2+^ complex (**A**) [73,74] and the labile nature of the perchlorate ion for the formation of metal–ligand complexes [66], we propose that an exchange of the labile ligands of the complex occurs when it interacts with the 1,1-dicarbonyl system of the donor–acceptor cyclopropane **2**. This exchange leads to the formation of intermediate **B**, which, in turn, stabilizes the formation of the polarized species **C**. In view of the steric demand, the nucleophilic attack of the C3 position of indole 1 on the sp^3^ stabilized carbon of cyclopropane, which bears a cationic character (δ^+^), leads to the formation of the intermediate **D** [75,76]. Subsequently, an intramolecular cyclization occurs through the attack of the malonic anion on the iminium ion generating the indole. Finally, the decoordination of the obtained cyclopenta[*b*]indole **3aa** regenerates the catalytic species **A**.

**Figure 2:**
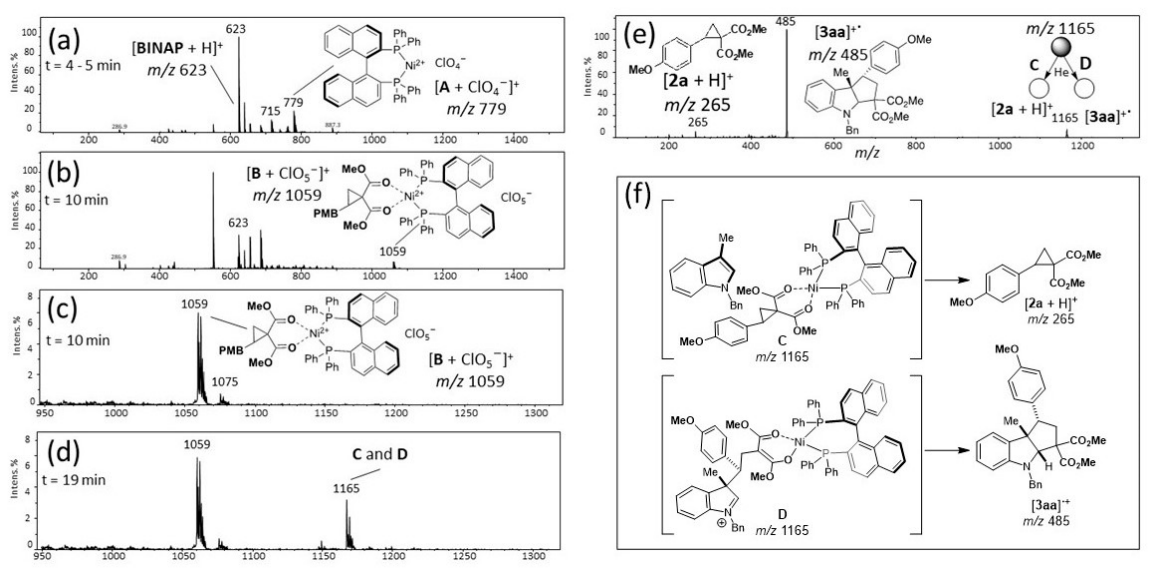
Online ESI-MS monitoring of the reaction of indole **1a** (1.0 eq.) and cyclopropane **2a** (1.2 eq.) treated with Ni(ClO_4_)_2_·6H_2_O (10 mol%) and *rac*-BINAP (10 mol%) in DCE at rt in different reactional times: (**a**) 4–5 min; (**b**) 10 min; (**c**) 10 min, *m*/*z* 950 to 1300; (**d**) 19 min. (**e**) ESI-MS/MS of isobaric intermediates **C** and **D**: C gives **2a**; **D** give final product **3aa**. (**f**) ESI-MS/MS experiments for the characterization of intermediates **C** (that affords **2a**) and **D** (that produces the final product **3aa**), which have the same *m/z*.

To gain further insight into the proposed mechanism, we performed online reaction monitoring by ESI-MS, a technique of great relevance in the advancement of organic catalysis [77,78,79], since molecules and transient intermediates [80] of high polarity and moiety complexity can be easily studied by mass spectrometry. ESI-MS has proven to be a remarkable “ion-fishing” technique, as it gently transfers preformed ions in solution directly to the gas phase. Thus, Figure 2 shows the screening of the reaction of **1a** and **2a** catalyzed with Ni(ClO_4_)_2_⋅6H_2_O (10 mol%) and rac-BINAP (10 mol%) in DCE by online reaction monitoring by ESI-MS. The goal of this study was to intercept the cationic species resulting from the Ni-catalyzed reaction using ESI-MS in the positive-ion mode (Scheme 3) [81,82,83]. The main advantage of using ESI is its capacity to transfer ions to the gas phase without inducing unwanted side reactions, and the composition of ESI-transferred ions often reflects that in solution [77,78,79]. The ESI-MS spectra collected for such a reaction are particularly clean and mechanistically enlightening. Shortly after four to five minutes of reaction of Ni(ClO_4_)_2_⋅6H_2_O and rac-BINAP, two cationic species directly related to the proposed catalytic cycle (Scheme 3) were detected as major ions (Figure 2a): [**A** + ClO_4_^−^]^+^ of *m*/*z* 779, and [**A** + Cl^−^]^+^ of *m*/*z* 715. After 10 min of reaction, **B** was observed as *m*/*z* 1059 (Figure 2b). The isotopic patterns of Ni species were in accordance with the calculated compounds. Then, following the proposed mechanism depicted in Scheme 3, the search began for the species **C** and **D** of *m*/*z* 1165 that have the same *m*/*z*. The formation of intermediates **C** and **D** of *m*/*z* 1165 was observed after 19 min of reaction time, as shown in Figure 2d. The overall appearance of the spectrum and relative intensities of the ions changed little from 19 to 120 min of reaction in solution, as revealed by continuous ESI-MS monitoring. Intermediates **C** and **D** in Figure 2e are isobars, but they have a different bond behavior, as consistent with Scheme 2. Furthermore, the ESI-MS/MS spectrum of the ion of *m*/*z* 1165 did not change with time, showing that these intermediates participate in the catalytic process. However, spectra of samples taken after 20 min to 2 h of reaction (Figure 2e) showed that the ion of *m*/*z* 1165 dissociates in the gas phase mainly into two pathways. A fragment ion of *m*/*z* 265 (**2a**) was formed from intermediate **C**, whereas a dissociated ion of *m*/*z* 485 could be distinguished from intermediate **D**, which afforded the observed final product **3aa**, as depicted in Figure 2e,f. Furthermore, this study demonstrates the important role of BINAP in the catalytic cycle, since its participation in forming all the key intermediates of the proposed mechanism was detected by ESI-MS.

In several studies, it is proposed that cycloadditions with catalyst-activated donor–acceptor cyclopropanes can occur via the S_N_2 or S_N_1 pathway [49,84,85,86,87]. However, according to the ESI-MS experiments and synthetic results in this work, the S_N_1 pathway could not be discarded. Therefore, **C** and **D** are undoubtedly present in the reaction mixture in the course of the reaction. This observation, made according to ESI-MS/MS experiments, suggests that intermediate **D** is the only species that produces the final product **3aa**.

## 3. Experimental Section

### 3.1. General Section

All the reactions were conducted in dry solvents under a N_2_ atmosphere unless otherwise stated. All reagents used were obtained from commercial suppliers and used without further purification. The abbreviation “rt” refers to reactions carried out at approximately 25 °C. Reaction mixtures were stirred using Teflon-coated magnetic stirring bars. Reaction temperatures were maintained using Thermowatch-controlled silicone oil baths. The reactions were monitored by thin-layer chromatography (TLC), which was performed on silica gel Merck 60 F_254_, and the components were visualized by observation under UV light (254 and 365 nm) and/or by treating the plates with *p*-anisaldehyde, oleum, phosphomolybdic acid, or cerium nitrate solutions, followed by heating. Flash chromatography was carried out on silica gel (63–200 µm) unless otherwise stated. Drying was performed with anhydrous Na_2_SO_4_. Concentration refers to the removal of volatile solvents via distillation using a Büchi rotary evaporator R-300 followed by residual solvent removal under a high vacuum. Melting points were determined using a Stuart SMP3 apparatus. Infrared spectra were measured using a Perkin-Elmer FT-IR Spectrometer Spectrum Two (Llantrisant, UK) with KBr pellets. NMR spectra were recorded in CDCl_3_, at 300, 400, 500, or 700 MHz (Bruker Advance III, Oxford, UK). Chemical shifts were reported in parts per million (δ) using the residual solvent signals (CDCl_3_: δ_H_ 7.26, δ_C_ 77.16) as the internal standards for the ^1^H and ^13^C NMR spectra and coupling constants (*J*) in Hz. Carbon types and structure assignments were determined from DEPT-NMR and two-dimensional experiments (HSQC and HMBC, COSY and NOESY). Mass spectra (ESI-MS) were acquired using an Agilent 1200 ESI/APCI Q-TOF in tandem with an Agilent Mass Q-TOF 6520 (Santa Clara, CA, USA). For the crystal structure determination, data were collected by applying the omega and phi scan method on a Bruker D8 VENTURE PHOTON III-14 diffractometer (Karlsurehe, Germany) using an Incoatec multilayer mirror monochromated with Mo-Kα radiation (λ = 0.71073Å) from a microfocus sealed tube source at 100 K, with a detector resolution of 7.3910 pixels mm^−1^. For details of the synthesis and characterization of indoles **1a–m** and donor–acceptor cyclopropanes **2a–i**, see the Supplementary Materials.

### 3.2. General Procedure for [3+2] Cycloaddition (Exemplified for the Synthesis of Dimethyl (1S*,3aR*,8bS*)-4-Benzyl-1-(4-methoxyphenyl)-8b-methyl-1,3a,4,8b-tetrahydrocyclopenta[b]indole-3,3(2H)-dicarboxylate (**3aa**))

A solution of *N*-benzylskatole **1a** (20 mg, 90.37 μmol, 1.0 eq), cyclopropane **2a** (28.7 mg, 108.45 μmol, 1.2 eq), Ni(ClO_4_)_2_·6H_2_O (3.3 mg, 9.04 μmol, 10 mol%), and rac-BINAP (5.63 mg, 9.04 μmol, 10 mol%) and 400 mg of 4 Å molecular sieves in dry DCE (903 μL, 0.1 M) were stirred under a nitrogen atmosphere at room temperature for 5 h. After completing the reaction, as indicated by TLC, the mixture was filtered through celite^®^ and washed AcOEt (50 mL). The filtrate was concentrated and purified by flash chromatography (SiO_2_, 63-200 μm, 10% AcOEt/hexane) to afford cyclopenta[*b*]indole **3aa** (32 mg, 73% yield) as a colorless oil (*dr* = 6:1). **IR** (**KBr**, cm^−1^) **𝜈**: 2994, 2950, 2834, 1731, 1599, 1514, 1275. **^1^H NMR:** (500 MHz, CDCl_3_) δ 7.24–7.17 (m, 2.43H), 7.17–7.10 (m, 2.76H), 7.05 (t, *J* = 9.5 Hz, 0.57H), 6.95 (t, *J* = 7.7 Hz, 0.20H), 6.90–6.78 (m, 2H), 6.73 (d, *J* = 8.2 Hz, 1.84H), 6.63 (d, *J* = 7.2 Hz, 0.14H), 6.59 (t, *J* = 7.3 Hz, 0.14H), 6.36 (d, *J* = 8.0 Hz, 0.14H), 6.32 (d, *J* = 7.9 Hz, 0.86H), 6.28 (t, *J* = 7.4 Hz, 0.86H), 5.47 (d, *J* = 7.5 Hz, 0.86H), 4.58 (d, *J* = 16.5 Hz, 0.18H), 4.55–4.49 (m, 1.78H), 4.25 (d, *J* = 16.3 Hz, 0.16H), 4.08 (d, *J* = 16.1 Hz, 0.89H), 3.75 (s, 0.5H), 3.73 (s, 2.79H), 3.71 (s, 0.36H), 3.69 (s, 2.52H), 3.39 (s, 2.56H), 3.34 (s, 0.44H), 2.90 (dd, *J* = 14.8, 4.6 Hz, 0.82H), 2.68 (dd, *J* = 14.8, 12.7 Hz, 0.90H), 2.60 (dd, *J* = 13.1, 5.7 Hz, 0.16H), 2.30 (t, *J* = 13.3 Hz, 0.21H), 2.26–2.21 (m, 0.94H), 1.26 (s, 2.82H), 1.18 (s, 1.53H). **^13^C NMR**: (126 MHz, CDCl_3_) δ 172.84, 171.97, 169.21, 158.79, 153.45, 150.88, 138.77, 138.62, 137.21, 133.00, 130.82, 130.44, 130.08, 129.75, 128.45, 128.37, 127.86, 127.85, 127.76, 127.59, 127.05, 126.89, 125.95, 118.39, 118.21, 114.14, 113.99, 113.73, 113.50, 113.25, 109.67, 109.54, 82.33, 80.19, 65.30, 64.12, 57.57, 56.66, 55.39, 55.37, 55.13, 53.74, 53.41, 52.90, 52.55, 52.44, 39.36, 36.87, 29.83, 28.15. **HRMS** (ESI): Calculated for C_30_H_32_NO_5_ [M + H]^+^ = 486.2275; found: 486.2286. NMR data of the major isomer deduced from the 6:1 mixture: **^1^H NMR:** (500 MHz, CDCl_3_) δ 7.24–7.09 (m, 6H), 6.89–6.78 (m, 2H), 6.73 (d, *J* = 8.3 Hz, 2H), 6.34–6.25 (m, 2H), 5.47 (d, *J* = 7.5 Hz, 1H), 4.52 (d, 2H), 4.08 (d, *J* = 16.1 Hz, 1H), 3.73 (s, 3H), 3.69 (s, 3H), 3.39 (s, 3H), 2.90 (dd, *J* = 14.8, 4.6 Hz, 1H), 2.73–2.63 (m, 1H), 2.23 (dd, *J* = 13.0, 4.4 Hz, 1H), 1.26 (s, 3H). **^13^C NMR:** (126 MHz, CDCl_3_) δ 171.97, 169.21, 158.79, 153.45, 138.77, 133.00, 130.44, 130.08, 128.45, 128.37, 127.86, 127.83, 127.76, 127.59, 127.05, 125.95, 118.21, 113.73, 113.50, 113.25, 109.67, 80.19, 65.30, 57.57, 56.66, 55.39, 55.37, 52.90, 52.55, 52.44, 36.87, 29.83, 28.15. The spectroscopic data agreed with the literature [48].

#### 3.2.1. Dimethyl (1S*,3aR*,8bS*)-4-Benzyl-8b-methyl-1-phenyl-1,3a,4,8b-tetrahydrocyclopenta[b]indole-3,3(2H)-dicarboxylate (**3ab**)

Colorless oil (37 mg, 90%, *dr* = 4.8:1) from *N*-benzylskatole **1a** (20 mg, 90.37 μmol) and cyclopropane **2b** (25.4 mg, 108.45 μmol). **IR** (**KBr**, cm^−1^) **𝜈**: 3026, 2949, 2849, 1731, 1599, 1484, 1452, 1264, 698. **^1^H NMR**: (700 MHz, CDCl_3_) δ 7.34 (t, *J* = 7.4 Hz, 0.74H), 7.32–7.25 (m, 3.26H), 7.24–7.21 (m, 1.38H), 7.21–7.18 (m, 1.69H), 7.08–7.01 (m, 1.67H), 7.02 (s, 0.21H), 6.91 (td, *J* = 7.6, 1.3 Hz, 0.95H), 6.72 (dd, *J* = 7.4, 1.4 Hz, 0.30H), 6.68 (d, *J* = 7.3 Hz, 0.32H), 6.45 (d, *J* = 8.0 Hz, 0.33H), 6.40 (d, *J* = 7.9 Hz, 0.81H), 6.35–6.30 (m, 0.85H), 5.49–5.44 (m, 0.74H), 4.71 (s, 0.26H), 4.66 (d, *J* = 16.3 Hz, 0.43H), 4.63 (d, *J* = 1.5 Hz, 0.71H), 4.60 (d, *J* = 16.1 Hz, 0.79H), 4.34 (d, *J* = 16.3 Hz, 0.35H), 4.16 (d, *J* = 16.0 Hz, 0.80H), 3.81 (s, 0.81H), 3.78 (s, 2.22H), 3.47 (s, 2.22H), 3.42 (s, 0.72H), 3.02 (dd, *J* = 14.8, 4.7 Hz, 0.79H), 2.82 (dd, *J* = 14.9, 12.8 Hz, 0.88H), 2.71 (dd, *J* = 13.1, 5.8 Hz, 0.37H), 2.44 (t, *J* = 13.4 Hz, 0.42H), 2.37–2.32 (m, 1H), 1.37 (s, 2.15H), 0.94 (s, 0.87H). **^13^C NMR**: (176 MHz, CDCl_3_) δ 172.70, 171.84, 170.28, 170.22, 169.04, 167.07, 153.34, 150.77, 138.73, 138.65, 138.49, 138.27, 136.95, 134.61, 132.77, 129.10, 129.07, 128.64, 128.61, 128.46, 128.43, 128.35, 128.28, 128.27, 128.25, 128.20, 127.99, 127.80, 127.76, 127.64, 127.58, 127.46, 127.43, 127.34, 127.01, 126.94, 126.91, 126.78, 125.79, 125.68, 124.85, 122.79, 118.30, 118.07, 109.60, 109.45, 82.28, 80.18, 65.20, 64.06, 57.55, 56.60, 55.08, 53.97, 53.61, 53.14, 52.83, 52.81, 52.58, 52.46, 52.36, 52.23, 38.99, 36.44, 32.59, 29.72, 28.06, 22.92, 19.14. **HRMS** (ESI): Calculated for C_29_H_30_NO_4_ [M + H]^+^ = 456.2169; found: 456.2181. NMR data of the major isomer deduced from the 4.8:1 mixture: **^1^H NMR**: (700 MHz, CDCl_3_) δ 7.32–7.25 (m, 4H), 7.24–7.21 (m, 2H), 7.21–7.18 (m, 2H), 7.08–7.01 (m, 2H), 6.91 (td, *J* = 7.6, 1.3 Hz, 1H), 6.40 (d, *J* = 7.9 Hz, 1H), 6.35–6.30 (m, 1H), 5.49–5.44 (m, 1H), 4.63 (d, *J* = 1.5 Hz, 1H), 4.60 (d, *J* = 16.1 Hz, 1H), 4.16 (d, *J* = 16.0 Hz, 1H), 3.78 (s, 3H), 3.47 (s, 3H), 3.02 (dd, *J* = 14.8, 4.7 Hz, 1H), 2.82 (dd, *J* = 14.9, 12.8 Hz, 1H), 2.37–2.32 (m, 1H), 1.37 (s, 3H). **^13^C NMR**: (176 MHz, CDCl_3_) δ 171.84, 169.04, 153.34, 138.65, 138.27, 132.77, 129.10, 129.07, 128.46, 128.35, 128.27, 128.20, 127.99, 127.80, 127.76, 127.64, 127.46, 127.01, 126.94, 126.78, 125.68, 122.79, 118.30, 118.07, 109.60, 109.45, 80.18, 65.20, 57.55, 56.60, 53.14, 52.83, 52.81, 52.36, 36.44, 28.06. The spectroscopic data agreed with the literature [48].

#### 3.2.2. Dimethyl (1S*,3aR*,8bS*)-4-Benzyl-1-(4-chlorophenyl)-8b-methyl-1,3a,4,8b-tetrahydrocyclopenta[b]indole-3,3(2H)-dicarboxylate (**3ac**)

White solid (38.1 mg, 86%, *dr* = 4:1) from *N*-benzylskatole **1a** (20 mg, 90.37 μmol) and cyclopropane **2c** (29.2 mg, 108.45 μmol). **mp:** 117–120 °C. **IR** (**KBr**, cm^−1^) **𝜈**: 3026, 2949, 2849, 1731, 1599, 1484, 1452, 1264, 698. **^1^H NMR:** (700 MHz, CDCl_3_) δ 7.34–7.25 (m, 3H), 7.23 (d, *J* = 8.2 Hz, 2H), 7.18 (d, *J* = 7.7 Hz, 1.39H), 7.04 (d, *J* = 7.7 Hz, 0.41H), 7.00–6.91 (m, 1.42H), 6.45 (d, *J* = 8.0 Hz, 0.24H), 6.41 (d, *J* = 8.0 Hz, 0.70H), 6.38 (t, *J* = 7.4 Hz, 0.69H), 5.53 (d, *J* = 7.5 Hz, 0.76H), 4.71 (s, 0.19H), 4.65 (d, *J* = 16.3 Hz, 0.30H), 4.62 (s, 0.68H), 4.59 (d, *J* = 16.1 Hz, 0.71H), 4.32 (d, *J* = 16.3 Hz, 0.22H), 4.15 (d, *J* = 16.1 Hz, 0.76H), 3.81 (s, 0.57H), 3.78 (s, 2.08H), 3.47 (s, 2.11H), 3.42 (s, 0.58H), 2.98 (dd, *J* = 14.8, 4.7 Hz, 0.75H), 2.76 (dd, *J* = 14.7, 12.8 Hz, 0.82H), 2.69 (dd, *J* = 13.1, 5.7 Hz, 0.22H), 2.39 (d, *J* = 13.5 Hz, 0.27H), 2.31 (dd, *J* = 12.8, 4.7 Hz, 0.75H), 1.34 (s, 2.18H), 0.93 (s, 0.58H). **^13^C NMR:** (176 MHz, CDCl_3_) δ 172.65, 171.84, 170.25, 169.03, 153.50, 150.95, 138.65, 138.51, 137.31, 136.97, 136.68, 132.94, 132.90, 132.51, 130.52, 130.45, 129.04, 128.79, 128.56, 128.50, 128.41, 128.28, 128.12, 128.07, 128.02, 127.93, 127.74, 127.58, 127.16, 127.12, 126.95, 125.87, 125.73, 124.98, 122.73, 122.19, 119.44, 118.63, 118.54, 118.37, 109.91, 109.81, 109.60, 82.38, 80.20, 65.27, 64.09, 57.63, 56.75, 55.18, 53.90, 53.56, 53.00, 52.82, 52.79, 52.69, 52.65, 52.53, 49.71, 47.05, 39.13, 36.74, 32.08, 29.85, 29.81, 28.07, 22.84, 22.72. **HRMS** (ESI): Calculated for C_29_H_29_ClNO_4_ [M + H]^+^ = 490.1780; found: 490.1796. NMR data of the major isomer deduced from the 4:1 mixture: **^1^H NMR**: (700 MHz, CDCl_3_) δ 7.34–7.25 (m, 4H), 7.23 (d, *J* = 8.2 Hz, 3H), 7.18 (d, *J* = 7.7 Hz, 2H), 7.00–6.91 (m, 1H), 6.41 (d, *J* = 8.0 Hz, 1H), 6.38 (t, *J* = 7.4 Hz, 1H), 5.53 (d, *J* = 7.5 Hz, 1H), 4.62 (s, 1H), 4.59 (d, *J* = 16.1 Hz, 1H), 4.15 (d, *J* = 16.1 Hz, 1H), 3.78 (s, 3H), 3.47 (s, 3H), 2.98 (dd, *J* = 14.8, 4.7 Hz, 1H), 2.76 (dd, *J* = 14.7, 12.8 Hz, 1H), 2.31 (dd, *J* = 12.8, 4.7 Hz, 1H), 1.34 (s, 3H). **^13^C NMR:** (176 MHz, CDCl_3_) δ 171.72, 168.90, 153.37, 138.52, 136.85, 132.81, 132.38, 130.39, 130.32, 128.67, 128.37, 128.28, 128.15, 127.99, 127.89, 127.62, 127.46, 126.99, 125.60, 118.24, 109.79, 80.07, 65.14, 57.50, 56.62, 52.87, 52.69, 52.41, 36.61, 27.94. Further confirmation of the structure of **3ac** was obtained by X-ray crystallography (CCDC: 2302398) of single crystals obtained from a hexane/EtOAc (9:1) mixture [72]. 
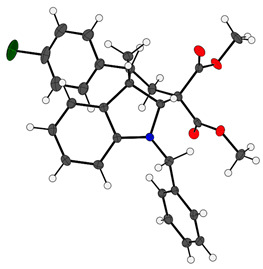


#### 3.2.3. Dimethyl (1S*,3aR*,8bS*)-4-Benzyl-1-(4-bromophenyl)-8b-methyl-1,3a,4,8b-tetrahydrocyclopenta[b]indole-3,3(2H)-dicarboxylate (**3ad**)

Yellow solid (40.1 mg, 83%, *dr* = 3:1) from *N*-benzylskatole **1a** (20 mg, 90.37 μmol) and cyclopropane **2d** (34 mg, 108.45 μmol). **IR** (**KBr**, cm^−1^) **𝜈**: 2945, 2913, 2863, 1731, 1594, 1487, 1271, 756. **^1^H NMR**: (700 MHz, CDCl_3_) δ 7.48–7.45 (m, 0.32H), 7.38 (d, *J* = 8.2 Hz, 1.59H), 7.31–7.25 (m, 1.76H), 7.24–7.21 (m, 0.85H), 7.18 (d, *J* = 7.7 Hz, 1.45H), 7.09 (dd, *J* = 8.4, 1.6 Hz, 0.30H), 7.08–7.06 (m, 0.17H), 7.04 (dd, *J* = 8.3, 5.2 Hz, 0.23H), 6.99 (d, *J* = 8.0 Hz, 0.11H), 6.94 (t, *J* = 7.8 Hz, 0.68H), 6.90 (d, *J* = 7.9 Hz, 1.09H), 6.80 (s, 0.16H), 6.68–6.67 (m, 0.28H), 6.45 (d, *J* = 8.1 Hz, 0.17H), 6.41 (d, *J* = 8.0 Hz, 0.58H), 6.38 (t, *J* = 7.4 Hz, 0.60H), 5.54 (d, *J* = 7.5 Hz, 0.54H), 4.71 (d, *J* = 1.5 Hz, 0.16H), 4.65 (d, *J* = 16.2 Hz, 0.18H), 4.62 (s, 0.58H), 4.59 (d, *J* = 16.0 Hz, 0.64H), 4.32 (d, *J* = 16.3 Hz, 0.17H), 4.15 (d, *J* = 16.0 Hz, 0.62H), 3.81 (s, 0.51H), 3.78 (s, 2.05H), 3.47 (d, *J* = 1.7 Hz, 1.84H), 3.42 (s, 0.64H), 2.97 (dd, *J* = 14.7, 4.6 Hz, 0.68H), 2.75 (t, *J* = 12.9 Hz, 0.70H), 2.69 (dd, *J* = 13.1, 5.5 Hz, 0.17H), 2.39 (d, *J* = 13.5 Hz, 0.15H), 2.31 (dd, *J* = 12.8, 4.6 Hz, 0.64H), 1.34 (s, 1.87H), 0.93 (s, 0.45H). **^13^C NMR**: (176 MHz, CDCl_3_) δ 172.63, 171.83, 170.23, 169.01, 153.49, 150.95, 138.63, 138.50, 137.83, 137.49, 136.65, 132.47, 131.74, 131.47, 131.23, 130.96, 130.91, 130.84, 130.33, 129.41, 128.80, 128.55, 128.50, 128.47, 128.41, 128.13, 128.08, 127.93, 127.74, 127.58, 127.50, 127.12, 126.95, 125.87, 125.73, 122.73, 121.06, 121.02, 118.55, 118.39, 109.92, 109.82, 82.38, 80.18, 65.27, 64.08, 57.59, 56.74, 55.14, 53.90, 53.62, 53.04, 53.00, 52.89, 52.69, 52.66, 52.56, 52.53, 49.70, 39.05, 36.69, 31.95, 29.85, 28.06, 22.71 **HRMS** (ESI): Calculated for C_29_H_29_BrNO_4_ [M + H]^+^ = 534.1274; found: 534.1257. NMR data of the major isomer deduced from the 3:1 mixture: **^1^H NMR**: (700 MHz, CDCl_3_) δ 7.38 (d, *J* = 8.2 Hz, 2H), 7.31–7.25 (m, 3H), 7.24–7.21 (m, 1H), 7.18 (d, *J* = 7.7 Hz, 2H), 6.94 (t, *J* = 7.8 Hz, 1H), 6.90 (d, *J* = 7.9 Hz, 1H), 6.41 (d, *J* = 8.0 Hz, 1H), 6.38 (t, *J* = 7.4 Hz, 1H), 5.54 (d, *J* = 7.5 Hz, 1H), 4.62 (s, 1H), 4.59 (d, *J* = 16.0 Hz, 1H), 4.15 (d, *J* = 16.0 Hz, 1H), 3.78 (d, *J* = 1.6 Hz, 3H), 3.47 (s, 3H), 2.97 (dd, *J* = 14.7, 4.6 Hz, 1H), 2.75 (t, *J* = 12.9 Hz, 1H), 2.31 (dd, *J* = 12.8, 4.6 Hz, 1H), 1.34 (s, 3H). **^13^C NMR:** (176 MHz, CDCl_3_) δ 171.83, 169.01, 153.49, 138.63, 137.49, 132.47, 131.23, 130.96, 130.91, 130.84, 128.50, 128.41, 128.13, 127.74, 127.58, 127.12, 125.73, 121.06, 118.39, 109.92, 80.18, 65.27, 57.59, 56.74, 53.00, 52.89, 52.53, 36.69, 28.06.

#### 3.2.4. Dimethyl (1S*,3aR*,8bS*)-4-Benzyl-1-(4-fluorophenyl)-8b-methyl-1,3a,4,8b-tetrahydrocyclopenta[b]indole-3,3(2H)-dicarboxylate (**3ae**)

White solid (25.3 mg, 59%, *dr* = 3.7:1) from *N*-benzylskatole **1a** (20 mg, 90.37 μmol) and cyclopropane **2e** (27.4mg, 108.45 μmol). **IR** (**KBr**, cm^−1^) **𝜈**: 2995, 2995, 2920, 2848, 1731, 1600, 1513, 1273, 1222. **^1^H NMR**: (700 MHz, CDCl_3_) δ 7.31–7.27 (m, 1.70H), 7.22 (t, *J* = 7.3 Hz, 0.91H), 7.20–7.15 (m, 3.11H), 7.11 (d, *J* = 7.7 Hz, 0.43H), 7.03 (t, *J* = 8.4 Hz, 0.68H), 6.96 (dd, *J* = 9.8, 7.5 Hz, 3.59H), 6.94–6.89 (m, 1.25H), 6.69–6.65 (m, 0.34H), 6.45 (d, *J* = 8.0 Hz, 0.31H), 6.40 (d, *J* = 8.0 Hz, 0.68H), 6.36 (td, *J* = 7.2, 2.9 Hz, 0.70H), 5.49 (d, *J* = 7.0 Hz, 0.64H), 4.71 (s, 0.23H), 4.65 (d, *J* = 16.3 Hz, 0.38H), 4.62 (s, 0.56H), 4.59 (d, *J* = 16.0 Hz, 0.68H), 4.32 (d, *J* = 16.3 Hz, 0.36H), 4.15 (d, *J* = 16.1 Hz, 0.74H), 3.81 (s, 0.49H), 3.79 (s, 2.26H), 3.42 (s, 0.49H), 3.39 (s, 2.26H), 2.99 (dd, *J* = 14.8, 4.6 Hz, 0.73H), 2.75 (dd, *J* = 14.8, 12.8 Hz, 0.78H), 2.69 (dd, *J* = 13.1, 5.6 Hz, 0.35H), 2.37 (t, *J* = 13.5 Hz, 0.39H), 2.35–2.29 (m, 0.79H), 1.34 (s, 1.58H), 0.92 (s, 0.53H). **^13^C NMR:** (176 MHz, CDCl_3_) δ 171.88, 170.26, 169.08, 167.11, 162.29 (d, *J* = 246.13 Hz), 162.18 (d, *J* = 245.07 Hz)153.53, 138.69, 134.15, 132.65, 130.30 (d, *J* = 8.17 Hz), 128.50, 128.41, 128.06, 128.02, 127.75, 127.58, 127.11, 126.94, 125.74, 118.51, 118.31, 115.27 (d, *J* = 21.59 Hz), 114.70 (d, *J* = 21.00 Hz), 109.87, 109.77, 82.37, 80.21, 65.26, 64.08, 57.61, 56.76, 53.88, 53.42, 53.00, 52.99, 52.66, 52.64, 52.52, 52.46, 37.23, 36.87, 31.91, 29.86, 28.07, 19.39. **HRMS** (ESI): Calculated for C_29_H_29_FNO_4_ [M + H]^+^ = 474.2075l; found: 474.2052. NMR data of the major isomer deduced from the 3.7:1 mixture: **^1^H NMR**: (700 MHz, CDCl_3_) δ 7.30–7.27 (m, 2H), 7.20–7.14 (m, 4H), 6.96 (t, *J* = 8.6 Hz, 4H), 6.40 (d, *J* = 8.0 Hz, 1H), 6.36 (t, *J* = 7.2 Hz, 1H), 5.51–5.48 (m, 1H), 4.62 (s, 1H), 4.59 (d, *J* = 16.0 Hz, 1H), 4.15 (d, *J* = 16.1 Hz, 1H), 3.79 (s, 3H), 3.39 (s, 3H), 2.99 (dd, *J* = 14.8, 4.6 Hz, 1H), 2.75 (dd, *J* = 14.8, 12.8 Hz, 1H), 2.35–2.28 (m, 1H), 1.34 (s, 3H). **^13^C NMR**: (176 MHz, CDCl_3_) δ 171.88, 170.26, 169.08, 167.11, 162.29 (d, *J* = 246.1 Hz), 153.53, 138.69, 132.65, 130.47, 130.45, 130.30 (d, *J* = 8.2 Hz), 128.50, 128.06, 127.75, 127.58, 127.11, 125.74, 118.31, 115.27 (d, *J* = 21.6 Hz), 114.76, 114.64, 109.87, 80.21, 65.26, 57.61, 56.76, 53.00, 52.99, 52.66, 52.52, 52.46, 37.23, 36.87, 31.91, 28.07, 19.39.

#### 3.2.5. Dimethyl (1S*,3aR*,8bS*)-4-Benzyl-1-(4-nitrophenyl)-8b-methyl-1,3a,4,8b-tetrahydrocyclopenta[b]indole-3,3(2H)-dicarboxylate (**3ag**)

Yellow solid (23.1 mg, 51%, *dr* = 3.5:1) from *N*-benzylskatole **1a** (20 mg, 90.37 μmol) and cyclopropane **2g** (30.3 mg, 108.45 μmol). **mp:** 134–140 °C. **IR** (**KBr**, cm^−1^) **𝜈**: 3077, 3022, 2948, 1728, 1599, 1515, 1346. ^**1**^**H NMR**: (700 MHz, CDCl_3_) δ 8.15 (d, *J* = 8.5 Hz, 1.61H), 8.12 (d, *J* = 8.3 Hz, 1.15H), 7.36 (d, *J* = 8.3 Hz, 1.77H), 7.29 (t, *J* = 7.5 Hz, 1.81H), 7.23 (t, *J* = 7.4 Hz, 1.22H), 7.18 (t, *J* = 5.9 Hz, 2.85H), 6.97–6.92 (m, 0.79H), 6.70 (t, *J* = 7.4 Hz, 0.22H), 6.63 (d, *J* = 7.4 Hz, 0.18H), 6.48 (d, *J* = 8.0 Hz, 0.20H), 6.44 (d, *J* = 8.0 Hz, 0.73H), 6.34 (t, *J* = 7.4 Hz, 0.71H), 5.44 (d, *J* = 7.5 Hz, 0.59H), 4.73 (s, 0.17H), 4.66 (d, *J* = 16.6 Hz, 0.30H), 4.64 (s, 0.62H), 4.60 (d, *J* = 16.0 Hz, 0.76H), 4.32 (d, *J* = 16.3 Hz, 0.23H), 4.15 (d, *J* = 16.0 Hz, 0.78H), 3.83 (s, 0.71H), 3.81 (s, 2.34H), 3.53 (s, 0.34H), 3.42 (s, 2.69H), 3.12 (dd, *J* = 14.7, 4.8 Hz, 0.71H), 2.83 (dd, *J* = 14.7, 12.8 Hz, 0.85H), 2.74 (dd, *J* = 13.0, 5.5 Hz, 0.26H), 2.45 (t, *J* = 13.4 Hz, 0.28H), 2.39–2.34 (m, 0.78H), 1.38 (s, 2.16H), 0.94 (s, 0.49H). **^13^C NMR**: (176 MHz, CDCl_3_) δ 171.63, 170.09, 169.68, 168.78, 166.65, 153.55, 147.39, 147.26, 147.23, 146.51, 142.60, 138.44, 131.89, 130.06, 129.92, 129.49, 128.78, 128.55, 128.53, 128.48, 128.45, 128.41, 127.73, 127.57, 127.31, 127.22, 127.04, 125.87, 125.28, 124.97, 123.80, 123.56, 123.33, 123.04, 122.59, 119.69, 118.78, 118.73, 118.51, 110.28, 110.16, 82.37, 80.21, 65.28, 64.14, 58.07, 56.82, 55.60, 54.04, 54.00, 53.44, 53.25, 53.11, 52.91, 52.80, 52.78, 52.74, 52.70, 52.64, 49.57, 47.11, 38.97, 37.86, 36.70, 31.71, 29.84, 29.81, 28.03, 22.55, 19.49, 9.79, 1.16. **HRMS** (ESI): Calculated for C_29_H_29_N_2_O_6_ [M + H]^+^ = 501.2020; found: 501.1997. NMR data of the major isomer deduced from the 3.5:1 mixture: **^1^H NMR**: (700 MHz, CDCl_3_) δ 8.15 (d, *J* = 8.5 Hz, 2H), 7.36 (d, *J* = 8.3 Hz, 2H), 7.29 (t, *J* = 7.5 Hz, 2H), 7.18 (t, *J* = 5.9 Hz, 3H), 6.97–6.92 (m, 1H), 6.44 (d, *J* = 8.0 Hz, 1H), 6.34 (t, *J* = 7.4 Hz, 1H), 5.44 (d, *J* = 7.5 Hz, 1H), 4.64 (s, 1H), 4.60 (d, *J* = 16.0 Hz, 1H), 4.15 (d, *J* = 16.0 Hz, 1H), 3.80 (s, 3H), 3.49 (s, 3H), 3.12 (dd, *J* = 14.7, 4.8 Hz, 1H), 2.83 (dd, *J* = 14.7, 12.8 Hz, 1H), 2.39–2.34 (m, 1H), 1.38 (s, 3H). **^13^C NMR**: (176 MHz, CDCl_3_) δ 171.63, 169.68, 168.78, 166.65, 153.55, 146.51, 142.60, 138.44, 131.89, 130.06, 129.49, 128.55, 128.48, 128.45, 127.57, 127.22, 125.28, 123.56, 123.04, 118.51, 110.28, 80.21, 65.28, 58.07, 56.82, 53.44, 53.25, 53.11, 52.74, 52.64, 37.86, 36.70, 31.71, 29.84, 28.03, 19.49, 1.16.

#### 3.2.6. Dimethyl (1S*,3aR*,8bS*)-4-Benzyl-8b-methyl-1-((E)-styryl)-1,3a,4,8b-tetrahydrocyclopenta[b]indole-3,3(2H)-dicarboxylate (**3ai**)

White solid (40.4 mg, 93%, *dr* = 1.7:1) from *N*-benzylskatole **1a** (20 mg, 90.37 μmol) and cyclopropane **2i** (28.2 mg, 108.45 μmol). **IR** (**KBr**, cm^−1^) **𝜈**: 3023, 2949, 2916, 2847, 1731, 1598, 1483, 1259. **^1^H NMR**: (700 MHz, CDCl_3_) δ 7.41–7.39 (m, 0.65H), 7.36 (d, *J* = 7.1 Hz, 1.48H), 7.34–7.32 (m, 1.76H), 7.29–7.27 (m, 2.58H), 7.25–7.20 (m, 2.25H), 7.19 (d, *J* = 7.6 Hz, 1.59H), 7.17–7.14 (m, 0.45H), 7.11 (t, *J* = 7.6 Hz, 0.69H), 7.08–7.05 (m, 0.30H), 7.05–7.01 (m, 0.89H), 6.99 (dd, *J* = 7.3, 1.3 Hz, 0.32H), 6.87–6.86 (m, 0.65H), 6.69 (q, *J* = 7.2 Hz, 0.92H), 6.50–6.43 (m, 1.84H), 6.25 (dd, *J* = 15.8, 8.6 Hz, 0.34H), 6.02 (dd, *J* = 15.8, 8.8 Hz, 0.65H), 4.63 (s, 0.40H), 4.59 (s, 0.60H), 4.58 (d, *J* = 6.7 Hz, 0.59H), 4.32 (d, *J* = 16.2 Hz, 0.31H), 4.17 (d, *J* = 16.1 Hz, 0.65H), 3.79 (s, 3H), 3.46 (s, 0.78H), 3.45 (s, 1.69H), 2.64 (dd, *J* = 13.23, 6.08 Hz, 0.31H), 2.51 (ddd, *J* = 13.8, 8.8, 4.9 Hz, 0.67H), 2.42 (t, *J* = 13.3 Hz, 0.76H), 2.27 (ddd, *J* = 12.7, 4.9, 1.5 Hz, 0.68H), 2.04 (t, *J* = 12.6 Hz, 0.36H), 1.33 (s, 1.96H), 1.26 (s, 0.81H). **^13^C NMR**: (176 MHz, CDCl_3_) δ 172.73, 171.89, 170.24, 169.06, 153.43, 150.58, 138.70, 138.48, 137.54, 137.52, 137.38, 133.20, 132.21, 131.31, 129.87, 128.79, 128.74, 128.49, 128.41, 128.24, 128.20, 128.18, 128.15, 127.89, 127.53, 127.46, 127.06, 127.00, 126.37, 126.35, 125.55, 122.13, 121.74, 118.67, 118.60, 110.16, 109.35, 81.71, 80.16, 77.34, 77.16, 76.98, 72.26, 65.81, 65.36, 57.61, 56.59, 55.91, 53.25, 52.94, 52.92, 52.81, 52.53, 52.45, 52.03, 40.02, 38.35, 29.85, 27.74, 22.88. **HRMS** (ESI): Calculated for C_30_H_32_NO_4_ [M + H]^+^ = 482.2326; found: 482.2298. NMR data of the major isomer deduced from the 1.7:1 mixture: **^1^H NMR**: (700 MHz, CDCl_3_) δ 7.41–7.39 (m, 1H), 7.36 (d, *J* = 7.1 Hz, 2H), 7.34–7.32 (m, 2H), 7.29–7.27 (m, 3H), 7.19 (d, *J* = 7.6 Hz, 2H), 6.87–6.86 (m, 1H), 6.69 (q, *J* = 7.2 Hz, 1H), 6.50–6.43 (m, 2H), 6.02 (dd, *J* = 15.8, 8.8 Hz, 1H), 4.59 (d, *J* = 1.4 Hz, 1H), 4.58 (d, *J* = 6.7 Hz, 1H), 4.17 (d, *J* = 16.1 Hz, 1H), 3.79 (s, 3H), 3.45 (s, 3H), 2.51 (ddd, *J* = 13.8, 8.8, 4.9 Hz, 1H), 2.42 (t, *J* = 13.3 Hz, 1H), 2.27 (ddd, *J* = 12.7, 4.9, 1.5 Hz, 1H), 1.33 (s, 3H). **^13^C NMR**: (176 MHz, CDCl_3_) δ 171.89, 169.06, 153.43, 138.70, 137.38, 133.20, 132.21, 131.31, 129.87, 128.79, 128.49, 127.53, 126.35, 125.55, 122.13, 118.67, 110.16, 80.16, 65.81, 57.61, 56.59, 52.94, 52.45, 52.03, 38.35, 27.74.

#### 3.2.7. Dimethyl (1S*,3aR*,8bS*)-4-Benzyl-7-methoxy-1-(4-methoxyphenyl)-8b-methyl-1,3a,4,8b-tetrahydrocyclopenta[b]indole-3,3(2H)-dicarboxylate (**3ca**)

Colorless oil (34 mg, 83%, *dr* = 4:1) from *N*-benzylskatole **1c** (20 mg, 79.58 μmol) and cyclopropane **2a** (25.2 mg, 95.49 μmol). **IR** (**KBr**, cm^−1^) **𝜈**: 2951, 2916, 2833, 1731, 1610, 1514, 1261, 1013. **^1^H NMR**: (700 MHz, CDCl_3_) δ 7.30–7.27 (m, 4H), 7.24–7.18 (m, 4H), 7.13 (dd, *J* = 9.6, 2.9 Hz, 0.61H), 6.96 (d, *J* = 7.4 Hz, 1.33H), 6.87 (d, *J* = 8.6 Hz, 0.46H), 6.81 (d, *J* = 8.4 Hz, 1.45H), 6.58 (dd, *J* = 8.5, 2.7 Hz, 0.22H), 6.49 (dd, *J* = 8.6, 2.7 Hz, 0.72H), 6.32 (d, *J* = 8.6 Hz, 0.22H), 6.30 (d, *J* = 8.6 Hz, 0.68H), 6.28 (d, *J* = 2.7 Hz, 0.18H), 5.12 (d, *J* = 2.7 Hz, 0.70H), 4.69 (s, 0.22H), 4.60 (d, *J* = 16.2 Hz, 0.25H), 4.57 (s, 0.70H), 4.52 (d, *J* = 15.8 Hz, 0.80H), 4.23 (d, *J* = 16.1 Hz, 0.27H), 4.09 (d, *J* = 15.8 Hz, 0.81H), 3.82 (s, 0.67H), 3.79 (s, 2.25H), 3.77 (s, 1.94H), 3.68 (s, 0.56H), 3.48 (s, 1.97H), 3.40 (s, 0.57H), 3.34 (s, 2.22H), 2.97 (dd, *J* = 14.8, 4.6 Hz, 0.75H), 2.76 (dd, *J* = 14.8, 12.8 Hz, 0.82H), 2.71 (dd, *J* = 13.1, 5.7 Hz, 0.25H), 2.41–2.36 (m, 0.38H), 2.34–2.25 (m, 0.95H), 1.31 (s, 2.48H), 0.93 (s, 0.56H). **^13^C NMR**: (176 MHz, CDCl_3_) δ 172.91, 172.07, 170.36, 169.20, 158.89, 158.77, 153.28, 152.76, 147.62, 145.34, 139.03, 138.91, 138.39, 134.37, 130.62, 130.49, 130.09, 130.02, 129.90, 129.76, 129.49, 129.15, 128.84, 128.44, 128.34, 127.81, 127.79, 127.54, 127.34, 127.04, 126.83, 124.98, 114.57, 114.15, 113.74, 113.48, 113.36, 113.30, 112.67, 111.34, 110.81, 110.41, 109.92, 83.14, 80.82, 65.43, 64.01, 58.30, 57.67, 56.02, 55.69, 55.47, 55.45, 55.42, 55.40, 53.41, 52.92, 52.91, 52.72, 52.58, 52.53, 52.44, 39.46, 36.67, 29.85, 27.92, 22.38, 14.27. **HRMS** (ESI): Calculated for C_31_H_34_NO_6_ [M + H]^+^ = 516.2381; found: 516.2376. NMR data of the major isomer deduced from the 4:1 mixture: **^1^H NMR**: (700 MHz, CDCl_3_) δ 7.30–7.27 (m, 3H), 7.23–7.18 (m, 3H), 6.96 (d, *J* = 7.5 Hz, 2H), 6.81 (d, *J* = 8.4 Hz, 2H), 6.49 (dd, *J* = 8.6, 2.7 Hz, 1H), 6.30 (d, *J* = 8.6 Hz, 1H), 5.12 (d, *J* = 2.7 Hz, 1H), 4.56 (d, *J* = 1.5 Hz, 1H), 4.52 (d, *J* = 15.8 Hz, 1H), 4.09 (d, *J* = 15.8 Hz, 1H), 3.79 (s, 3H), 3.77 (s, 3H), 3.48 (s, 3H), 3.34 (s, 3H), 2.97 (dd, *J* = 14.8, 4.6 Hz, 1H), 2.76 (dd, *J* = 14.8, 12.7 Hz, 1H), 2.30 (ddt, *J* = 15.5, 4.3, 2.4 Hz, 1H), 1.31 (s, 3H). **^13^C NMR**: (176 MHz, CDCl_3_) δ 172.07, 169.20, 158.89, 152.76, 147.62, 139.03, 134.37, 130.49, 130.09, 128.44, 128.34, 127.81, 127.79, 127.04, 114.57, 113.48, 113.36, 111.34, 110.81, 80.82, 65.43, 58.30, 57.67, 55.69, 55.47, 55.40, 52.91, 52.53, 52.44, 36.67, 29.85, 27.92. The spectroscopic data agreed with the literature [48].

#### 3.2.8. Dimethyl (1S*,3aR*,8bS*)-4-Benzyl-7-bromo-1-(4-methoxyphenyl)-8b-methyl-1,3a,4,8b-tetrahydrocyclopenta[b]indole-3,3(2H)-dicarboxylate (**3da**)

Colorless oil (21.4 mg, 57%, *dr* = 4:1) from *N*-benzylskatole **1d** (20 mg, 66.62 μmol) and cyclopropane **2a** (21.1 mg, 79.95 μmol). **IR** (**KBr**, cm^−1^) **𝜈**: 2927, 2917, 2848, 1731, 1610, 1514, 1260. **^1^H NMR**: (700 MHz, CDCl_3_) δ 7.31–7.27 (m, 1.78H), 7.25–7.21 (m, 1.37H), 7.17–7.14 (m, 1.37H), 7.14–7.12 (m, 0.48H), 7.12–7.10 (m, 0.28H), 7.00 (dd, *J* = 8.4, 2.1 Hz, 0.70H), 6.96–6.92 (m, 1.13H), 6.90 (d, *J* = 8.6 Hz, 0.57H), 6.87–6.83 (m, 1.33H), 6.79 (d, *J* = 2.1 Hz, 0.28H), 6.76 (d, *J* = 8.7 Hz, 0.14H), 6.31 (d, *J* = 8.4 Hz, 0.21H), 6.26 (d, *J* = 8.5 Hz, 0.70H), 5.49 (d, *J* = 2.1 Hz, 0.74H), 4.68 (s, 0.20H), 4.62 (d, *J* = 1.5 Hz, 0.78H), 4.55 (d, *J* = 16.0 Hz, 0.77H), 4.32 (d, *J* = 16.1 Hz, 0.38H), 4.11 (d, *J* = 16.0 Hz, 0.76H), 3.84 (s, 0.72H), 3.83 (s, 1.93H), 3.81 (s, 0.71H), 3.77 (s, 1.93H), 3.52 (s, 1.87H), 3.47 (s, 0.55H), 2.95 (dd, *J* = 14.8, 4.7 Hz, 0.75H), 2.71 (dd, *J* = 14.8, 12.9 Hz, 0.79H), 2.65 (dd, *J* = 13.1, 5.8 Hz, 0.28H), 2.34–2.30 (m, 0.81H), 2.31–2.26 (m, 0.57H), 1.29 (s, 2.25H), 0.88 (s, 0.72H). **^13^C NMR**: (176 MHz, CDCl_3_) δ 172.63, 171.81, 170.26, 169.07, 159.17, 158.89, 152.21, 149.66, 139.62, 138.09, 137.96, 135.39, 130.55, 130.51, 130.38, 130.03, 130.00, 129.78, 129.49, 129.06, 128.93, 128.84, 128.64, 128.60, 128.51, 127.79, 127.63, 127.54, 127.33, 127.17, 126.84, 125.90, 125.81, 124.98, 114.15, 113.71, 113.50, 110.98, 110.69, 110.04, 109.95, 82.01, 80.00, 65.25, 64.21, 57.73, 56.13, 55.61, 55.42, 54.89, 53.33, 53.04, 53.02, 53.00, 52.73, 52.71, 52.67, 52.65, 52.55, 52.51, 41.29, 39.16, 36.69, 32.08, 29.85, 29.81, 29.51, 27.71, 23.32, 22.84, 14.27, 1.17. **HRMS** (ESI): Calculated for C_30_H_31_BrNO_5_ [M + H]^+^ = 564.1380; found: 564.1361. NMR data of the major isomer deduced from the 4:1 mixture: **^1^H NMR**: (700 MHz, CDCl_3_) δ 7.31–7.27 (m, 2H), 7.25–7.21 (m, 2H), 7.17–7.14 (m, 2H), 7.00 (dd, *J* = 8.4, 2.1 Hz, 1H), 6.96–6.92 (m, 1H), 6.87–6.83 (m, 2H), 6.26 (d, *J* = 8.5 Hz, 1H), 5.49 (d, *J* = 2.1 Hz, 1H), 4.62 (d, *J* = 1.5 Hz, 1H), 4.55 (d, *J* = 16.0 Hz, 1H), 4.11 (d, *J* = 16.0 Hz, 1H), 3.83 (s, 3H), 3.77 (s, 3H), 3.52 (s, 3H), 2.95 (dd, *J* = 14.8, 4.7 Hz, 1H), 2.71 (dd, *J* = 14.8, 12.9 Hz, 1H), 2.34–2.30 (m, 1H), 1.29 (s, 3H). **^13^C NMR**: (176 MHz, CDCl_3_) δ 171.81, 169.07, 159.17, 152.21, 138.09, 135.39, 130.51, 130.03, 130.00, 129.78, 129.06, 128.60, 128.51, 127.79, 127.63, 127.33, 113.71, 113.50, 110.98, 109.95, 80.00, 65.25, 57.73, 56.12, 55.61, 53.00, 52.55, 52.51, 36.69, 29.85, 27.71. The spectroscopic data agreed with the literature [48].

#### 3.2.9. Dimethyl (1S*,3aR*,8bS*)-4-Benzyl-7-chloro-1-(4-methoxyphenyl)-8b-methyl-1,3a,4,8b-tetrahydrocyclopenta[b]indole-3,3(2H)-dicarboxylate (**3ea**)

Colorless oil (23.6 mg, 58%, *dr* = 5:1) from *N*-benzylskatole **1e** (20 mg, 78.2 μmol) and cyclopropane **2a** (24.8 mg, 93.84 μmol). **IR** (**KBr**, cm^−1^) **𝜈**: 3024, 2950, 2834, 1731, 1599, 1514, 1246. **^1^H NMR**: (700 MHz, CDCl_3_) δ 7.32–7.24 (m, 4H), 7.24–7.16 (m, 3H), 7.04–7.01 (m, 0.29H), 6.92 (td, *J* = 7.7, 1.3 Hz, 1H), 6.88 (d, *J* = 8.6 Hz, 0.40H), 6.82–6.79 (m, 1.52H), 6.44 (d, *J* = 7.9 Hz, 0.19H), 6.40 (d, *J* = 7.9 Hz, 0.78H), 6.36 (td, *J* = 7.4, 1.0 Hz, 0.80H), 5.54 (dd, *J* = 7.5, 1.2 Hz, 0.81H), 4.70 (s, 0.21H), 4.65 (d, *J* = 16.3 Hz, 0.25H), 4.61 (d, *J* = 1.4 Hz, 0.65H), 4.59 (d, *J* = 16.1 Hz, 0.88H), 4.33 (d, *J* = 16.3 Hz, 0.25H), 4.16 (d, *J* = 16.1 Hz, 0.78H), 3.83 (s, 0.71H), 3.81 (s, 1.90H), 3.81 (s, 0.66H), 3.77 (s, 2.05H), 3.47 (s, 2.1H), 3.42 (s, 0.47H), 2.97 (dd, *J* = 14.8, 4.7 Hz, 0.80H), 2.75 (dd, *J* = 14.8, 12.8 Hz, 0.83H), 2.68 (dd, *J* = 13.1, 5.7 Hz, 0.24H), 2.31 (ddd, *J* = 12.6, 4.7, 1.5 Hz, 0.85H), 2.22 (t, *J* = 6.8 Hz, 0.14H), 1.34 (s, 2H), 0.93 (s, 0.60H). **^13^C NMR**: (176 MHz, CDCl_3_) δ 172.86, 172.00, 170.38, 169.22, 158.79, 158.74, 153.48, 150.91, 138.80, 138.64, 137.20, 133.02, 130.83, 130.45, 130.10, 129.19, 128.47, 128.38, 127.87, 127.85, 127.77, 127.60, 127.06, 126.90, 125.97, 125.45, 124.98, 122.84, 118.41, 118.22, 113.50, 113.26, 109.69, 109.57, 82.37, 80.22, 65.31, 64.12, 57.59, 56.70, 55.41, 55.40, 55.15, 53.79, 53.41, 52.95, 52.92, 52.57, 52.56, 52.47, 39.37, 36.87, 29.85, 28.16, 22.97, 21.61. **HRMS** (ESI): Calculated for C_30_H_31_ClNO_5_ [M + H]^+^ = 520.1885; found: 520.1854. NMR data of the major isomer deduced from the 5:1 mixture: **^1^H NMR**: (700 MHz, CDCl_3_) δ 7.32–7.24 (m, 3H), 7.22 (t, *J* = 7.3 Hz, 1H), 7.24–7.16 (m, 2H), 6.99–6.86 (m, 1H), 6.82–6.79 (m, 2H), 6.40 (d, *J* = 7.9 Hz, 1H), 6.36 (td, *J* = 7.4, 1.0 Hz, 1H), 5.54 (dd, *J* = 7.5, 1.2 Hz, 1H), 4.61 (d, *J* = 1.4 Hz, 1H), 4.59 (d, *J* = 16.1 Hz, 1H), 4.16 (d, *J* = 16.1 Hz, 1H), 3.81 (s, 3H), 3.77 (s, 3H), 3.47 (s, 3H), 2.97 (dd, *J* = 14.8, 4.7 Hz, 1H), 2.75 (dd, *J* = 14.8, 12.8 Hz, 1H), 2.31 (ddd, *J* = 12.6, 4.7, 1.5 Hz, 1H), 1.34 (s, 3H). **^13^C NMR**: (176 MHz, CDCl_3_) δ 171.99, 169.22, 158.79, 153.48, 138.80, 133.02, 130.45, 130.10, 129.19, 128.47, 128.38, 127.87, 127.77, 127.60, 127.06, 125.97, 118.22, 113.50, 113.26, 109.69, 80.22, 65.31, 57.59, 56.70, 55.41, 52.92, 52.56, 52.47, 36.87, 28.16. The spectroscopic data agreed with the literature [48].

#### 3.2.10. Dimethyl (1S*,3aR*,8bS*)-4-Benzyl-6-chloro-1-(4-methoxyphenyl)-8b-methyl-1,3a,4,8b-tetrahydrocyclopenta[b]indole-3,3(2H)-dicarboxylate (**3fa**)

Pale yellow oil (29.7 mg, 73%, *dr* = 8.6:1) from *N*-benzylskatole **1f** (20 mg, 78.2 μmol) and cyclopropane **2a** (24.8 mg, 93.84 μmol). **IR** (**KBr**, cm^−1^) **𝜈**: 3000, 2978, 2952, 2919, 2847, 1731, 1594, 1514, 1247. **^1^H NMR**: (700 MHz, CDCl_3_) δ 7.32–7.22 (m, 1.80H), 7.24 (t, *J* = 7.4 Hz, 1H), 7.18–7.15 (m, 2H), 7.12 (dd, *J* = 8.8, 2.6 Hz, 0.82H), 7.08–7.04 (m, 0.30H), 6.95 (d, *J* = 8.0 Hz, 1.34H), 6.89–6.87 (m, 0.28H), 6.83–6.78 (m, 1.87H), 6.62 (d, *J* = 1.3 Hz, 0.20H), 6.43 (s, 0.14H), 6.38 (d, *J* = 1.8 Hz, 0.82H), 6.31 (dd, *J* = 8.0, 1.9 Hz, 0.85H), 5.40 (d, *J* = 8.0 Hz, 0.82H), 4.69 (s, 0.14H), 4.64 (d, *J* = 1.4 Hz, 0.85H), 4.61 (d, *J* = 16.3 Hz, 0.21H), 4.56 (d, *J* = 16.1 Hz, 0.89H), 4.35 (d, *J* = 16.3 Hz, 0.20H), 4.13 (d, *J* = 16.1 Hz, 0.89H), 3.82 (s, 0.32H), 3.81 (s, 2.34H), 3.80 (s, 0.34H), 3.77 (s, 2.28H), 3.50 (s, 2.44H), 3.48 (s, 0.30H), 2.94 (dd, *J* = 14.8, 4.6 Hz, 0.88H), 2.72 (dd, *J* = 14.8, 12.8 Hz, 0.96H), 2.64 (dd, *J* = 13.1, 5.8 Hz, 0.20H), 2.31 (ddd, *J* = 12.8, 4.7, 1.5 Hz, 0.92H), 1.29 (s, 2.49H), 0.87 (s, 0.52H). **^13^C NMR**: (176 MHz, CDCl_3_) δ 172.62, 171.80, 170.49, 170.24, 169.06, 167.32, 167.08, 159.06, 158.90, 154.43, 137.90, 137.77, 136.02, 133.74, 131.61, 130.55, 130.04, 130.02, 129.76, 129.49, 129.19, 129.04, 128.84, 128.64, 128.56, 128.38, 128.04, 127.79, 127.58, 127.54, 127.36, 127.22, 126.66, 126.61, 125.45, 124.98, 123.51, 118.12, 118.09, 114.15, 113.74, 113.62, 113.37, 109.57, 109.28, 82.02, 80.10, 65.12, 64.19, 57.65, 57.15, 55.70, 55.42, 55.41, 55.36, 54.53, 53.43, 53.02, 53.00, 52.90, 52.71, 52.66, 52.56, 52.40, 52.32, 50.12, 41.29, 39.17, 37.23, 36.82, 32.37, 29.85, 29.81, 27.92, 23.32, 19.43, 1.17. **HRMS** (ESI): Calculated for C_30_H_31_ClNO_5_ [M + H]^+^ = 520.1885; found: 520.1854. NMR data of the major isomer deduced from the 8.6:1 mixture: **^1^H NMR**: (700 MHz, CDCl_3_) δ 7.32–7.22 (m, 2H), 7.18–7.15 (m, 1H), 7.12 (dd, *J* = 8.8, 2.6 Hz, 2H), 6.95 (d, *J* = 8.0 Hz, 1H), 6.83–6.78 (m, 1H), 6.38 (d, *J* = 1.8 Hz, 2H), 6.31 (dd, *J* = 8.0, 1.9 Hz, 1H), 5.40 (d, *J* = 8.0 Hz, 1H), 4.64 (d, *J* = 1.4 Hz, 1H), 4.56 (d, *J* = 16.1 Hz, 1H), 4.13 (d, *J* = 16.1 Hz, 1H), 3.81 (s, 3H), 3.77 (s, 3H), 3.50 (s, 3H), 2.94 (dd, *J* = 14.8, 4.6 Hz, 1H), 2.72 (dd, *J* = 14.8, 12.8 Hz, 1H), 2.31 (ddd, *J* = 12.8, 4.7, 1.5 Hz, 1H), 1.29 (s, 3H). **^13^C NMR**: (176 MHz, CDCl_3_) δ 171.80, 169.06, 158.90, 154.43, 137.90, 133.74, 131.61, 130.04, 130.02, 129.76, 128.64, 127.58, 127.36, 126.66, 118.09, 113.74, 113.37, 109.57, 80.10, 65.12, 57.15, 55.70, 55.42, 55.41, 55.36, 53.00, 52.90, 52.71, 52.56, 52.40, 52.32, 37.23, 36.82, 32.37, 29.85, 27.92, 19.43.

## 4. Conclusions

In conclusion, the nickel-catalyzed regio- and diastereoselective formal [3+2] cycloaddition of *N*-benzylskatoles and donor–acceptor cyclopropanes was developed. The reaction tolerated different monosubstitutions in a series of indoles and cyclopropanes, providing yields of up to 93%, with *dr* 8.6:1 and complete regioselectivity. Of the synthesized compounds, we were able to determine six new cyclopenta[*b*]indoles and obtain five previously reported derivatives. Based on the results obtained and the bibliographic precedents, a reaction mechanism was proposed and studied via online reaction monitoring by ESI-MS. All the proposed intermediates were detected experimentally, allowing us to establish the active species of the catalytic cycle and the important role of rac-BINAP in the proposed mechanism. This method enables the rapid construction of the medicinally relevant cyclopenta[*b*]indole scaffold using an inexpensive nickel catalyst.

## Figures and Tables

**Table 1:** Screening of the metal salts ^1^.

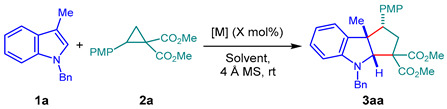						
Entry	[M] (X mol%)	Solvent	t (h)	Conv. (%) ^2^	Yield (%) ^3^	*dr* ^4^
1	Cu(OTf)_2_ (20)	DCE	24	>99	79	5:1
2	Co(ClO_4_)_2_·6H_2_O (20)	DCE	24	-	42	6:1
3 ^5^	Zn(ClO_4_)_2_·6H_2_O (20)	DCE	48	63	28	6.4:1
**4**	**Ni(ClO_4_)_2_·6H_2_O (20)**	**DCE**	**24**	**>99**	**95**	**6:1**
5	Ni(ClO_4_)_2_·6H_2_O (10)	DCE	48	50	48	6:1
6	Ni(OAc)_2_·4H_2_O (10)	DCE	24	15	9.3	5:1
7	NiSO_4_·6H_2_O (10)	DCE	24	86	56	4:1
8	Ni(NO_3_)_2_·6H_2_O (10)	DCE	24	91	48	9:1
9	NiCl_2_ (10)	DCE	24	57	55	3.6:1
**10**	**Ni(OTf)_2_ (10)**	**DCE**	**24**	**>99**	**78**	**5:1**
11	Ni(OTf)_2_ (10)	DCM	24	73	47	7.7:1
12	Ni(OTf)_2_ (10)	CHCl_3_	24	85	25	1:1
13	Ni(OTf)_2_ (10)	Toluene	24	76	29	1:1
14	Ni(OTf)_2_ (10)	CH_3_CN	48	<5	-	-
15	Ni(OTf)_2_ (10)	AcOEt	48	<5	-	-

^1^ Reaction conditions: Indole **1a** (1.0 eq.) and cyclopropane **2a** (1.2 eq.), was treated with [M] (X mol%) in the indicated solvent (0.1 M) at rt and time (h). ^2^ Based on recovery of starting material. ^3^ Isolated yield. ^4^ Calculated from the ^1^H NMR. ^5^ 24 h rt, and then 24 h at 50 °C.

**Table 2:** Screening of the ligand influence ^1^.

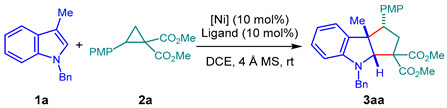					
Entry	[Ni]/Ligand	t (h)	Conv. (%) ^2^	Yield (%) ^3^	*dr* ^4^
1 ^5^	Ni(OTf)_2_/Bipy	144	<5	-	-
2 ^5^	Ni(OTf)_2_/Phen	72	26	12	1:1
3	Ni(OTf)_2_/dppe	144	7.5	-	-
4	Ni(OTf)_2_/d^F^ppe	144	24	13	4:1
5 ^5^	Ni(OTf)_2_/L_2_-PrPr_2_	72	<5	-	-
6 ^5^	Ni(OTf)_2_/L_3_-PrPr_2_	72	<5	-	-
7	Ni(ClO_4_)_2_·6H_2_O/Phen	96	63	16	4.4:1
8	Ni(ClO_4_)_2_·6H_2_O/dppe	96	-	19	5.7:1
9	Ni(ClO_4_)_2_·6H_2_O/d^F^ppe	168	-	43	5.5:1
10	Ni(ClO_4_)_2_·6H_2_O/dppBz	120	70	53	5.5:1
11 ^5^	Ni(ClO_4_)_2_·6H_2_O/dppf	144	<5	-	-
**12**	**Ni(ClO_4_)_2_·6H_2_O/rac-BINAP**	**5**	**>99**	**73**	**6:1**
13 ^6^	Ni(ClO_4_)_2_·6H_2_O/rac-BINAP	5	>99	52	3:1
14 ^5^	Ni(ClO_4_)_2_·6H_2_O/rac-BINAP	5	>99	28	4:1

^1^ Reaction conditions: Indole **1** (1.0 eq.) and cyclopropane **2a** (1.2 eq.), was treated with [Ni] (10 mol%) and ligand (10 mol%) in DCE (0.1 M) at rt and time (h). ^2^ Based on recovery of starting material. ^3^ Isolated yield. ^4^ Calculated from the ^1^H NMR. ^5^ Carried out at 85 °C. ^6^ Carried out at 50 °C.

## Data Availability

Data are contained within the article and Supplementary Materials.

## Supplementary Materials

The following supporting information can be downloaded at https://www.mdpi.com/article/10.3390/molecules29071604/s1: Synthesis procedures and characterization of indoles **1a-m** and donor–acceptor cyclopropanes **2a-I**; Tables S1–S5: Copies of IR, ^1^H, ^13^C-NMR, 2D-NMR, and HRMS-ESI spectra. References [88,89,90,91,92,93,94,95,96,97,98,99,100] are cited in the supplementary materials.
