# Supplementary material for: Nickel(II)-Catalyzed Formal [3+2] Cycloadditions between Indoles and Donor–Acceptor Cyclopropanes
Source: Molecules. 2024 Apr 3;29(7):1604. doi: 10.3390/molecules29071604 (PMC11013886; doi:10.3390/molecules29071604)
Supplement: Supplementary file 1 [file molecules-29-01604-s001.zip › molecules-2942692-supplementary.pdf]

SUPPLEMENTARY MATERIALS

Nickel(II)-Catalyzed Formal [3+2] Cycloadditions Between Indoles and Donor-Acceptor Cyclopropanes

Víctor Quezada [1], Mariña Castroagudín [1], Felipe Verdugo [2], Sergio Ortiz [3], Guillermo Zaragoza [4], Fabiane M. Nachtigall [5], Francisco A. A. Reis [6], Alejandro Castro-Alvarez [7,]*, Leonardo S. Santos [6,]*, Ronald Nelson [1,]*

1 Departamento de Química, Facultad de Ciencias, Universidad Católica del Norte, Avda. Angamos 0610, Antofagasta 1270709, Chile.rnelson@ucn.cl
2 Departamento de Química Orgánica, Facultad de Ciencias Químicas, Universidad de Concepción, Edmundo Larenas 129, Concepción 4070371, Chile.
3 UMR 7200 Laboratoire d'Innovation Thérapeutique, CNRS, Strasbourg Drug Discovery and Development Institute (IMS), Université de Strasbourg, 67400 Illkirch-Graffenstaden, France.
4 Unidade de Difracción de Raios X, RIAIDT, Universidade de Santiago de Compostela, Campus VIDA, Santiago de Compostela 15782, Spain.
5 Instituto de Ciencias Aplicadas, Universidad Autónoma de Chile, Talca 3467987, Chile.
6 Laboratory of Asymmetric Synthesis, Chemistry Institute of Natural Resources, Universidad de Talca, Talca 3460000, Chile; lssantos@utalca.cl
7 Departamento de Ciencias Preclínicas, Facultad de Medicina, Universidad de La Frontera, Temuco 4811230, Chile; alejandro.castro.a@ufrontera.cl

General Contents

1. General Procedures **2**

1-Synthesis of indole derivative **3**

Synthesis of substituted skatoles 1b to 1f (exemplified for the synthesis of 1-benzyl-5-chloro-3-methyl-1*H*-indole (1e)). **4**

Synthesis of (1-benzyl-5-nitro-1*H*-indol-3-yl)methyl 4-nitrobenzoate (1g) **7**

Synthesis of 3-methyl-1-tosyl-1*H*-indole (1k). **8**

Synthesis of *tert*-butyl 3-methyl-1*H*-indole-1-carboxylate (1l). **9**

Synthesis of 3-methyl-1-benzoyl-1*H*-indole (1m). **9**

2. Synthesis of donor-acceptor cyclopropanes (exemplified for the synthesis of dimethyl 2-(4-methoxyphenyl)cyclopropane-1,1-dicarboxylate (2a). **11**

Synthesis of dimethyl 2-vinylcyclopropane-1,1-dicarboxylate (2h). **14**

3. Optimization studies. **15**

4. General procedure for [3+2] cycloaddition (exemplified for the synthesis of dimethyl (1S*,3aR*,8bS*)-4-benzyl-1-(4-methoxyphenyl)-8b-methyl-1,3a,4,8b-tetrahydrocyclopenta[b]indole-3,3(2H)-dicarboxylate (3aa)): **20**

5. ^{1}H NMR, ^{13}C NMR, FT-IR and Mass spectra. **33**

6. Crystal data and structure refinement for 3ac. **104**

1. General Procedures

All the reactions were conducted in dry solvents under N_2 atmosphere unless otherwise stated. All reagents used were obtained from commercial suppliers and used without further purification. The abbreviation "rt" refers to reactions carried out approximately at 25 °C. Reaction mixtures were stirred using Teflon-coated magnetic stirring bars. Reaction temperatures were maintained using Thermowatch-controlled silicone oil baths. The reactions were monitored by Thin-layer chromatography (TLC) was performed on silica gel Merck 60 F_{254} and components were visualized by observation under UV light (254 and 365 nm), and/or by treating the plates with *p*-anisaldehyde, oleum, phosphomolybdic acid or cerium nitrate solutions, followed by heating. Flash chromatography was carried out on silica gel (63-200 µm) unless otherwise stated. Dryings were performed with anhydrous Na_2SO_4. Concentration refers to the removal of volatile solvents via distillation using a Büchi rotary evaporator R-300 followed by residual solvent removal under high vacuum. Melting points were determined using a Stuart SMP3 apparatus and were uncorrected. Infrared spectra were measured using a Perkin-Elmer FT-IR Spectrometer Spectrum Two with KBr pellets. NMR spectra were recorded in $CDCl_3$, at 300, 400, 500 or 700 MHz (Bruker Advance III). Chemical shifts were reported in parts per million (δ) using the residual solvent signals ($CDCl_3$: δ_H 7.26, δ_C 77.16) as the internal standards for the ^1H and ^{13}C NMR spectra and coupling constants (*J*) in Hz. Carbon types and structure assignments were determined from DEPT-NMR and two-dimensional experiments (HSQC and HMBC, COSY and NOESY). The following abbreviations are used to indicate signal multiplicity: s, singlet; d, doublet; t, triplet; q, quartet; p, pentet; dd, double doublet; td, triple doublet; ddd, doublet of doublet of doublets; m, multiplet; br, broad. Mass spectra (ESI-MS) were acquired using an Agilent 1200 ESI/APCI QTof tandem Agilent Mass QTof 6520. For the crystal structure determination, data were collected by applying the omega and phi scans method on a Bruker D8 VENTURE PHOTON III-14 diffractometer using Incoatec multilayer mirror monochromated with Mo-Kα radiation (λ = 0.71073Å) from a microfocus sealed tube source at 100 K with detector resolution of 7.3910 pixels mm^{-1}. Enantioselectivities were determined in an Agilent HPLC-DAD 1260 Infinity with Chiralpak IF3 analytical columns using Hexane:*i*PrOH (99:1), 0.5 mL/min.

1-Synthesis of indole derivative

Synthesis of -benzyl-3-methyl-1*H*-indole (1a).[1]

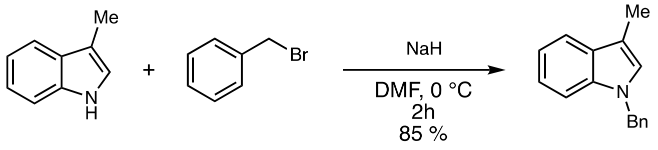

1a

NaH (60% dispersion in mineral oil, 1.10 g, 27.44 mmol, 1.2 eq) was added portion wise over 5 min to a solution of 3-methyl-1*H*-indole (3.00 g, 22.87 mmol, 1.00 eq) in DMF (33 mL) at 0 °C, and the mixture was stirred for 30 min at room temperature. The reaction mixture was then cooled to 0 °C and benzyl bromide (3.26 mL, 27.44 mmol, 1.2 eq) was added dropwise. The resulting white precipitate was further allowed to stir at room temperature for 2 h. After completion of the reaction (as indicated by TLC), the reaction mixture was quenched with saturated ammonium chloride solution (100 mL) and the aqueous phase was extracted with diethyl ether (3 x 50 mL). The combined organic phases were dried over anhydrous Na_2SO_4, filtered and the solvent was removed under reduced pressure to afford indole **1a** as a white solid (4.3 g, 85%). **IR (KBr, cm^{-1}) *ν*:** 3029, 2912, 2853, 1583, 1452, 1330. **^{1}H-NMR:** (300 MHz, $CDCl_3$) δ 7.73 – 7.66 (m, 1H), 7.38 – 7.31 (m, 4H), 7.28 (dd, *J* = 6.9, 1.4 Hz, 1H), 7.20 (m, 3H), 6.96 (d, *J* = 1.4 Hz, 1H), 5.31 (s, 2H), 2.44 (s, 3H).**^{13}C-NMR:** (75 MHz, $CDCl_3$) δ 138.03, 128.81, 127.59, 126.93, 125.94, 121.74, 119.15, 118.92, 110.97, 109.57, 49.87, 9.75. The spectroscopic data agree with the literature.[1]

A similar procedure was followed as applied for the synthesis of **1h**, **1i** and **1j**.

1-benzyl-2,3-dimethyl-1*H*-indole (1h).

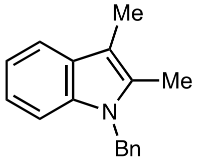

Orange solid (70%) **mp:** 62-67 °C. **IR (KBr, cm^{-1}) *ν*:** 3056, 3023, 2920, 2856, 1605, 1470, 737. **^{1}H-NMR:** (400 MHz, $CDCl_3$) δ 7.62 – 7.52 (m, 1H), 7.31 – 7.19 (m, 4H), 7.20 – 7.07 (m, 2H), 7.03 – 6.95 (m, 2H), 5.31 (s, 2H), 2.32 (s, 3H), 2.31 (s, 3H). **^{13}C-NMR:** (101 MHz, $CDCl_3$) δ 138.41, 136.52, 132.54, 128.82, 128.78, 127.27, 126.13, 120.89, 118.96, 118.12, 108.92, 107.13,

[1] T. R. Pradhan, H. W. Kim, J. K. Park, *Org. Lett.* **2018**, *20*, 5286–5290

46.62, 10.28, 9.01. **HRMS-ESI:** Calculated for $C_{17}H_{17}N$ $[M+H]^+$ = 235.1356, Found: 235.1349. The spectroscopic data agree with the literature.[2]

9-benzyl-2,3,4,9-tetrahydro-1*H*-carbazole (1i).

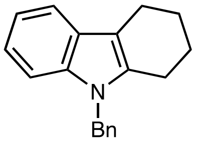

White solid (50%) **IR (cm^{-1}) ν:** 3028, 2917, 2839, 1603, 1467, 741. **^{1}H-NMR:** (400 MHz, $CDCl_3$) δ 7.68 (ddd, *J* = 6.7, 4.1, 2.6 Hz, 1H), 7.45 – 7.34 (m, 4H), 7.31 – 7.22 (m, 2H), 7.20 – 7.13 (m, 2H), 5.40 (s, 2H), 2.94 (tt, *J* = 5.8, 1.7 Hz, 2H), 2.80 (tt, *J* = 6.1, 1.7 Hz, 2H), 2.06 (dtddd, *J* = 16.1, 8.4, 5.9, 4.2, 1.9 Hz, 4H). **^{13}C-NMR:** (101 MHz, $CDCl_3$) δ 138.43, 136.70, 135.69, 128.80, 127.60, 127.27, 126.29, 120.88, 118.98, 117.90, 110.00, 109.08, 46.32, 23.38, 23.33, 22.27, 21.24. **HRMS-ESI:** Calculated for $C_{19}H_{20}N$ $[M+H]^+$ = 262.1587, Found: 262.15903. The spectroscopic data agree with the literature.[3]

Methyl 1-benzyl-1*H*-indole-3-carboxylate (1j).

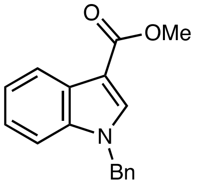

Pale yellow solid (96%) **mp:** 101-103 °C. **IR (cm^{-1}) ν:** 3116, 3056, 2950, 2858, 1697, 1536. **^{1}H-NMR:** (400 MHz, $CDCl_3$) δ 8.17 (dt, *J* = 7.8, 1.0 Hz, 1H), 7.80 (s, 1H), 7.31 – 7.17 (m, 6H), 7.14 – 7.07 (m, 2H), 5.27 (s, 2H), 3.87 (s, 3H). **^{13}C-NMR:** (101 MHz, $CDCl_3$) δ 165.55, 136.87, 136.01, 134.67, 129.10, 128.25, 127.19, 126.92, 123.07, 122.15, 121.89, 110.42, 107.66, 51.13, 50.81. **HRMS-ESI:** Calculated for $C_{17}H_{15}NO_2Na$ $[M+Na]^+$ = 288.0995, Found: 288.0988. The spectroscopic data agree with the literature.[4]

Synthesis of substituted skatoles 1b to 1f (exemplified for the synthesis of 1-benzyl-5-chloro-3-methyl-1*H*-indole (1e)).

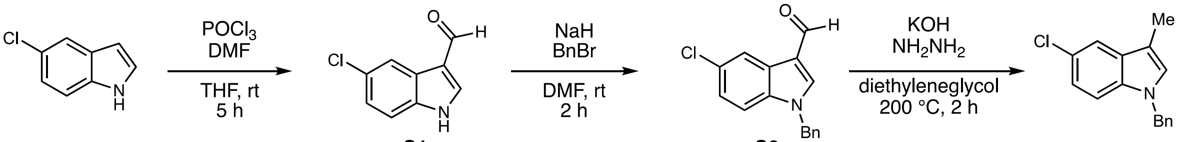

S1 S2 1e

$POCl_3$ (463 mL, 4.95 mmol, 1.50 eq) were added to 3.1 mL of DMF (39.58 mmol, 1.00 eq) at 0 °C and stirred for 45 minutes. After this time, a solution of 5-chloroindole (500

---

[2] A. Saito, A. Kanno, Y. Hanzawa, *Angew. Chem. Int. Ed.* **2007**, *46*, 3931–3933.
[3] J. Bergès, B. García, K. Muñiz, *Angew. Chem. Int. Ed.* **2018**, *57*, 15891–15895.
[4] T. Varlet, D. Bouchet, E. Van Elslande, G. Masson, *Chem. Eur. J.* **2022**, *28*, 3–8.

mg, 3.30 mmol, 1.00 eq) dissolved in 19.5 mL of THF was added and a precipitate was formed. The reaction was brought to room temperature and stirred for 4 hours. Finally, water was added to dissolve the precipitate, and the mixture was stirred overnight. It was diluted with EtOAc and washed with water (3 x10 mL), 1% $NaHCO_3$ solution, and brine solution. Crude residue was purified by column chromatographic (50% EtOAc/hexane) to give product **S1** as a pale yellow solid (392.2 mg, 66%). **mp:** 216-218 ºC; **IR (cm^{-1}) *ν*:** 3211, 3104, 3044, 1642. **^{1}H-NMR:** (400 MHz, $CDCl_3$) δ 10.05 (s, 1H), 8.70 (s, 1H), 8.34 (d, *J* = 2.0 Hz, 1H), 7.86 (d, *J* = 3.1 Hz, 1H), 7.37 (d, *J* = 8.6 Hz, 1H), 7.30 (dd, *J* = 8.7, 2.0 Hz, 1H). **HRMS-ESI:** Calculated for C_9H_7ClNO $[M+H]^+$ = 180.0211, Found: 180.0208.

Next, a protection step was carried out through an *N*-benzylation, as described in the literature:[1]. To a solution of 3-formylindole **S1** (300 mg, 1.67 mmol, 1.00 eq) in 2.4 mL of DMF at 0 °C, NaH (60% in mineral oil, 80.17 mg, 2 mmol, 1.20 eq) was carefully added, and the mixture was stirred for 35 minutes at 0 °C. After this time, benzyl bromide (328 mL, 2 mmol, 1.20 eq) was added, resulting in the formation of a pale yellow precipitate. The reaction was allowed to continue stirring for 2 hours until completion (as indicated by TLC), and it was quenched by adding a saturated solution of NH_4Cl. The aqueous phase was extracted with diethyl ether (3x 20 mL) and the combined organic phases were dried over anhydrous Na_2SO_4, filtered and concentrated. The crude product was purified by column chromatography (50% EtOAc/hexane) yielding product **S2** as a yellow solid (289 mg, 64%). **mp:** 144-149 ºC: **IR (cm^{-1}) *ν*:** 3102, 3025, 2819, 1654. **^{1}H-NMR:** (400 MHz, $CDCl_3$) δ 9.98 (s, 1H), 8.36 (dd, *J* = 1.8, 0.9 Hz, 1H), 7.74 (s, 1H), 7.44 – 7.36 (m, 3H), 7.31 – 7.24 (m, 2H), 7.23 – 7.17 (m, 2H), 5.37 (s, 2H). **^{13}C-NMR:** (101 MHz, $CDCl_3$) δ 184.44, 184.41, 139.26, 135.89, 135.01, 129.32, 129.19, 128.69, 127.29, 126.56, 124.67, 121.88, 118.03, 111.57, 51.29. **HRMS-ESI:** Calculated for $C_{16}H_{12}ClNa$ $[M+Na]^+$ = 292.0493, Found: 292.0499.

Finally, product **S2** was reduced through a Wolff/Kishner reaction by sequentially adding KOH (374 mg, 6.67 mmol, 9.00 eq) and hydrazine monohydrate (540 mL, 11.12 mmol, 15.0 eq) to a previously prepared solution of product **S2** (200 mg, 741 mmol, 1.00 equiv.) in diethylene glycol (7.4 mL) under a nitrogen atmosphere at room temperature. The mixture was then stirred for 2 hours at 200 °C and monitored by chromatography (TLC). Once the reaction was complete, it was cooled to room temperature, diluted with Et_2O, and

washed with saturated solution of NH_4Cl, water, and brine. The organic phase was dried over anhydrous Na_2SO_4, filtered and concentrated. The crude product was purified by column chromatography (10% EtOAc/hexane) to obtain product **1e** (147 mg, 78%) as a white solid. **mp:** 60-61 ºC; **IR (cm^{-1}) *ν*:** 3060, 3025, 2916, 2854, 1604, 1577, 1450. **^{1}H-NMR:** (400 MHz, $CDCl_3$) δ 7.53 (d, *J* = 1.9 Hz, 1H), 7.27 (dddd, *J* = 11.1, 8.9, 5.8, 2.4 Hz, 3H), 7.15 – 7.03 (m, 4H), 6.89 (d, *J* = 1.2 Hz, 1H), 5.21 (s, 2H), 2.28 (d, *J* = 1.0 Hz, 3H). **^{13}C-NMR:** (101 MHz, $CDCl_3$) δ 137.57, 135.14, 130.13, 128.92, 127.81, 127.36, 126.84, 124.80, 121.99, 118.73, 110.72, 110.66, 50.13. **HRMS-ESI:** Calculated for $C_{16}H_{15}ClN$ $[M+H]^+$ = 256.0888, Found: 256.0890. The spectroscopic data agree with the literature.[5]

1-benzyl-3,7-dimethyl-1*H*-indole (1b).

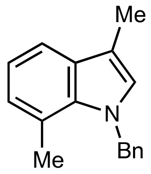

Brown solid (55%) **mp:** 48-50 °C. **IR (cm^{-1}) *ν*:** 3033, 2969, 2927, 2853, 1603, 1450, 742. **^{1}H-NMR:** (400 MHz, $CDCl_3$) δ 7.45 (d, *J* = 7.9 Hz, 1H), 7.30 – 7.17 (m, 3H), 7.06 – 6.96 (m, 1H), 6.95 – 6.85 (m, 3H), 6.82 (d, *J* = 1.2 Hz, 1H), 5.50 (s, 2H), 2.51 (s, 3H), 2.33 (s, 3H).**^{13}C-NMR:** (101 MHz, $CDCl_3$) δ 140.12, 135.46, 130.05, 128.88, 127.89, 127.30, 125.61, 124.64, 121.01, 119.24, 117.19, 110.99, 51.96, 19.62, 9.76. **HRMS-ESI:** Calculated for $C_{17}H_{17}N$ $[M+H]^+$ = 235.1356, Found: 235.1349. The spectroscopic data agree with the literature.[5]

1-benzyl-5-methoxy-3-methyl-1*H*-indole (1c).

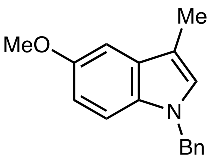

White solid (75%) **mp:** 75-77 °C. **IR (cm^{-1}) *ν*:** 3029, 2915, 1581, 1493, 1231, 1041. **^{1}H-NMR:** (400 MHz, $CDCl_3$) δ 7.47 – 7.36 (m, 3H), 7.29 – 7.22 (m, 3H), 7.18 (d, *J* = 2.5 Hz, 1H), 7.03 (d, *J* = 1.1 Hz, 1H), 6.98 (dd, *J* = 8.8, 2.5 Hz, 1H), 5.37 (s, 2H), 4.02 (s, 3H), 2.46 (s, 3H). **^{13}C-NMR:** (101 MHz, $CDCl_3$) δ 153.87, 138.12, 132.09, 129.30, 128.82, 127.60, 126.86, 126.70, 111.91, 110.41, 101.02, 50.11, 9.83. **HRMS-ESI:** Calculated for $C_{17}H_{17}NO$ $[M+H]^+$ = 251.1305, Found: 251.1293. The spectroscopic data agree with the literature.[6]

1-benzyl-5-bromo-3-methyl-1*H*-indole (1d).

[5] H. Xiong, H. Xu, S. Liao, Z. Xie, Y. Tang, *J. Am. Chem. Soc.* **2013**, *135*, 7851–7854.
[6] T. Mandal, G. Chakraborti, J. Dash, *Tetrahedron Lett.* **2020**, *61*, 152109.

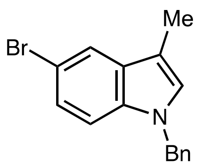

Pale brown solid (70%) **mp:** 55-57 °C. **IR (cm^{-1}) ν:** 3060, 3023, 2922, 2857, 1578, 1471, 1451, 1356, 1287, 790. **^{1}H-NMR:** (400 MHz, $CDCl_3$) δ 7.88 (d, *J* = 1.9 Hz, 1H), 7.46 (dddd, *J* = 9.6, 7.7, 5.1, 2.0 Hz, 3H), 7.41 (dd, *J* = 8.6, 1.9 Hz, 1H), 7.30 – 7.22 (m, 3H), 7.07 (d, *J* = 1.1 Hz, 1H), 5.40 (s, 2H), 2.47 (s, 3H). **^{13}C-NMR**: (101 MHz, $CDCl_3$) δ 137.52, 135.40, 130.79, 128.93, 127.82, 127.21, 126.83, 124.53, 121.85, 112.32, 111.12, 110.68, 50.12, 9.63. **HRMS-ESI:** Calculated for $C_{16}H_{15}NBr$ $[M+H]^+$ = 300.03824, Found: 300.0385. The spectroscopic data agree with the literature.[5]

1-benzyl-6-chloro-3-methyl-1*H*-indole (1f).

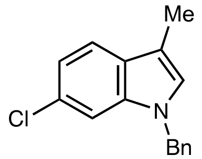

Yellow solid (82%) **mp:** 77-79 °C. **IR (cm^{-1}) ν:** 3083, 3060, 3028, 2918, 2858, 1605, 1469, 1327, 813. **^{1}H-NMR:** (400 MHz, $CDCl_3$) δ 7.65 (d, *J* = 8.4 Hz, 1H), 7.51 – 7.39 (m, 4H), 7.26 (ddd, *J* = 10.2, 7.9, 1.9 Hz, 3H), 7.05 (q, *J* = 1.1 Hz, 1H), 5.38 (s, 2H), 2.48 (s, 3H). **^{13}C-NMR**: (101 MHz, $CDCl_3$) δ 137.45, 137.14, 128.95, 127.88, 127.83, 127.65, 126.87, 126.63, 120.09, 119.63, 111.29, 109.56, 49.97, 9.67. **HRMS-ESI:** Calculated for $C_{16}H_{15}NCl$ $[M+H]^+$ = 259.09, Found: 256.09. The spectroscopic data agree with the literature.[7]

Synthesis of (1-benzyl-5-nitro-1*H*-indol-3-yl)methyl 4-nitrobenzoate (1g).

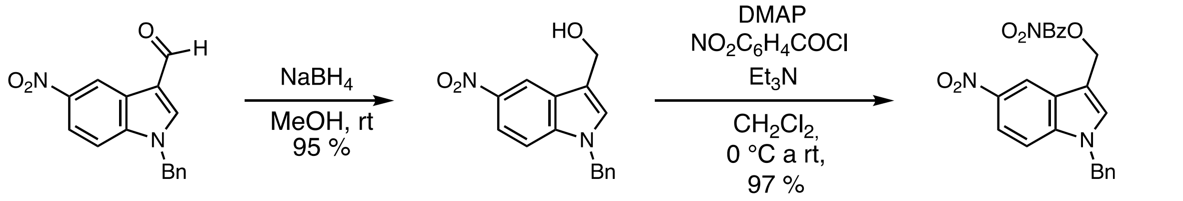

S3 **S4** **1g**

To a solution of aldehyde **S3** (100 mg, 0.357 mmol, 1.00 eq) in methanol (446 mL), $NaBH_4$ (20.25 mg, 0.535 mmol, 1.5 eq) was added, and the mixture was stirred at room temperature. After 1 hour of reaction, the starting material was completely consumed (monitored by TLC). Subsequently, 2 mL of cold water was added, and the yellow precipitate was filtered and dried under vacuum, to afford alcohol **S4** (96.2 mg, 95% yield). **^{1}H-NMR:** (700 MHz, $CDCl_3$) δ 8.72 (d, *J* = 2.3 Hz, 1H), 8.12 (dd, *J* = 9.1, 2.3 Hz, 1H), 7.42 – 7.25 (m,

[7] L. Yu, Y. Zhong, J. Yu, L. Gan, Z. Cai, R. Wang, X. Jiang, *Chem. Commun.* **2018**, *54*, 2353–2356.

5H), 7.16 (dd, *J* = 8.1, 1.5 Hz, 2H), 5.36 (s, 2H), 4.94 (d, 2H). **^{13}C-NMR:** (176 MHz, $CDCl_3$) δ 141.84, 139.77, 136.10, 129.92, 129.19, 128.39, 127.05, 126.74, 118.17, 118.02, 116.99, 109.93, 56.89, 50.70.

Finally, a solution of alcohol **S4** (50.0 mg, 0.177 mmol, 1.00 eq) in DCM (3.54 mL) was treated with 4-nitrobenzoyl chloride (39.44 mg, 0.212 mmol, 1.2 eq) and Et_3N (29.62 μL, 0.212 mmol 1.2 eq). The mixture was cooled in an ice bath, and then DMAP (2.16 mg, 0.0177 mmol, 0.10 eq) was added. After 10 minutes, the ice bath was removed, and the reaction was allowed to proceed at room temperature until completion (monitored by TLC). After 8 hours of reaction, water (10 mL) was added, and the mixture was extracted with DCM (3x 20 mL). The organic phase was washed with brine, dried over anhydrousNa_2SO_4, filtered and concentrated under vacuum. Crude product was purified by column chromatography (50% EtOAc/hexane) to give product **1g** as a white solid (74.1 mg, 97%) **mp:** 164-166 °C. **IR (KBr, cm^{-1})** ν**:** 3088, 2943, 1721, 1530, 1330, 1271.

Synthesis of 3-methyl-1-tosyl-1*H*-indole (1k).

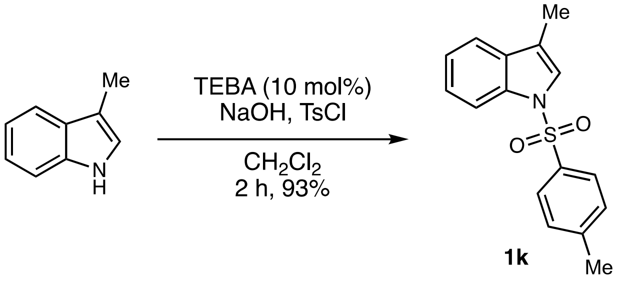

1k

p-toluenesulfonyl chloride (3.20 g, 16.77 mmol, 1.10 eq), 3-methyl-1*H*-indole (2.00 g, 15.25 mmol, 1.00 eq), NaOH (1.07 g, 26.68 mmol, 1.75 eq), and TEBA (347.28 mg, 0.381 mmol, 0.10 eq) were mixed in DCM (60 mL) in a 100 mL round-bottom flask and allowed to react at room temperature for 2 hours (monitored by TLC). Once the reaction was complete, the mixture was filtered and concentrated under vacuum, and then purified by column chromatography, resulting in the isolation of indole derivative **1k** (93%). **IR (KBr, cm^{-1})**ν **:** 3110, 3054, 2918, 2856, 1914, 1594, 1444, 1362, 1170. **^{1}H-NMR:** (300 MHz, $CDCl_3$) δ 8.00 (dt, *J* = 8.2, 1.0 Hz, 1H), 7.78 – 7.73 (m, 2H), 7.46 (ddd, *J* = 7.7, 1.5, 0.8 Hz, 1H), 7.37 – 7.29 (m, 2H), 7.26 (dd, *J* = 7.5, 1.2 Hz, 1H), 7.23 – 7.17 (m, 2H), 2.33 (s, 3H), 2.25 (s, 3H). **^{13}C-NMR:** (75 MHz, $CDCl_3$) δ 144.74, 135.63, 135.43, 131.93, 129.88, 126.86, 124.68,

123.20, 123.10, 119.50, 118.69, 113.81, 21.63, 9.77. The spectroscopic data agree with the literature.[8]

Synthesis of *tert*-butyl 3-methyl-1*H*-indole-1-carboxylate (1l).

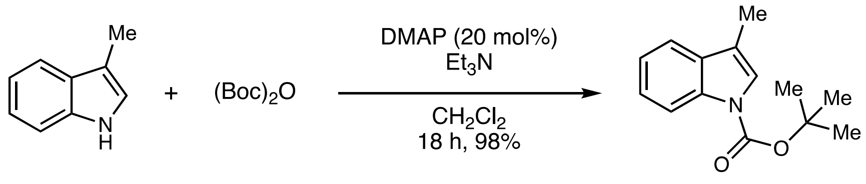

1l

3-methyl-1*H*-indole (1.00 g, 7.22 mmol, 1.00 eq), Et_3N (2.31 g, 22.87 mmol, 3.00 eq), and DMAP (186.3 mg, 1.52 mmol, 0.20 eq) were dissolved in 20.0 mL of dichloromethane, and subsequently, $(Boc)_2O$ (1.83 g, 8.39 mmol, 1.10 eq) was dissolved in dichloromethane and added dropwise to the mixture. After 18 hours of reaction, water was added, and the mixture was extracted with DCM (3x 30 mL). The combined organic phase was dried over anhydrous Na_2SO_4 and concentrated. Finally, it was purified by column chromatography (20% EtOAc/hexane) to obtain product **1l** (98%). **IR (KBr, cm^{-1}) ν:** 2976, 2924, 1730, 1454, 1370, 1252, 1160, 744. **^{1}H-NMR:** (400 MHz, $CDCl_3$) δ 7.51 (dt, *J* = 7.7, 1.1 Hz, 1H), 7.38 (s, 1H), 7.34 (ddd, *J* = 8.4, 7.2, 1.4 Hz, 1H), 7.26 (td, *J* = 7.4, 1.1 Hz, 1H), 2.29 (d, *J* = 1.4 Hz, 3H), 1.68 (s, 9H), 1.50 (s, 1H). **^{13}C-NMR:** (101 MHz, $CDCl_3$) δ 149.92, 135.57, 131.55, 124.29, 122.87, 122.38, 118.97, 116.42, 115.22, 83.22, 81.08, 28.32, 27.99, 9.69, 9.67.

Synthesis of 3-methyl-1-benzoyl-1*H*-indole (1m).

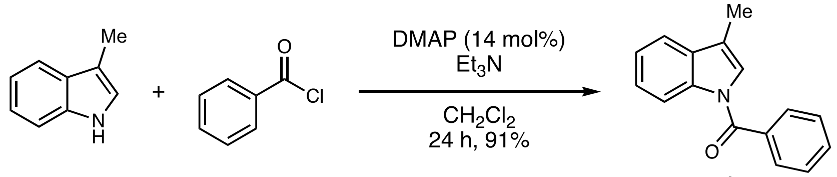

1m

Compound **1m** was obtained using the methodology described previously, by adding indole (1.00 g, 7.62 mmol, 1.00 eq), Et_3N (1.16 mg, 11.43 mmol, 1.50 eq), DMAP (130.4 mg, 1.07 mmol, 0.14 eq), and benzoyl chloride (1.29 mg, 9.15 mmol, 1.20 eq) in DCM (15.25 mL). After 24 hours of reaction, product **1m** was obtained (91%). **IR (KBr, cm^{-1}) ν:** 3052,

[8] S. W. Youn, T. Y. Ko, M. J. Jang, S. S. Jang, *Adv. Synth. Catal.* **2015**, *357*, 227–234.

2914, 2860, 1786, 1668, 1446. **^{1}H-NMR:** (400 MHz, $CDCl_3$) δ 8.61 (dd, *J* = 7.9, 1.3 Hz, 1H), 7.96 – 7.88 (m, 2H), 7.83 – 7.68 (m, 4H), 7.63 – 7.50 (m, 2H), 7.26 (q, *J* = 1.4 Hz, 1H), 2.45 (d, *J* = 1.4 Hz, 3H). **^{13}C-NMR:** (101 MHz, $CDCl_3$) δ 168.47, 136.41, 135.08, 131.93, 131.70, 129.09, 128.63, 125.09, 124.48, 123.80, 118.98, 117.96, 116.59, 9.76, 9.74. The spectroscopic data agree with the literature.[9]

[9] S. Pan, N. Ryu, T. Shibata, *J. Am. Chem. Soc.* **2012**, *134*, 17474–17477

2. Synthesis of donor-acceptor cyclopropanes (exemplified for the synthesis of dimethyl 2-(4-methoxyphenyl)cyclopropane-1,1-dicarboxylate (2a).

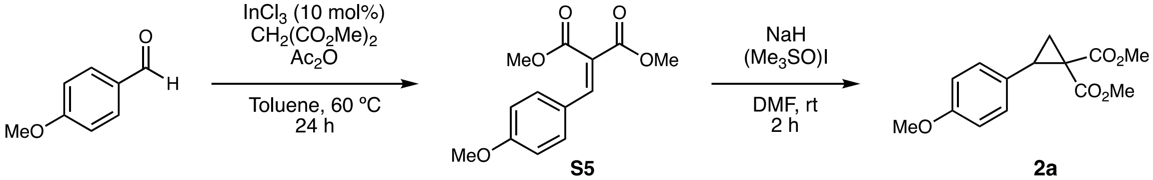

In a 25 mL round-bottom flask, $InCl_3$ (325 mg, 1.47 mmol, 0.10 eq), toluene (14.7 mL), anisaldehyde (2.0 g, 14.7 mmol, 1.00 eq), methyl malonate (2.13 g, 16.16 mmol, 1.10 eq), and acetic anhydride (1.65 g, 16.16 mmol, 1.10 eq) were added. The mixture was heated to 60 °C and stirred until the reaction was complete (monitored by TLC). Once the reaction was finished, a saturated solution of $NaHCO_3$ (20 mL) was added, and the mixture was then extracted with ethyl acetate (3 x 20 mL). The organic phase was dried over anhydrous Na_2SO_4, filtered, and concentrated using a rotary evaporator. The crude product was purified by column chromatography (10% EtOAc/hexane) to yield product **S5** (1.86 g, 90%).

A suspension of NaH (60% in mineral oil, 256.7 mg, 6.29 mmol, 1.20 eq) in DMF (10 mL) was prepared, and trimethylsulfoxonium iodide (TMSOI) (1.58 g, 7.19 mmol, 1.20 eq) was added. Gas evolution was observed for approximately 5 minutes. After 15 minutes, a solution of product **S5** (1.5 g, 5.99 mmol, 1.00 eq) in DMF (5 mL) was added, and the mixture was stirred for 2 hours. Then, a cold HCl solution (10 mL, 10%) was added and the resulting acidic solution (pH = 1-2) was extracted with diethyl ether (4 x 5 mL). The extract was washed with H_2O (4 x 5 mL), dried over anhydrous Na_2SO_4 and concentrated to yield product **2a** as a colorless oil (872 mg, 55%). **IR (KBr, cm^{-1}) ν:** 2997, 2953, 2837, 1724, 1612, 1517, 836. **^{1}H-NMR:** (300 MHz, $CDCl_3$) δ 7.16 – 7.05 (m, 2H), 6.83 – 6.74 (m, 2H), 3.76 (s, 3H), 3.75 (s, 3H), 3.37 (s, 3H), 3.22 – 3.12 (m, 1H), 2.14 (dd, J = 8.0, 5.1 Hz, 1H), 1.70 (dd, J = 9.3, 5.1 Hz, 1H). **^{13}C-NMR:** (75 MHz, $CDCl_3$) δ 170.33, 167.17, 158.99, 129.66, 126.53, 113.65, 55.22, 52.73, 52.23, 37.13, 32.22, 19.28. The spectroscopic data agree with the literature.[10]

[10] R. Goudreau, D. Marcoux, B. Charette, *J. Org. Chem.* **2009**, *74*, 470–473.

Dimethyl 2-phenylcyclopropane-1,1-dicarboxylate (2b).

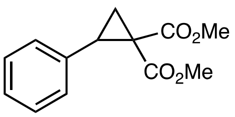

Yellow oil (70%) **IR (KBr, cm^{-1}) ν:** 3001, 2951, 2842, 1727, 1436, 1276, 1128, 697. **^{1}H-NMR:** (400 MHz, $CDCl_3$) δ 7.32 – 7.16 (m, 5H), 3.80 (s, 3H), 3.37 (s, 3H), 3.24 (t, *J* = 8.6 Hz, 1H), 2.21 (dd, *J* = 8.0, 5.2 Hz, 1H), 1.75 (dd, *J* = 9.2, 5.2 Hz, 1H). **^{13}C-NMR:** (101 MHz, $CDCl_3$) δ 170.34, 167.14, 134.67, 128.53, 128.26, 127.50, 52.91, 52.30, 37.33, 32.66, 19.20. **HRMS-ESI:** Calculated for $C_{13}H_{14}O_4Na$ $[M+Na]^+$ = 257.07843, Found: 257.0781. The spectroscopic data agree with the literature.[10]

Dimethyl 2-(4-chlorophenyl)cyclopropane-1,1-dicarboxylate (2c).

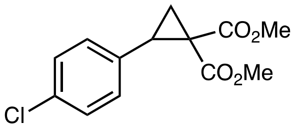

Pale yellow oil (97%) **IR (KBr, cm^{-1}) ν:** 2997, 2953, 2843, 1725, 1437, 1279, 836. **^{1}H-NMR:** (400 MHz, $CDCl_3$) δ 7.17 (d, *J* = 8.5 Hz, 2H), 7.05 (d, *J* = 8.3 Hz, 2H), 3.72 (s, 3H), 3.33 (s, 3H), 3.11 (dd, *J* = 9.2, 8.0 Hz, 1H), 2.08 (dd, *J* = 8.0, 5.3 Hz, 1H), 1.67 (dd, *J* = 9.3, 5.3 Hz, 1H). **^{13}C-MNR:** (101 MHz, $CDCl_3$) δ 170.11, 166.97, 133.44, 133.29, 129.96, 128.49, 53.01, 52.50, 37.31, 31.86, 19.25. **HRMS-ESI:** Calculated for $C_{13}H_{13}ClO_4Na$ $[M+Na]^+$ = 291.03946, Found: 291.0388. The spectroscopic data agree with the literature.[11]

Dimethyl 2-(4-bromophenyl)cyclopropane-1,1-dicarboxylate (2d).

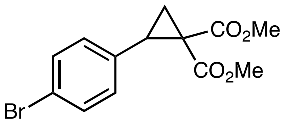

Colorless oil (48%) **IR (KBr, cm^{-1}) ν:** 2992, 2952, 2844, 1725, 1436, 1278, 833. **^{1}H-NMR:** (400 MHz, $CDCl_3$) δ 7.42 – 7.35 (m, 2H), 7.09 – 7.02 (m, 2H), 3.78 (s, 3H), 3.40 (s, 3H), 3.15 (t, *J* = 8.6 Hz, 1H), 2.14 (dd, *J* = 8.0, 5.3 Hz, 1H), 1.73 (dd, *J* = 9.2, 5.3 Hz, 1H). **^{13}C-NMR:** (101 MHz, $CDCl_3$) δ 170.08, 166.94, 133.82, 131.43, 130.30, 121.56, 53.01, 52.52, 37.27, 31.89, 19.20. **HRMS-ESI:** Calculated for $C_{13}H_{13}BrO_4Na$ $[M+Na]^+$ = 332.98894, Found: 332.9881. The spectroscopic data agree with the literature.[11]

[11] R. A. Novikov, A. V. Tarasova, V. A. Korolev, V. P. Timofeev, Y. V. Tomilov, *Angew. Chem. Int. Ed.* **2014**, *53*, 3187–3191.

Dimethyl 2-(4-fluorophenyl)cyclopropane-1,1-dicarboxylate (2e).

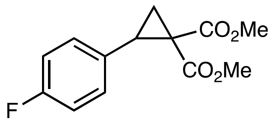

Pale yellow oil (59%) **IR (KBr, cm^{-1}) ν:** 2997, 2954, 2844, 1726, 1515, 1278, 1218, 843. **^{1}H-NMR:** (400 MHz, $CDCl_3$) δ 7.26 (dd, *J* = 8.6, 5.4 Hz, 2H), 7.06 (t, *J* = 8.7 Hz, 2H), 3.89 (s, 3H), 3.49 (s, 3H), 3.29 (t, *J* = 8.6 Hz, 1H), 2.25 (dd, *J* = 8.0, 5.2 Hz, 1H), 1.84 (dd, *J* = 9.3, 5.3 Hz, 1H). **^{13}C-NMR:** (101 MHz, $CDCl_3$) δ 170.20, 167.05, 162.25 (d, *J* = 246.19 Hz), 130.27 (d, *J* = 8.19 Hz), 115.22 (d, *J* = 21.56 Hz), 52.96, 52.41, 37.19, 31.85, 19.33. **HRMS-ESI:** Calculated for $C_{13}H_{13}FO_4Na$ $[M+Na]^+$ = 275.06901, Found: 275.0682. The spectroscopic data agree with the literature.[10]

Dimethyl 2-(2,4-dichlorophenyl)cyclopropane-1,1-dicarboxylate (2f).

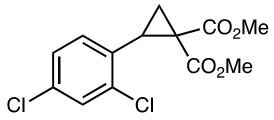

White solid (64%). **IR (KBr, cm^{-1}) ν:** 3040, 3007, 2955, 2921, 2851, 1723, 1587, 1435, 1281, 817. **^{1}H-NMR:** (400 MHz, $CDCl_3$) δ 7.38 (d, *J* = 2.1 Hz, 1H), 7.17 (dd, *J* = 8.3, 2.1 Hz, 1H), 7.03 (d, *J* = 8.3 Hz, 1H), 3.82 (s, 3H), 3.42 (s, 3H), 3.29 (t, *J* = 8.7 Hz, 1H), 2.20 (dd, *J* = 8.2, 5.3 Hz, 1H), 1.79 (dd, *J* = 9.1, 5.3 Hz, 1H). **^{13}C-NMR:** (101 MHz, $CDCl_3$) δ 169.79, 167.02, 137.27, 134.10, 131.61, 129.96, 129.26, 126.83, 36.48. **HRMS-ESI:** Calculated for $C_{13}H_{13}Cl_2O_4Na$ $[M+Na]^+$ = 325.00049, Found: 325.0005.

Dimethyl 2-(4-nitrophenyl)cyclopropane-1,1-dicarboxylate (2g).

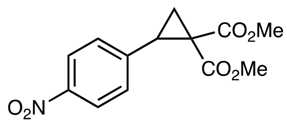

Pale yellow solid (57%) **IR (KBr, cm^{-1}) ν:** 3091, 3085, 3005, 2956, 2845, 1731, 1599, 1516, 1344, 857. **^{1}H-NMR:** (400 MHz, $CDCl_3$) δ 8.16 – 8.10 (m, 2H), 7.39 – 7.31 (m, 2H), 3.80 (s, 3H), 3.41 (s, 3H), 3.27 (t, *J* = 8.6 Hz, 1H), 2.22 (dd, *J* = 8.0, 5.5 Hz, 1H), 1.83 (dd, *J* = 9.1, 5.5 Hz, 1H). **^{13}C-NMR:** (101 MHz, $CDCl_3$) δ 169.65, 166.63, 147.36, 142.58, 129.47, 123.53, 53.23, 52.71, 37.83, 31.68, 19.46. **HRMS-ESI:** Calculated for $C_{13}H_{13}NO_6Na$ $[M+Na]^+$ = 302.059, Found: 302.0636. The spectroscopic data agree with the literature.[12]

[12] C. Perreault, S. R. Goudreau, L. E. Zimmer, A. B. Charette, *Org. Lett.* **2008**, *10*, 689–692.

Dimethyl (E)-2-styrylcyclopropane-1,1-dicarboxylate (2i).

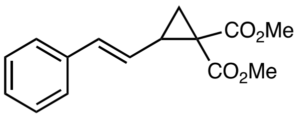

Orange oil (81%). **IR (KBr, cm^{-1}) ν:** 3024, 2952, 2844, 1725, 1436, 1281, 962, 693. **^{1}H-NMR:** (400 MHz, $CDCl_3$) δ 7.28 – 7.15 (m, 5H), 6.60 (d, *J* = 15.8 Hz, 1H), 5.77 (dd, *J* = 15.8, 8.7 Hz, 1H), 3.72 (s, 3H), 3.69 (s, 3H), 2.71 (q, *J* = 7.9 Hz, 1H), 1.81 (dd, *J* = 7.6, 5.0 Hz, 1H), 1.66 (dd, *J* = 9.0, 4.9 Hz, 1H). **^{13}C-NMR:** (101 MHz, $CDCl_3$) δ 170.09, 168.08, 136.80, 134.00, 128.71, 127.76, 126.29, 124.67, 52.91, 52.82, 36.17, 31.79, 21.45. **HRMS-ESI:** Calculated for $C_{15}H_{16}O_4Na$ $[M+Na]^+$ = 283.0941, Found: 283.0938. The spectroscopic data agree with the literature.[13]

Synthesis of dimethyl 2-vinylcyclopropane-1,1-dicarboxylate (2h).

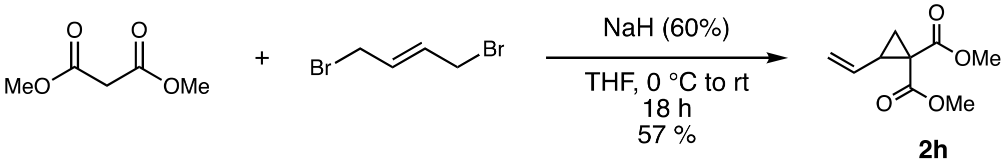

In a dry 50 mL round-bottom flask, NaH (60% in mineral oil, 666 mg, 16.65 mmol, 2.20 eq) and THF (17 mL) were added. The suspension was cooled to 0 °C, and then dimethyl malonate (1.0 g, 7.57 mmol, 1.00 eq) was carefully added. The suspension was stirred at room temperature for 45 minutes, and 1,4-dibromo-2-butene was added in one portion (1.78 g, 8.83 mmol, 1.10 eq), and the mixture was stirred for 24 hours. Upon completion of the reaction (confirmed by TLC), Et_2O (20 mL) and H_2O (20 mL) were added in equal portions, and the mixture was extracted with Et_2O (2 x 20 mL). The organic phase was washed with H_2O (20 mL) and brine (20 mL), dried over anhydrous Na_2SO_4 and concentrated using a rotary evaporator. The crude product was purified by column chromatography (20% EtOAc/hexane) to obtain product **2h** as a colorless oil (793 mg, 57%). **IR (KBr, cm^{-1}) ν:** 2939, 2923, 2852, 1731, 1442, 1438, 1274, 1129. **^{1}H-NMR:** (400 MHz, $CDCl_3$) δ 5.43 (ddd, *J* = 17.0, 10.1, 8.2 Hz, 1H), 5.34 – 5.25 (m, 1H), 5.18 – 5.11 (m, 1H), 3.74 (s, 6H), 2.63 – 2.54 (m, 1H), 1.72 (dd, *J* = 7.6, 4.9 Hz, 1H), 1.59 (dd, *J* = 9.0, 4.9 Hz, 1H). **^{13}C-NMR:** (101 MHz, $CDCl_3$) δ 170.17, 167.93, 133.11, 130.94, 118.85, 35.88, 31.63, 29.84, 20.75. **HRMS-**

[13] R. Talukdar, D. P. Tiwari, A. Saha, M. K. Ghorai, *Org. Lett.* **2014**, *16*, 3954–3957.

ESI: Calculated for $C_9H_{12}O_4Na$ $[M+Na]^+$ = 207.06278, Found: 207.06224. The spectroscopic data agree with the literature.[14]

3. Optimization studies.

Table S1: Screening of metal salt.[a]

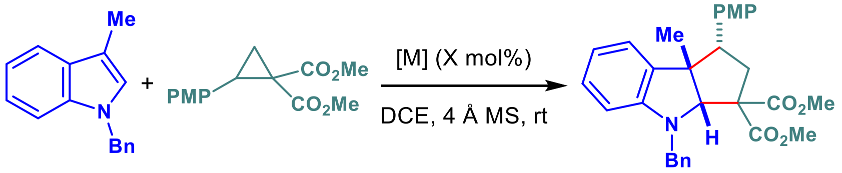

1a **2a** **3aa**

Entry	[M]	X mol%	t(h)	yield (%)[b]	Conv. (%)[c]	*dr*[d]
1	$Cu(OTf)_2$	20	24	79	>99	*5:1*
2	$Co(ClO_4)_2·6H_2O$	20	24	42	-	*6:1*
3[e]	$Zn(ClO_4)_2·6H_2O$	20	48	28	63	*6.4:1*
4	$Ni(ClO_4)_2·6H_2O$	20	24	95	>99	*6:1*
5	$Cu(ClO_4)_2·6H_2O$	20	24	70	>99	*7.8:1*
6	$MnCl_2$	20	24	8	25	-
7	$FeCl_2$	20	24	27	33	*4:1*
8	$FeCl_3$	20	24	3.7	51	-
9	$AlCl_3$	20	24	41	74	*4:1*
10	$Ni(ClO_4)_2·6H_2O$	10	24	48	47	*6:1*
11	$Ni(OAc)_2·4H_2O$	10	24	9.3	15	*5:1*
12	$Ni(OTf)_2$	10	24	78	>99	*5:1*
13	$NiSO_4·6H_2O$	10	24	56	-	*4:1*
14	$Ni(NO_3)_2·6H_2O$	10	24	48	-	*9:1*
15	$NiCl_2$	10	24	55	57	*3.6:1*
16	$Ni(acac)_2$	10	24	-	<5	-

[a] Reaction conditions: Indole **1a** (1.0 eq.) and cyclopropane **2a** (1.2 eq.), was treated with [M] (X mol%) in DCE (0.1 M) at rt and time (h). [b] Isolated yield. [c] Based on recovery of starting material. [d] Calculated from the ^1H NMR. [e] 24 h rt, then 24 h at 50 °C.

[14] A. T. Parsons, M. J. Campbell, J. S. Johnson, *Org. Lett.* **2008**, *10*, 2541–2544

Table S2: Screening of solvents.[a]

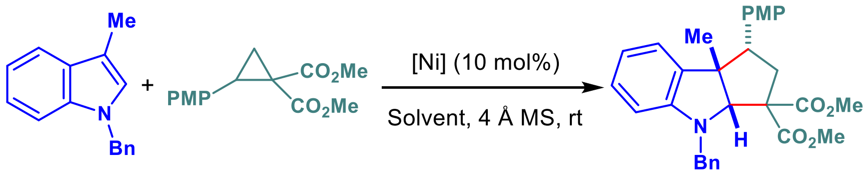

Entry	[Ni]	Solvent	t(h)	yield (%)[b]	Conv. (%)[c]	*dr*[d]
1	$Ni(OTf)_2$	DCE	24	78	>99	*5:1*
2	$Ni(OTf)_2$	CH_3CN	48	-	<5	-
3	$Ni(OTf)_2$	Toluene	24	29	76	*1:1*
4	$Ni(OTf)_2$	DCM	24	47	73	*7.7:1*
5	$Ni(OTf)_2$	$CHCl_3$	24	25	85	*1:1*
6	$Ni(OTf)_2$	AcOEt	48	-	<5	-
7	$Ni(OTf)_2$	THF	48	-	<5	-
8	$Ni(ClO_4)_2{\cdot}6H_2O$	AcOEt	48	33	60	*7.5:1*
9	$Ni(ClO_4)_2{\cdot}6H_2O$	CH_3CN	48	-	<5	-
10	-	DCE	48	-	0	-
11[e]	$Ni(ClO_4)_2{\cdot}6H_2O$	DCE		-	-	-

[a] Reaction conditions: Indole **1a** (1.0 eq.) and cyclopropane **2a** (1.2 eq.), was treated with [Ni] (10 mol%) in the indicated solvent (0.1 M) at rt and time (h). [b] Isolated yield. [c] Based on recovery of starting material. [d] Calculated from the ^{1}H-NMR. [e] Reaction with addition of water (1 ppm).

Table S3: Screening of indoles with Ni(OTf)$_2$ (10 mol%).[a]

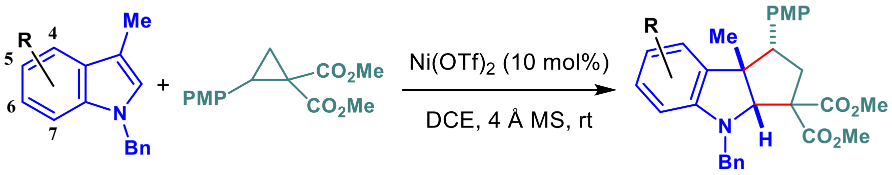

Entry	R (**1**)	t(h)	**3** yield (%)[b]	Conv. (%)[c]	*dr*[d]
1	H (**1a**)	24	78 (**3aa**)	>99	*5:1*
2	7-Me (**1b**)	192	- (**3ba**)	<5	-
3	5-OMe (**1c**)	144	44 (**3ca**)	90	*4:1*
4	5-Br (**1d**)	144	28 (**3da**)	73	*2:1*
5	5-Cl (**1e**)	120	30 (**3ea**)	>99	*3:1*
6	6-Cl (**1f**)	168	42 (**3fa**)	>99	*5:1*
7	**1h**	192	- (**3ha**)	<5	-
8	**1i**	192	- (**3ia**)	<5	-
9	**1j**	192	- (**3ja**)	<5	-

[a] Reaction conditions: Indole **1** (1.0 eq.) and cyclopropane **2a** (1.2 eq.), was treated with Ni(OTf)$_2$ (10 mol%) in DCE (0.1 M) at rt and time (h). [b] Isolated yield. [c] Based on recovery of starting material. [d] Calculated from the ^{1}H NMR. [e] 24 h rt, then 24 h at 50 °C.

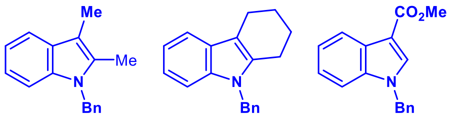

1h **1i** **1j**

Table S4: Screening of ligands.[a]

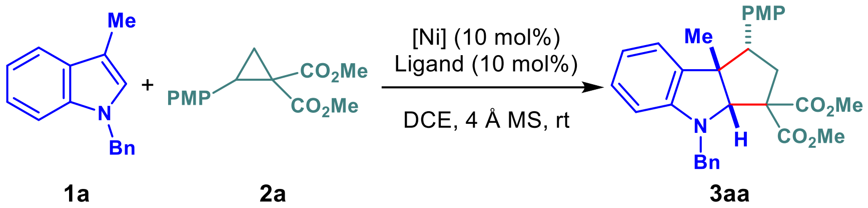

Entry	[Ni]	Ligand	T (ºC)	t (h)	Yield (%)[b]	Conv.(%)[c]	*dr*[d]
1	$Ni(OTf)_2$	Bipy	rt	144	-	<5	-
2	$Ni(OTf)_2$	Phen	85	72	12	26	*1:1*
3	$Ni(OTf)_2$	dppe	rt	144	-[e]	7.5	-
4	$Ni(OTf)_2$	d^Fppe	rt	144	13	24	*4:1*
5	$Ni(OTf)_2$	L_2-$PrPr_2$	50	72	-[e]	<5	-
6	$Ni(OTf)_2$	L_3-$PrPr_2$	50	72	-[e]	<5	-
7	$Ni(ClO_4)_2·6H_2O$	Phen	rt	96	16	63	*4.4:1*
8	$Ni(ClO_4)_2·6H_2O$	dppe	rt	96	19	-	*5.7:1*
9	$Ni(ClO_4)_2·6H_2O$	d^Fppe	rt	168	43	-	*5.5:1*
10	$Ni(ClO_4)_2·6H_2O$	$(C_6F_5)_3P$	rt	168	-	<5	-
11	$Ni(ClO_4)_2·6H_2O$	dppBz	rt	120	53	70	*5.5:1*
12	$Ni(ClO_4)_2·6H_2O$	dppf	rt	144	-	<5	-
13	$Ni(ClO_4)_2·6H_2O$	rac-BINAP	rt	5	73	>99	*6:1*
14	$Ni(OTf)_2$	rac-BINAP	rt	96	-	<5	-
15	$Ni(ClO_4)_2·6H_2O$	rac-BINAP	50	5	52	>99	
16	$Ni(ClO_4)_2·6H_2O$	rac-BINAP	80	5	28	>99	

[a] Reaction conditions: Indole **1a** (1.0 eq.) and cyclopropane **2a** (1.2 eq.), was treated with [Ni] (10 mol%) and ligand (10 mol%) in DCE (0.1 M) at rt and time (h). [b] Isolated yield. [c] Based on recovery of starting material. [d] Calculated from the ^{1}H NMR. [e] complex mixture.

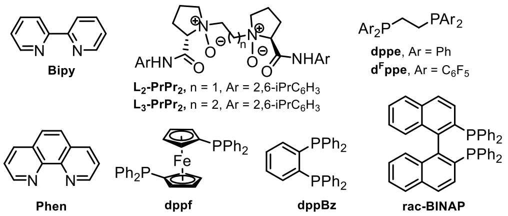

Table S5: Screening of chiral ligands.[a]

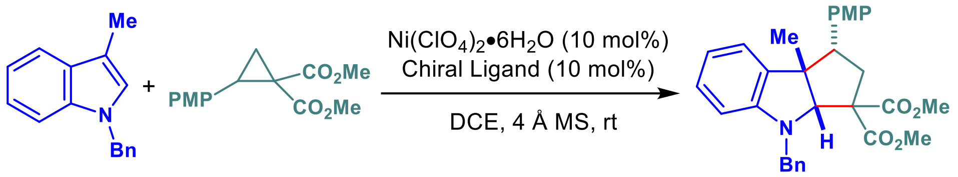

1a **2a** **3aa**

Entry	Chiral Ligand	t (h)	Yield (%)[b]	Conv.(%)[c]	*dr*[d]	*ee(%)*[e]
1	(R)-BINAP	20	76	>99	*5.7:1*	*7.1*
2	(R)-DM-BINAP	20	81	>99	*6.2:1*	*39.1*
3	(S)-DTBM-SEGPHOS	24	36	59	*5.2:1*	*12.5*

[a] Reaction conditions: Indole **1a** (1.0 eq.) and cyclopropane **2a** (1.2 eq.), was treated with $Ni(ClO_4)_2{\cdot}6H_2O$ (10 mol%) and chiral ligand (10 mol%) in DCE (0.1 M) at rt and time (h). [b] Isolated yield. [c] Based on recovery of starting material. [d] Calculated from the ^{1}H NMR. [e] complex mixture.

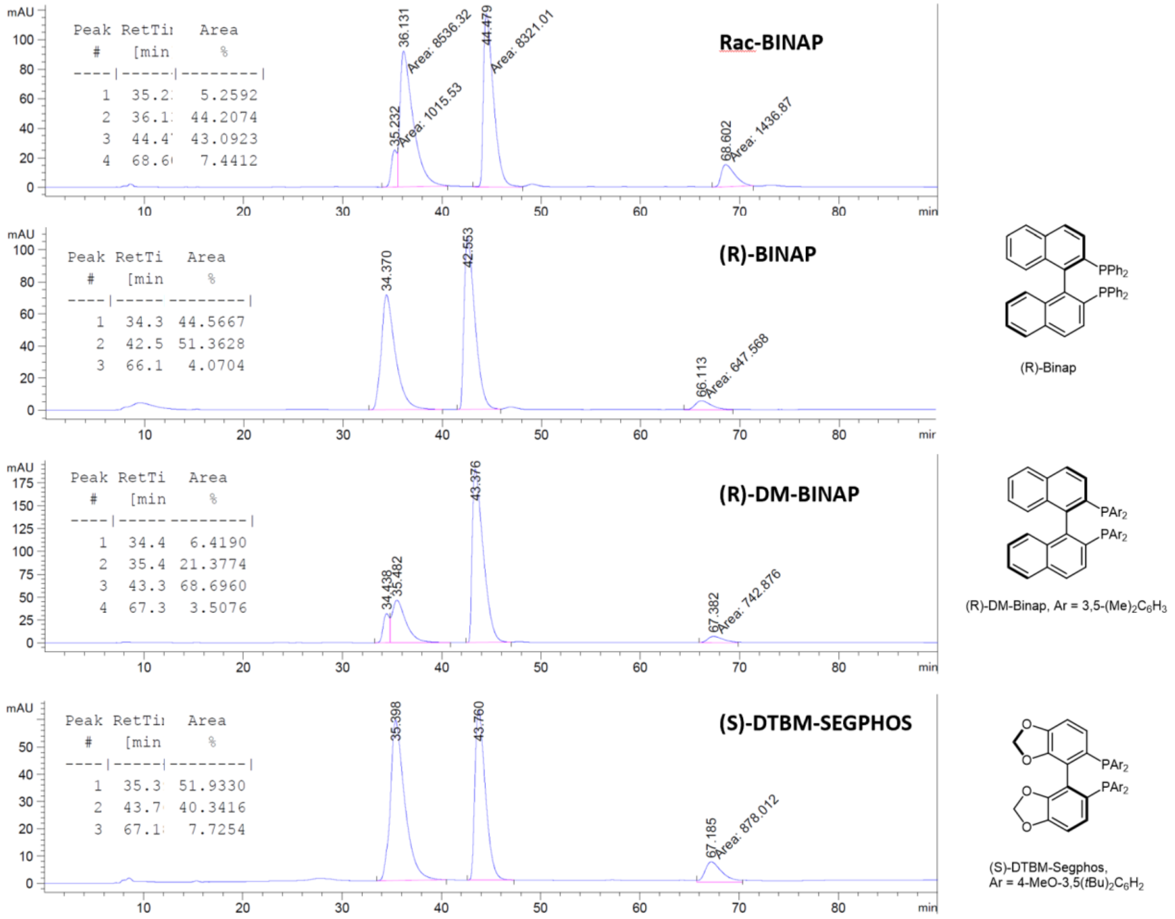

4. General procedure for [3+2] cycloaddition (exemplified for the synthesis of dimethyl (1*S**,3a*R**,8b*S**)-4-benzyl-1-(4-methoxyphenyl)-8b-methyl-1,3a,4,8b-tetrahydrocyclopenta[*b*]indole-3,3(2*H*)-dicarboxylate (3aa)):

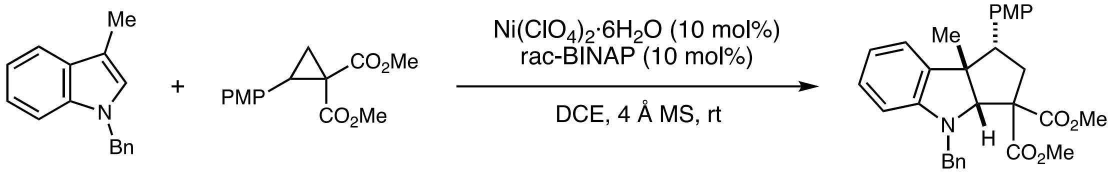

1a **2a** **3aa**

A solution of *N*-benzylskatole **1a** (20 mg, 90.37 μmol, 1.0 eq), cyclopropane **2a** (28.7 mg, 108.45 μmol, 1.2 eq), $Ni(ClO_4)_2{\cdot}6H_2O$ (1.65 mg, 4.52 μmol, 10 mol%), rac-BINAP (2.81 mg, 4.52 μmol, 10 mol%) and 400 mg of 4 Å molecular sieves in dry DCE (903 μL, 0.1 M), were stirred under a nitrogen atmosphere at room temperature for 5 h. After completing the reaction, as indicated by TLC, the mixture was filtered through celite® and washed AcOEt (50 mL). The filtrate was concentrated and purified by flash chromatography (SiO_2, 63-200 μm, 10% AcOEt/hexane) to afford cyclopenta[*b*]indole **3aa** (32 mg, 73% yield) as a colorless oil (*dr*=6:1). **IR (KBr, cm^{-1}) *ν*:** 2994, 2950, 2834, 1731, 1599, 1514, 1275. **^{1}H-NMR:** (500 MHz, $CDCl_3$) δ 7.24 – 7.17 (m, 2.43H), 7.17 – 7.10 (m, 2.76H), 7.05 (t, *J* = 9.5 Hz, 0.57H), 6.95 (t, *J* = 7.7 Hz, 0.20H), 6.90 – 6.78 (m, 2H), 6.73 (d, *J* = 8.2 Hz, 1.84H), 6.63 (d, *J* = 7.2 Hz, 0.14H), 6.59 (t, *J* = 7.3 Hz, 0.14H), 6.36 (d, *J* = 8.0 Hz, 0.14H), 6.32 (d, *J* = 7.9 Hz, 0.86H), 6.28 (t, *J* = 7.4 Hz, 0.86H), 5.47 (d, *J* = 7.5 Hz, 0.86H), 4.58 (d, *J* = 16.5 Hz, 0.18H), 4.55 – 4.49 (m, 1.78H), 4.25 (d, *J* = 16.3 Hz, 0.16H), 4.08 (d, *J* = 16.1 Hz, 0.89H), 3.75 (s, 0.5H), 3.73 (s, 2.79H), 3.71 (s, 0.36H), 3.69 (s, 2.52H), 3.39 (s, 2.56H), 3.34 (s, 0.44H), 2.90 (dd, *J* = 14.8, 4.6 Hz, 0.82H), 2.68 (dd, *J* = 14.8, 12.7 Hz, 0.90H), 2.60 (dd, *J* = 13.1, 5.7 Hz, 0.16H), 2.30 (t, *J* = 13.3 Hz, 0.21H), 2.26 – 2.21 (m, 0.94H), 1.26 (s, 2.82H), 1.18 (s, 1.53H). **^{13}C-NMR**: (126 MHz, $CDCl_3$) δ 172.84, 171.97, 169.21, 158.79, 153.45, 150.88, 138.77, 138.62, 137.21, 133.00, 130.82, 130.44, 130.08, 129.75, 128.45, 128.37, 127.86, 127.85, 127.76, 127.59, 127.05, 126.89, 125.95, 118.39, 118.21, 114.14, 113.99, 113.73, 113.50, 113.25, 109.67, 109.54, 82.33, 80.19, 65.30, 64.12, 57.57, 56.66, 55.39, 55.37, 55.13, 53.74, 53.41, 52.90, 52.55, 52.44, 39.36, 36.87, 29.83, 28.15. **HRMS-ESI:** Calculated for $C_{30}H_{32}NO_5$ $[M+H]^+$ = 486.2275, Found: 486.2286.

NMR data of the major isomer deduced from the 6:1 mixture: **^{1}H-NMR:** (500 MHz, $CDCl_3$) δ 7.24 – 7.09 (m, 6H), 6.89 – 6.78 (m, 2H), 6.73 (d, *J* = 8.3 Hz, 2H), 6.34 – 6.25 (m, 2H), 5.47 (d, *J* = 7.5 Hz, 1H), 4.52 (d, 2H), 4.08 (d, *J* = 16.1 Hz, 1H), 3.73 (s, 3H), 3.69 (s, 3H), 3.39 (s, 3H), 2.90 (dd, *J* = 14.8, 4.6 Hz, 1H), 2.73 – 2.63 (m, 1H), 2.23 (dd, *J* = 13.0, 4.4 Hz, 1H), 1.26 (s, 3H). **^{13}C-NMR:** (126 MHz, $CDCl_3$) δ 171.97, 169.21, 158.79, 153.45, 138.77, 133.00, 130.44, 130.08, 128.45, 128.37, 127.86, 127.83, 127.76, 127.59, 127.05, 125.95, 118.21, 113.73, 113.50, 113.25, 109.67, 80.19, 65.30, 57.57, 56.66, 55.39, 55.37, 52.90, 52.55, 52.44, 36.87, 29.83, 28.15. The spectroscopic data agree with the literature.[5]

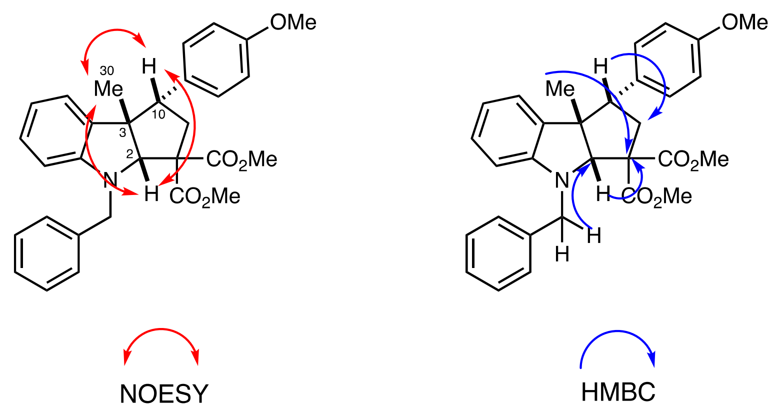

Figure 1: NOESY and HMBC observed for (**3aa**).

Dimethyl (1*S,3a*R**,8b*S**)-4-benzyl-8b-methyl-1-phenyl-1,3a,4,8b-tetrahydrocyclopenta[*b*]indole-3,3(2*H*)-dicarboxylate (3ab):**

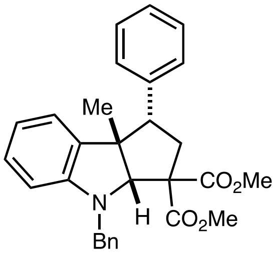

Colorless oil (90%, *dr = 4.8:1*). **IR (KBr, cm^{-1}) *ν*:** 3026, 2949, 2849, 1731, 1599, 1484, 1452, 1264, 698. **^{1}H-NMR:** (700 MHz, $CDCl_3$) δ 7.34 (t, *J* = 7.4 Hz, 0.74H), 7.32 – 7.25 (m, 3.26H), 7.24 – 7.21 (m, 1.38H), 7.21 – 7.18 (m, 1.69H), 7.08 – 7.01 (m, 1.67H), 7.02 (s, 0.21H), 6.91 (td, *J* = 7.6, 1.3 Hz, 0.95H), 6.72 (dd, *J* = 7.4, 1.4 Hz, 0.30H), 6.68 (d, *J* = 7.3 Hz, 0.32H), 6.45 (d, *J* = 8.0 Hz, 0.33H), 6.40 (d, *J* = 7.9 Hz, 0.81H), 6.35 – 6.30 (m, 0.85H), 5.49 – 5.44 (m, 0.74H), 4.71 (s, 0.26H), 4.66 (d, *J* = 16.3 Hz, 0.43H), 4.63 (d, *J* = 1.5 Hz, 0.71H), 4.60 (d, *J* = 16.1 Hz, 0.79H), 4.34 (d, *J* = 16.3 Hz, 0.35H), 4.16 (d, *J* = 16.0 Hz, 0.80H), 3.81 (s, 0.81H), 3.78 (s, 2.22H), 3.47 (s, 2.22H), 3.42 (s, 0.72H), 3.02 (dd, *J* = 14.8, 4.7 Hz, 0.79H), 2.82 (dd, *J* = 14.9, 12.8 Hz, 0.88H), 2.71 (dd, *J* = 13.1, 5.8 Hz, 0.37H), 2.44 (t, *J* = 13.4 Hz, 0.42H), 2.37 – 2.32 (m, 1H), 1.37 (s, 2.15H), 0.94 (s,

0.87H). **^{13}C-NMR:** (176 MHz, $CDCl_3$) δ 172.70, 171.84, 170.28, 170.22, 169.04, 167.07, 153.34, 150.77, 138.73, 138.65, 138.49, 138.27, 136.95, 134.61, 132.77, 129.10, 129.07, 128.64, 128.61, 128.46, 128.43, 128.35, 128.28, 128.27, 128.25, 128.20, 127.99, 127.80, 127.76, 127.64, 127.58, 127.46, 127.43, 127.34, 127.01, 126.94, 126.91, 126.78, 125.79, 125.68, 124.85, 122.79, 118.30, 118.07, 109.60, 109.45, 82.28, 80.18, 65.20, 64.06, 57.55, 56.60, 55.08, 53.97, 53.61, 53.14, 52.83, 52.81, 52.58, 52.46, 52.36, 52.23, 38.99, 36.44, 32.59, 29.72, 28.06, 22.92, 19.14. **HRMS-ESI:** Calculated for $C_{29}H_{30}NO_4$ $[M+H]^+$ = 456.2169, Found: 456.2181.

NMR data of the major isomer deduced from the 4.8:1 mixture: **^{1}H-NMR:** (700 MHz, $CDCl_3$) δ 7.32 – 7.25 (m, 4H), 7.24 – 7.21 (m, 2H), 7.21 – 7.18 (m, 2H), 7.08 – 7.01 (m, 2H), 6.91 (td, *J* = 7.6, 1.3 Hz, 1H), 6.40 (d, *J* = 7.9 Hz, 1H), 6.35 – 6.30 (m, 1H), 5.49 – 5.44 (m, 1H), 4.63 (d, *J* = 1.5 Hz, 1H), 4.60 (d, *J* = 16.1 Hz, 1H), 4.16 (d, *J* = 16.0 Hz, 1H), 3.78 (s, 3H), 3.47 (s, 3H), 3.02 (dd, *J* = 14.8, 4.7 Hz, 1H), 2.82 (dd, *J* = 14.9, 12.8 Hz, 1H), 2.37 – 2.32 (m, 1H), 1.37 (s, 3H). **^{13}C-NMR:** (176 MHz, $CDCl_3$) δ 171.84, 169.04, 153.34, 138.65, 138.27, 132.77, 129.10, 129.07, 128.46, 128.35, 128.27, 128.20, 127.99, 127.80, 127.76, 127.64, 127.46, 127.01, 126.94, 126.78, 125.68, 122.79, 118.30, 118.07, 109.60, 109.45, 80.18, 65.20, 57.55, 56.60, 53.14, 52.83, 52.81, 52.36, 36.44, 28.06. The spectroscopic data agree with the literature.[5]

Dimethyl (1*S,3a*R**,8b*S**)-4-benzyl-1-(4-chlorophenyl)-8b-methyl-1,3a,4,8b-tetrahydrocyclopenta[*b*]indole-3,3(2*H*)-dicarboxylate (3ac):**

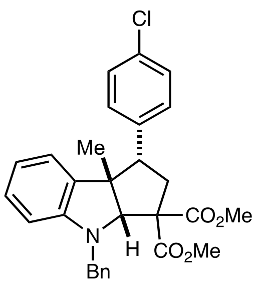

White solid (86%, *dr = 4:1*). **mp:** 117-120 °C. **IR (KBr, cm^{-1}) *ν*:** 3026, 2949, 2849, 1731, 1599, 1484, 1452, 1264, 698. **^{1}H-NMR:** (700 MHz, $CDCl_3$) δ 7.34 – 7.25 (m, 3H), 7.23 (d, *J* = 8.2 Hz, 2H), 7.18 (d, *J* = 7.7 Hz, 1.39H), 7.04 (d, *J* = 7.7 Hz, 0.41H), 7.00 – 6.91 (m, 1.42H), 6.45 (d, *J* = 8.0 Hz, 0.24H), 6.41 (d, *J* = 8.0 Hz, 0.70H), 6.38 (t, *J* = 7.4 Hz, 0.69H), 5.53 (d, *J* = 7.5 Hz, 0.76H), 4.71 (s, 0.19H), 4.65 (d, *J* = 16.3 Hz, 0.30H), 4.62 (s, 0.68H), 4.59 (d, *J* = 16.1 Hz, 0.71H), 4.32 (d, *J* = 16.3 Hz, 0.22H), 4.15 (d, *J* = 16.1 Hz, 0.76H), 3.81 (s, 0.57H), 3.78 (s, 2.08H), 3.47 (s, 2.11H), 3.42 (s, 0.58H), 2.98 (dd, *J* = 14.8, 4.7 Hz, 0.75H), 2.76 (dd, *J* = 14.7, 12.8 Hz, 0.82H), 2.69 (dd, *J* = 13.1, 5.7 Hz, 0.22H), 2.39 (d, *J* = 13.5 Hz, 0.27H), 2.31 (dd, *J* = 12.8, 4.7 Hz, 0.75H), 1.34 (s,

2.18H), 0.93 (s, 0.58H). **^{13}C-NMR:** (176 MHz, $CDCl_3$) δ 172.65, 171.84, 170.25, 169.03, 153.50, 150.95, 138.65, 138.51, 137.31, 136.97, 136.68, 132.94, 132.90, 132.51, 130.52, 130.45, 129.04, 128.79, 128.56, 128.50, 128.41, 128.28, 128.12, 128.07, 128.02, 127.93, 127.74, 127.58, 127.16, 127.12, 126.95, 125.87, 125.73, 124.98, 122.73, 122.19, 119.44, 118.63, 118.54, 118.37, 109.91, 109.81, 109.60, 82.38, 80.20, 65.27, 64.09, 57.63, 56.75, 55.18, 53.90, 53.56, 53.00, 52.82, 52.79, 52.69, 52.65, 52.53, 49.71, 47.05, 39.13, 36.74, 32.08, 29.85, 29.81, 28.07, 22.84, 22.72. **HRMS-ESI:** Calculated for $C_{29}H_{29}ClNO_4$ $[M+H]^+$ = 490.1780, Found: 490.1796.

NMR data of the major isomer deduced from the 4:1 mixture: **^{1}H-NMR:** (700 MHz, $CDCl_3$) δ 7.34 – 7.25 (m, 4H), 7.23 (d, *J* = 8.2 Hz, 3H), 7.18 (d, *J* = 7.7 Hz, 2H), 7.00 – 6.91 (m, 1H), 6.41 (d, *J* = 8.0 Hz, 1H), 6.38 (t, *J* = 7.4 Hz, 1H), 5.53 (d, *J* = 7.5 Hz, 1H), 4.62 (s, 1H), 4.59 (d, *J* = 16.1 Hz, 1H), 4.15 (d, *J* = 16.1 Hz, 1H), 3.78 (s, 3H), 3.47 (s, 3H), 2.98 (dd, *J* = 14.8, 4.7 Hz, 1H), 2.76 (dd, *J* = 14.7, 12.8 Hz, 1H), 2.31 (dd, *J* = 12.8, 4.7 Hz, 1H), 1.34 (s, 3H). **^{13}C-NMR:** (176 MHz, $CDCl_3$) δ 171.72, 168.90, 153.37, 138.52, 136.85, 132.81, 132.38, 130.39, 130.32, 128.67, 128.37, 128.28, 128.15, 127.99, 127.89, 127.62, 127.46, 126.99, 125.60, 118.24, 109.79, 80.07, 65.14, 57.50, 56.62, 52.87, 52.69, 52.41, 36.61, 27.94.

Further confirmation of the structure of **3ac** was obtained by X-ray crystallography (CCDC: 2302398) of single crystals obtained from hexane/EtOAc (9:1) mixture.

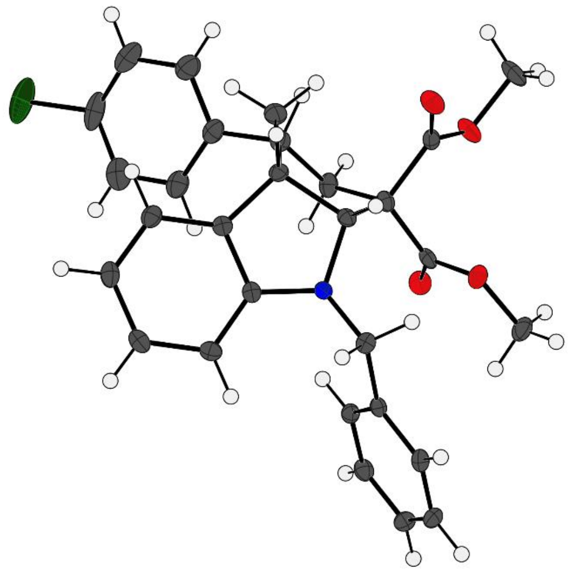

Dimethyl (1*S,3a*R**,8b*S**)-4-benzyl-1-(4-bromophenyl)-8b-methyl-1,3a,4,8b-tetrahydrocyclopenta[*b*]indole-3,3(2*H*)-dicarboxylate (3ad):**

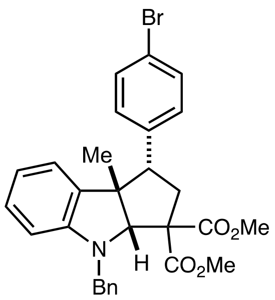

Yellow solid (83%., *dr = 3:1*). **IR (KBr, cm^{-1}) ν:** 2945, 2913, 2863, 1731, 1594, 1487, 1271, 756. **^{1}H-NMR:** (700 MHz, $CDCl_3$) δ 7.48 – 7.45 (m, 0.32H), 7.38 (d, *J* = 8.2 Hz, 1.59H), 7.31 – 7.25 (m, 1.76H), 7.24 – 7.21 (m, 0.85H), 7.18 (d, *J* = 7.7 Hz, 1.45H), 7.09 (dd, *J* = 8.4, 1.6 Hz, 0.30H), 7.08 – 7.06 (m, 0.17H), 7.04 (dd, *J* = 8.3, 5.2 Hz, 0.23H), 6.99 (d, *J* = 8.0 Hz, 0.11H), 6.94 (t, *J* = 7.8 Hz, 0.68H), 6.90 (d, *J* = 7.9 Hz, 1.09H), 6.80 (s, 0.16H), 6.68 – 6.67 (m, 0.28H), 6.45 (d, *J* = 8.1 Hz, 0.17H), 6.41 (d, *J* = 8.0 Hz, 0.58H), 6.38 (t, *J* = 7.4 Hz, 0.60H), 5.54 (d, *J* = 7.5 Hz, 0.54H), 4.71 (d, *J* = 1.5 Hz, 0.16H), 4.65 (d, *J* = 16.2 Hz, 0.18H), 4.62 (s, 0.58H), 4.59 (d, *J* = 16.0 Hz, 0.64H), 4.32 (d, *J* = 16.3 Hz, 0.17H), 4.15 (d, *J* = 16.0 Hz, 0.62H), 3.81 (s, 0.51H), 3.78 (s, 2.05H), 3.47 (d, *J* = 1.7 Hz, 1.84H), 3.42 (s, 0.64H), 2.97 (dd, *J* = 14.7, 4.6 Hz, 0.68H), 2.75 (t, *J* = 12.9 Hz, 0.70H), 2.69 (dd, *J* = 13.1, 5.5 Hz, 0.17H), 2.39 (d, *J* = 13.5 Hz, 0.15H), 2.31 (dd, *J* = 12.8, 4.6 Hz, 0.64H), 1.34 (s, 1.87H), 0.93 (s, 0.45H). **^{13}C-NMR:** (176 MHz, $CDCl_3$) δ 172.63, 171.83, 170.23, 169.01, 153.49, 150.95, 138.63, 138.50, 137.83, 137.49, 136.65, 132.47, 131.74, 131.47, 131.23, 130.96, 130.91, 130.84, 130.33, 129.41, 128.80, 128.55, 128.50, 128.47, 128.41, 128.13, 128.08, 127.93, 127.74, 127.58, 127.50, 127.12, 126.95, 125.87, 125.73, 122.73, 121.06, 121.02, 118.55, 118.39, 109.92, 109.82, 82.38, 80.18, 65.27, 64.08, 57.59, 56.74, 55.14, 53.90, 53.62, 53.04, 53.00, 52.89, 52.69, 52.66, 52.56, 52.53, 49.70, 39.05, 36.69, 31.95, 29.85, 28.06, 22.71 **HRMS-ESI:** Calculated for $C_{29}H_{29}BrNO_4$ $[M+H]^+$ = 534.1274, Found: 534.1257.

NMR data of the major isomer deduced from the 3:1 mixture: **^{1}H-NMR:** (700 MHz, $CDCl_3$) δ 7.38 (d, *J* = 8.2 Hz, 2H), 7.31 – 7.25 (m, 3H), 7.24 – 7.21 (m, 1H), 7.18 (d, *J* = 7.7 Hz, 2H), 6.94 (t, *J* = 7.8 Hz, 1H), 6.90 (d, *J* = 7.9 Hz, 1H), 6.41 (d, *J* = 8.0 Hz, 1H), 6.38 (t, *J* = 7.4 Hz, 1H), 5.54 (d, *J* = 7.5 Hz, 1H), 4.62 (s, 1H), 4.59 (d, *J* = 16.0 Hz, 1H), 4.15 (d, *J* = 16.0 Hz, 1H), 3.78 (d, *J* = 1.6 Hz, 3H), 3.47 (s, 3H), 2.97 (dd, *J* = 14.7, 4.6 Hz, 1H), 2.75 (t, *J* = 12.9 Hz, 1H), 2.31 (dd, *J* = 12.8, 4.6 Hz, 1H), 1.34 (s, 3H). **^{13}C-NMR:** (176 MHz, $CDCl_3$) δ 171.83, 169.01, 153.49, 138.63, 137.49, 132.47, 131.23, 130.96, 130.91, 130.84, 128.50,

128.41, 128.13, 127.74, 127.58, 127.12, 125.73, 121.06, 118.39, 109.92, 80.18, 65.27, 57.59, 56.74, 53.00, 52.89, 52.53, 36.69, 28.06.

Dimethyl (1*S,3a*R**,8b*S**)-4-benzyl-1-(4-fluorophenyl)-8b-methyl-1,3a,4,8b-tetrahydrocyclopenta[*b*]indole-3,3(2*H*)-dicarboxylate (3ae):**

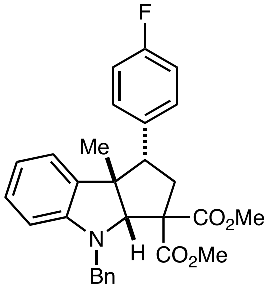

White solid (59%, *dr = 3.7:1*). **IR (KBr, cm^{-1}) *ν*:** 2995, 2995, 2920, 2848, 1731, 1600, 1513, 1273, 1222. **^{1}H-NMR:** (700 MHz, $CDCl_3$) δ 7.31 – 7.27 (m, 1.70H), 7.22 (t, *J* = 7.3 Hz, 0.91H), 7.20 – 7.15 (m, 3.11H), 7.11 (d, *J* = 7.7 Hz, 0.43H), 7.03 (t, *J* = 8.4 Hz, 0.68H), 6.96 (dd, *J* = 9.8, 7.5 Hz, 3.59H), 6.94 – 6.89 (m, 1.25H), 6.69 – 6.65 (m, 0.34H), 6.45 (d, *J* = 8.0 Hz, 0.31H), 6.40 (d, *J* = 8.0 Hz, 0.68H), 6.36 (td, *J* = 7.2, 2.9 Hz, 0.70H), 5.49 (d, *J* = 7.0 Hz, 0.64H), 4.71 (s, 0.23H), 4.65 (d, *J* = 16.3 Hz, 0.38H), 4.62 (s, 0.56H), 4.59 (d, *J* = 16.0 Hz, 0.68H), 4.32 (d, *J* = 16.3 Hz, 0.36H), 4.15 (d, *J* = 16.1 Hz, 0.74H), 3.81 (s, 0.49H), 3.79 (s, 2.26H), 3.42 (s, 0.49H), 3.39 (s, 2.26H), 2.99 (dd, *J* = 14.8, 4.6 Hz, 0.73H), 2.75 (dd, *J* = 14.8, 12.8 Hz, 0.78H), 2.69 (dd, *J* = 13.1, 5.6 Hz, 0.35H), 2.37 (t, *J* = 13.5 Hz, 0.39H), 2.35 – 2.29 (m, 0.79H), 1.34 (s, 1.58H), 0.92 (s, 0.53H). **^{13}C-NMR:** (176 MHz, $CDCl_3$) δ 171.88, 170.26, 169.08, 167.11, 162.29 (d, *J* = 246.13 Hz), 162.18 (d, *J* = 245.07 Hz)153.53, 138.69, 134.15, 132.65, 130.30 (d, *J* = 8.17 Hz), 128.50, 128.41, 128.06, 128.02, 127.75, 127.58, 127.11, 126.94, 125.74, 118.51, 118.31, 115.27 (d, *J* = 21.59 Hz), 114.70 (d, *J* = 21.00 Hz), 109.87, 109.77, 82.37, 80.21, 65.26, 64.08, 57.61, 56.76, 53.88, 53.42, 53.00, 52.99, 52.66, 52.64, 52.52, 52.46, 37.23, 36.87, 31.91, 29.86, 28.07, 19.39. **HRMS-ESI:** Calculated for $C_{29}H_{29}FNO_4$ $[M+H]^+$ = 474.2075, Found: 474.2052.

NMR data of the major isomer deduced from the 3.7:1 mixture: **^{1}H-NMR:** (700 MHz, $CDCl_3$) δ 7.30 – 7.27 (m, 2H), 7.20 – 7.14 (m, 4H), 6.96 (t, *J* = 8.6 Hz, 4H), 6.40 (d, *J* = 8.0 Hz, 1H), 6.36 (t, *J* = 7.2 Hz, 1H), 5.51 – 5.48 (m, 1H), 4.62 (s, 1H), 4.59 (d, *J* = 16.0 Hz, 1H), 4.15 (d, *J* = 16.1 Hz, 1H), 3.79 (s, 3H), 3.39 (s, 3H), 2.99 (dd, *J* = 14.8, 4.6 Hz, 1H), 2.75 (dd, *J* = 14.8, 12.8 Hz, 1H), 2.35 – 2.28 (m, 1H), 1.34 (s, 3H). **^{13}C-NMR:** (176 MHz, $CDCl_3$) δ 171.88, 170.26, 169.08, 167.11, 162.29 (d, *J* = 246.1 Hz), 153.53, 138.69, 132.65, 130.47, 130.45, 130.30 (d, *J* = 8.2 Hz), 128.50, 128.06, 127.75, 127.58, 127.11, 125.74,

118.31, 115.27 (d, *J* = 21.6 Hz), 114.76, 114.64, 109.87, 80.21, 65.26, 57.61, 56.76, 53.00, 52.99, 52.66, 52.52, 52.46, 37.23, 36.87, 31.91, 28.07, 19.39.

Dimethyl (1*S,3a*R**,8b*S**)-4-benzyl-1-(4-nitrophenyl)-8b-methyl-1,3a,4,8b-tetrahydrocyclopenta[*b*]indole-3,3(2*H*)-dicarboxylate (3ag):**

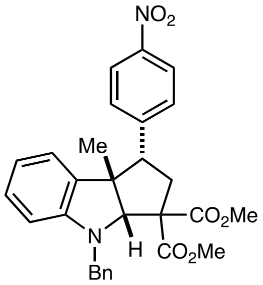

Yellow solid (51%, *dr = 3.5:1*) **mp:** 134-140 °C.. **IR (KBr, cm^{-1}) *ν*:** 3077, 3022, 2948, 1728, 1599, 1515, 1346. **^{1}H-NMR:** (700 MHz, $CDCl_3$) δ 8.15 (d, *J* = 8.5 Hz, 1.61H), 8.12 (d, *J* = 8.3 Hz, 1.15H), 7.36 (d, *J* = 8.3 Hz, 1.77H), 7.29 (t, *J* = 7.5 Hz, 1.81H), 7.23 (t, *J* = 7.4 Hz, 1.22H), 7.18 (t, *J* = 5.9 Hz, 2.85H), 6.97 – 6.92 (m, 0.79H), 6.70 (t, *J* = 7.4 Hz, 0.22H), 6.63 (d, *J* = 7.4 Hz, 0.18H), 6.48 (d, *J* = 8.0 Hz, 0.20H), 6.44 (d, *J* = 8.0 Hz, 0.73H), 6.34 (t, *J* = 7.4 Hz, 0.71H), 5.44 (d, *J* = 7.5 Hz, 0.59H), 4.73 (s, 0.17H), 4.66 (d, *J* = 16.6 Hz, 0.30H), 4.64 (s, 0.62H), 4.60 (d, *J* = 16.0 Hz, 0.76H), 4.32 (d, *J* = 16.3 Hz, 0.23H), 4.15 (d, *J* = 16.0 Hz, 0.78H), 3.83 (s, 0.71H), 3.81 (s, 2.34H), 3.53 (s, 0.34H), 3.42 (s, 2.69H), 3.12 (dd, *J* = 14.7, 4.8 Hz, 0.71H), 2.83 (dd, *J* = 14.7, 12.8 Hz, 0.85H), 2.74 (dd, *J* = 13.0, 5.5 Hz, 0.26H), 2.45 (t, *J* = 13.4 Hz, 0.28H), 2.39 – 2.34 (m, 0.78H), 1.38 (s, 2.16H), 0.94 (s, 0.49H). **^{13}C-NMR**: (176 MHz, $CDCl_3$) δ 171.63, 170.09, 169.68, 168.78, 166.65, 153.55, 147.39, 147.26, 147.23, 146.51, 142.60, 138.44, 131.89, 130.06, 129.92, 129.49, 128.78, 128.55, 128.53, 128.48, 128.45, 128.41, 127.73, 127.57, 127.31, 127.22, 127.04, 125.87, 125.28, 124.97, 123.80, 123.56, 123.33, 123.04, 122.59, 119.69, 118.78, 118.73, 118.51, 110.28, 110.16, 82.37, 80.21, 65.28, 64.14, 58.07, 56.82, 55.60, 54.04, 54.00, 53.44, 53.25, 53.11, 52.91, 52.80, 52.78, 52.74, 52.70, 52.64, 49.57, 47.11, 38.97, 37.86, 36.70, 31.71, 29.84, 29.81, 28.03, 22.55, 19.49, 9.79, 1.16. **HRMS-ESI:** Calculated for $C_{29}H_{29}N_2O_6$ $[M+H]^+$ = 501.2020, Found: 501.1997.

NMR data of the major isomer deduced from the 3.5:1 mixture: **^{1}H-NMR:** (700 MHz, $CDCl_3$) δ 8.15 (d, *J* = 8.5 Hz, 2H), 7.36 (d, *J* = 8.3 Hz, 2H), 7.29 (t, *J* = 7.5 Hz, 2H), 7.18 (t, *J* = 5.9 Hz, 3H), 6.97 – 6.92 (m, 1H), 6.44 (d, *J* = 8.0 Hz, 1H), 6.34 (t, *J* = 7.4 Hz, 1H), 5.44 (d, *J* = 7.5 Hz, 1H), 4.64 (s, 1H), 4.60 (d, *J* = 16.0 Hz, 1H), 4.15 (d, *J* = 16.0 Hz, 1H), 3.80 (s, 3H), 3.49 (s, 3H), 3.12 (dd, *J* = 14.7, 4.8 Hz, 1H), 2.83 (dd, *J* = 14.7, 12.8 Hz, 1H), 2.39 – 2.34 (m, 1H), 1.38 (s, 3H). **^{13}C-NMR**: (176 MHz, $CDCl_3$) δ 171.63, 169.68, 168.78, 166.65, 153.55, 146.51, 142.60, 138.44, 131.89, 130.06, 129.49, 128.55, 128.48, 128.45, 127.57,

127.22, 125.28, 123.56, 123.04, 118.51, 110.28, 80.21, 65.28, 58.07, 56.82, 53.44, 53.25, 53.11, 52.74, 52.64, 37.86, 36.70, 31.71, 29.84, 28.03, 19.49, 1.16.

Dimethyl (1*S,3a*R**,8b*S**)-4-benzyl-8b-methyl-1-((*E*)-styryl)-1,3a,4,8b-tetrahydrocyclopenta[*b*]indole-3,3(2*H*)-dicarboxylate (3ai):**

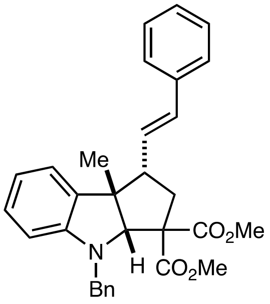

White solid (93%, *dr = 1.7:1*). **IR (KBr, cm^{-1}) ν:** 3023, 2949, 2916, 2847, 1731, 1598, 1483, 1259. **^{1}H-NMR:** (700 MHz, $CDCl_3$) δ 7.41 – 7.39 (m, 0.65H), 7.36 (d, *J* = 7.1 Hz, 1.48H), 7.34 – 7.32 (m, 1.76H), 7.29 – 7.27 (m, 2.58H), 7.25 – 7.20 (m, 2.25H), 7.19 (d, *J* = 7.6 Hz, 1.59H), 7.17 – 7.14 (m, 0.45H), 7.11 (t, *J* = 7.6 Hz, 0.69H), 7.08 – 7.05 (m, 0.30H), 7.05 – 7.01 (m, 0.89H), 6.99 (dd, *J* = 7.3, 1.3 Hz, 0.32H), 6.87 – 6.86 (m, 0.65H), 6.69 (q, *J* = 7.2 Hz, 0.92H), 6.50 – 6.43 (m, 1.84H), 6.25 (dd, *J* = 15.8, 8.6 Hz, 0.34H), 6.02 (dd, *J* = 15.8, 8.8 Hz, 0.65H), 4.63 (s, 0.40H), 4.59 (s, 0.60H), 4.58 (d, *J* = 6.7 Hz, 0.59H), 4.32 (d, *J* = 16.2 Hz, 0.31H), 4.17 (d, *J* = 16.1 Hz, 0.65H), 3.79 (s, 3H), 3.46 (s, 0.78H), 3.45 (s, 1.69H), 2.64 (dd, *J* = 13.23, 6.08 Hz, 0.31H), 2.51 (ddd, *J* = 13.8, 8.8, 4.9 Hz, 0.67H), 2.42 (t, *J* = 13.3 Hz, 0.76H), 2.27 (ddd, *J* = 12.7, 4.9, 1.5 Hz, 0.68H), 2.04 (t, *J* = 12.6 Hz, 0.36H), 1.33 (s, 1.96H), 1.26 (s, 0.81H). **^{13}C-NMR**: (176 MHz, $CDCl_3$) δ 172.73, 171.89, 170.24, 169.06, 153.43, 150.58, 138.70, 138.48, 137.54, 137.52, 137.38, 133.20, 132.21, 131.31, 129.87, 128.79, 128.74, 128.49, 128.41, 128.24, 128.20, 128.18, 128.15, 127.89, 127.53, 127.46, 127.06, 127.00, 126.37, 126.35, 125.55, 122.13, 121.74, 118.67, 118.60, 110.16, 109.35, 81.71, 80.16, 77.34, 77.16, 76.98, 72.26, 65.81, 65.36, 57.61, 56.59, 55.91, 53.25, 52.94, 52.92, 52.81, 52.53, 52.45, 52.03, 40.02, 38.35, 29.85, 27.74, 22.88. **HRMS-ESI:** Calculated for $C_{30}H_{32}NO_4$ $[M+H]^+$ = 482.2326, Found: 482.2298.

NMR data of the major isomer deduced from the 1.7:1 mixture: **^{1}H-NMR:** (700 MHz, $CDCl_3$) δ 7.41 – 7.39 (m, 1H), 7.36 (d, *J* = 7.1 Hz, 2H), 7.34 – 7.32 (m, 2H), 7.29 – 7.27 (m, 3H), 7.19 (d, *J* = 7.6 Hz, 2H), 6.87 – 6.86 (m, 1H), 6.69 (q, *J* = 7.2 Hz, 1H), 6.50 – 6.43 (m, 2H), 6.02 (dd, *J* = 15.8, 8.8 Hz, 1H), 4.59 (d, *J* = 1.4 Hz, 1H), 4.58 (d, *J* = 6.7 Hz, 1H), 4.17 (d, *J* = 16.1 Hz, 1H), 3.79 (s, 3H), 3.45 (s, 3H), 2.51 (ddd, *J* = 13.8, 8.8, 4.9 Hz, 1H), 2.42 (t, *J* = 13.3 Hz, 1H), 2.27 (ddd, *J* = 12.7, 4.9, 1.5 Hz, 1H), 1.33 (s, 3H). **^{13}C-NMR:** (176 MHz, $CDCl_3$) δ 171.89, 169.06, 153.43, 138.70, 137.38, 133.20, 132.21, 131.31, 129.87, 128.79,

128.49, 127.53, 126.35, 125.55, 122.13, 118.67, 110.16, 80.16, 65.81, 57.61, 56.59, 52.94, 52.45, 52.03, 38.35, 27.74.

Dimethyl (1*S,3a*R**,8b*S**)-4-benzyl-7-methoxy-1-(4-methoxyphenyl)-8b-methyl-1,3a,4,8b-tetrahydrocyclopenta[*b*]indole-3,3(2*H*)-dicarboxylate (3ca):**

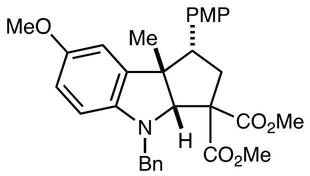

Colorless oil (83%, *dr = 4:1*). **IR (KBr, cm^{-1}) ν:** 2951, 2916, 2833, 1731, 1610, 1514, 1261, 1013. **^{1}H-NMR:** (700 MHz, $CDCl_3$) δ 7.30 – 7.27 (m, 4H), 7.24 – 7.18 (m, 4H), 7.13 (dd, *J* = 9.6, 2.9 Hz, 0.61H), 6.96 (d, *J* = 7.4 Hz, 1.33H), 6.87 (d, *J* = 8.6 Hz, 0.46H), 6.81 (d, *J* = 8.4 Hz, 1.45H), 6.58 (dd, *J* = 8.5, 2.7 Hz, 0.22H), 6.49 (dd, *J* = 8.6, 2.7 Hz, 0.72H), 6.32 (d, *J* = 8.6 Hz, 0.22H), 6.30 (d, *J* = 8.6 Hz, 0.68H), 6.28 (d, *J* = 2.7 Hz, 0.18H), 5.12 (d, *J* = 2.7 Hz, 0.70H), 4.69 (s, 0.22H), 4.60 (d, *J* = 16.2 Hz, 0.25H), 4.57 (s, 0.70H), 4.52 (d, *J* = 15.8 Hz, 0.80H), 4.23 (d, *J* = 16.1 Hz, 0.27H), 4.09 (d, *J* = 15.8 Hz, 0.81H), 3.82 (s, 0.67H), 3.79 (s, 2.25H), 3.77 (s, 1.94H), 3.68 (s, 0.56H), 3.48 (s, 1.97H), 3.40 (s, 0.57H), 3.34 (s, 2.22H), 2.97 (dd, *J* = 14.8, 4.6 Hz, 0.75H), 2.76 (dd, *J* = 14.8, 12.8 Hz, 0.82H), 2.71 (dd, *J* = 13.1, 5.7 Hz, 0.25H), 2.41 – 2.36 (m, 0.38H), 2.34 – 2.25 (m, 0.95H), 1.31 (s, 2.48H), 0.93 (s, 0.56H). **^{13}C-NMR:** (176 MHz, $CDCl_3$) δ 172.91, 172.07, 170.36, 169.20, 158.89, 158.77, 153.28, 152.76, 147.62, 145.34, 139.03, 138.91, 138.39, 134.37, 130.62, 130.49, 130.09, 130.02, 129.90, 129.76, 129.49, 129.15, 128.84, 128.44, 128.34, 127.81, 127.79, 127.54, 127.34, 127.04, 126.83, 124.98, 114.57, 114.15, 113.74, 113.48, 113.36, 113.30, 112.67, 111.34, 110.81, 110.41, 109.92, 83.14, 80.82, 65.43, 64.01, 58.30, 57.67, 56.02, 55.69, 55.47, 55.45, 55.42, 55.40, 53.41, 52.92, 52.91, 52.72, 52.58, 52.53, 52.44, 39.46, 36.67, 29.85, 27.92, 22.38, 14.27. **HRMS-ESI:** Calculated for $C_{31}H_{34}NO_6$ $[M+H]^+$ = 516.2381, Found: 516.2376.

NMR data of the major isomer deduced from the 4:1 mixture: **^{1}H-NMR:** (700 MHz, $CDCl_3$) δ 7.30 – 7.27 (m, 3H), 7.23 – 7.18 (m, 3H), 6.96 (d, *J* = 7.5 Hz, 2H), 6.81 (d, *J* = 8.4 Hz, 2H), 6.49 (dd, *J* = 8.6, 2.7 Hz, 1H), 6.30 (d, *J* = 8.6 Hz, 1H), 5.12 (d, *J* = 2.7 Hz, 1H), 4.56 (d, *J* = 1.5 Hz, 1H), 4.52 (d, *J* = 15.8 Hz, 1H), 4.09 (d, *J* = 15.8 Hz, 1H), 3.79 (s, 3H), 3.77 (s, 3H), 3.48 (s, 3H), 3.34 (s, 3H), 2.97 (dd, *J* = 14.8, 4.6 Hz, 1H), 2.76 (dd, *J* = 14.8, 12.7 Hz, 1H), 2.30 (ddt, *J* = 15.5, 4.3, 2.4 Hz, 1H), 1.31 (s, 3H). **^{13}C-NMR:** (176 MHz, $CDCl_3$) δ 172.07, 169.20, 158.89, 152.76, 147.62, 139.03, 134.37, 130.49, 130.09, 128.44, 128.34, 127.81,

127.79, 127.04, 114.57, 113.48, 113.36, 111.34, 110.81, 80.82, 65.43, 58.30, 57.67, 55.69, 55.47, 55.40, 52.91, 52.53, 52.44, 36.67, 29.85, 27.92. The spectroscopic data agree with the literature.[5]

Dimethyl (1*S,3a*R**,8b*S**)-4-benzyl-7-bromo-1-(4-methoxyphenyl)-8b-methyl-1,3a,4,8b-tetrahydrocyclopenta[*b*]indole-3,3(2*H*)-dicarboxylate (3da):**

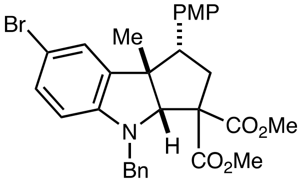

Colorless oil (57%, *dr = 4:1*). **IR (KBr, cm^{-1}) ν:** 2927, 2917, 2848, 1731, 1610, 1514, 1260. **^{1}H-NMR:** (700 MHz, $CDCl_3$) δ 7.31 – 7.27 (m, 1.78H), 7.25 – 7.21 (m, 1.37H), 7.17 – 7.14 (m, 1.37H), 7.14 – 7.12 (m, 0.48H), 7.12 – 7.10 (m, 0.28H), 7.00 (dd, *J* = 8.4, 2.1 Hz, 0.70H), 6.96 – 6.92 (m, 1.13H), 6.90 (d, *J* = 8.6 Hz, 0.57H), 6.87 – 6.83 (m, 1.33H), 6.79 (d, *J* = 2.1 Hz, 0.28H), 6.76 (d, *J* = 8.7 Hz, 0.14H), 6.31 (d, *J* = 8.4 Hz, 0.21H), 6.26 (d, *J* = 8.5 Hz, 0.70H), 5.49 (d, *J* = 2.1 Hz, 0.74H), 4.68 (s, 0.20H), 4.62 (d, *J* = 1.5 Hz, 0.78H), 4.55 (d, *J* = 16.0 Hz, 0.77H), 4.32 (d, *J* = 16.1 Hz, 0.38H), 4.11 (d, *J* = 16.0 Hz, 0.76H), 3.84 (s, 0.72H), 3.83 (s, 1.93H), 3.81 (s, 0.71H), 3.77 (s, 1.93H), 3.52 (s, 1.87H), 3.47 (s, 0.55H), 2.95 (dd, *J* = 14.8, 4.7 Hz, 0.75H), 2.71 (dd, *J* = 14.8, 12.9 Hz, 0.79H), 2.65 (dd, *J* = 13.1, 5.8 Hz, 0.28H), 2.34 – 2.30 (m, 0.81H), 2.31 – 2.26 (m, 0.57H), 1.29 (s, 2.25H), 0.88 (s, 0.72H). **^{13}C-NMR:** (176 MHz, $CDCl_3$) δ 172.63, 171.81, 170.26, 169.07, 159.17, 158.89, 152.21, 149.66, 139.62, 138.09, 137.96, 135.39, 130.55, 130.51, 130.38, 130.03, 130.00, 129.78, 129.49, 129.06, 128.93, 128.84, 128.64, 128.60, 128.51, 127.79, 127.63, 127.54, 127.33, 127.17, 126.84, 125.90, 125.81, 124.98, 114.15, 113.71, 113.50, 110.98, 110.69, 110.04, 109.95, 82.01, 80.00, 65.25, 64.21, 57.73, 56.13, 55.61, 55.42, 54.89, 53.33, 53.04, 53.02, 53.00, 52.73, 52.71, 52.67, 52.65, 52.55, 52.51, 41.29, 39.16, 36.69, 32.08, 29.85, 29.81, 29.51, 27.71, 23.32, 22.84, 14.27, 1.17. **HRMS-ESI:** Calculated for $C_{30}H_{31}BrNO_5$ $[M+H]^+$ = 564.1380, Found: 564.1361.

NMR data of the major isomer deduced from the 4:1 mixture: **^{1}H-NMR:** (700 MHz, $CDCl_3$) δ 7.31 – 7.27 (m, 2H), 7.25 – 7.21 (m, 2H), 7.17 – 7.14 (m, 2H), 7.00 (dd, *J* = 8.4, 2.1 Hz, 1H), 6.96 – 6.92 (m, 1H), 6.87 – 6.83 (m, 2H), 6.26 (d, *J* = 8.5 Hz, 1H), 5.49 (d, *J* = 2.1 Hz, 1H), 4.62 (d, *J* = 1.5 Hz, 1H), 4.55 (d, *J* = 16.0 Hz, 1H), 4.11 (d, *J* = 16.0 Hz, 1H), 3.83 (s, 3H), 3.77 (s, 3H), 3.52 (s, 3H), 2.95 (dd, *J* = 14.8, 4.7 Hz, 1H), 2.71 (dd, *J* = 14.8, 12.9 Hz, 1H), 2.34 – 2.30 (m, 1H), 1.29 (s, 3H). **^{13}C-NMR:** (176 MHz, $CDCl_3$) δ 171.81, 169.07,

159.17, 152.21, 138.09, 135.39, 130.51, 130.03, 130.00, 129.78, 129.06, 128.60, 128.51, 127.79, 127.63, 127.33, 113.71, 113.50, 110.98, 109.95, 80.00, 65.25, 57.73, 56.12, 55.61, 53.00, 52.55, 52.51, 36.69, 29.85, 27.71. The spectroscopic data agree with the literature.[5]

Dimethyl (1*S,3a*R**,8b*S**)-4-benzyl-7-chloro-1-(4-methoxyphenyl)-8b-methyl-1,3a,4,8b-tetrahydrocyclopenta[*b*]indole-3,3(2*H*)-dicarboxylate (3ea):**

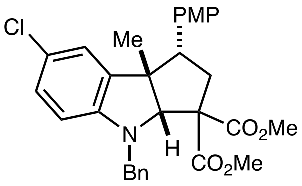

Colorless oil (58%, *dr = 5:1*). **IR (KBr, cm^{-1}) *ν*:** 3024, 2950, 2834, 1731, 1599, 1514, 1246. **^{1}H-NMR:** (700 MHz, $CDCl_3$) δ 7.32 – 7.24 (m, 4H), 7.24 – 7.16 (m, 3H), 7.04 – 7.01 (m, 0.29H), 6.92 (td, *J* = 7.7, 1.3 Hz, 1H), 6.88 (d, *J* = 8.6 Hz, 0.40H), 6.82 – 6.79 (m, 1.52H), 6.44 (d, *J* = 7.9 Hz, 0.19H), 6.40 (d, *J* = 7.9 Hz, 0.78H), 6.36 (td, *J* = 7.4, 1.0 Hz, 0.80H), 5.54 (dd, *J* = 7.5, 1.2 Hz, 0.81H), 4.70 (s, 0.21H), 4.65 (d, *J* = 16.3 Hz, 0.25H), 4.61 (d, *J* = 1.4 Hz, 0.65H), 4.59 (d, *J* = 16.1 Hz, 0.88H), 4.33 (d, *J* = 16.3 Hz, 0.25H), 4.16 (d, *J* = 16.1 Hz, 0.78H), 3.83 (s, 0.71H), 3.81 (s, 1.90H), 3.81 (s, 0.66H), 3.77 (s, 2.05H), 3.47 (s, 2.1H), 3.42 (s, 0.47H), 2.97 (dd, *J* = 14.8, 4.7 Hz, 0.80H), 2.75 (dd, *J* = 14.8, 12.8 Hz, 0.83H), 2.68 (dd, *J* = 13.1, 5.7 Hz, 0.24H), 2.31 (ddd, *J* = 12.6, 4.7, 1.5 Hz, 0.85H), 2.22 (t, *J* = 6.8 Hz, 0.14H), 1.34 (s, 2H), 0.93 (s, 0.60H). **^{13}C-NMR:** (176 MHz, $CDCl_3$) δ 172.86, 172.00, 170.38, 169.22, 158.79, 158.74, 153.48, 150.91, 138.80, 138.64, 137.20, 133.02, 130.83, 130.45, 130.10, 129.19, 128.47, 128.38, 127.87, 127.85, 127.77, 127.60, 127.06, 126.90, 125.97, 125.45, 124.98, 122.84, 118.41, 118.22, 113.50, 113.26, 109.69, 109.57, 82.37, 80.22, 65.31, 64.12, 57.59, 56.70, 55.41, 55.40, 55.15, 53.79, 53.41, 52.95, 52.92, 52.57, 52.56, 52.47, 39.37, 36.87, 29.85, 28.16, 22.97, 21.61. **HRMS-ESI:** Calculated for $C_{30}H_{31}ClNO_5$ $[M+H]^+$ = 520.1885, Found: 520.1854.

NMR data of the major isomer deduced from the 5:1 mixture: **^{1}H-NMR:** (700 MHz, $CDCl_3$) δ 7.32 – 7.24 (m, 3H), 7.22 (t, *J* = 7.3 Hz, 1H), 7.24 – 7.16 (m, 2H), 6.99 – 6.86 (m, 1H), 6.82 – 6.79 (m, 2H), 6.40 (d, *J* = 7.9 Hz, 1H), 6.36 (td, *J* = 7.4, 1.0 Hz, 1H), 5.54 (dd, *J* = 7.5, 1.2 Hz, 1H), 4.61 (d, *J* = 1.4 Hz, 1H), 4.59 (d, *J* = 16.1 Hz, 1H), 4.16 (d, *J* = 16.1 Hz, 1H), 3.81 (s, 3H), 3.77 (s, 3H), 3.47 (s, 3H), 2.97 (dd, *J* = 14.8, 4.7 Hz, 1H), 2.75 (dd, *J* = 14.8, 12.8 Hz, 1H), 2.31 (ddd, *J* = 12.6, 4.7, 1.5 Hz, 1H), 1.34 (s, 3H). **^{13}C-NMR:** (176 MHz, $CDCl_3$) δ 171.99, 169.22, 158.79, 153.48, 138.80, 133.02, 130.45, 130.10, 129.19, 128.47, 128.38, 127.87, 127.77, 127.60, 127.06, 125.97, 118.22, 113.50, 113.26, 109.69, 80.22,

65.31, 57.59, 56.70, 55.41, 52.92, 52.56, 52.47, 36.87, 28.16. The spectroscopic data agree with the literature.[5]

Dimethyl (1*S,3a*R**,8b*S**)-4-benzyl-6-chloro-1-(4-methoxyphenyl)-8b-methyl-1,3a,4,8b-tetrahydrocyclopenta[*b*]indole-3,3(2*H*)-dicarboxylate (3fa):**

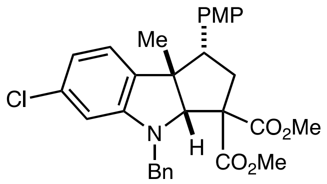

Pale yellow oil (73%, *dr = 8.6:1*). **IR (KBr, cm^{-1}) ν:** 3000, 2978, 2952, 2919, 2847, 1731, 1594, 1514, 1247. **^{1}H-NMR:** (700 MHz, $CDCl_3$) δ 7.32 – 7.22 (m, 1.80H), 7.24 (t, *J* = 7.4 Hz, 1H), 7.18 – 7.15 (m, 2H), 7.12 (dd, *J* = 8.8, 2.6 Hz, 0.82H), 7.08 – 7.04 (m, 0.30H), 6.95 (d, *J* = 8.0 Hz, 1.34H), 6.89 – 6.87 (m, 0.28H), 6.83 – 6.78 (m, 1.87H), 6.62 (d, *J* = 1.3 Hz, 0.20H), 6.43 (s, 0.14H), 6.38 (d, *J* = 1.8 Hz, 0.82H), 6.31 (dd, *J* = 8.0, 1.9 Hz, 0.85H), 5.40 (d, *J* = 8.0 Hz, 0.82H), 4.69 (s, 0.14H), 4.64 (d, *J* = 1.4 Hz, 0.85H), 4.61 (d, *J* = 16.3 Hz, 0.21H), 4.56 (d, *J* = 16.1 Hz, 0.89H), 4.35 (d, *J* = 16.3 Hz, 0.20H), 4.13 (d, *J* = 16.1 Hz, 0.89H), 3.82 (s, 0.32H), 3.81 (s, 2.34H), 3.80 (s, 0.34H), 3.77 (s, 2.28H), 3.50 (s, 2.44H), 3.48 (s, 0.30H), 2.94 (dd, *J* = 14.8, 4.6 Hz, 0.88H), 2.72 (dd, *J* = 14.8, 12.8 Hz, 0.96H), 2.64 (dd, *J* = 13.1, 5.8 Hz, 0.20H), 2.31 (ddd, *J* = 12.8, 4.7, 1.5 Hz, 0.92H), 1.29 (s, 2.49H), 0.87 (s, 0.52H). **^{13}C-NMR:** (176 MHz, $CDCl_3$) δ 172.62, 171.80, 170.49, 170.24, 169.06, 167.32, 167.08, 159.06, 158.90, 154.43, 137.90, 137.77, 136.02, 133.74, 131.61, 130.55, 130.04, 130.02, 129.76, 129.49, 129.19, 129.04, 128.84, 128.64, 128.56, 128.38, 128.04, 127.79, 127.58, 127.54, 127.36, 127.22, 126.66, 126.61, 125.45, 124.98, 123.51, 118.12, 118.09, 114.15, 113.74, 113.62, 113.37, 109.57, 109.28, 82.02, 80.10, 65.12, 64.19, 57.65, 57.15, 55.70, 55.42, 55.41, 55.36, 54.53, 53.43, 53.02, 53.00, 52.90, 52.71, 52.66, 52.56, 52.40, 52.32, 50.12, 41.29, 39.17, 37.23, 36.82, 32.37, 29.85, 29.81, 27.92, 23.32, 19.43, 1.17. **HRMS-ESI:** Calculated for $C_{30}H_{31}ClNO_5$ $[M+H]^+$ = 520.1885, Found: 520.1854.

NMR data of the major isomer deduced from the 8.6:1 mixture: **^{1}H-NMR:** (700 MHz, $CDCl_3$) δ 7.32 – 7.22 (m, 2H), 7.18 – 7.15 (m, 1H), 7.12 (dd, *J* = 8.8, 2.6 Hz, 2H), 6.95 (d, *J* = 8.0 Hz, 1H), 6.83 – 6.78 (m, 1H), 6.38 (d, *J* = 1.8 Hz, 2H), 6.31 (dd, *J* = 8.0, 1.9 Hz, 1H), 5.40 (d, *J* = 8.0 Hz, 1H), 4.64 (d, *J* = 1.4 Hz, 1H), 4.56 (d, *J* = 16.1 Hz, 1H), 4.13 (d, *J* = 16.1 Hz, 1H), 3.81 (s, 3H), 3.77 (s, 3H), 3.50 (s, 3H), 2.94 (dd, *J* = 14.8, 4.6 Hz, 1H), 2.72 (dd, *J* = 14.8, 12.8 Hz, 1H), 2.31 (ddd, *J* = 12.8, 4.7, 1.5 Hz, 1H), 1.29 (s, 3H). **^{13}C-NMR:** (176 MHz, $CDCl_3$) δ 171.80, 169.06, 158.90, 154.43, 137.90, 133.74, 131.61, 130.04, 130.02,

129.76, 128.64, 127.58, 127.36, 126.66, 118.09, 113.74, 113.37, 109.57, 80.10, 65.12, 57.15, 55.70, 55.42, 55.41, 55.36, 53.00, 52.90, 52.71, 52.56, 52.40, 52.32, 37.23, 36.82, 32.37, 29.85, 27.92, 19.43.

5. ^{1}H NMR, ^{13}C NMR, FT-IR and Mass spectra.

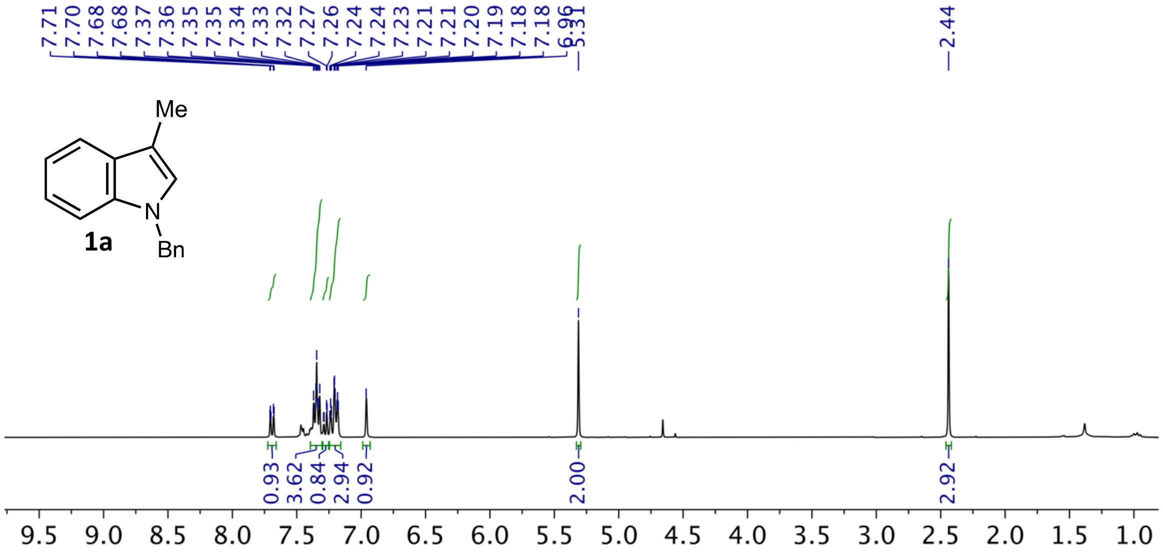

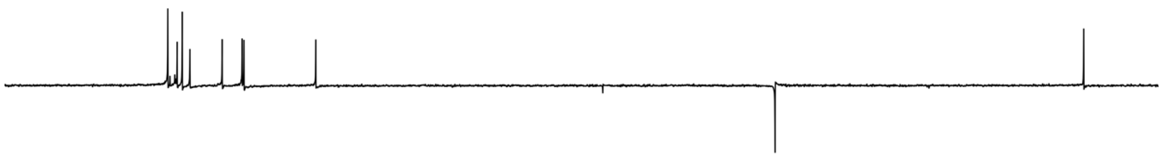

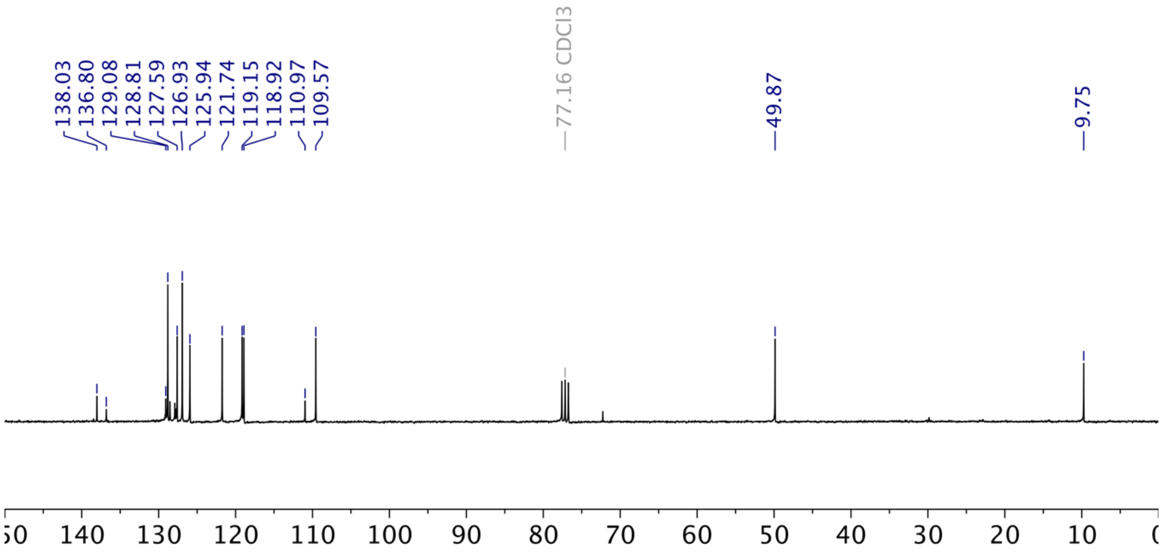

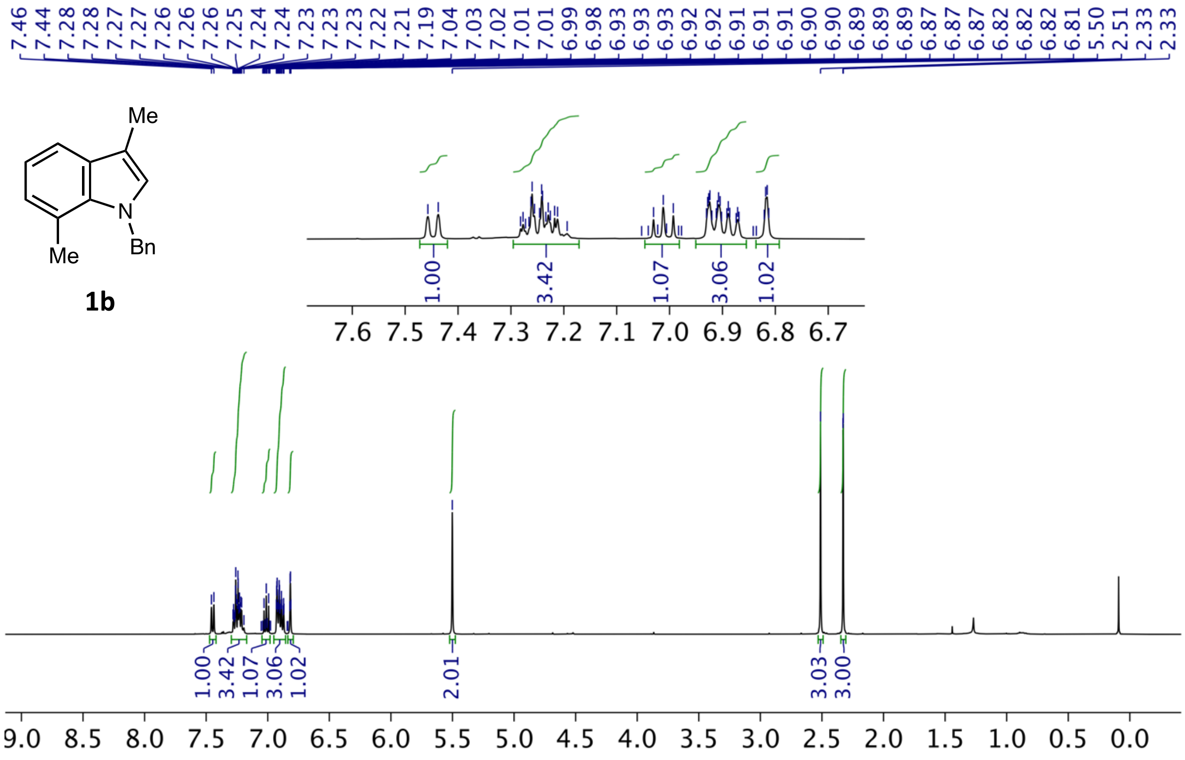
Me
N
Me
Bn
7.46
7.44
7.28
7.28
7.27
7.27
7.26
7.26
7.26
7.25
7.24
7.24
7.23
7.23
7.23
7.22
7.21
7.19
7.04
7.03
7.02
7.01
7.01
6.99
6.98
6.93
6.93
6.92
6.92
6.91
6.91
6.91
6.90
6.90
6.89
6.89
6.87
6.87
6.87
6.82
6.82
6.82
6.81
5.50
2.51
2.33
2.33
1.00
3.42
1.07
3.06
1.02
7.6 7.5 7.4 7.3 7.2 7.1 7.0 6.9 6.8 6.7
1.00
3.42
1.07
3.06
1.02
2.01
3.03
3.00
9.0 8.5 8.0 7.5 7.0 6.5 6.0 5.5 5.0 4.5 4.0 3.5 3.0 2.5 2.0 1.5 1.0 0.5 0.0

1b

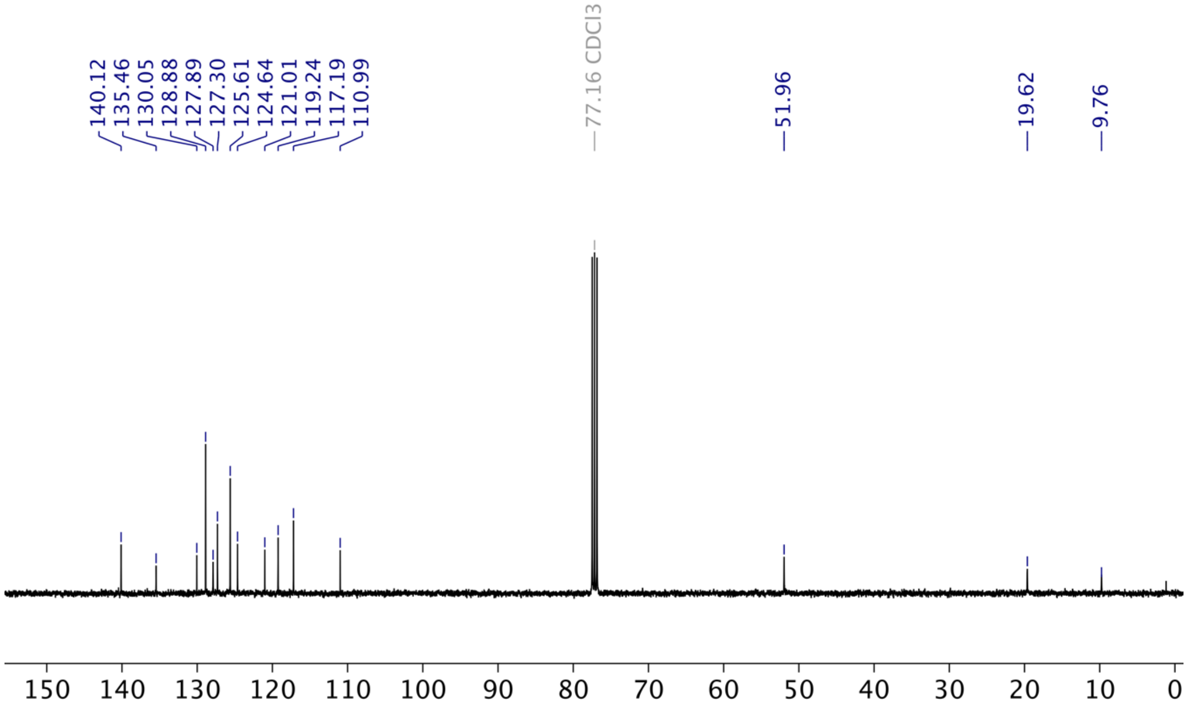
140.12
135.46
130.05
128.88
127.89
127.30
125.61
124.64
121.01
119.24
117.19
110.99
77.16 CDCl3
51.96
19.62
9.76
150 140 130 120 110 100 90 80 70 60 50 40 30 20 10 0

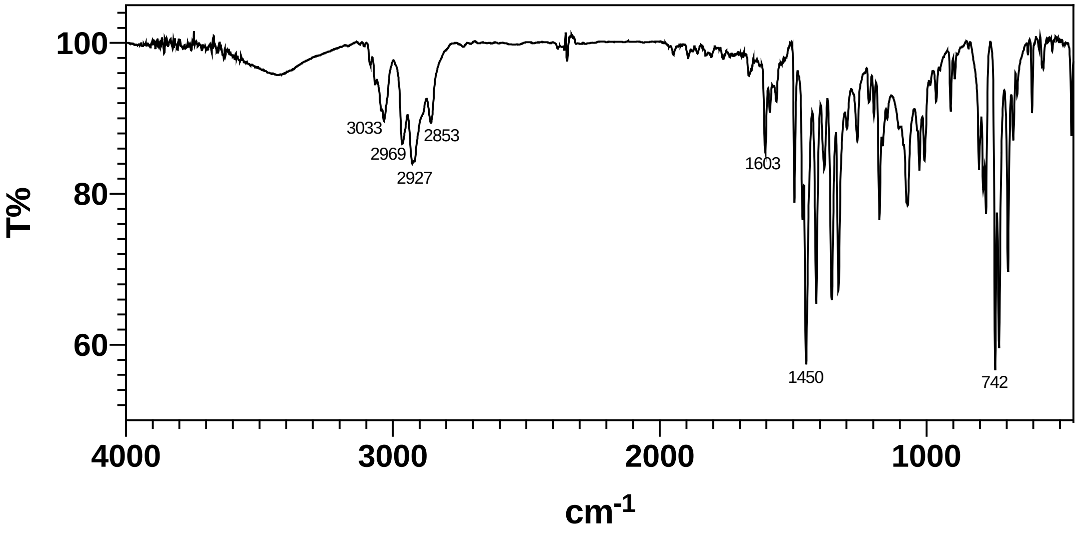

Acquisition Parameter

				Set Corrector Fill	74 V
Source Type	ESI	Ion Polarity	Positive	Set Pulsar Pull	799 V
n/a	n/a	Set Capillary Exit	80.0 V	Set Pulsar Push	799 V
Scan Begin	50 m/z	Set Hexapole RF	60.0 V	Set Reflector	1700 V
Scan End	3000 m/z	Set Skimmer 1	50.0 V	Set Flight Tube	8600 V
		Set Hexapole 1	24.3 V	Set Detector TOF	2311 V

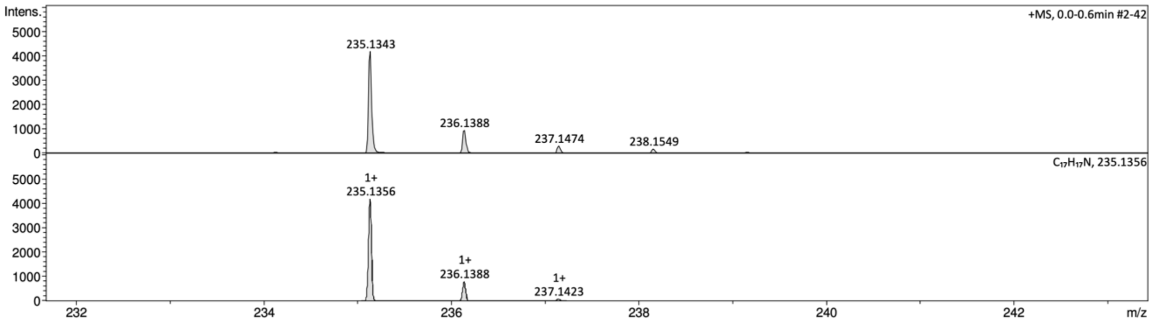

Meas. m/z	#	Ion Formula	m/z	err [ppm]	mSigma	# mSigma	Score	rdb	e⁻ Conf	N-Rule
235.1343	1	C17H17N	235.1356	5.2	37.1	1	100.00	10.0	odd	ok

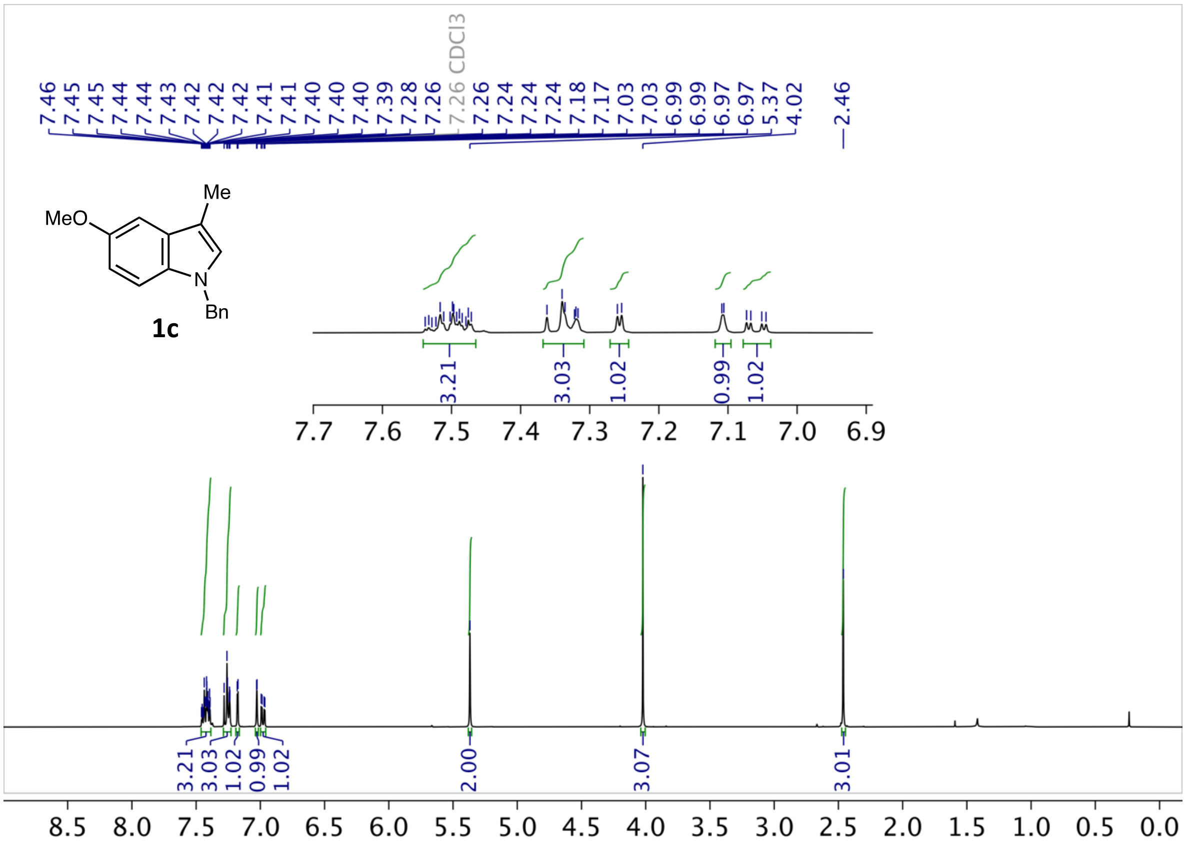
7.46
7.45
7.45
7.44
7.44
7.43
7.42
7.42
7.42
7.41
7.41
7.40
7.40
7.40
7.39
7.28
7.26
7.26 CDCl3
7.26
7.24
7.24
7.24
7.18
7.17
7.03
7.03
6.99
6.99
6.97
6.97
5.37
4.02
2.46
Me
MeO
N
Bn
3.21
3.03
1.02
0.99
1.02
2.00
3.07
3.01

1c

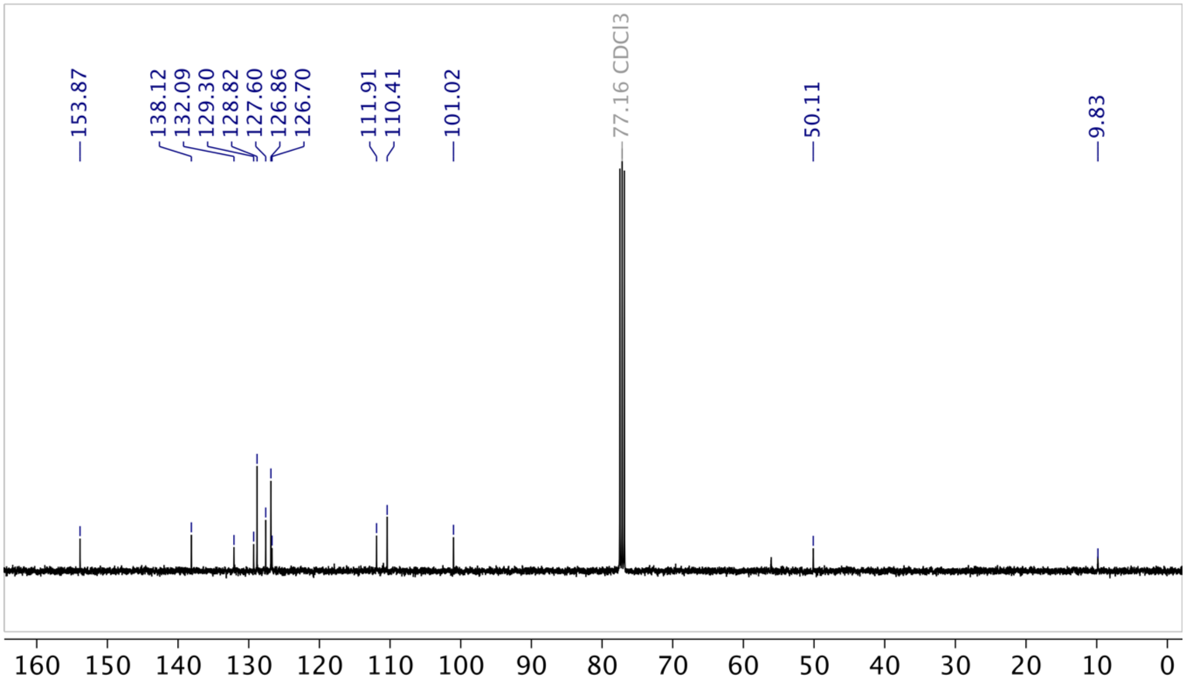
153.87
138.12
132.09
129.30
128.82
127.60
126.86
126.70
111.91
110.41
101.02
77.16 CDCl3
50.11
9.83

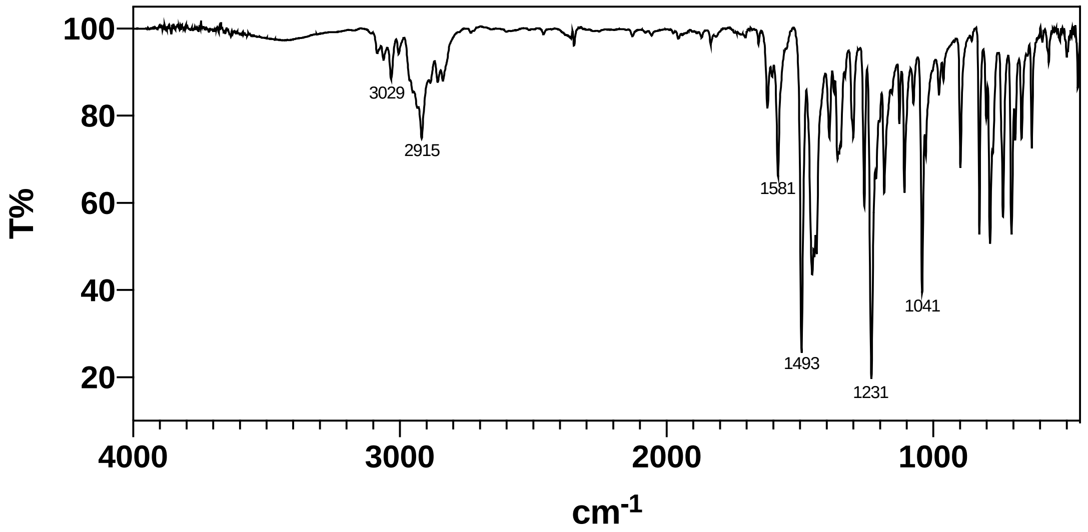

Acquisition Parameter

				Set Corrector Fill	74 V
Source Type	ESI	Ion Polarity	Positive	Set Pulsar Pull	799 V
n/a	n/a	Set Capillary Exit	80.0 V	Set Pulsar Push	799 V
Scan Begin	50 m/z	Set Hexapole RF	60.0 V	Set Reflector	1700 V
Scan End	3000 m/z	Set Skimmer 1	50.0 V	Set Flight Tube	8600 V
		Set Hexapole 1	24.3 V	Set Detector TOF	2311 V

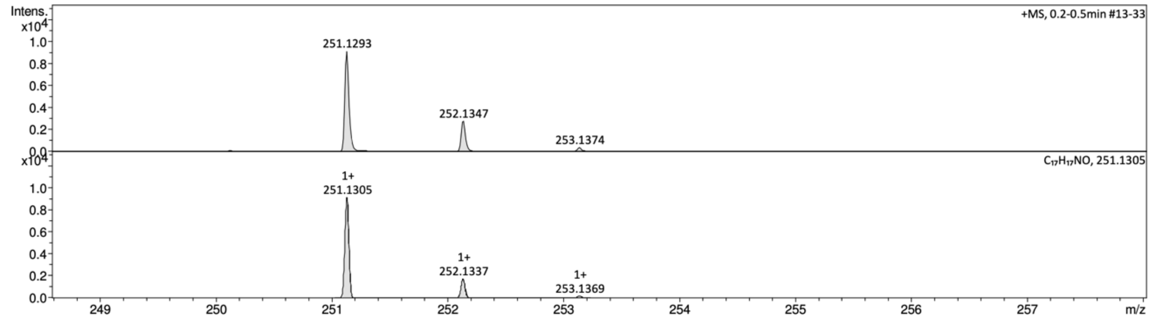

Meas. m/z	#	Ion Formula	m/z	err [ppm]	mSigma	# mSigma	Score	rdb	e⁻ Conf	N-Rule
251.1293	1	C17H17NO	251.1305	4.7	64.8	1	100.00	10.0	odd	ok

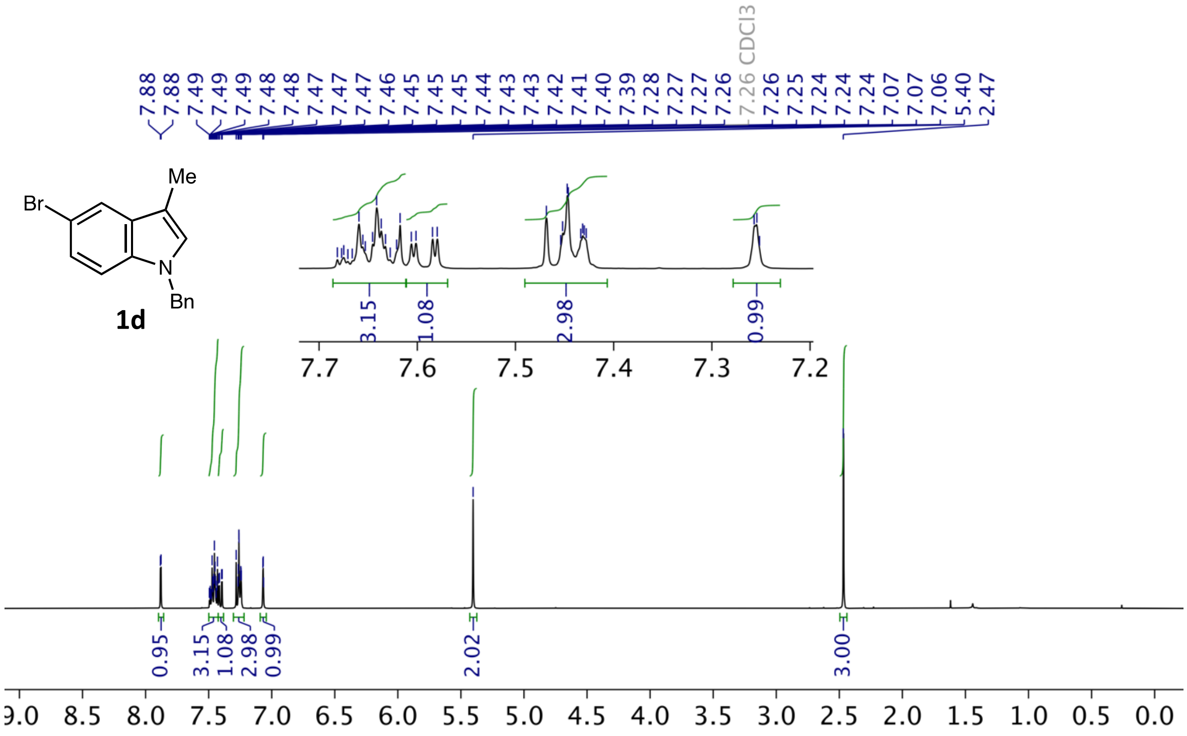
Br
Me
N
Bn
7.88 7.88 7.49 7.49 7.49 7.48 7.48 7.47 7.47 7.47 7.46 7.45 7.45 7.45 7.44 7.43 7.43 7.42 7.41 7.40 7.39 7.28 7.27 7.27 7.26 7.26 CDCl3 7.26 7.25 7.24 7.24 7.24 7.07 7.07 7.06 5.40 2.47
3.15 1.08 2.98 0.99
7.7 7.6 7.5 7.4 7.3 7.2
0.95 3.15 1.08 2.98 0.99 2.02 3.00
9.0 8.5 8.0 7.5 7.0 6.5 6.0 5.5 5.0 4.5 4.0 3.5 3.0 2.5 2.0 1.5 1.0 0.5 0.0

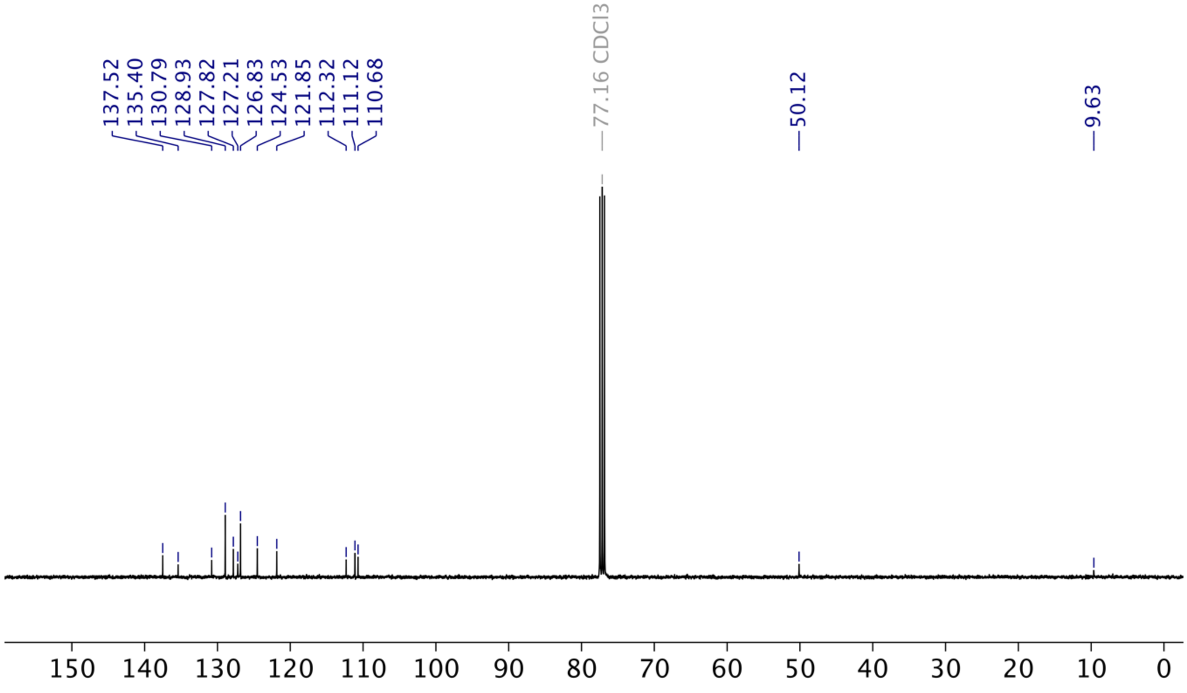
137.52 135.40 130.79 128.93 127.82 127.21 126.83 124.53 121.85 112.32 111.12 110.68
77.16 CDCl3
50.12
9.63
150 140 130 120 110 100 90 80 70 60 50 40 30 20 10 0

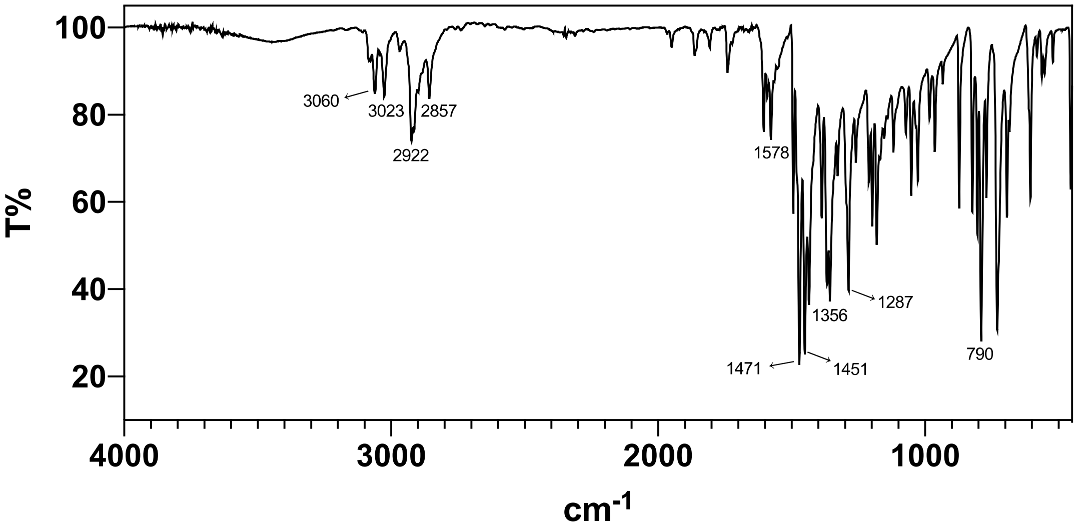

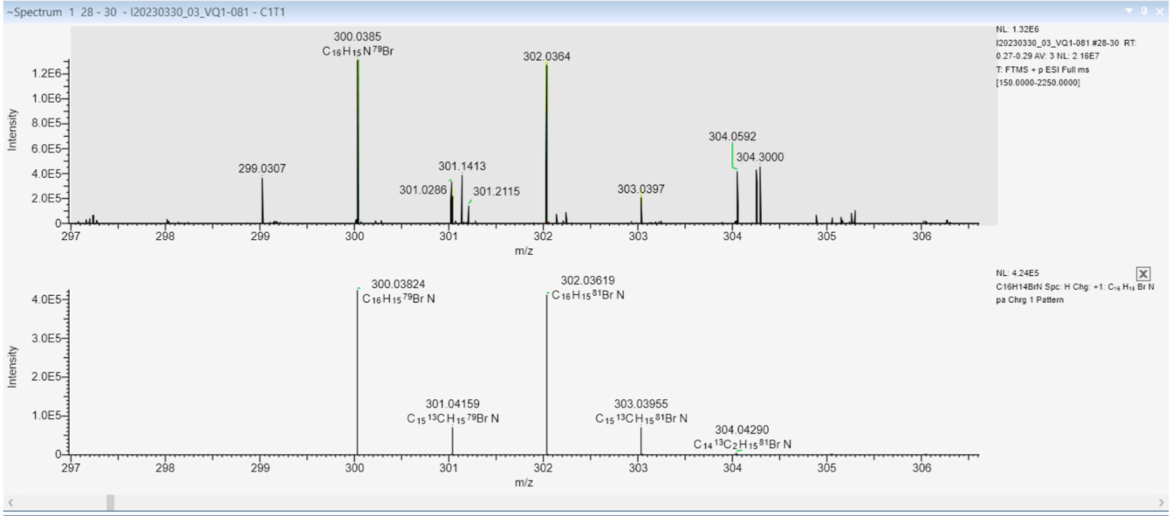

Elemental Composition Results - Spectrum 1

Peak Mass	Display Formula	S Fit	RDB	Delta [ppm]	Theo. mass	Rank	Combined Score	# Matched Iso.	# Missed Iso.	MS Cov. [%]	Pattern Cov. [%]
300.0385	$C_{16}H_{15}N^{79}Br$	36.58	9.50	0.82	300.03824	1	66.00	6	2	67.63	99.09

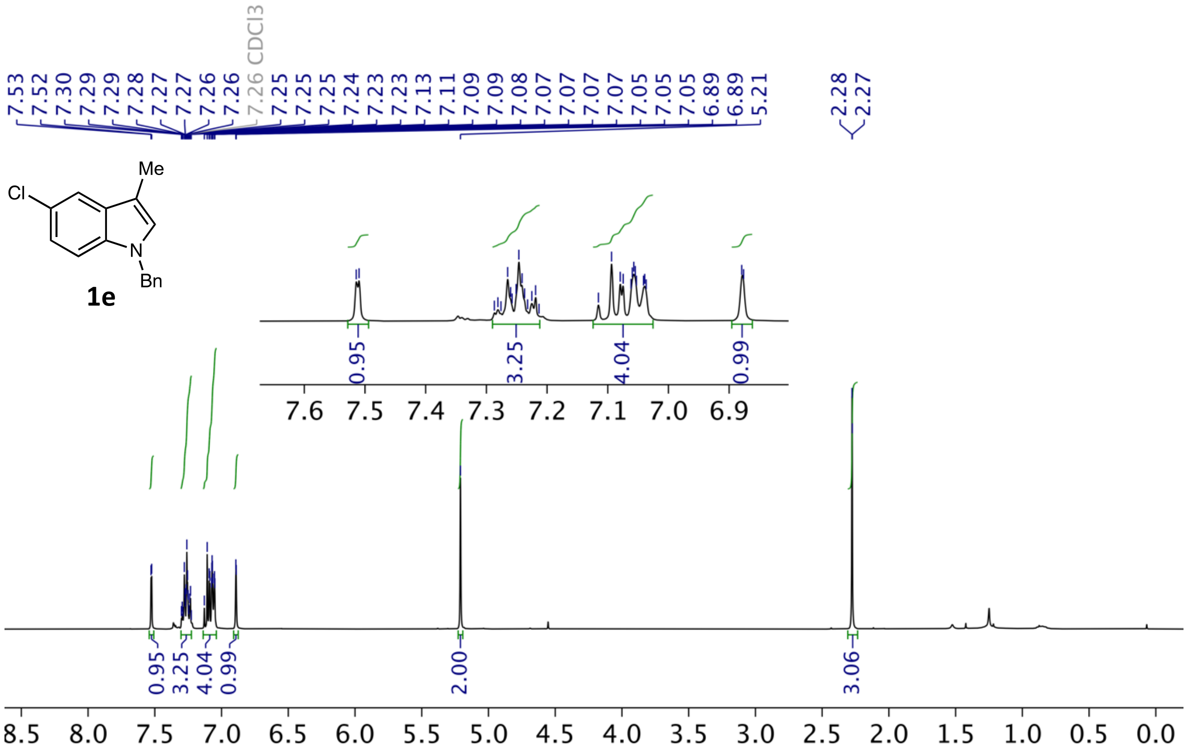
7.53 7.52 7.30 7.29 7.29 7.28 7.27 7.27 7.26 7.26 7.26 CDCl3 7.25 7.25 7.25 7.24 7.23 7.23 7.13 7.11 7.09 7.09 7.08 7.07 7.07 7.07 7.07 7.05 7.05 7.05 6.89 6.89 5.21
2.28 2.27
Me
Cl
N
Bn
1e
0.95 3.25 4.04 0.99
7.6 7.5 7.4 7.3 7.2 7.1 7.0 6.9
0.95 3.25 4.04 0.99 2.00 3.06
8.5 8.0 7.5 7.0 6.5 6.0 5.5 5.0 4.5 4.0 3.5 3.0 2.5 2.0 1.5 1.0 0.5 0.0

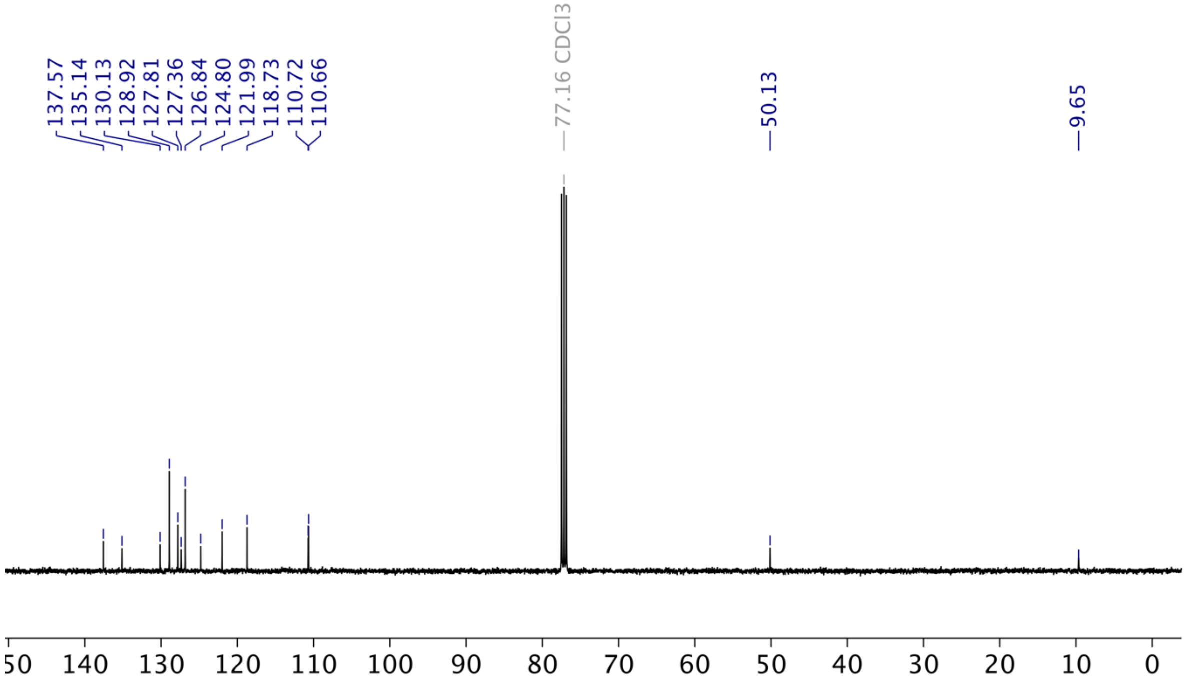
137.57 135.14 130.13 128.92 127.81 127.36 126.84 124.80 121.99 118.73 110.72 110.66
77.16 CDCl3
50.13
9.65
50 140 130 120 110 100 90 80 70 60 50 40 30 20 10 0

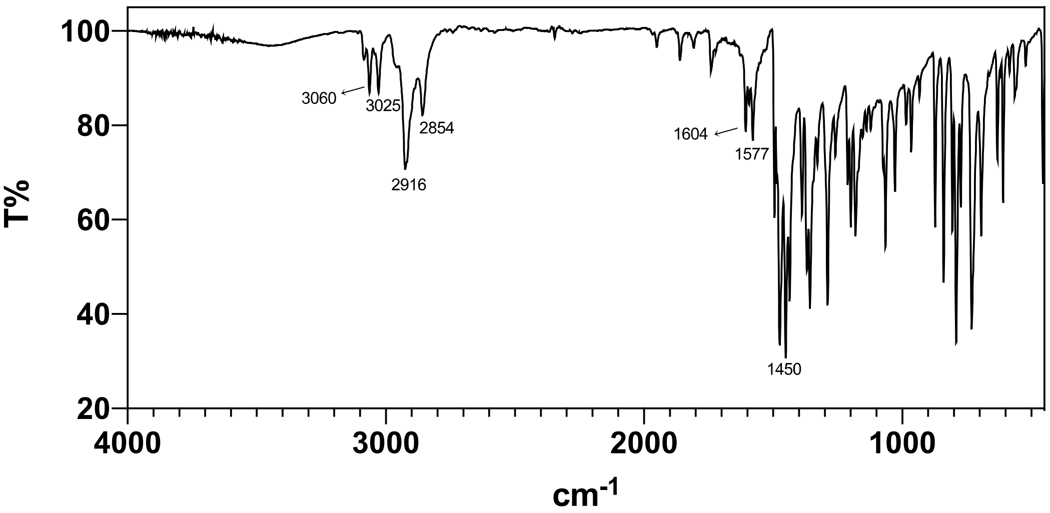

Acquisition Parameter

Source Type	ESI	Ion Polarity	Positive	Set Corrector Fill	74 V
n/a	n/a	Set Capillary Exit	80.0 V	Set Pulsar Pull	799 V
Scan Begin	50 m/z	Set Hexapole RF	60.0 V	Set Pulsar Push	799 V
Scan End	3000 m/z	Set Skimmer 1	50.0 V	Set Reflector	1700 V
		Set Hexapole 1	24.3 V	Set Flight Tube	8600 V
				Set Detector TOF	2311 V

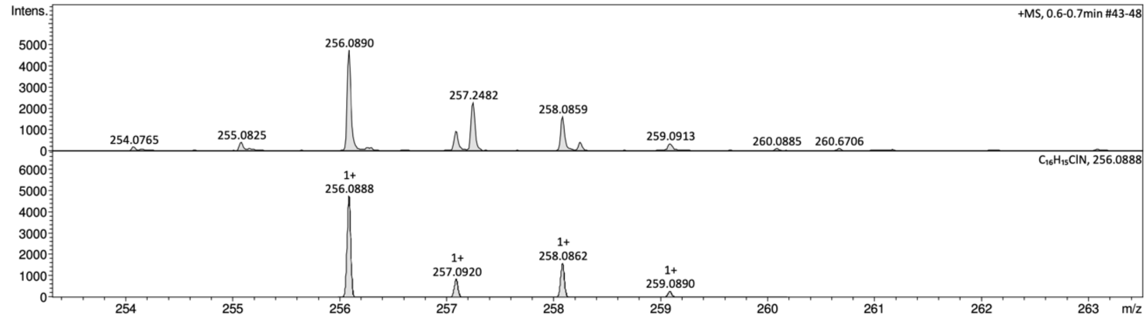

Meas. m/z	#	Ion Formula	m/z	err [ppm]	mSigma	# mSigma	Score	rdb	e⁻ Conf	N-Rule
256.0890	1	C16H15ClN	256.0888	-1.0	11.7	1	100.00	9.5	even	ok

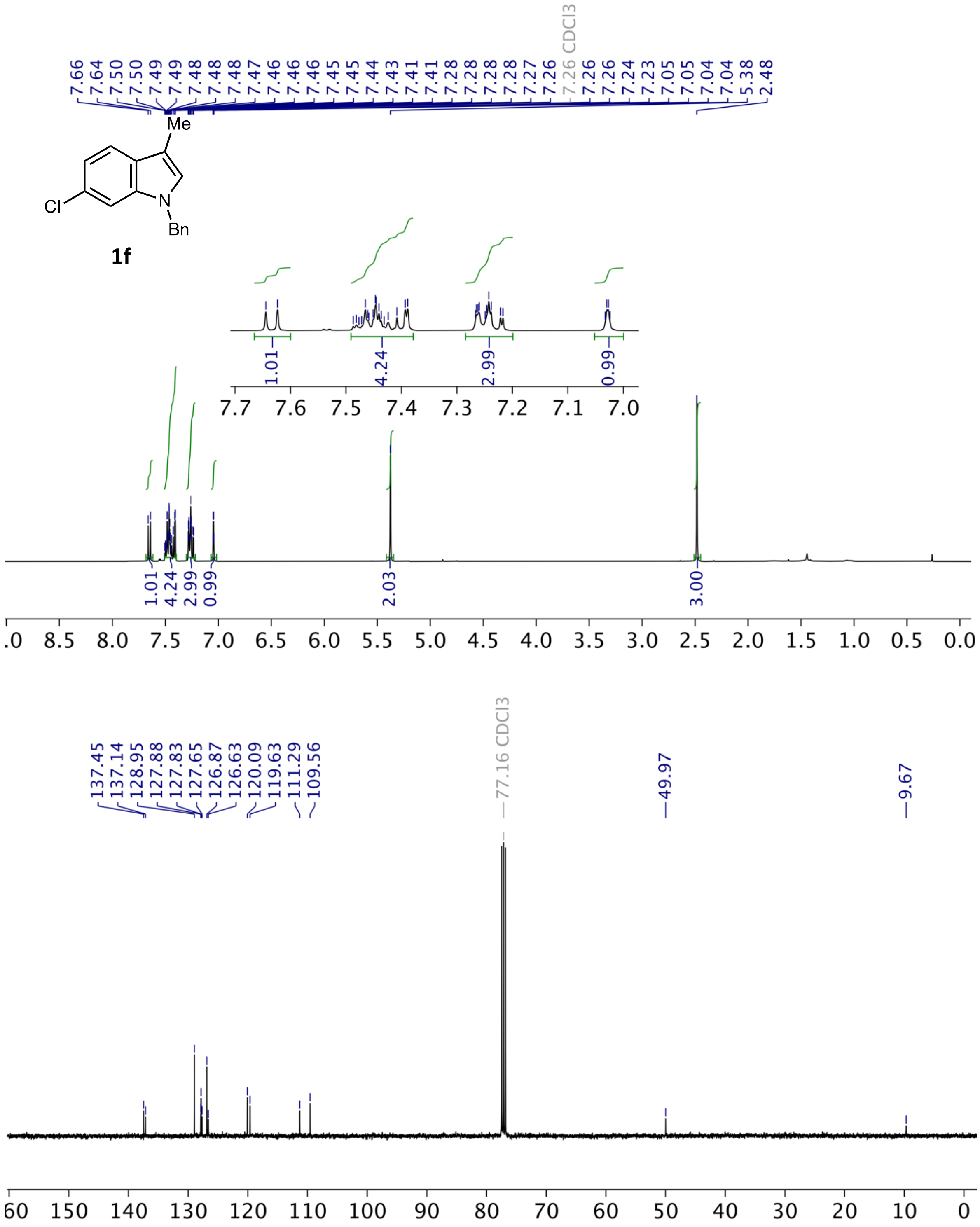
Me
Cl
N
Bn
1f

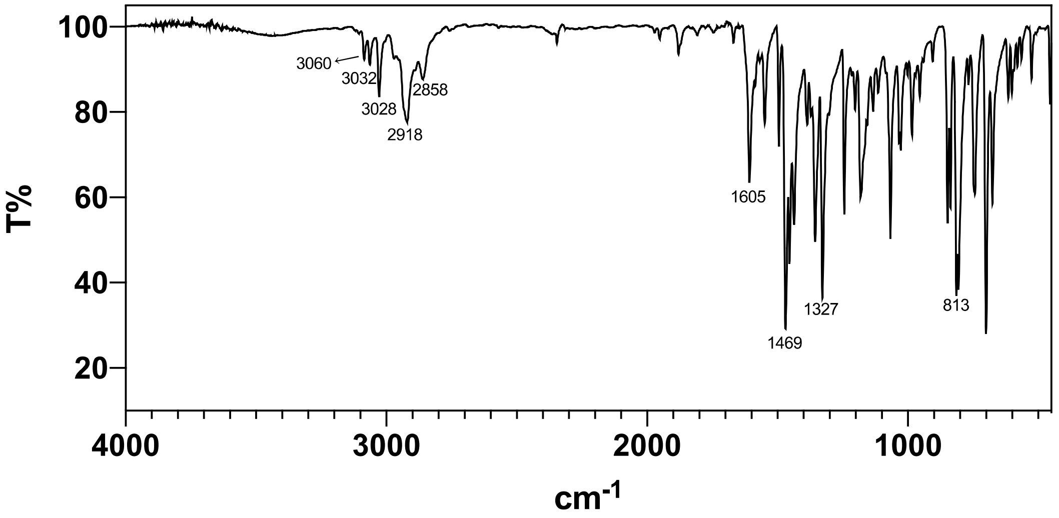

Acquisition Parameter

Source Type	ESI	Capillary	4500 V	Nebulizer	0.4 Bar	Corona	172 nA
Ion Polarity	Positive	Set Capillary Exit	80.0 V	Dry Gas	4.0 l/min	Set Hexapole RF	60.0 V
n/a	n/a	Set Skimmer 1	50.0 V	Dry Heater	180 °C	APCI Heater	514 °C

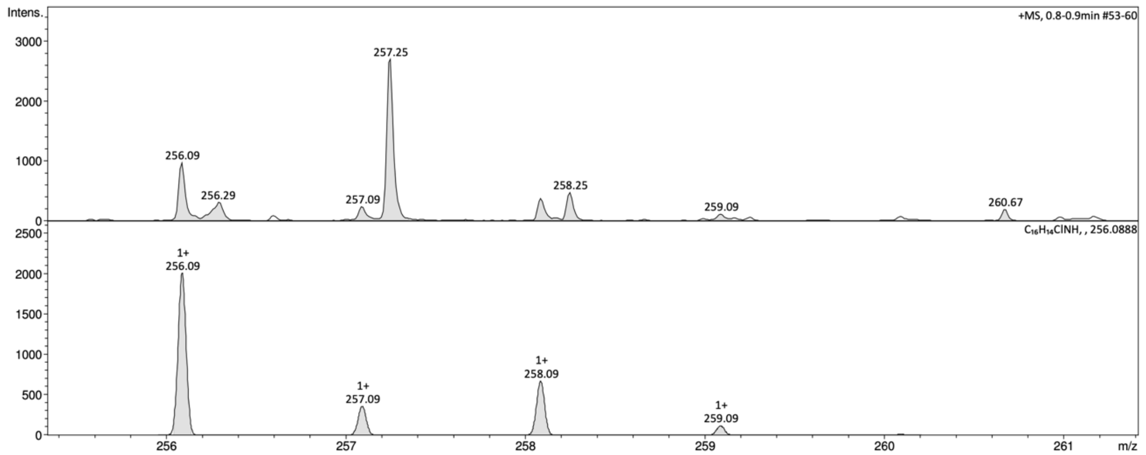

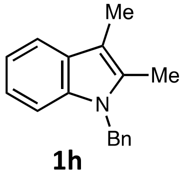

1h

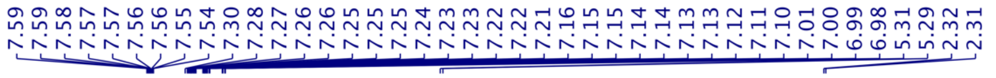

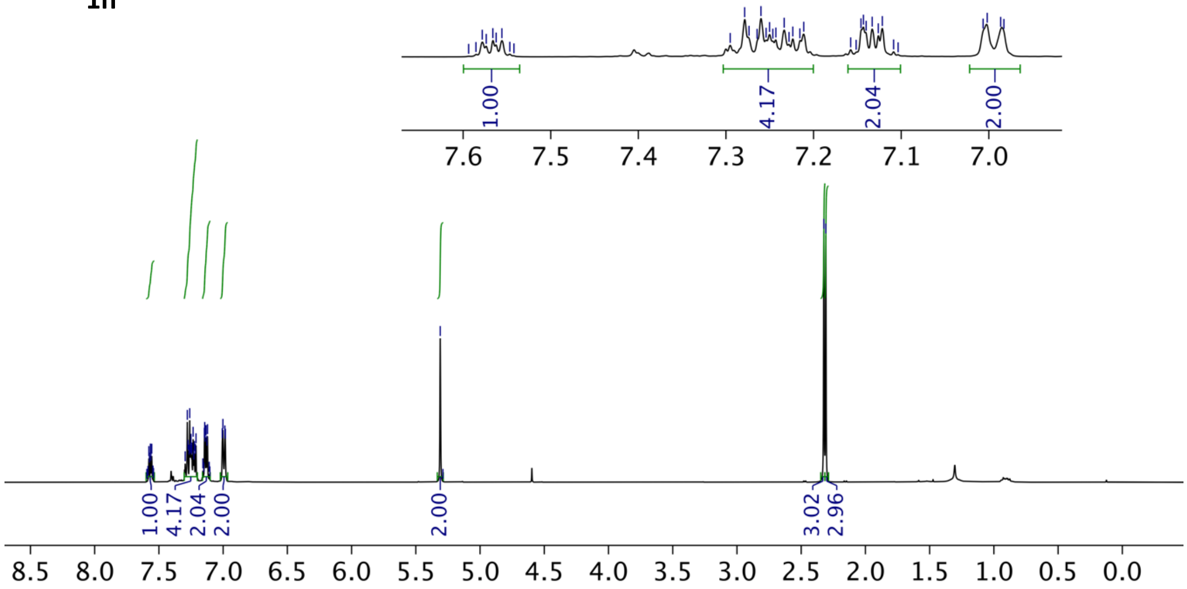

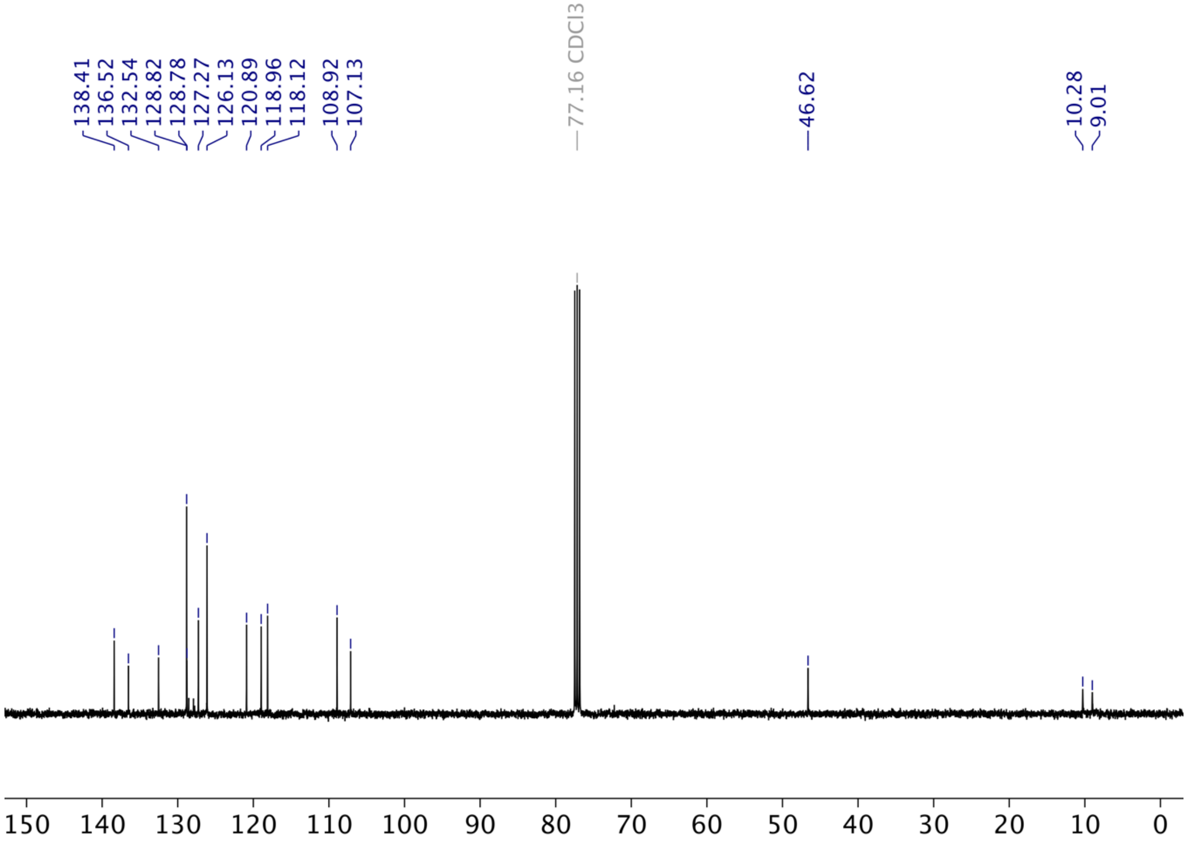

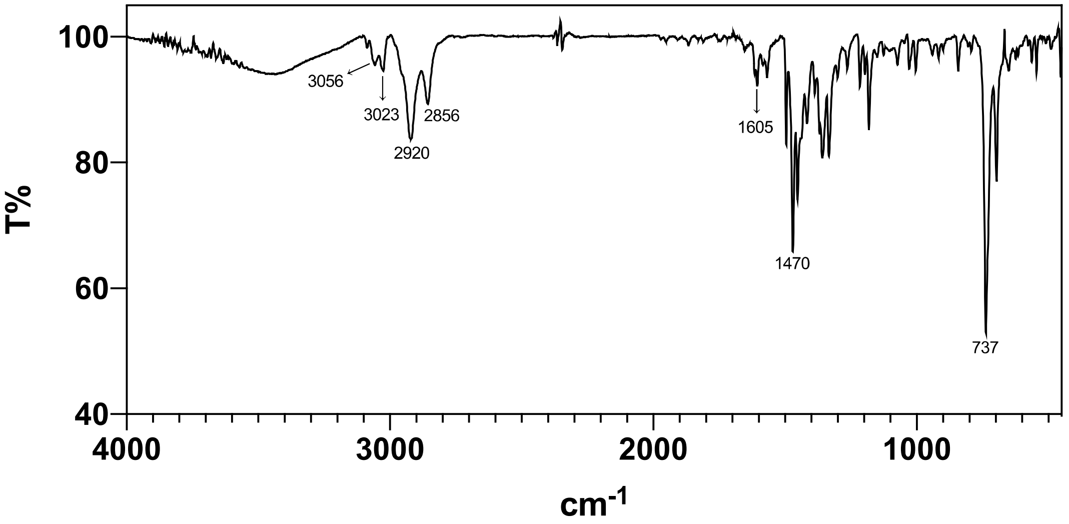

Acquisition Parameter

				Set Corrector Fill	74 V
Source Type	ESI	Ion Polarity	Positive	Set Pulsar Pull	799 V
n/a	n/a	Set Capillary Exit	80.0 V	Set Pulsar Push	799 V
Scan Begin	50 m/z	Set Hexapole RF	60.0 V	Set Reflector	1700 V
Scan End	3000 m/z	Set Skimmer 1	50.0 V	Set Flight Tube	8600 V
		Set Hexapole 1	24.3 V	Set Detector TOF	2311 V

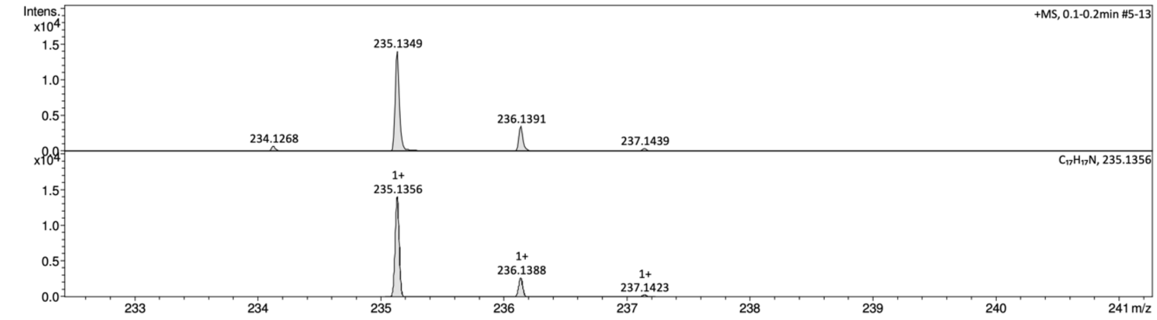

Meas. m/z	#	Ion Formula	m/z	err [ppm]	mSigma	# mSigma	Score	rdb	e⁻ Conf	N-Rule
235.1349	1	C17H17N	235.1356	2.9	33.9	1	100.00	10.0	odd	ok

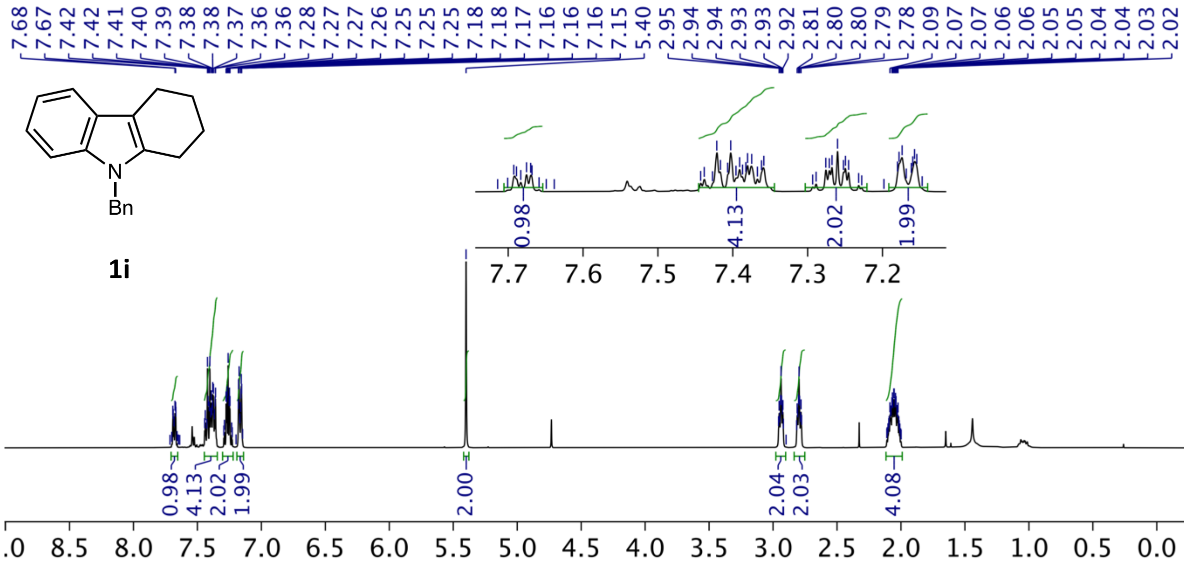

7.68 7.67 7.42 7.42 7.41 7.40 7.39 7.38 7.38 7.37 7.36 7.28 7.27 7.27 7.26 7.25 7.25 7.25 7.18 7.18 7.17 7.16 7.16 7.16 7.15 5.40 2.95 2.94 2.93 2.93 2.92 2.81 2.80 2.80 2.79 2.78 2.09 2.07 2.07 2.06 2.06 2.05 2.05 2.04 2.04 2.03 2.02
N
Bn
1i
0.98 4.13 2.02 1.99
7.7 7.6 7.5 7.4 7.3 7.2
0.98 4.13 2.02 1.99 2.00 2.04 2.03 4.08
.0 8.5 8.0 7.5 7.0 6.5 6.0 5.5 5.0 4.5 4.0 3.5 3.0 2.5 2.0 1.5 1.0 0.5 0.0

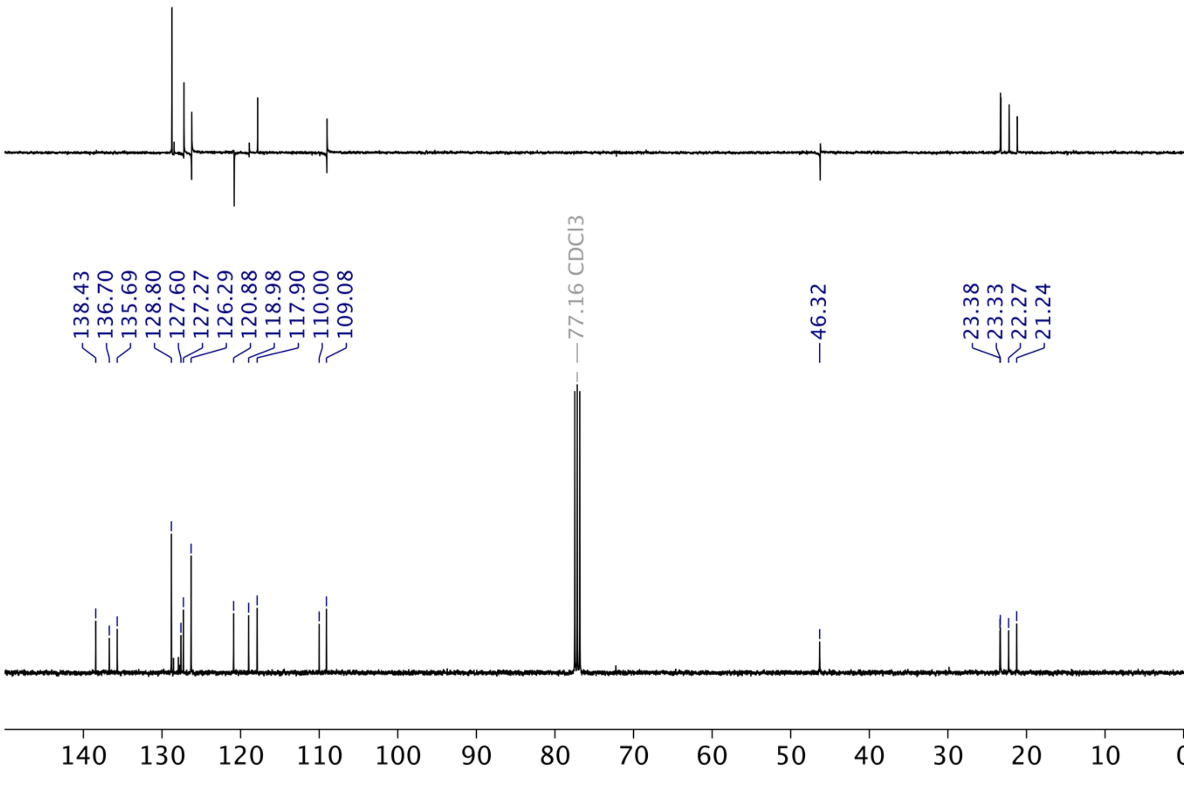

138.43 136.70 135.69 128.80 127.60 127.27 126.29 120.88 118.98 117.90 110.00 109.08
77.16 CDCl3
46.32
23.38 23.33 22.27 21.24
140 130 120 110 100 90 80 70 60 50 40 30 20 10

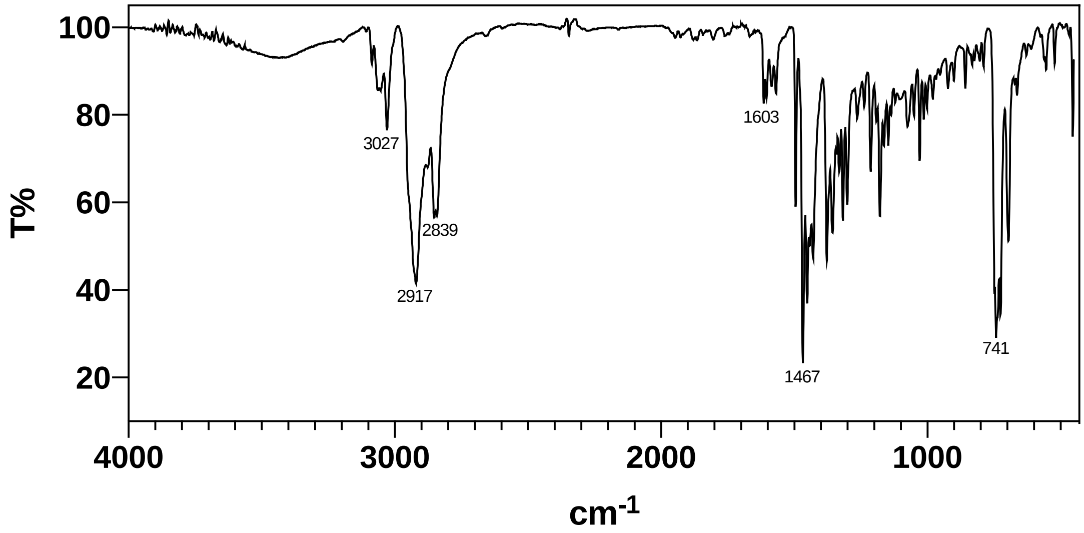

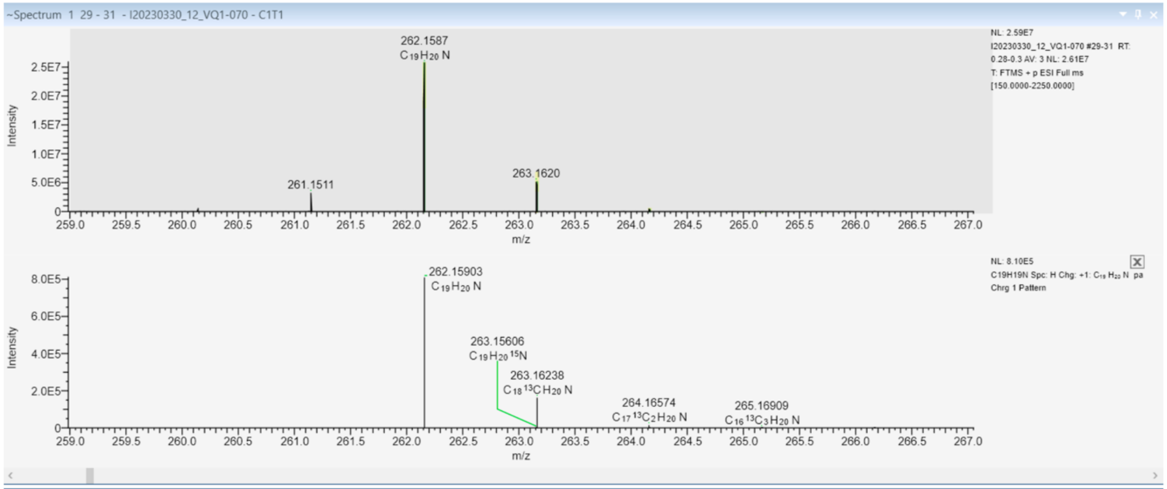

Elemental Composition Results - Spectrum 1

Peak Mass	Display Formula	S Fit	RDB	Delta [ppm]	Theo. mass	Rank	Combined Score	# Matched Iso.	# Missed Iso.	MS Cov. [%]	Pattern Cov. [%]
262.1587	$C_{19}H_{20}N$	53.37	10.50	-1.21	262.15903	1	96.36	4	1	98.75	99.70

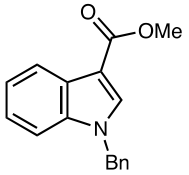
O
OMe
N
Bn

1j

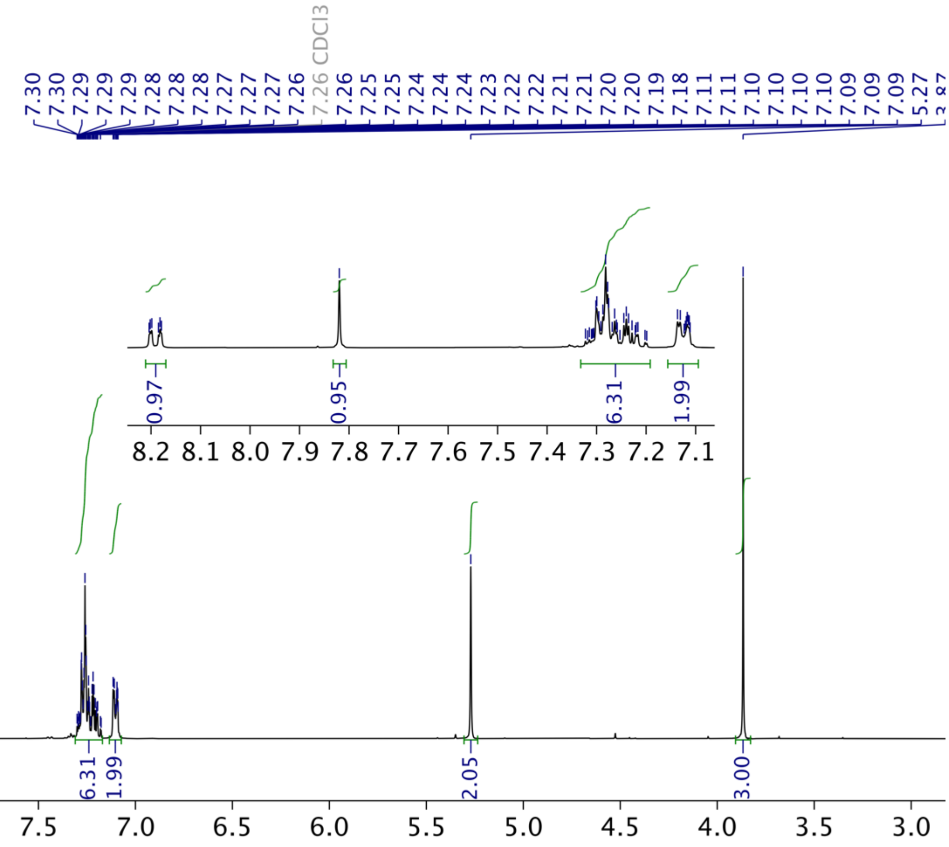
8.18
8.18
8.18
8.16
8.16
8.16
7.80
7.26 CDCl3
5.27
0.97
0.95
6.31
1.99
2.05
3.00
8.2 8.1 8.0 7.9 7.8 7.7 7.6 7.5 7.4 7.3 7.2 7.1
8.5 8.0 7.5 7.0 6.5 6.0 5.5 5.0 4.5 4.0 3.5 3.0

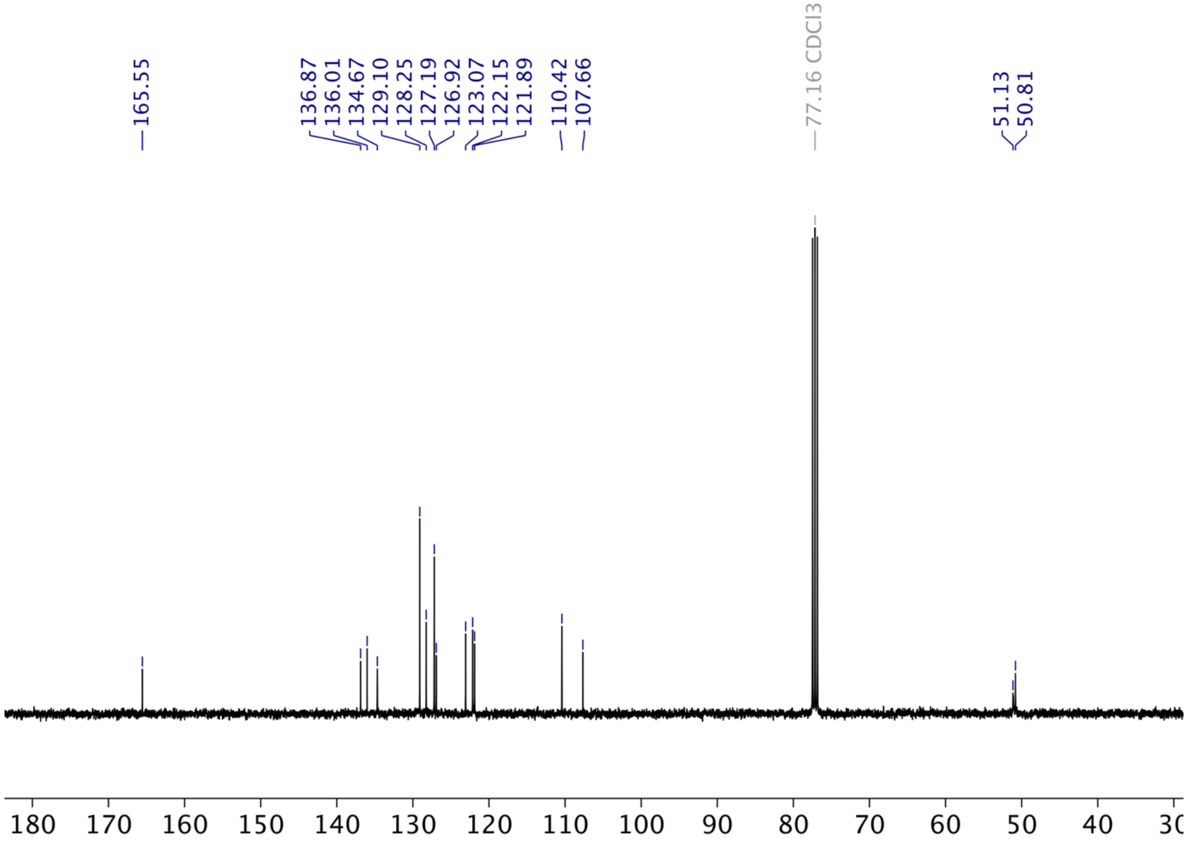
165.55
136.87
136.01
134.67
129.10
128.25
127.19
126.92
123.07
122.15
121.89
110.42
107.66
77.16 CDCl3
51.13
50.81
180 170 160 150 140 130 120 110 100 90 80 70 60 50 40

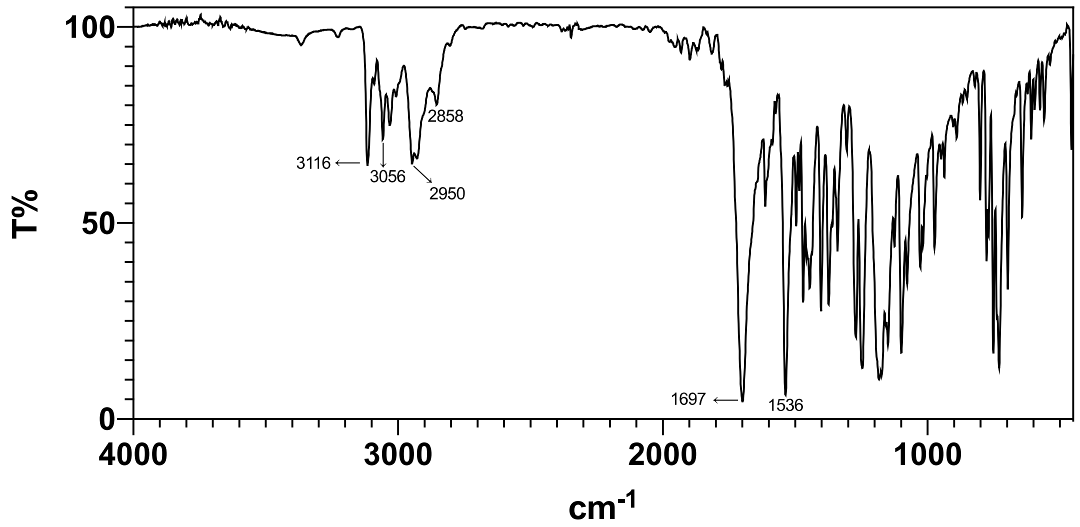

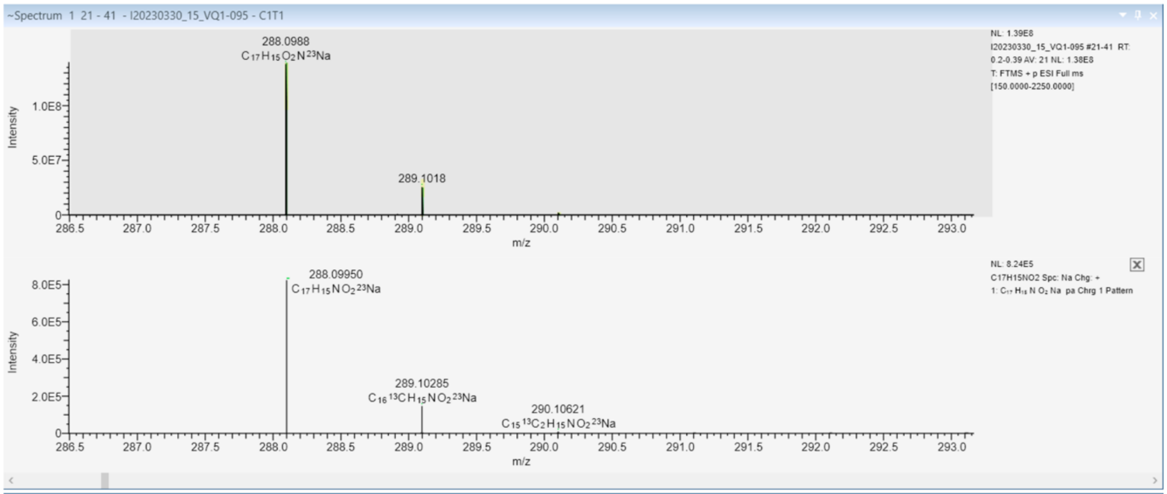

Elemental Composition Results - Spectrum 1

Peak Mass	Display Formula	S Fit	RDB	Delta [ppm]	Theo. mass	Rank	Combined Score	# Matched Iso.	# Missed Iso.	MS Cov. [%]	Pattern Cov. [%]
288.0988	$C_{17}H_{15}O_2N^{23}Na$	44.94	10.50	-2.41	288.09950	1	96.52	4	0	99.39	99.69

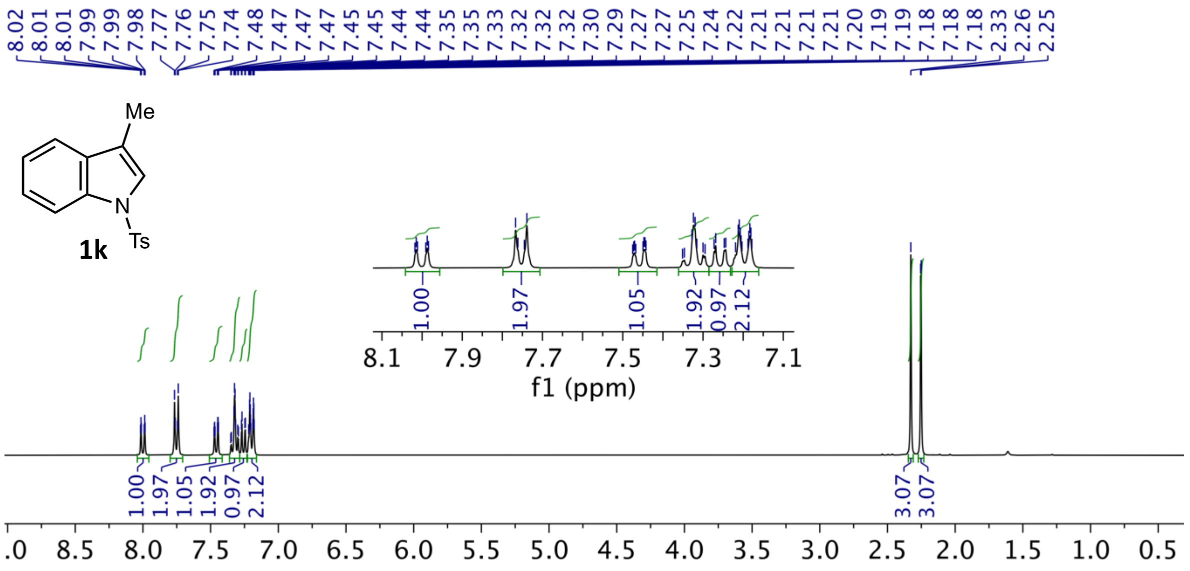
8.02 8.01 8.01 7.99 7.99 7.98 7.77 7.76 7.75 7.74 7.48 7.47 7.47 7.47 7.45 7.45 7.44 7.44 7.35 7.35 7.33 7.32 7.32 7.32 7.30 7.29 7.27 7.27 7.25 7.24 7.22 7.21 7.21 7.21 7.21 7.20 7.19 7.19 7.18 7.18 7.18 2.33 2.26 2.25
Me
N
Ts
1k
1.00 1.97 1.05 1.92 0.97 2.12
8.1 7.9 7.7 7.5 7.3 7.1
f1 (ppm)
3.07 3.07
.0 8.5 8.0 7.5 7.0 6.5 6.0 5.5 5.0 4.5 4.0 3.5 3.0 2.5 2.0 1.5 1.0 0.5

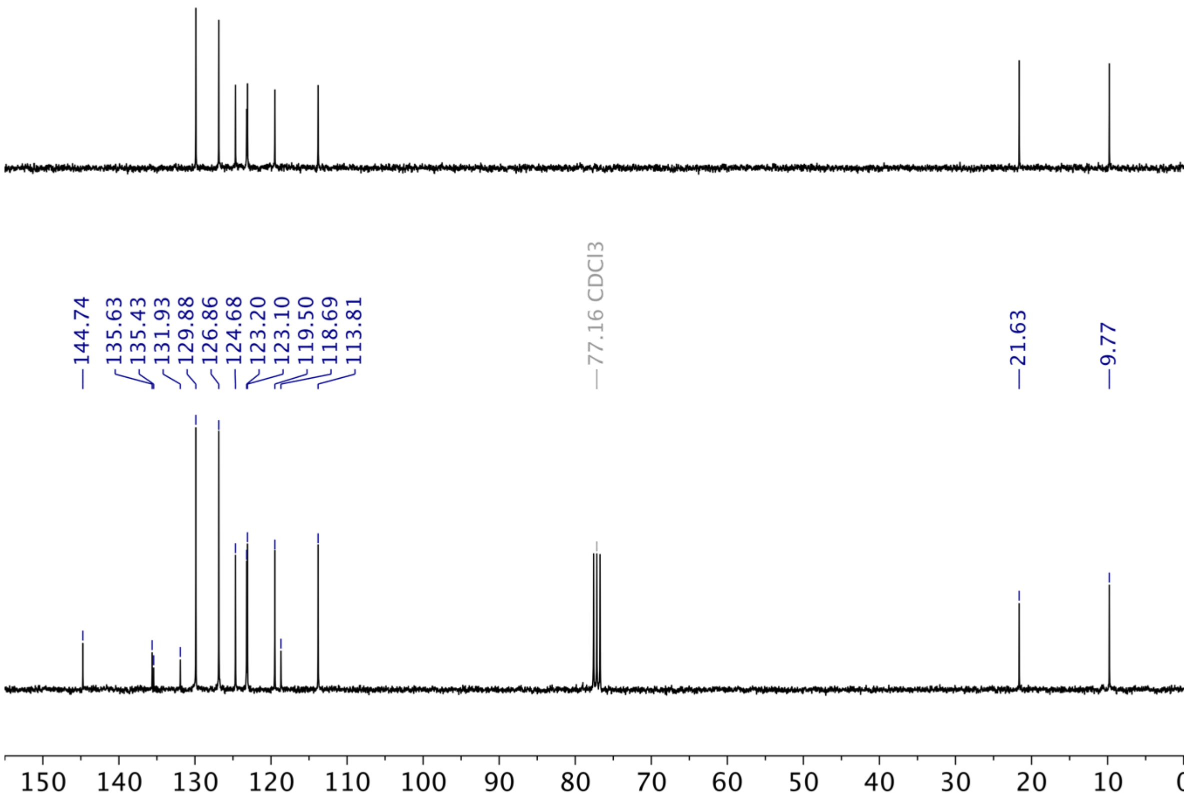
144.74 135.63 135.43 131.93 129.88 126.86 124.68 123.20 123.10 119.50 118.69 113.81
77.16 CDCl3
21.63
9.77
150 140 130 120 110 100 90 80 70 60 50 40 30 20 10 0

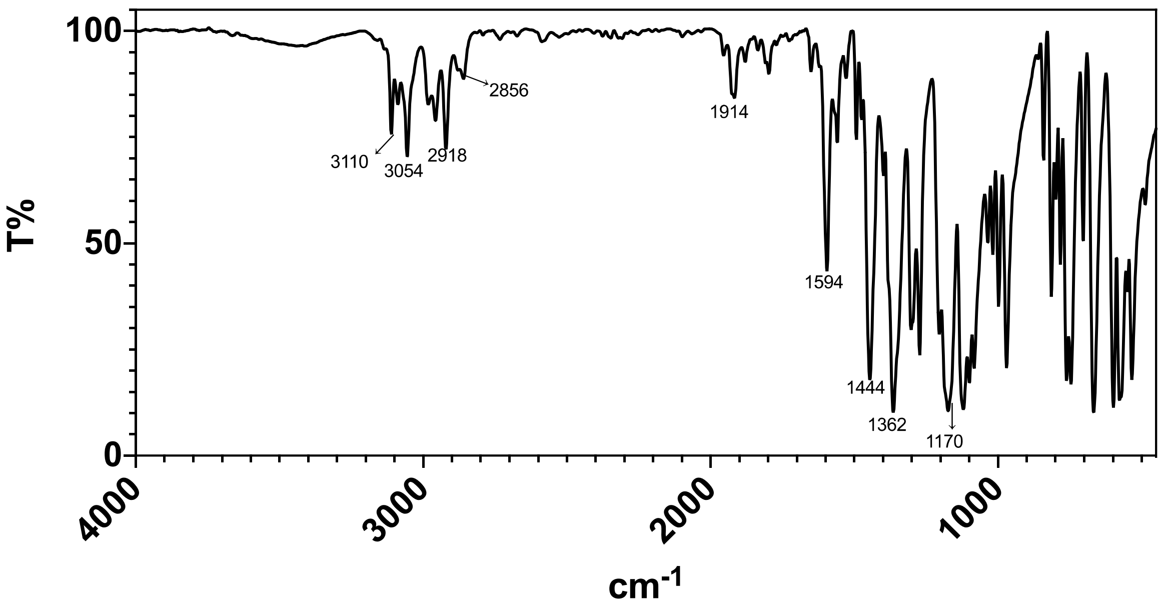
100
50
0
T%
3110
3054
2918
2856
1914
1594
1444
1362
1170
4000
3000
2000
1000
cm-1

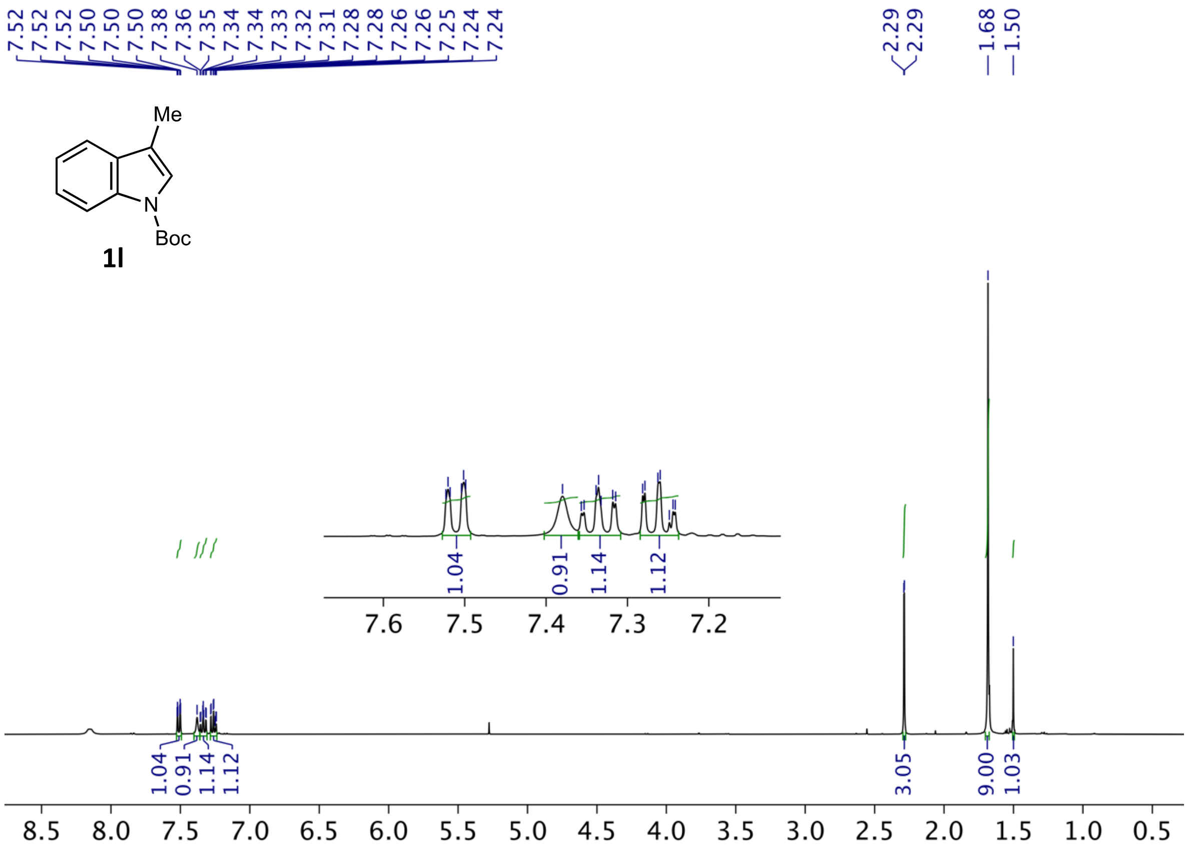
Me
N
Boc
1l

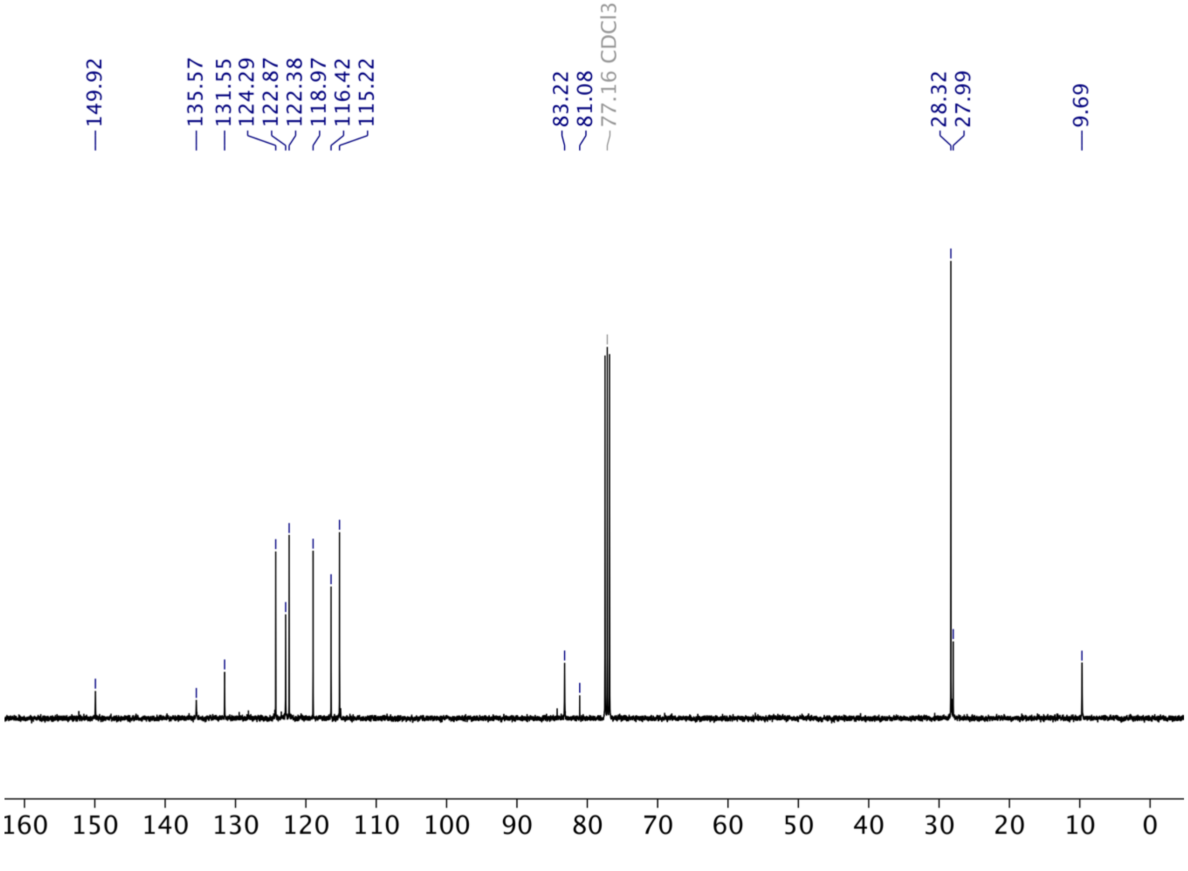

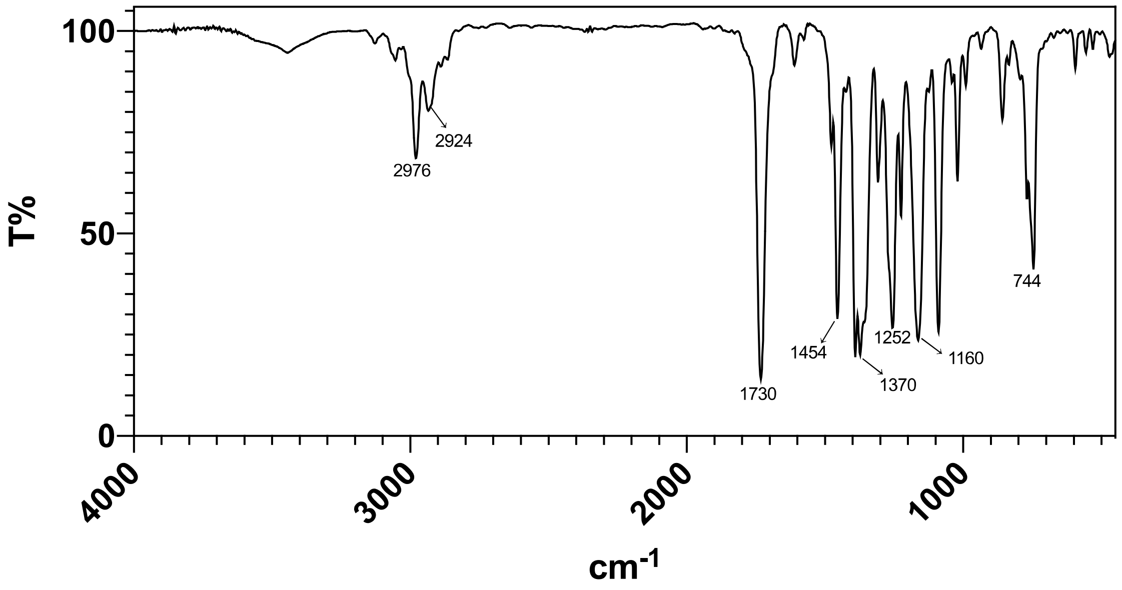
100
50
0
T%
2976
2924
1730
1454
1370
1252
1160
744
4000
3000
2000
1000
cm-1

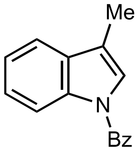
Me
N
Bz

1m

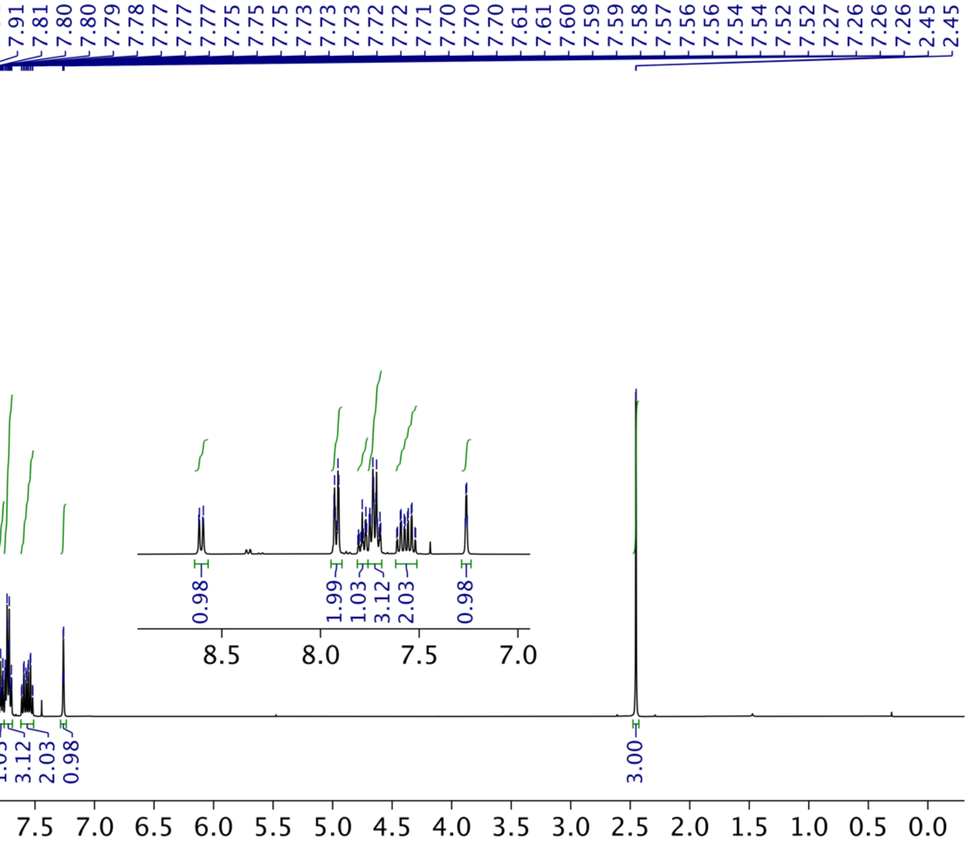
8.62 8.61 8.60 8.59 7.93 7.93 7.92 7.92 7.91 7.91 7.81 7.80 7.80 7.79 7.78 7.77 7.77 7.77 7.75 7.75 7.75 7.73 7.73 7.73 7.72 7.72 7.71 7.70 7.70 7.70 7.61 7.61 7.60 7.59 7.59 7.58 7.57 7.56 7.56 7.54 7.54 7.52 7.52 7.27 7.26 7.26 7.26 2.45 2.45
0.98
1.99
1.03
3.12
2.03
0.98
3.00
8.5
8.0
7.5
7.0
9.5 9.0 8.5 8.0 7.5 7.0 6.5 6.0 5.5 5.0 4.5 4.0 3.5 3.0 2.5 2.0 1.5 1.0 0.5 0.0

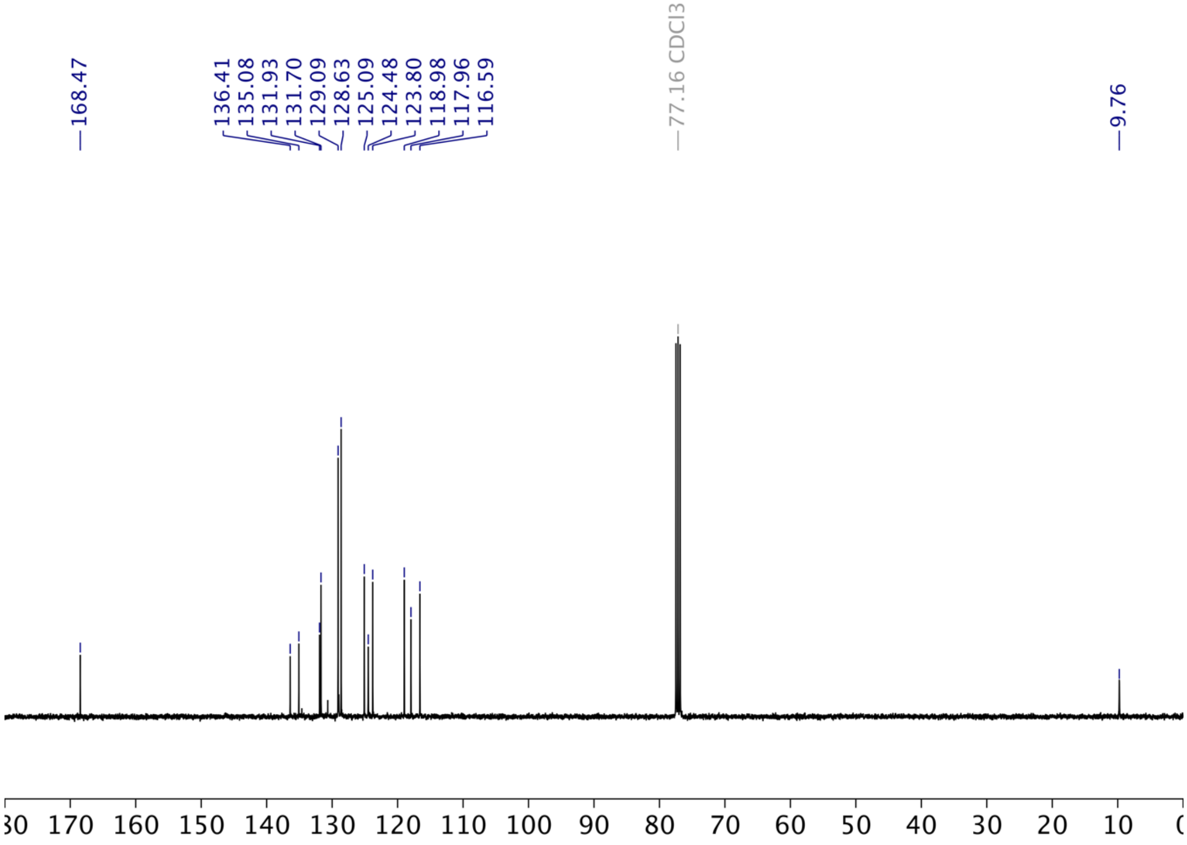
168.47
136.41 135.08 131.93 131.70 129.09 128.63 125.09 124.48 123.80 118.98 117.96 116.59
77.16 CDCl3
9.76
180 170 160 150 140 130 120 110 100 90 80 70 60 50 40 30 20 10 0

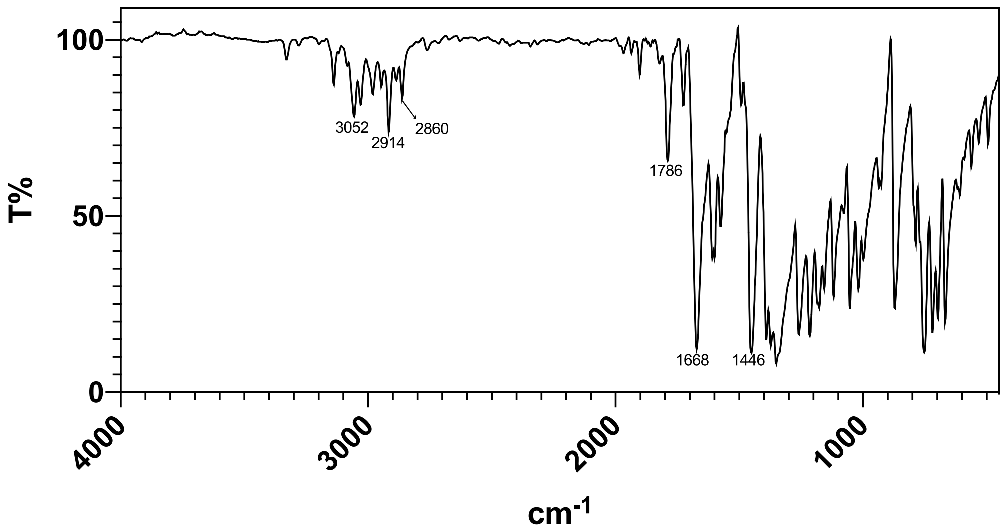
100
50
0
T%
3052
2914
2860
1786
1668
1446
4000
3000
2000
1000
cm-1

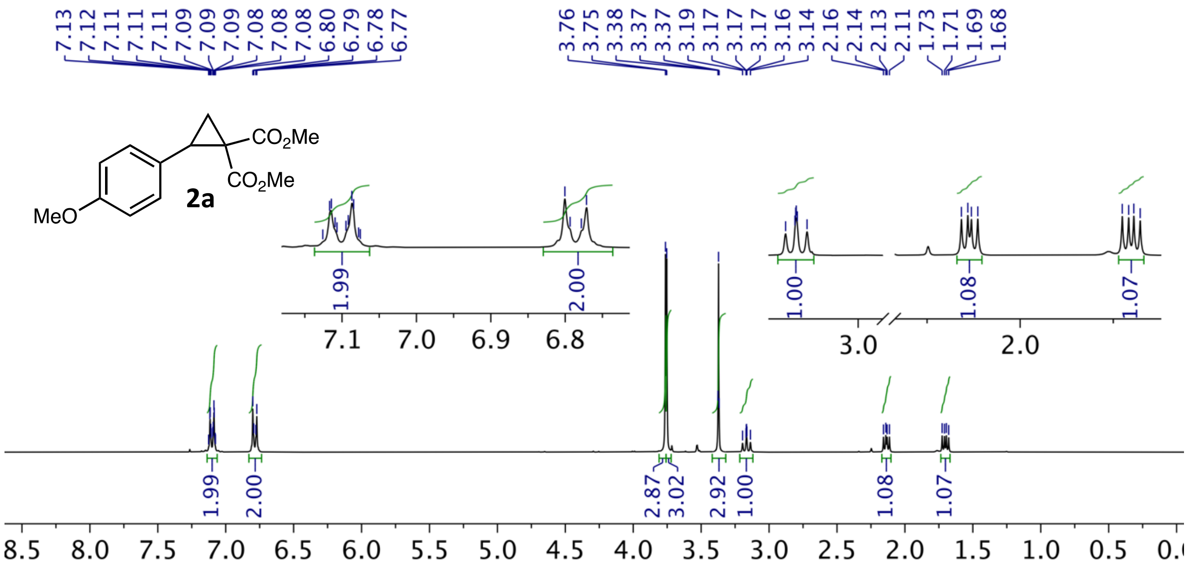

7.13 7.12 7.11 7.11 7.11 7.09 7.09 7.08 7.08 7.08 6.80 6.79 6.78 6.77
3.76 3.75 3.38 3.37 3.37 3.19 3.17 3.17 3.17 3.16 3.14 2.16 2.14 2.13 2.11 1.73 1.71 1.69 1.68
CO2Me
CO2Me
MeO
2a
1.99
2.00
7.1 7.0 6.9 6.8
1.00
1.08
1.07
3.0
2.0
1.99 2.00 2.87 3.02 2.92 1.00 1.08 1.07
8.5 8.0 7.5 7.0 6.5 6.0 5.5 5.0 4.5 4.0 3.5 3.0 2.5 2.0 1.5 1.0 0.5

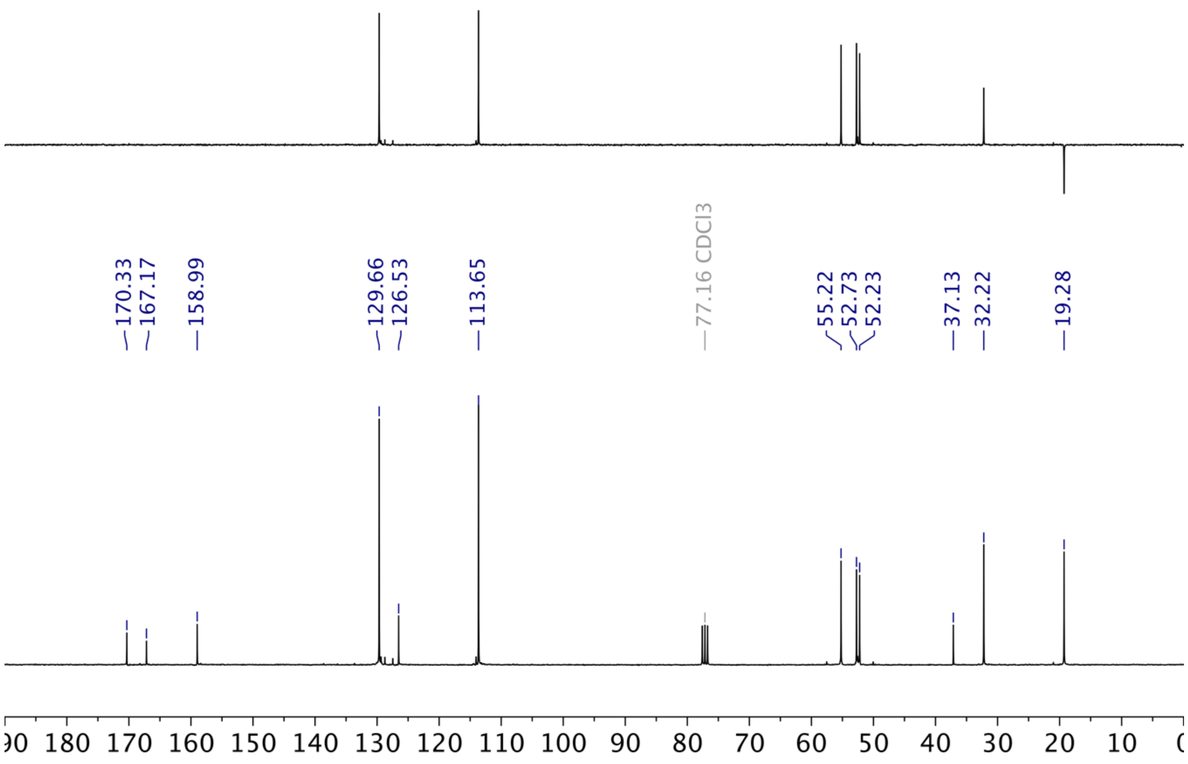

170.33
167.17
158.99
129.66
126.53
113.65
77.16 CDCl3
55.22
52.73
52.23
37.13
32.22
19.28
180 170 160 150 140 130 120 110 100 90 80 70 60 50 40 30 20 10

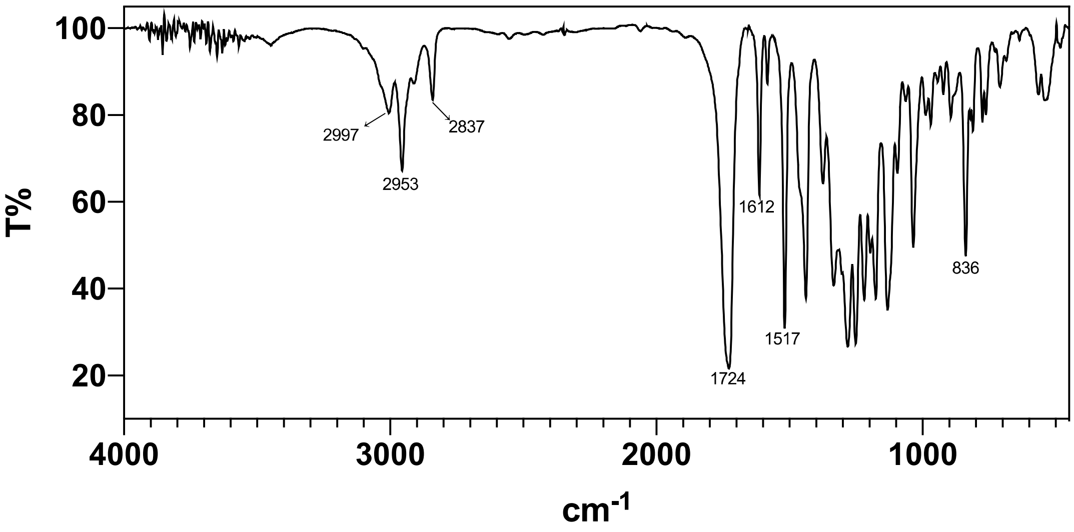

100
80
60
40
20
T%
2997
2953
2837
1724
1612
1517
836
4000
3000
2000
1000
cm-1

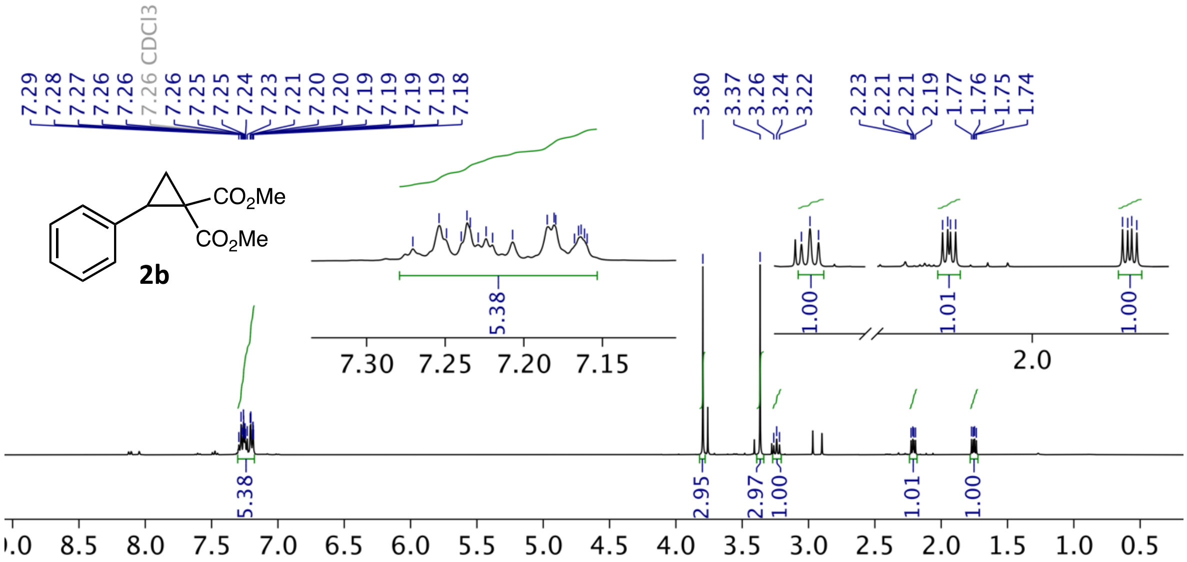
7.29
7.28
7.27
7.26
7.26
7.26 CDCl3
7.26
7.25
7.25
7.24
7.23
7.21
7.20
7.20
7.19
7.19
7.19
7.19
7.18
3.80
3.37
3.26
3.24
3.22
2.23
2.21
2.21
2.19
1.77
1.76
1.75
1.74
CO2Me
CO2Me
2b
5.38
7.30 7.25 7.20 7.15
1.00
1.01
1.00
2.0
5.38
2.95
2.97
1.00
1.01
1.00
9.0 8.5 8.0 7.5 7.0 6.5 6.0 5.5 5.0 4.5 4.0 3.5 3.0 2.5 2.0 1.5 1.0 0.5

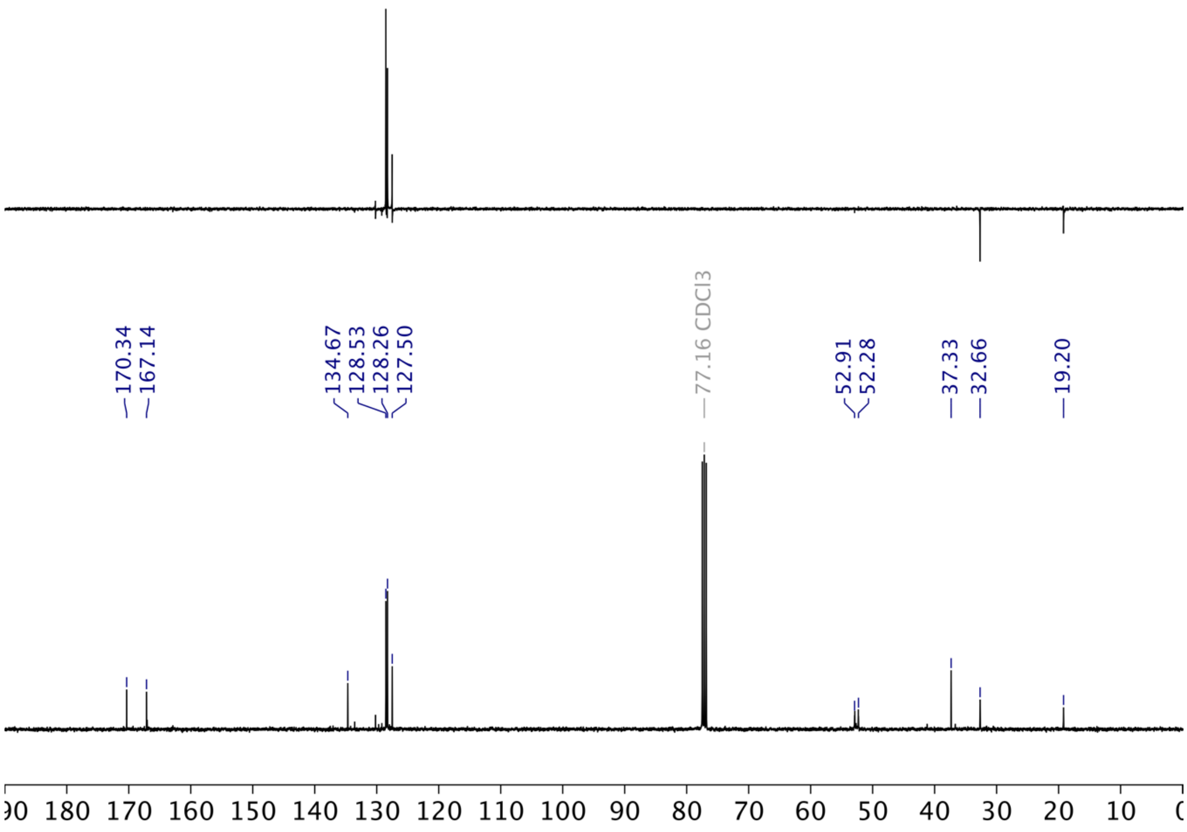
170.34
167.14
134.67
128.53
128.26
127.50
77.16 CDCl3
52.91
52.28
37.33
32.66
19.20
190 180 170 160 150 140 130 120 110 100 90 80 70 60 50 40 30 20 10 0

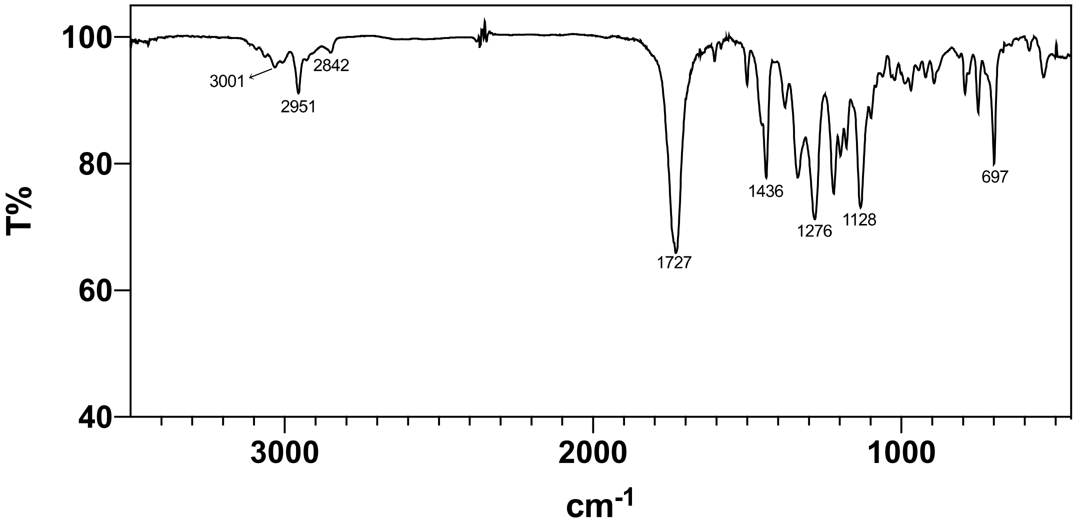

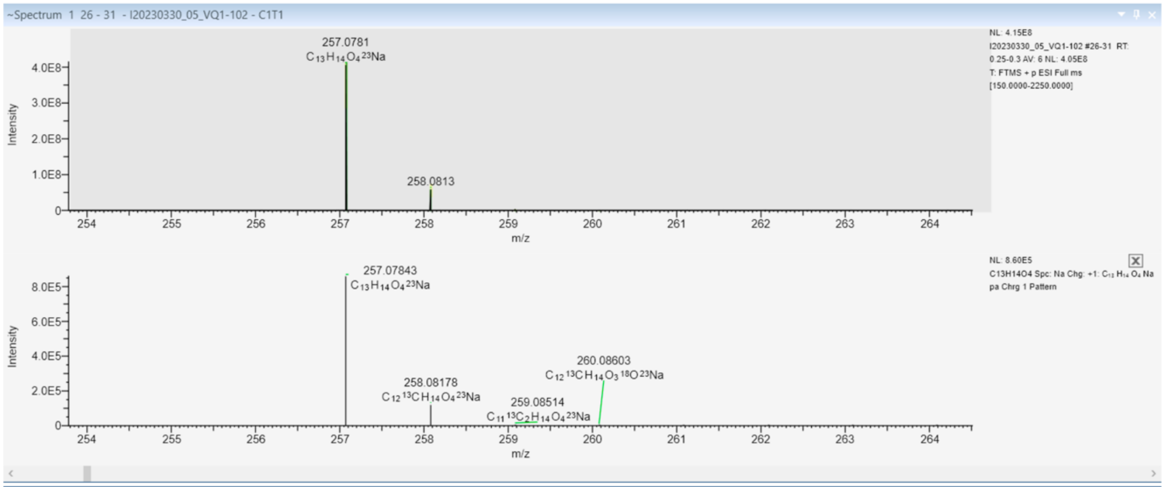

Elemental Composition Results - Spectrum 1

Peak Mass	Display Formula	S Fit	RDB	Delta [ppm]	Theo. mass	Rank	Combined Score	# Matched Iso.	# Missed Iso.	MS Cov. [%]	Pattern Cov. [%]
257.0781	$C_{13}H_{14}O_4{}^{23}Na$	54.21	6.50	-1.15	257.07843	1	97.42	5	0	99.82	100.00

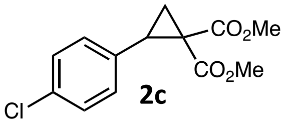
CO2Me
CO2Me
Cl
2c

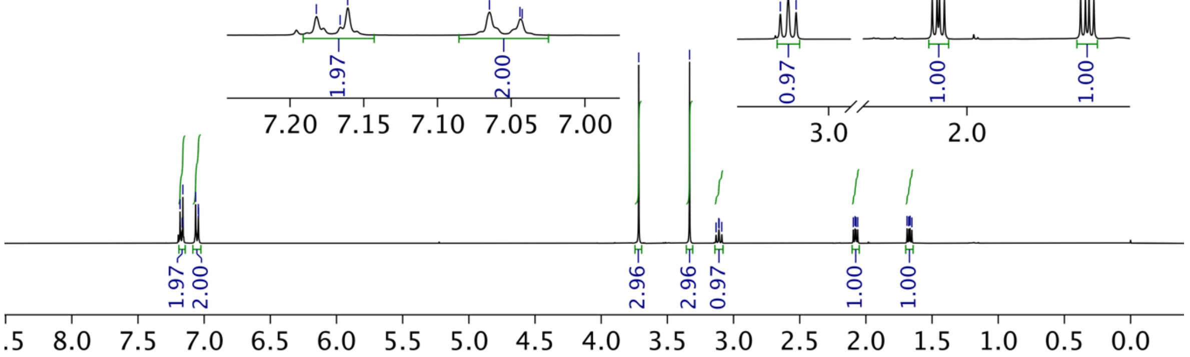
7.18
7.17
7.16
7.06
7.04
7.04
3.72
3.33
3.13
3.11
3.11
3.09
2.09
2.08
2.07
2.06
1.69
1.68
1.67
1.65
1.97
2.00
7.20 7.15 7.10 7.05 7.00
0.97
1.00
1.00
3.0
2.0
2.96
2.96
0.97
1.00
1.00
.5 8.0 7.5 7.0 6.5 6.0 5.5 5.0 4.5 4.0 3.5 3.0 2.5 2.0 1.5 1.0 0.5 0.0

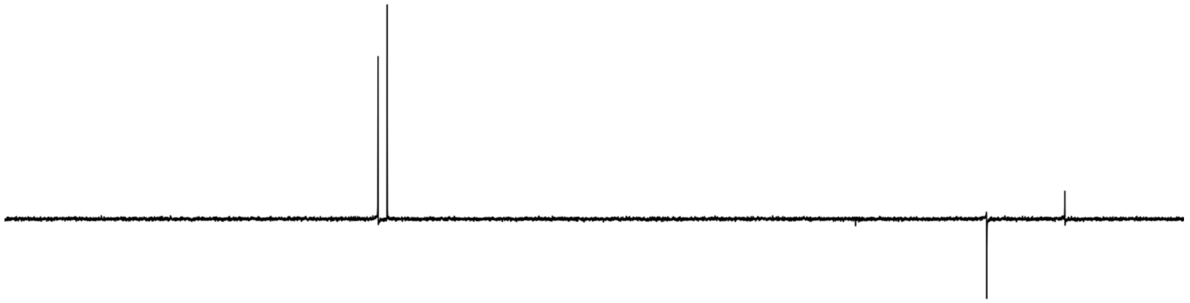

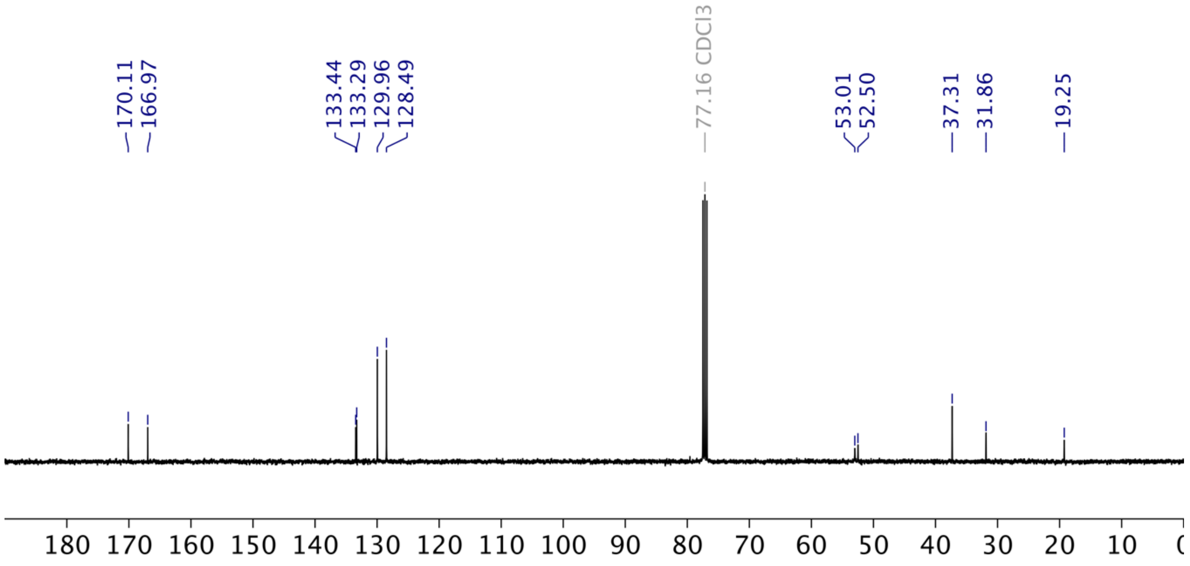
170.11
166.97
133.44
133.29
129.96
128.49
77.16 CDCl3
53.01
52.50
37.31
31.86
19.25
180 170 160 150 140 130 120 110 100 90 80 70 60 50 40 30 20 10

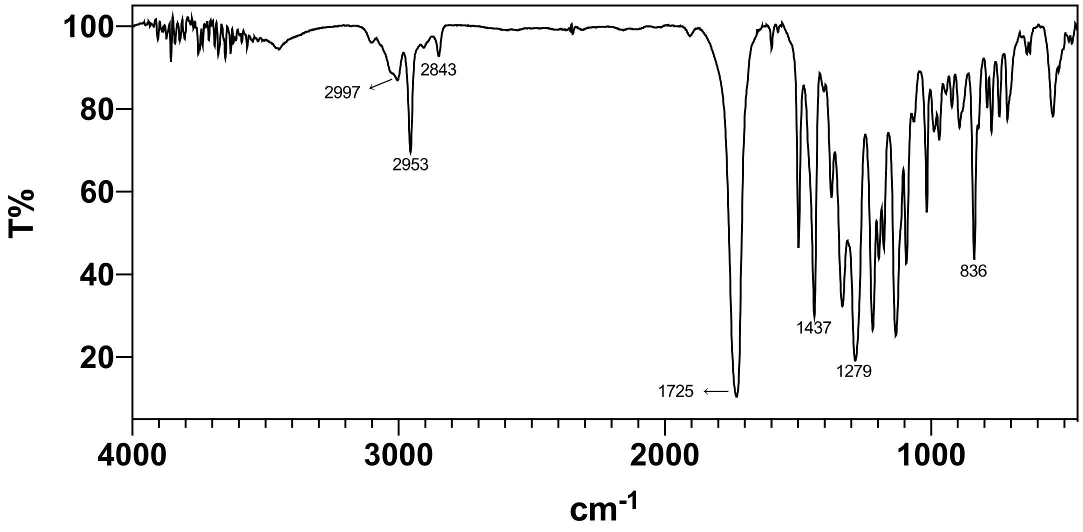

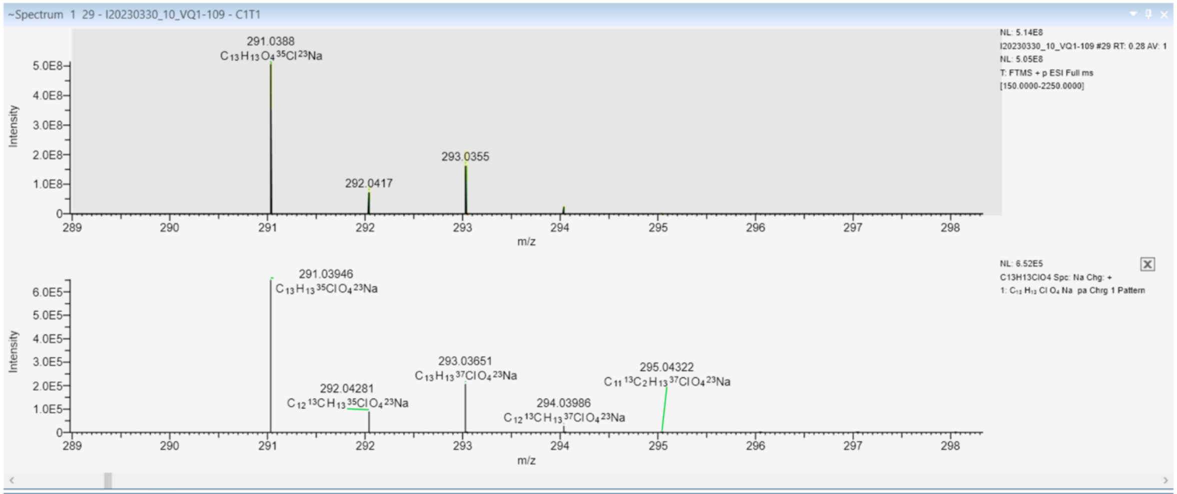

Elemental Composition Results - Spectrum 1

Peak Mass	Display Formula	S Fit	RDB	Delta [ppm]	Theo. mass	Rank	Combined Score	# Matched Iso.	# Missed Iso.	MS Cov. [%]	Pattern Cov. [%]
291.0388	$C_{13}H_{13}O_4{}^{35}Cl^{23}Na$	23.65	6.50	-2.30	291.03946	1	95.52	6	3	99.51	98.37

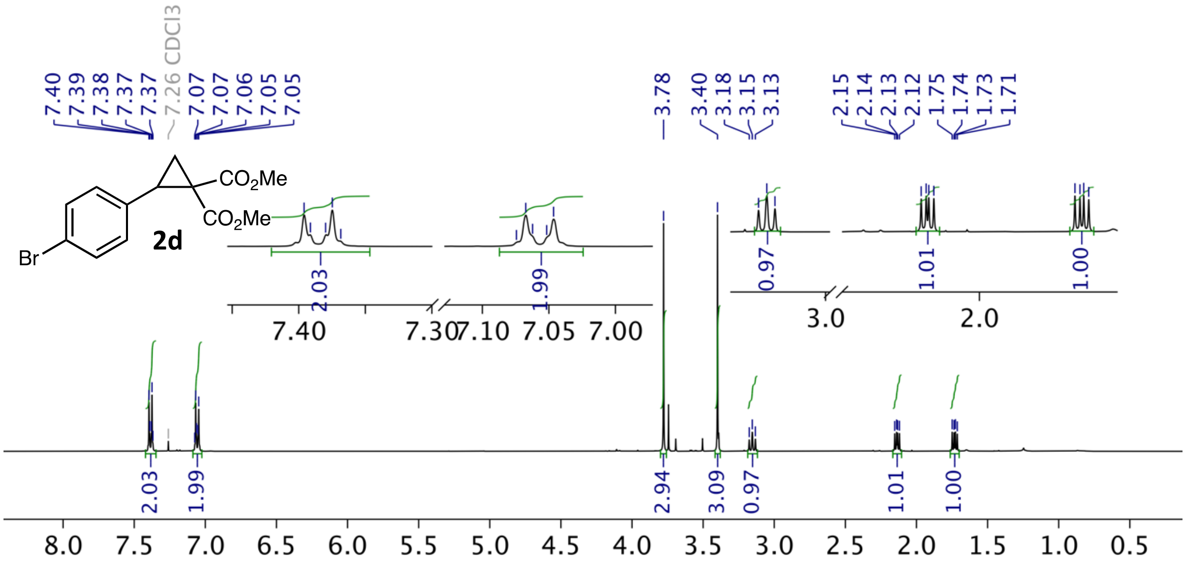
7.40 7.39 7.38 7.37 7.37
7.26 CDCl3
7.07 7.07 7.06 7.05 7.05
3.78
3.40 3.18 3.15 3.13
2.15 2.14 2.13 2.12 1.75 1.74 1.73 1.71
CO_2Me
CO_2Me
Br
2d
2.03 1.99 0.97 1.01 1.00
7.40 7.30 7.10 7.05 7.00
3.0 2.0
2.03 1.99 2.94 3.09 0.97 1.01 1.00
8.0 7.5 7.0 6.5 6.0 5.5 5.0 4.5 4.0 3.5 3.0 2.5 2.0 1.5 1.0 0.5

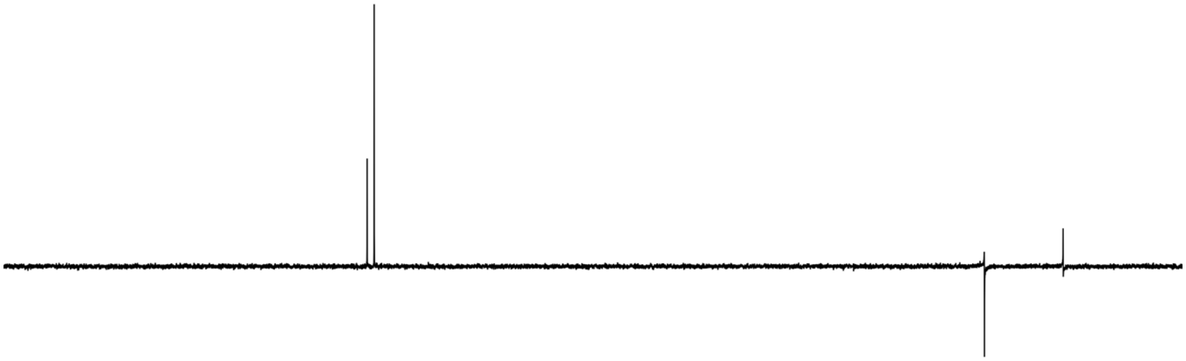

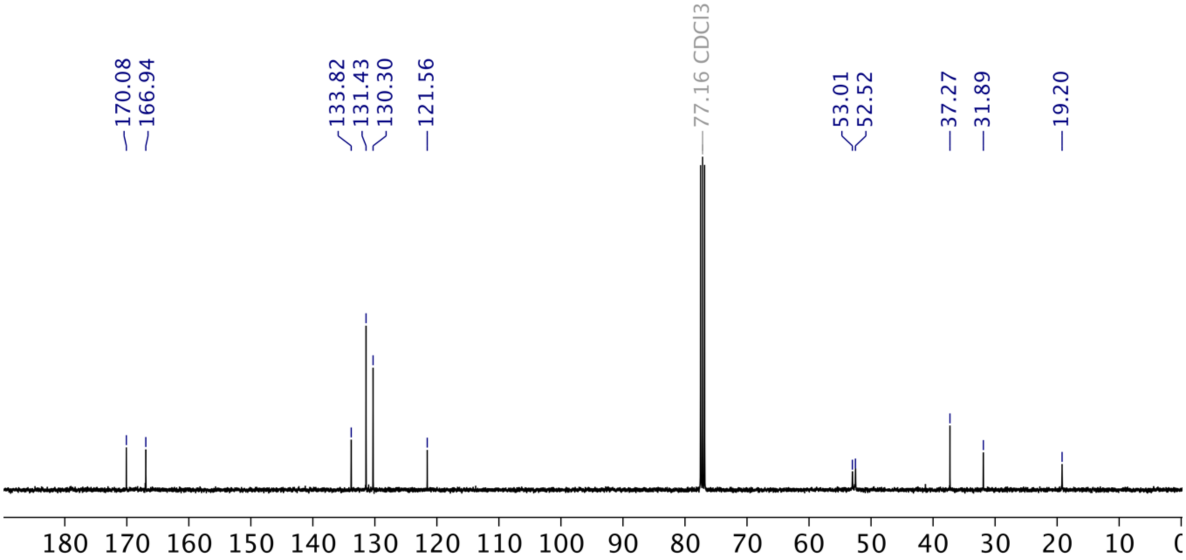
170.08 166.94
133.82 131.43 130.30
121.56
77.16 CDCl3
53.01 52.52
37.27 31.89
19.20
180 170 160 150 140 130 120 110 100 90 80 70 60 50 40 30 20 10

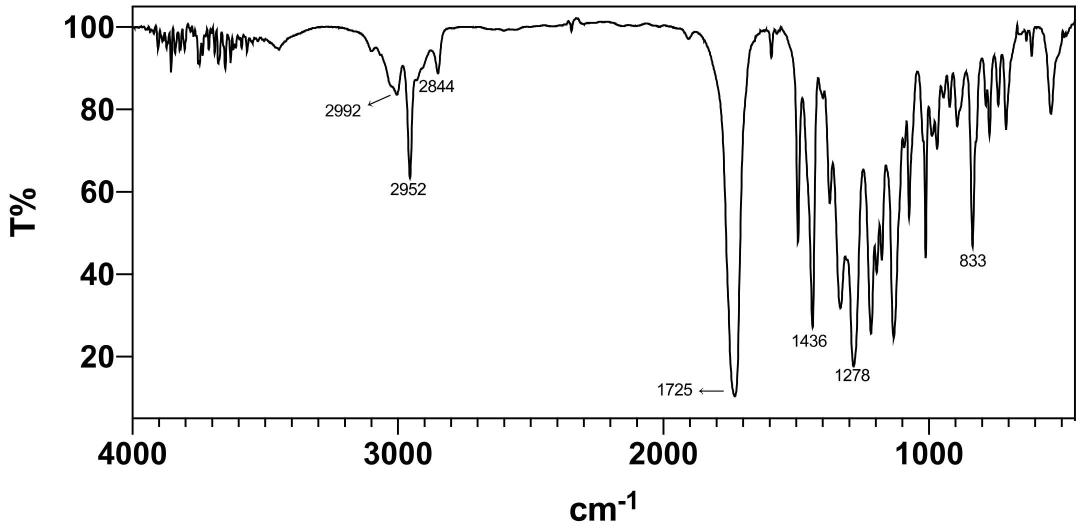

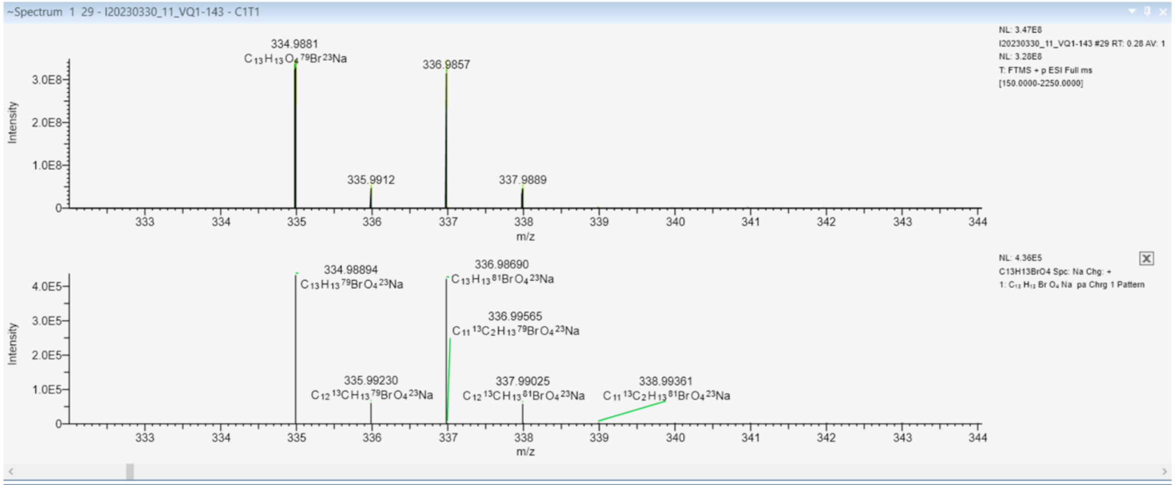

Elemental Composition Results - Spectrum 1

Peak Mass	Display Formula	S Fit	RDB	Delta [ppm]	Theo. mass	Rank	Combined Score	# Matched Iso.	# Missed Iso.	MS Cov. [%]	Pattern Cov. [%]
334.9881	$C_{13}H_{13}O_4{}^{79}Br^{23}Na$	34.51	6.50	-2.52	334.98894	1	95.73	6	2	99.13	99.05

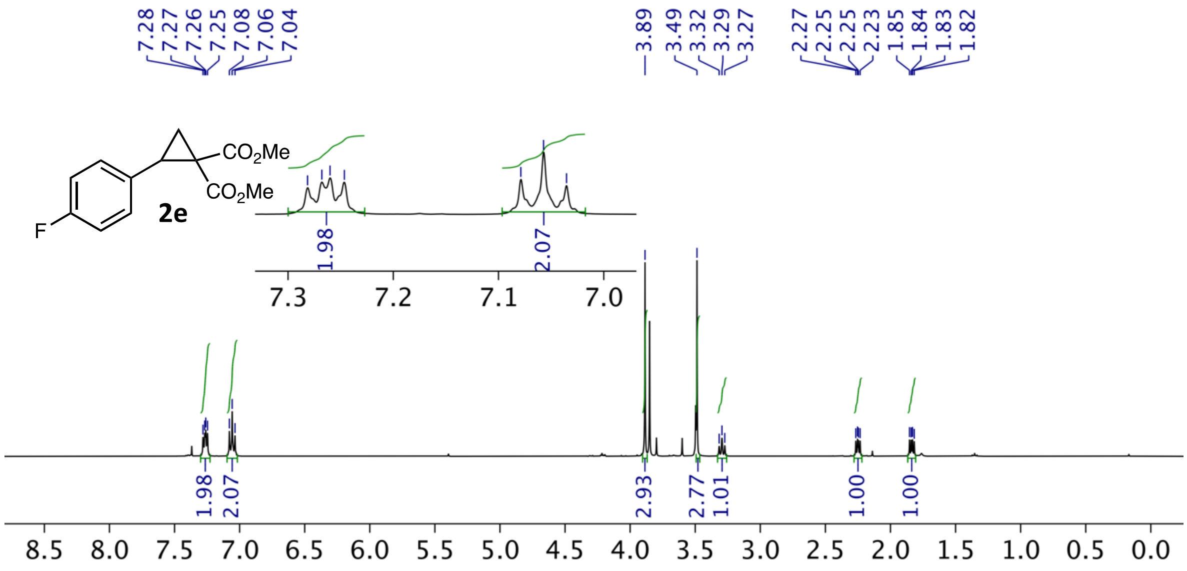
7.28
7.27
7.26
7.25
7.08
7.06
7.04
3.89
3.49
3.32
3.29
3.27
2.27
2.25
2.25
2.23
1.85
1.84
1.83
1.82
CO_2Me
CO_2Me
F
1.98
2.07
7.3
7.2
7.1
7.0
2.93
2.77
1.01
1.00
1.00
8.5 8.0 7.5 7.0 6.5 6.0 5.5 5.0 4.5 4.0 3.5 3.0 2.5 2.0 1.5 1.0 0.5 0.0

2e

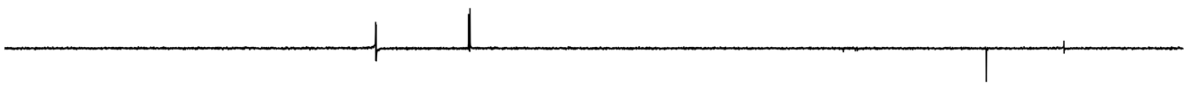

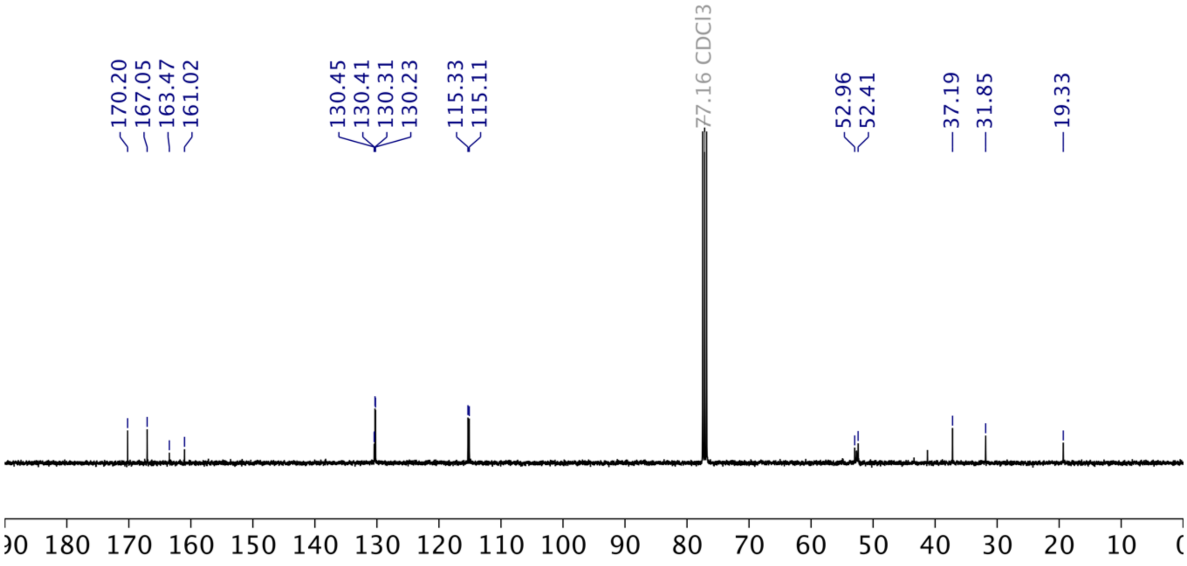
170.20
167.05
163.47
161.02
130.45
130.41
130.31
130.23
115.33
115.11
77.16 CDCl3
52.96
52.41
37.19
31.85
19.33
180 170 160 150 140 130 120 110 100 90 80 70 60 50 40 30 20 10

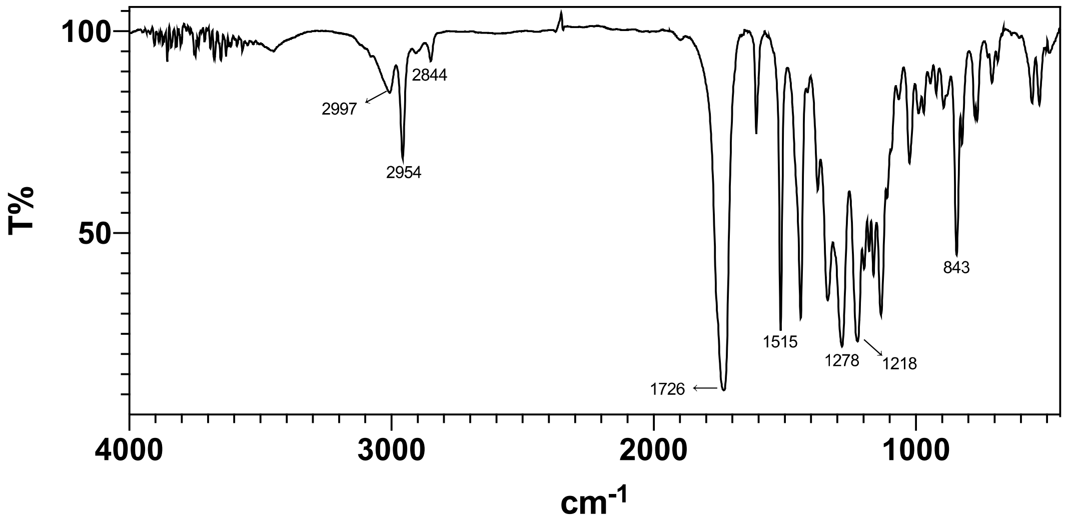

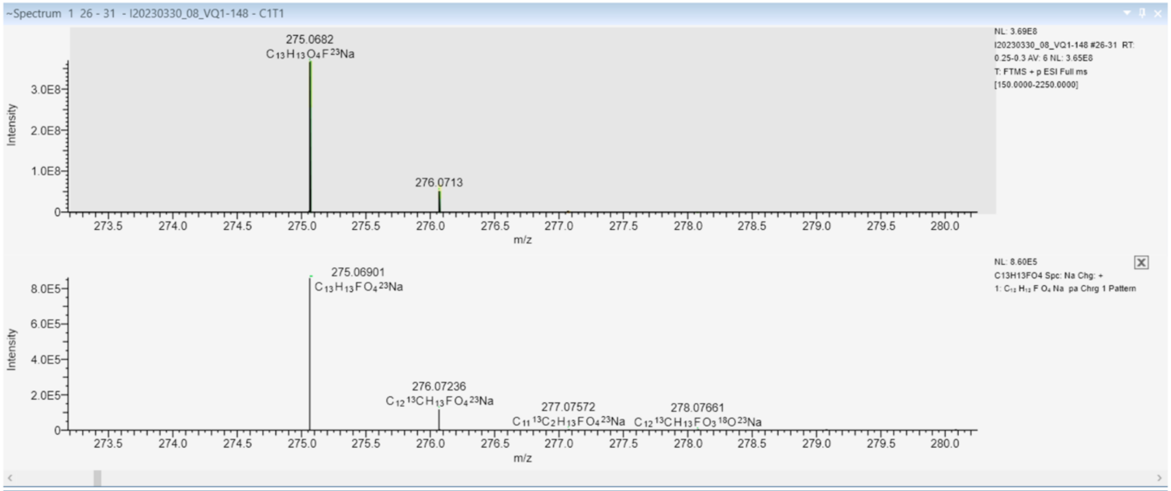

Peak Mass	Display Formula	S Fit	RDB	Delta [ppm]	Theo. mass	Rank	Combined Score	# Matched Iso.	# Missed Iso.	MS Cov. [%]	Pattern Cov. [%]
275.0682	$C_{13}H_{13}O_4F^{23}Na$	32.42	6.50	-2.80	275.06901	1	95.31	5	0	98.80	98.45

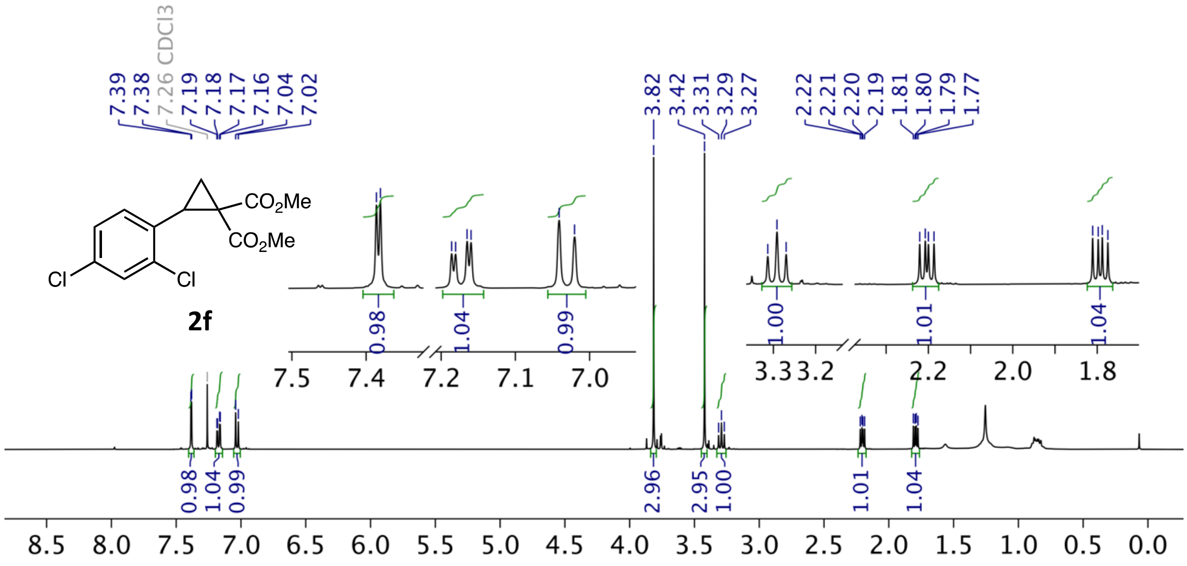
7.39
7.38
7.26 CDCl3
7.19
7.18
7.17
7.16
7.04
7.02
3.82
3.42
3.31
3.29
3.27
2.22
2.21
2.20
2.19
1.81
1.80
1.79
1.77
CO2Me
CO2Me
Cl
Cl
2f
0.98
1.04
0.99
1.00
1.01
1.04
7.5 7.4 7.2 7.1 7.0
3.3 3.2 2.2 2.0 1.8
0.98
1.04
0.99
2.96
2.95
1.00
1.01
1.04
8.5 8.0 7.5 7.0 6.5 6.0 5.5 5.0 4.5 4.0 3.5 3.0 2.5 2.0 1.5 1.0 0.5 0.0

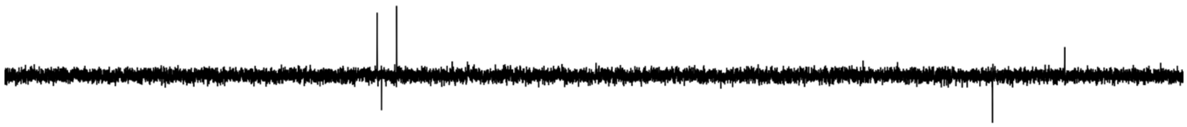

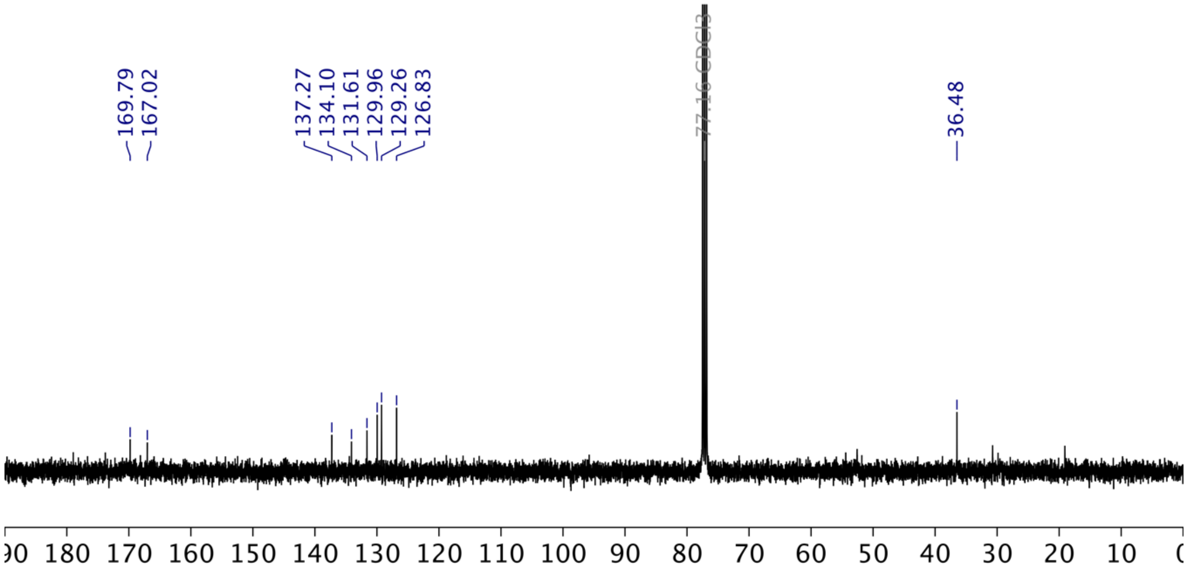
169.79
167.02
137.27
134.10
131.61
129.96
129.26
126.83
77.16 CDCl3
36.48
90 180 170 160 150 140 130 120 110 100 90 80 70 60 50 40 30 20 10 0

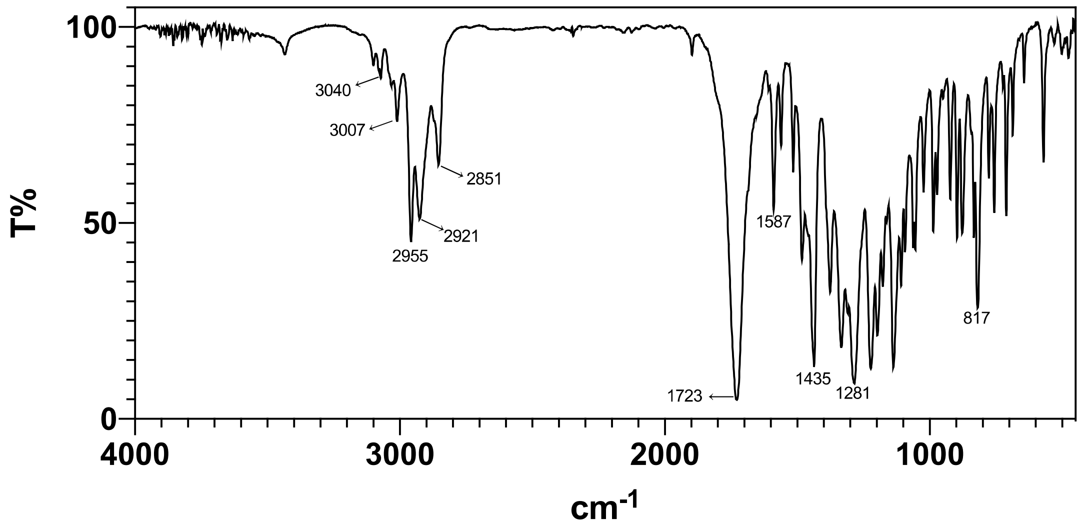

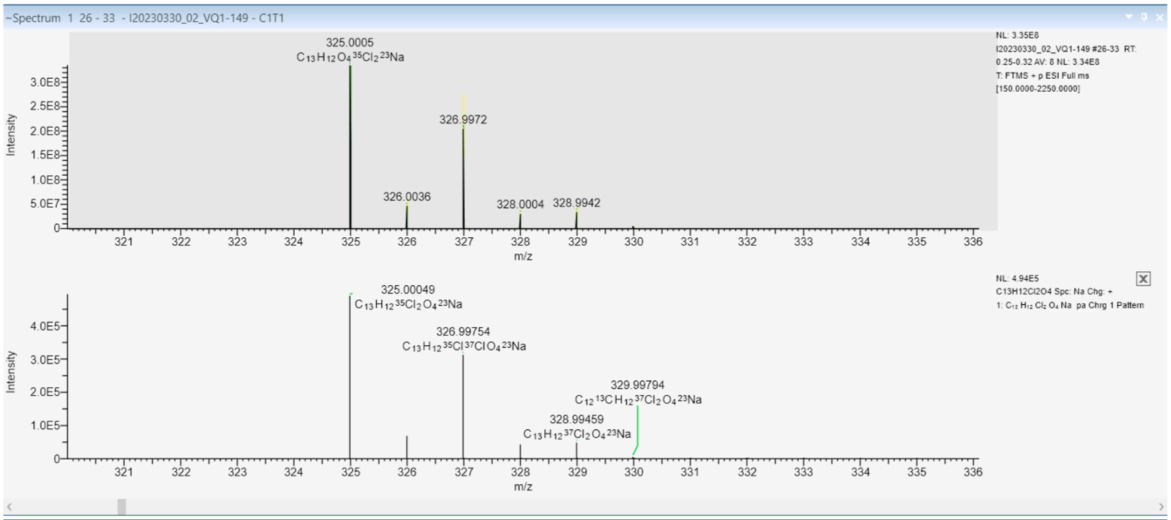

Elemental Composition Results - Spectrum 1

Peak Mass	Display Formula	S Fit	RDB	Delta [ppm]	Theo. mass	Rank	Combined Score	# Matched Iso.	# Missed Iso.	MS Cov. [%]	Pattern Cov. [%]
325.0005	$C_{13}H_{12}O_4{}^{35}Cl_2{}^{23}Na$	36.46	6.50	0.13	325.00049	1	95.92	10	0	99.23	99.04

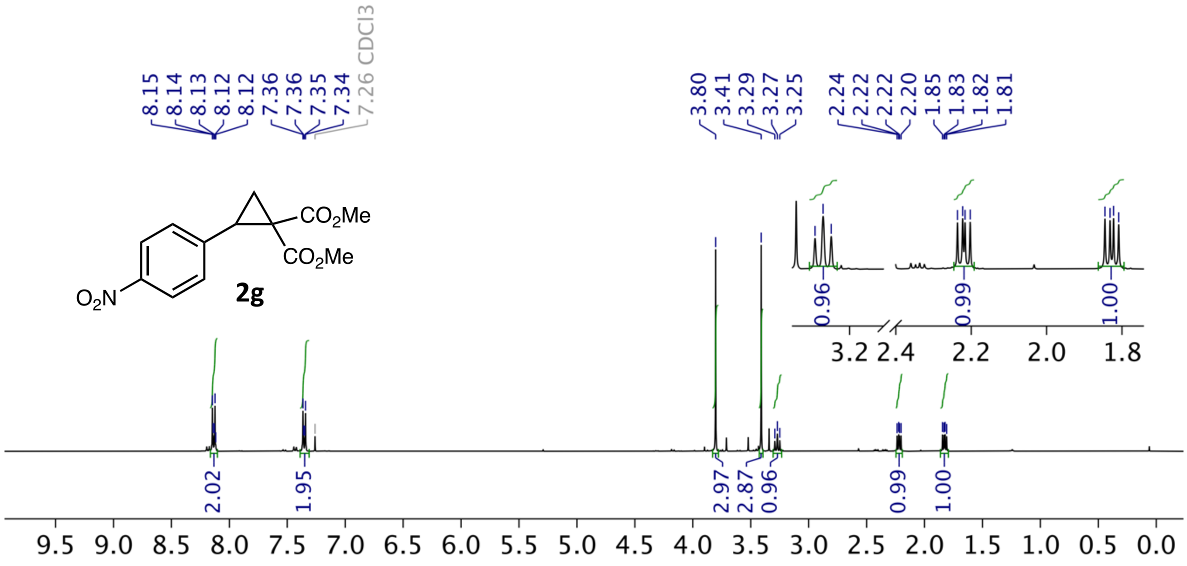
8.15
8.14
8.13
8.12
8.12
7.36
7.36
7.35
7.34
7.26 CDCl3
3.80
3.41
3.29
3.27
3.25
2.24
2.22
2.22
2.20
1.85
1.83
1.82
1.81
CO2Me
CO2Me
O2N
2g
0.96
0.99
1.00
3.2 2.4 2.2 2.0 1.8
2.02
1.95
2.97
2.87
0.96
0.99
1.00
9.5 9.0 8.5 8.0 7.5 7.0 6.5 6.0 5.5 5.0 4.5 4.0 3.5 3.0 2.5 2.0 1.5 1.0 0.5 0.0

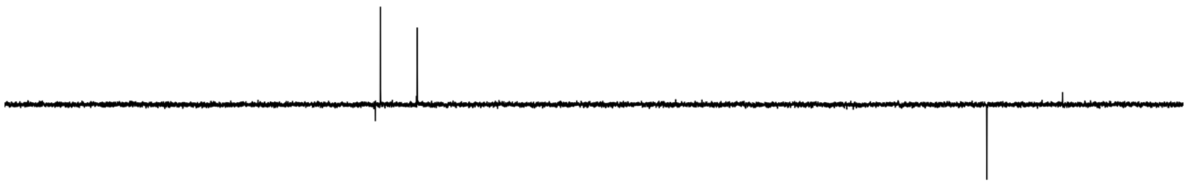

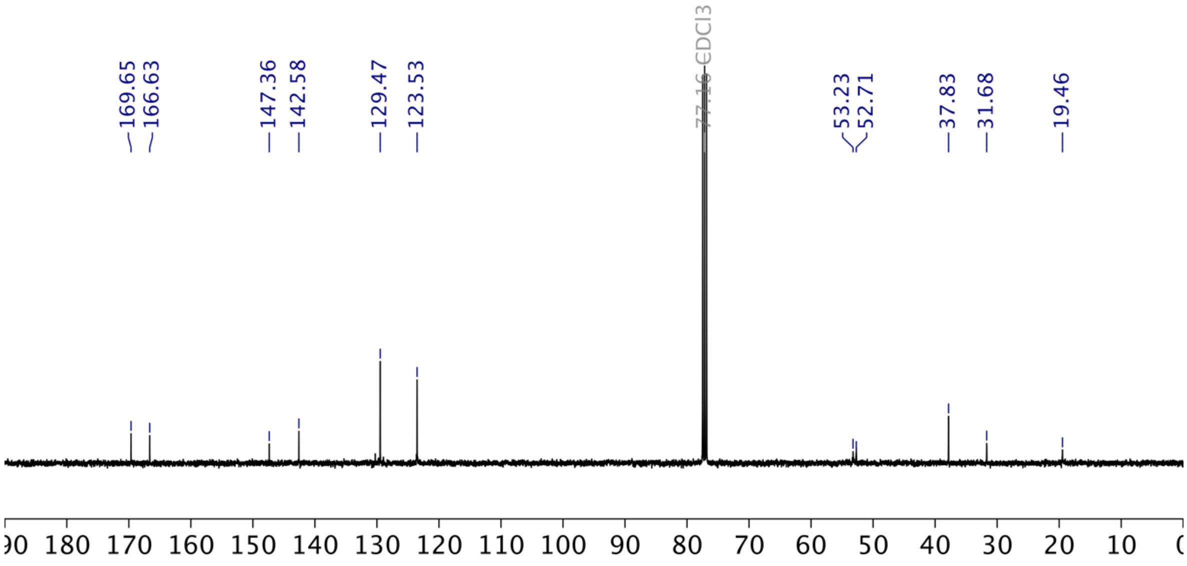
169.65
166.63
147.36
142.58
129.47
123.53
77.16 CDCl3
53.23
52.71
37.83
31.68
19.46
180 170 160 150 140 130 120 110 100 90 80 70 60 50 40 30 20 10

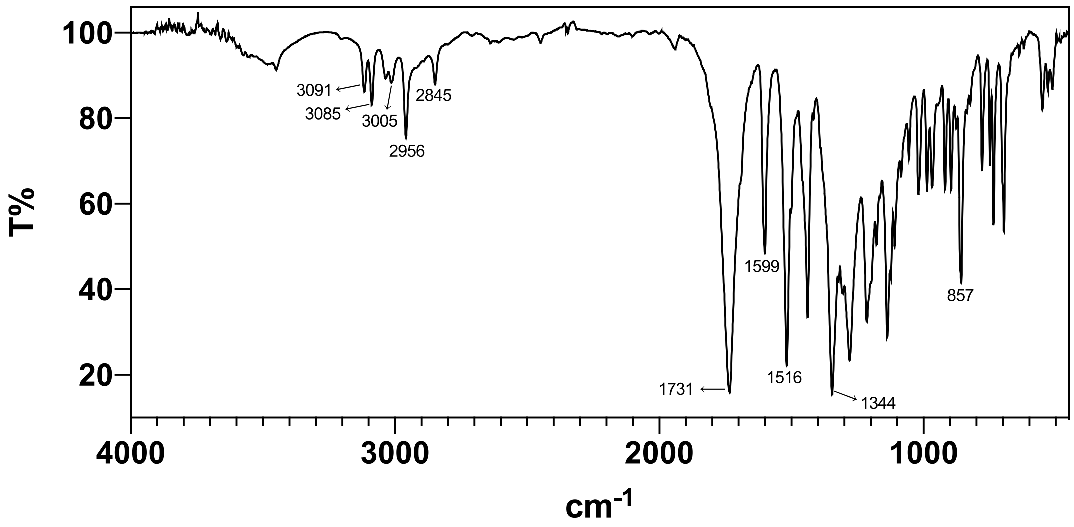

ThermoFisher Orbitrap: Exactive Plus with Extend Mass Range: Source HESI II Ion Polarity: Positive

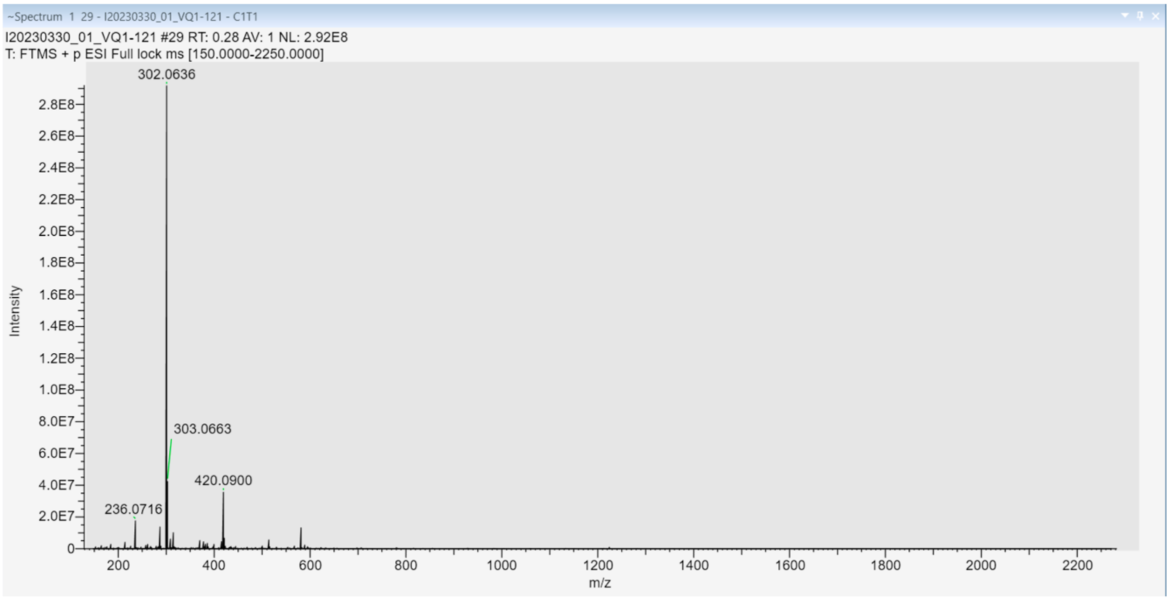

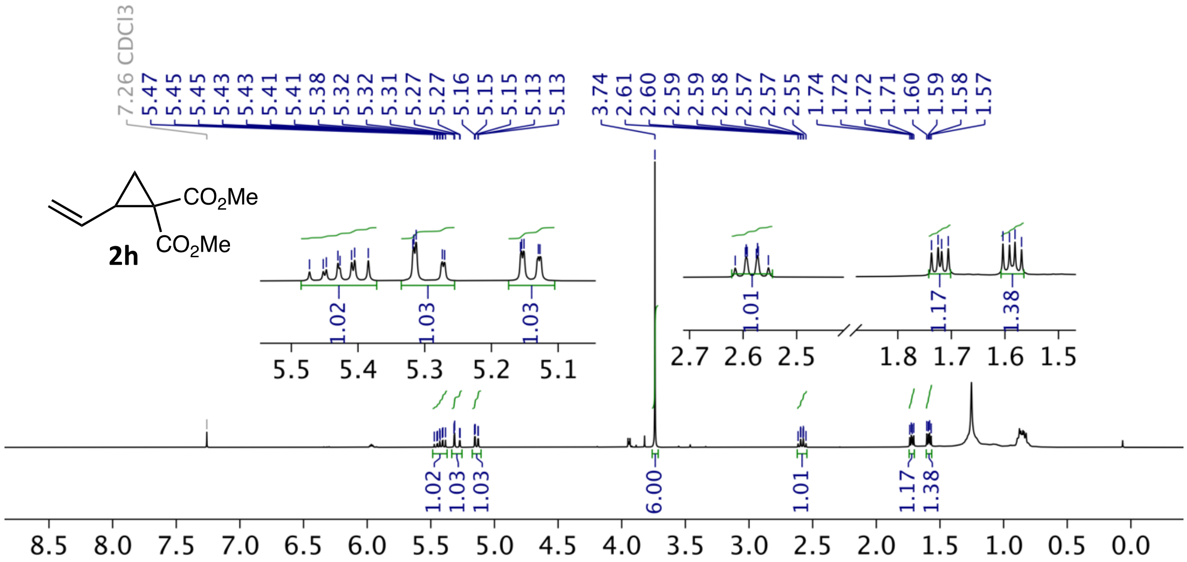
7.26 CDCl3
5.47 5.45 5.45 5.43 5.43 5.41 5.41 5.38 5.32 5.32 5.31 5.27 5.27 5.16 5.15 5.15 5.13 5.13
3.74 2.61 2.60 2.59 2.59 2.58 2.57 2.57 2.55 1.74 1.72 1.72 1.71 1.60 1.59 1.58 1.57
CO2Me
CO2Me
2h
1.02 1.03 1.03 6.00 1.01 1.17 1.38
8.5 8.0 7.5 7.0 6.5 6.0 5.5 5.0 4.5 4.0 3.5 3.0 2.5 2.0 1.5 1.0 0.5 0.0

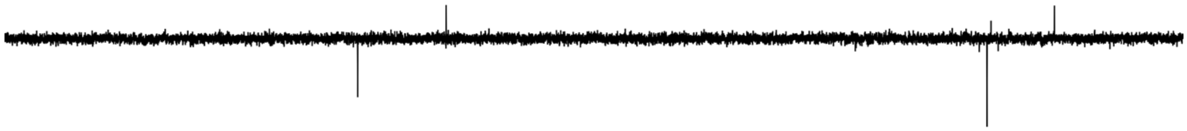

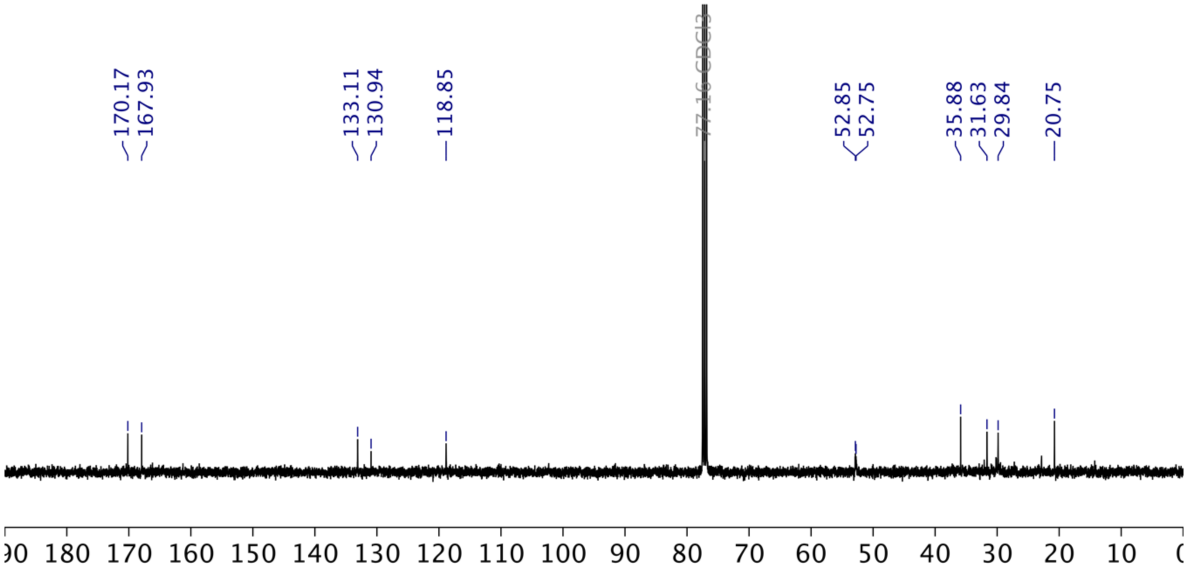
170.17 167.93
133.11 130.94
118.85
77.16 CDCl3
52.85 52.75
35.88 31.63 29.84
20.75
180 170 160 150 140 130 120 110 100 90 80 70 60 50 40 30 20 10

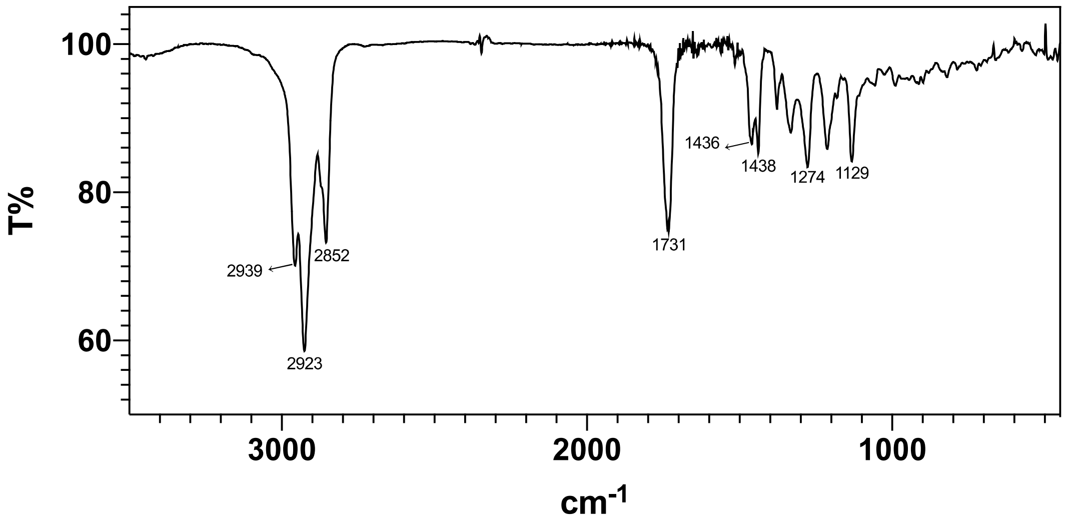
100
80
60
T%
2939
2923
2852
1731
1436
1438
1274
1129
3000
2000
1000
cm-1

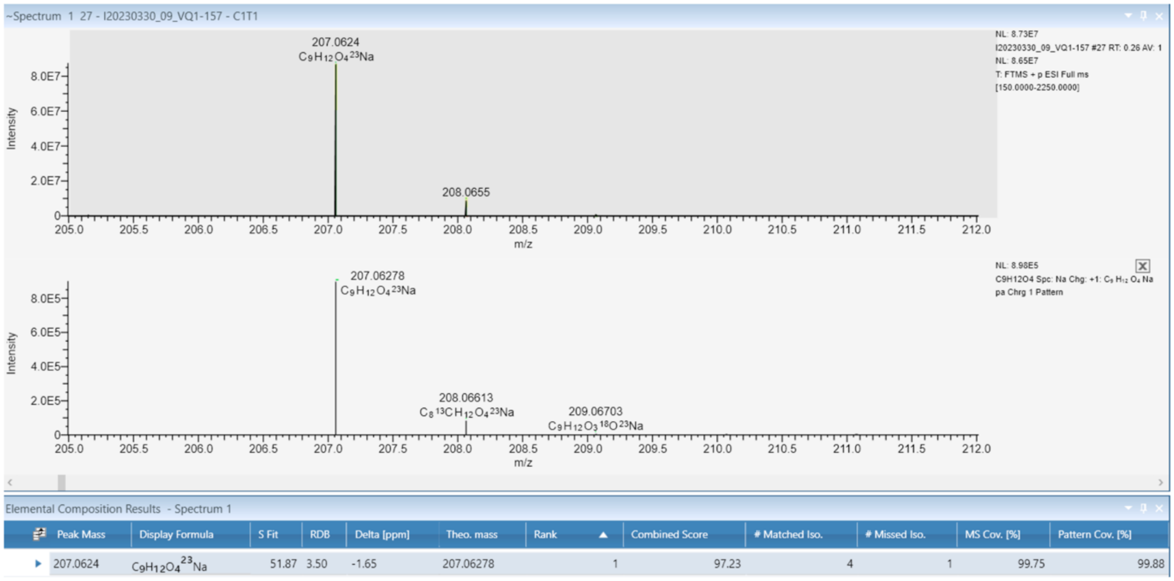
~Spectrum 1 27 - I20230330_09_VQ1-157 - C1T1
207.0624
C9H12O4 23Na
208.0655
Intensity
m/z
NL: 8.73E7
I20230330_09_VQ1-157 #27 RT: 0.26 AV: 1
NL: 8.65E7
T: FTMS + p ESI Full ms
[150.0000-2250.0000]
207.06278
C9H12O4 23Na
208.06613
C8 13CH12O4 23Na
209.06703
C9H12O3 18O 23Na
NL: 8.98E5
C9H12O4 Spc: Na Chg: +1: C9 H12 O4 Na
pa Chrg 1 Pattern
Elemental Composition Results - Spectrum 1
Peak Mass | Display Formula | S Fit | RDB | Delta [ppm] | Theo. mass | Rank | Combined Score | # Matched Iso. | # Missed Iso. | MS Cov. [%] | Pattern Cov. [%]
207.0624 | C9H12O4 23Na | 51.87 | 3.50 | -1.65 | 207.06278 | 1 | 97.23 | 4 | 1 | 99.75 | 99.88

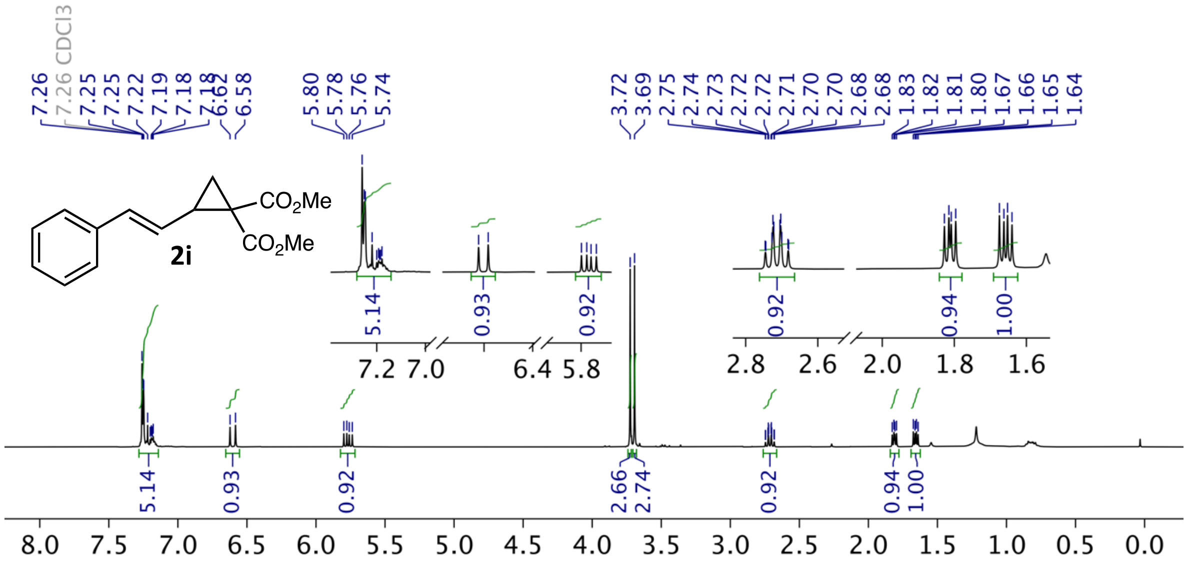
7.26
7.26 CDCl3
7.25
7.25
7.22
7.19
7.18
7.18
6.62
6.58
5.80
5.78
5.76
5.74
3.72
3.69
2.75
2.74
2.73
2.72
2.72
2.71
2.70
2.70
2.68
2.68
1.83
1.82
1.81
1.80
1.67
1.66
1.65
1.64
CO2Me
CO2Me
2i
5.14
0.93
0.92
0.92
0.94
1.00
7.2 7.0
6.4 5.8
2.8 2.6 2.0 1.8 1.6
2.66
2.74
8.0 7.5 7.0 6.5 6.0 5.5 5.0 4.5 4.0 3.5 3.0 2.5 2.0 1.5 1.0 0.5 0.0

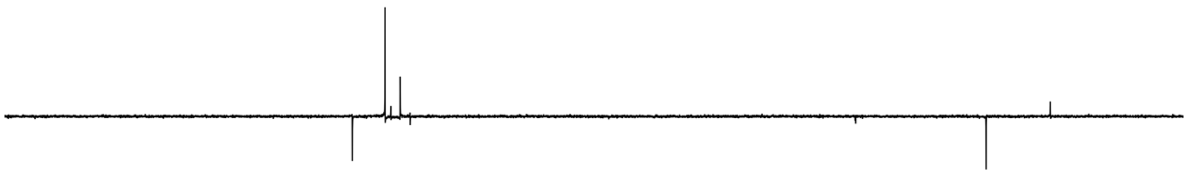

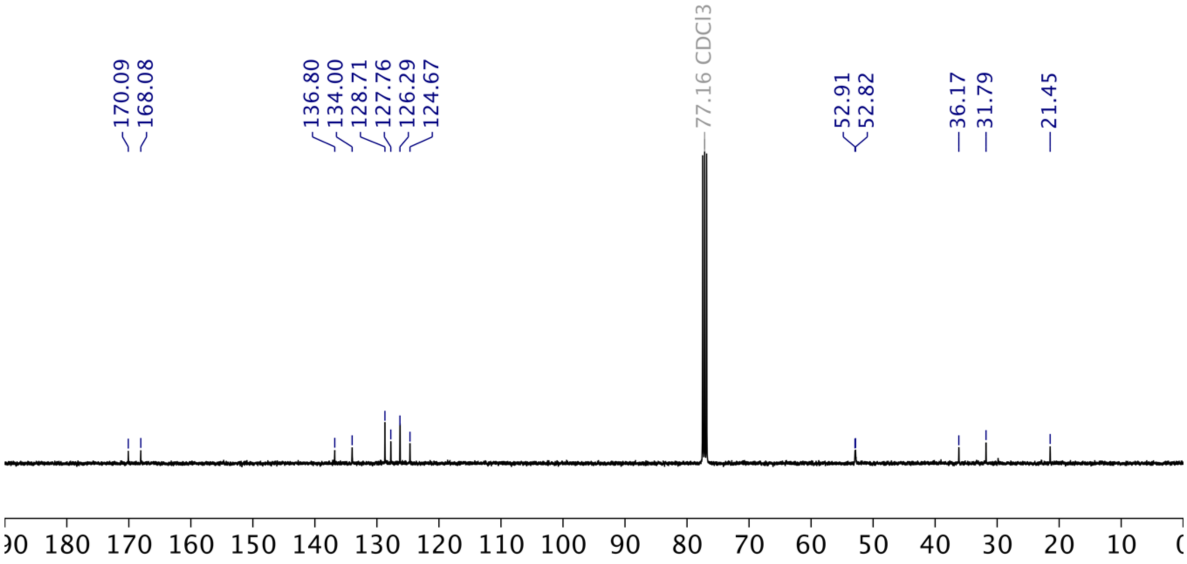
170.09
168.08
136.80
134.00
128.71
127.76
126.29
124.67
77.16 CDCl3
52.91
52.82
36.17
31.79
21.45
180 170 160 150 140 130 120 110 100 90 80 70 60 50 40 30 20 10

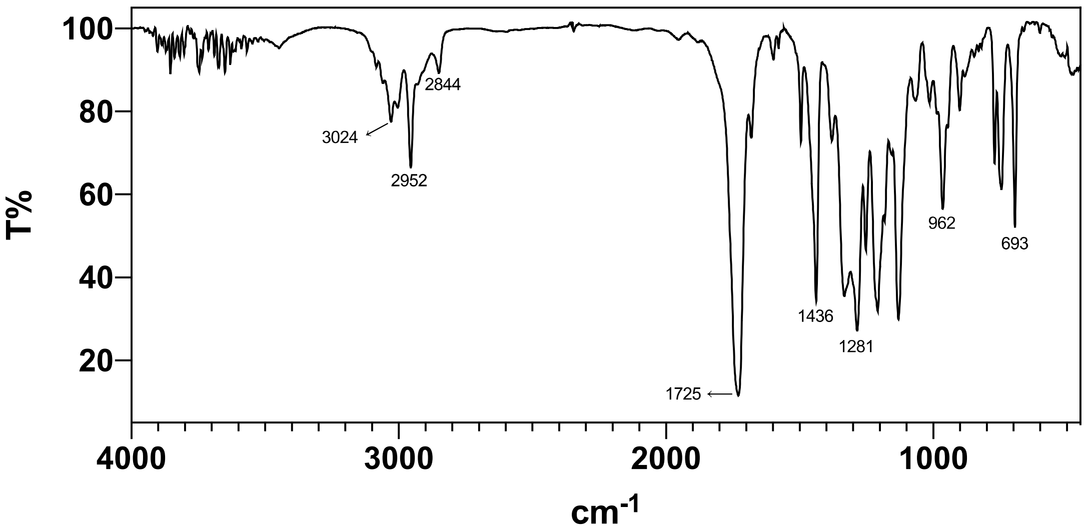

Acquisition Parameter				Set Corrector Fill	48.2 V
Source Type	ESI	Ion Polarity	Positive	n/a	n/a
n/a	n/a	n/a	n/a	n/a	n/a
Scan Begin	50 m/z	n/a	n/a	Set Reflector	1800.0 V
Scan End	3000 m/z	n/a	n/a	Set Flight Tube	8600.0 V
		n/a	n/a	Set Detector TOF	2021.6 V

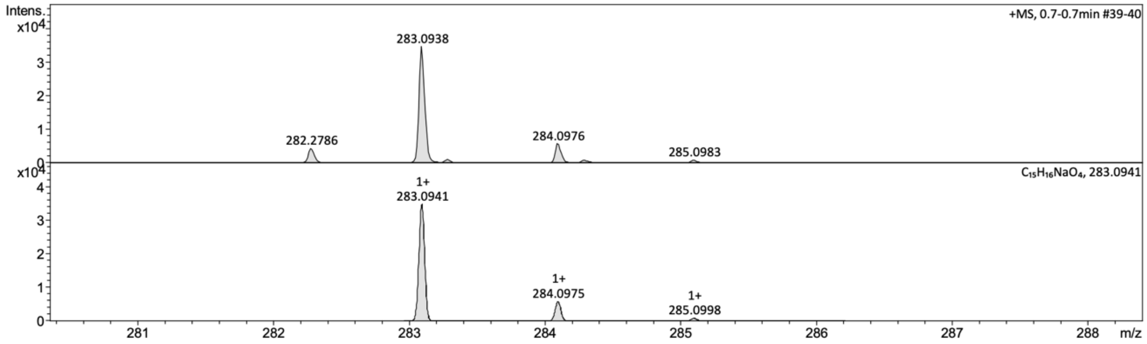

Meas. m/z	#	Ion Formula	m/z	err [ppm]	Mean err [ppm]	rdb	N-Rule	e⁻ Conf	mSigma	Std I	Std Mean m/z	Std I VarNorm	Std m/z Diff	Std Comb Dev
283.093844	1	C15H16NaO4	283.094080	0.8	-8.4	7.5	ok	even	2.8	4.1	n.a.	n.a.	n.a.	n.a.

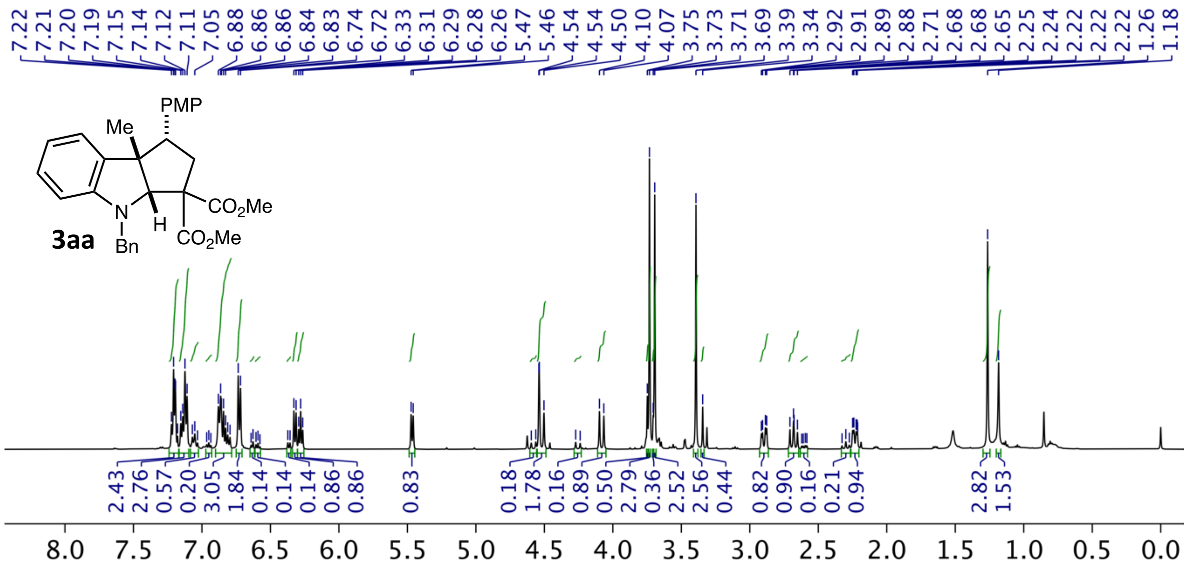
7.22 7.21 7.20 7.19 7.15 7.14 7.12 7.11 7.05 6.88 6.86 6.84 6.83 6.74 6.72 6.33 6.31 6.29 6.28 6.26 5.47 5.46 4.54 4.54 4.50 4.10 4.07 3.75 3.73 3.71 3.69 3.39 3.34 2.92 2.91 2.89 2.88 2.71 2.68 2.68 2.65 2.25 2.24 2.22 2.22 2.22 1.26 1.18
PMP
Me
CO_2Me
CO_2Me
N
H
Bn
3aa
2.43 2.76 0.57 0.20 3.05 1.84 0.14 0.14 0.14 0.86 0.86 0.83 0.18 1.78 0.16 0.89 0.50 2.79 0.36 2.52 2.56 0.44 0.82 0.90 0.16 0.21 0.94 2.82 1.53
8.0 7.5 7.0 6.5 6.0 5.5 5.0 4.5 4.0 3.5 3.0 2.5 2.0 1.5 1.0 0.5 0.0

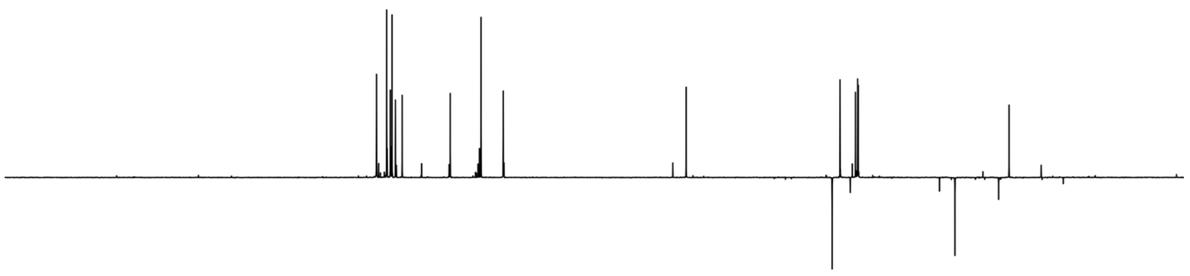

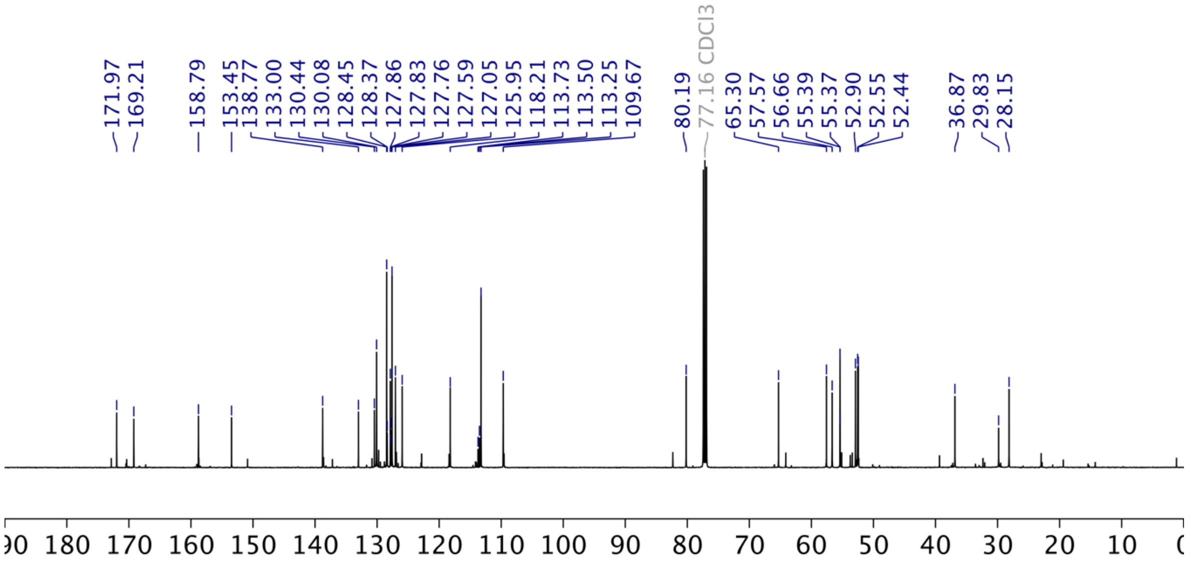
171.97 169.21 158.79 153.45 138.77 133.00 130.44 130.08 128.45 128.37 127.86 127.83 127.76 127.59 127.05 125.95 118.21 113.73 113.50 113.25 109.67 80.19 77.16 CDCl3 65.30 57.57 56.66 55.39 55.37 52.90 52.55 52.44 36.87 29.83 28.15
90 180 170 160 150 140 130 120 110 100 90 80 70 60 50 40 30 20 10 0

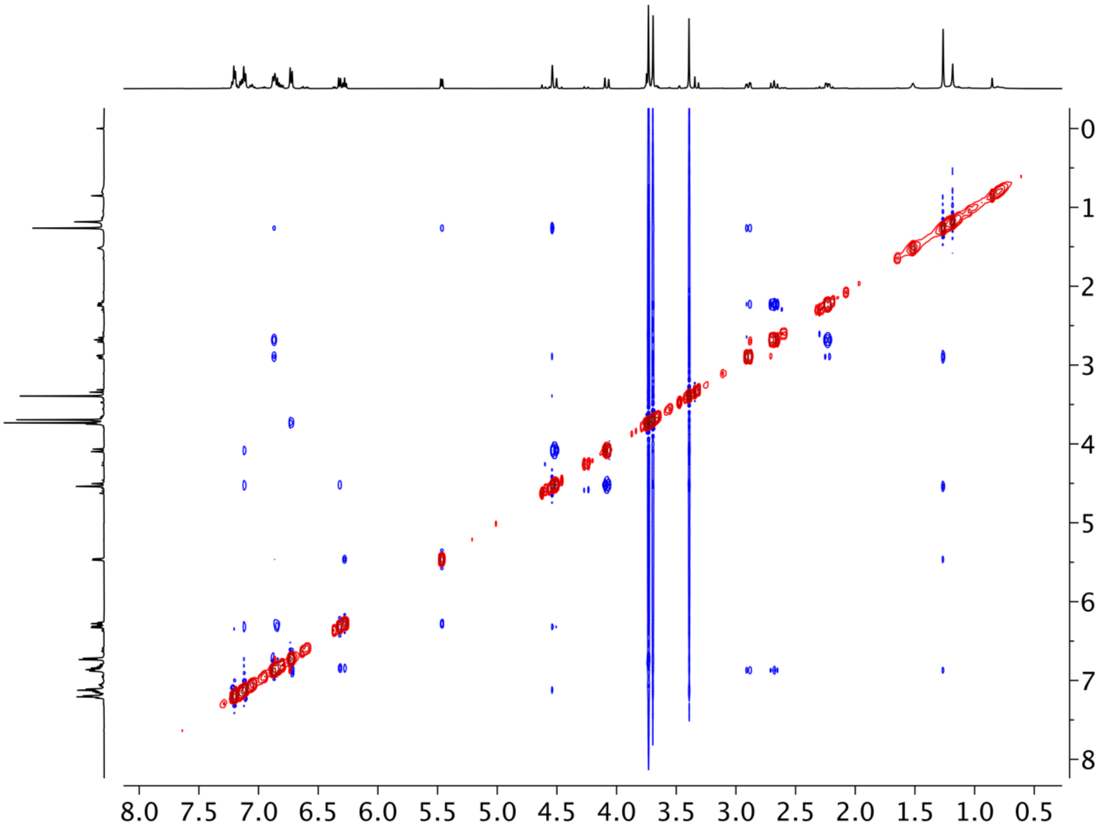
0
1
2
3
4
5
6
7
8
8.0 7.5 7.0 6.5 6.0 5.5 5.0 4.5 4.0 3.5 3.0 2.5 2.0 1.5 1.0 0.5

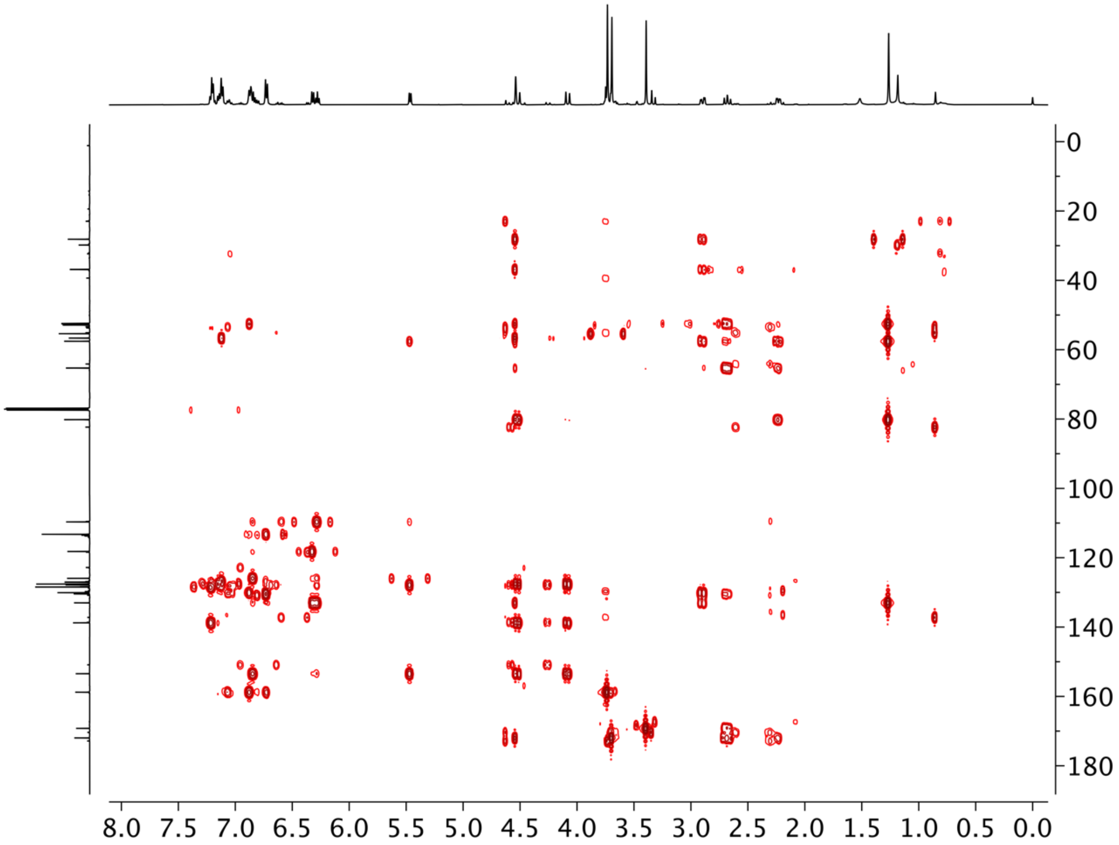
0
20
40
60
80
100
120
140
160
180
8.0 7.5 7.0 6.5 6.0 5.5 5.0 4.5 4.0 3.5 3.0 2.5 2.0 1.5 1.0 0.5 0.0

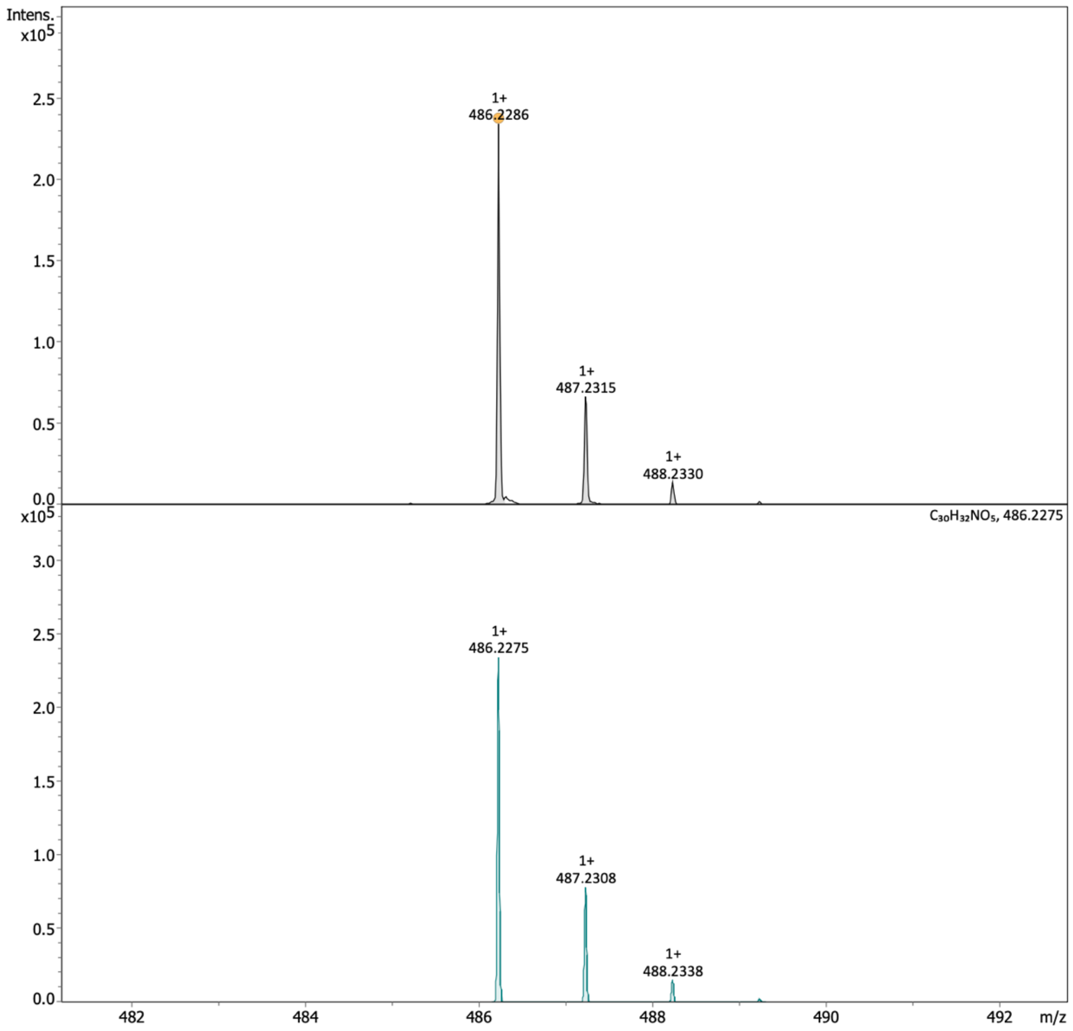
Intens.
x10^5
1+
486.2286
1+
487.2315
1+
488.2330
C30H32NO5, 486.2275
1+
486.2275
1+
487.2308
1+
488.2338
m/z

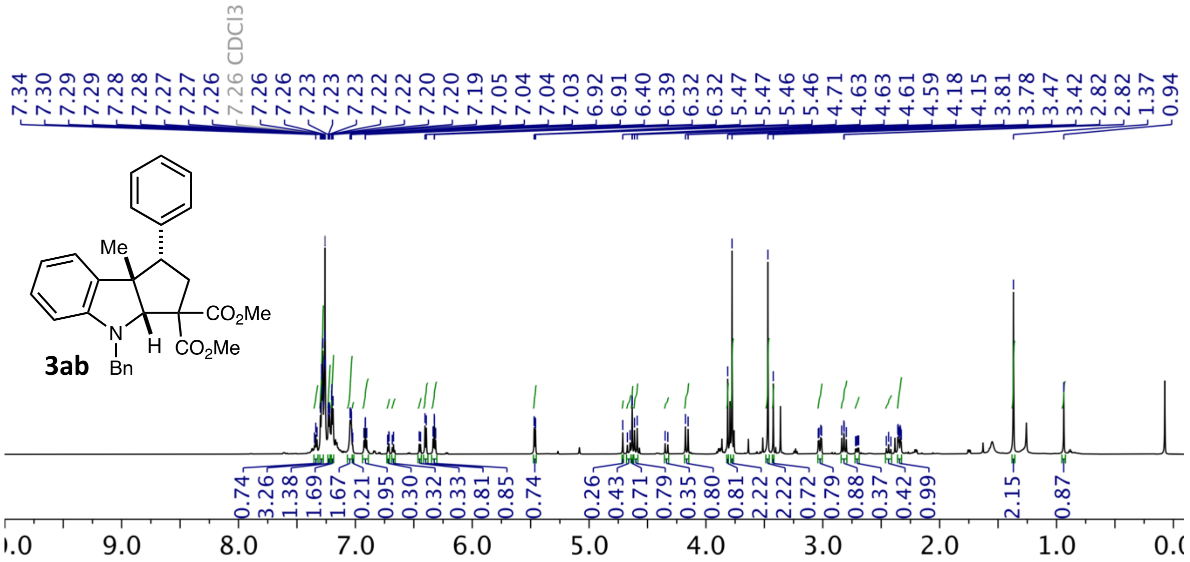
7.34
7.30
7.29
7.29
7.28
7.28
7.27
7.27
7.26 CDCl3
7.26
7.26
7.23
7.23
7.23
7.22
7.22
7.20
7.20
7.19
7.05
7.04
7.04
7.03
6.92
6.91
6.40
6.39
6.32
6.32
5.47
5.47
5.46
5.46
4.71
4.63
4.63
4.61
4.59
4.18
4.15
3.81
3.78
3.47
3.42
2.82
1.37
0.94
Me
CO_2Me
CO_2Me
N
H
Bn
3ab
0.74
3.26
1.38
1.69
1.67
0.21
0.95
0.30
0.32
0.33
0.81
0.85
0.74
0.26
0.43
0.71
0.79
0.35
0.80
0.81
2.22
2.22
0.72
0.79
0.88
0.37
0.42
0.99
2.15
0.87
9.0
8.0
7.0
6.0
5.0
4.0
3.0
2.0
1.0

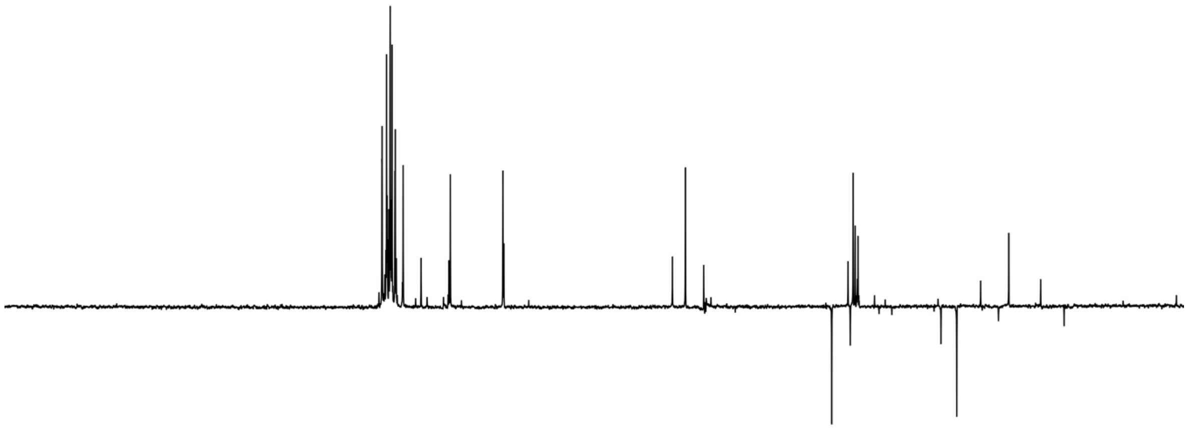

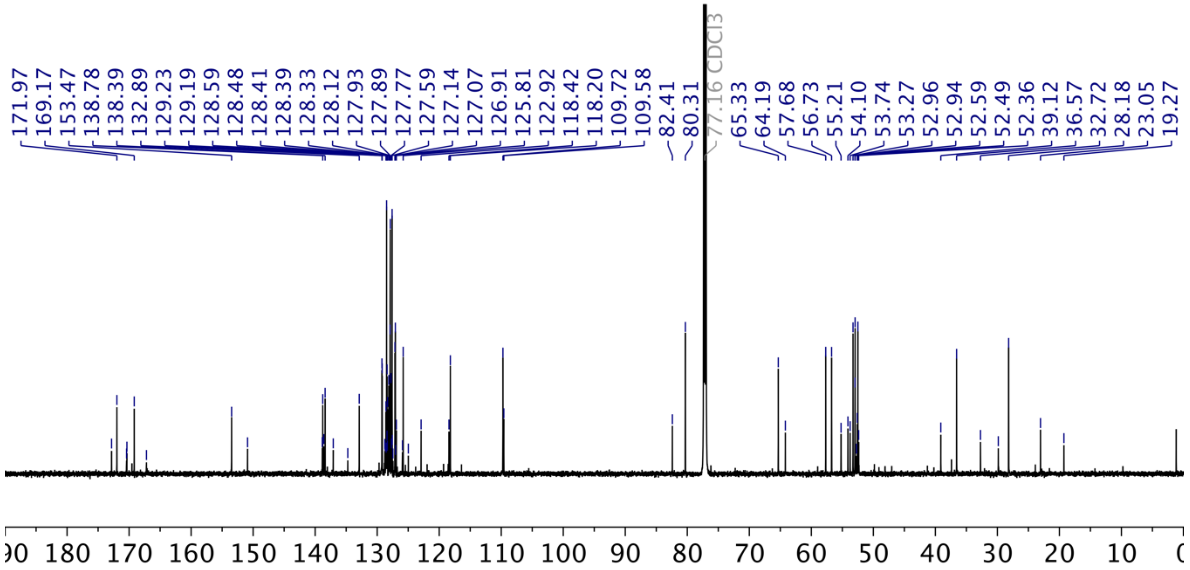
171.97
169.17
153.47
138.78
138.39
132.89
129.23
129.19
128.59
128.48
128.41
128.39
128.33
128.12
127.93
127.89
127.77
127.59
127.14
127.07
126.91
125.81
122.92
118.42
118.20
109.72
109.58
82.41
80.31
77.16 CDCl3
65.33
64.19
57.68
56.73
55.21
54.10
53.74
53.27
52.96
52.94
52.59
52.49
52.36
39.12
36.57
32.72
28.18
23.05
19.27
180
170
160
150
140
130
120
110
100
90
80
70
60
50
40
30
20
10

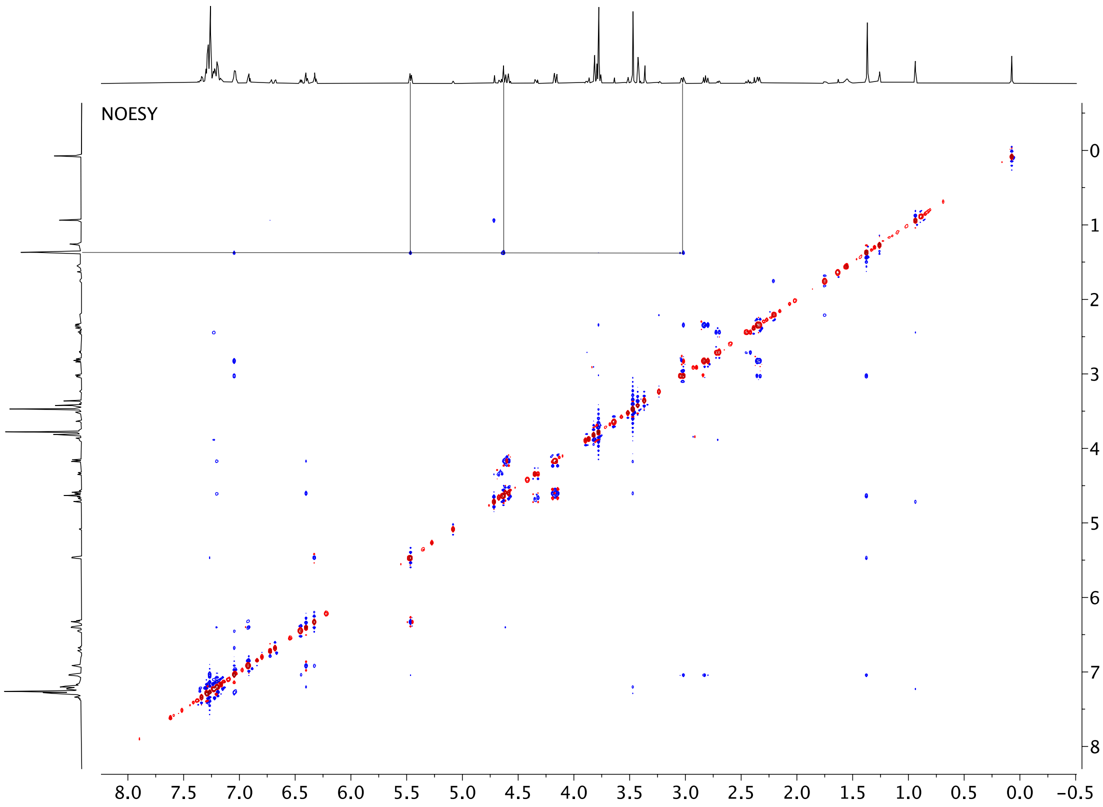
NOESY
8.0 7.5 7.0 6.5 6.0 5.5 5.0 4.5 4.0 3.5 3.0 2.5 2.0 1.5 1.0 0.5 0.0 −0.5
0 1 2 3 4 5 6 7 8

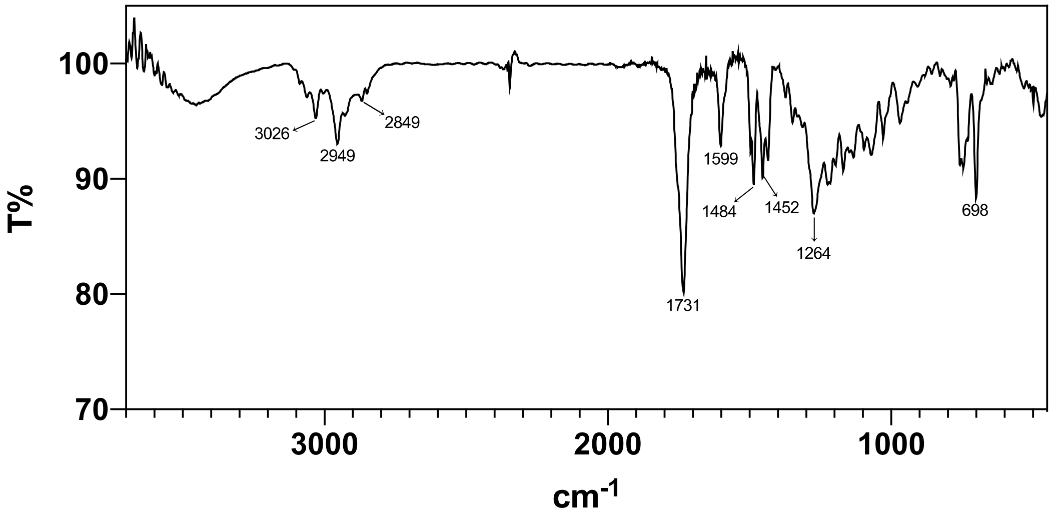
T%
100
90
80
70
3000
2000
1000
cm-1
3026
2949
2849
1731
1599
1484
1452
1264
698

Acquisition Parameter

Source Type	ESI	Ion Polarity	Positive	Set Corrector Fill	74 V
n/a	n/a	Set Capillary Exit	80.0 V	Set Pulsar Pull	799 V
Scan Begin	50 m/z	Set Hexapole RF	60.0 V	Set Pulsar Push	799 V
Scan End	3000 m/z	Set Skimmer 1	50.0 V	Set Reflector	1700 V
		Set Hexapole 1	24.3 V	Set Flight Tube	8600 V
				Set Detector TOF	2311 V

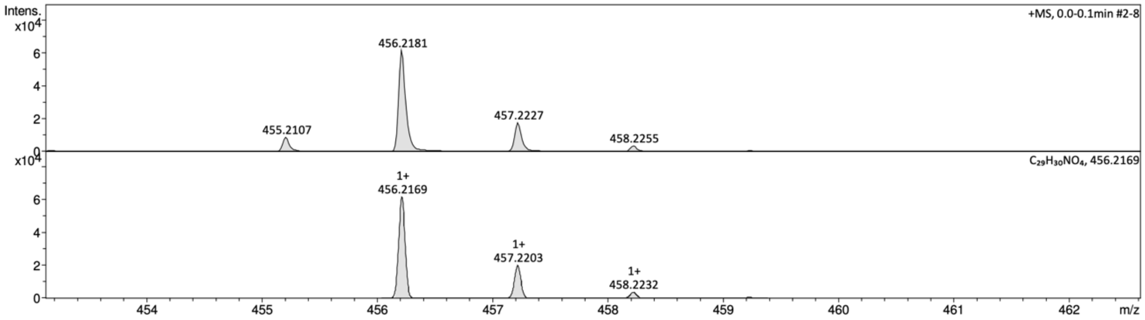

Meas. m/z	#	Ion Formula	m/z	err [ppm]	mSigma	# mSigma	Score	rdb	e⁻ Conf	N-Rule
456.2181	1	C29H30NO4	456.2169	-2.5	19.8	1	100.00	15.5	even	ok

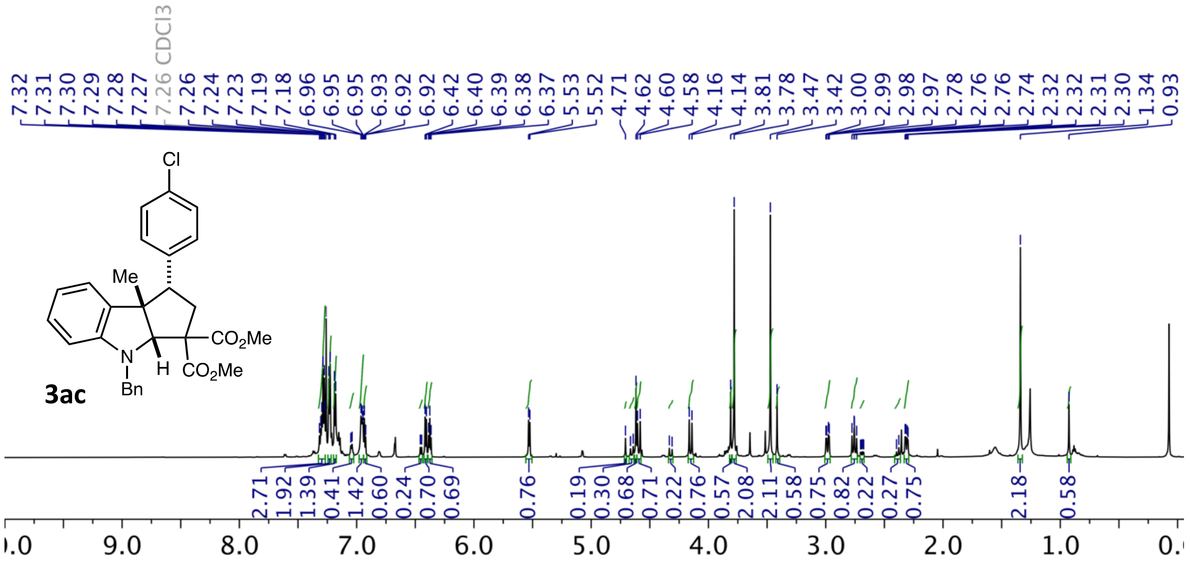
7.32 7.31 7.30 7.29 7.28 7.27 7.26 CDCl3 7.26 7.24 7.23 7.19 7.18 6.96 6.95 6.95 6.93 6.92 6.92 6.42 6.40 6.39 6.38 6.37 5.53 5.52 4.71 4.62 4.60 4.58 4.16 4.14 3.81 3.78 3.47 3.42 3.00 2.99 2.98 2.97 2.78 2.76 2.76 2.74 2.32 2.32 2.31 2.30 1.34 0.93
Cl
Me
CO2Me
CO2Me
N
H
Bn
3ac
2.71 1.92 1.39 0.41 1.42 0.60 0.24 0.70 0.69 0.76 0.19 0.30 0.68 0.71 0.22 0.76 0.57 2.08 2.11 0.58 0.75 0.82 0.22 0.27 0.75 2.18 0.58
10.0 9.0 8.0 7.0 6.0 5.0 4.0 3.0 2.0 1.0 0.0

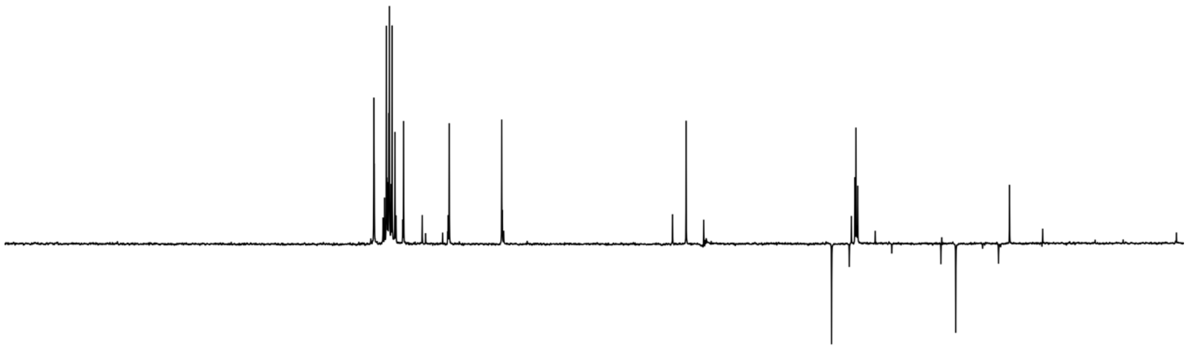

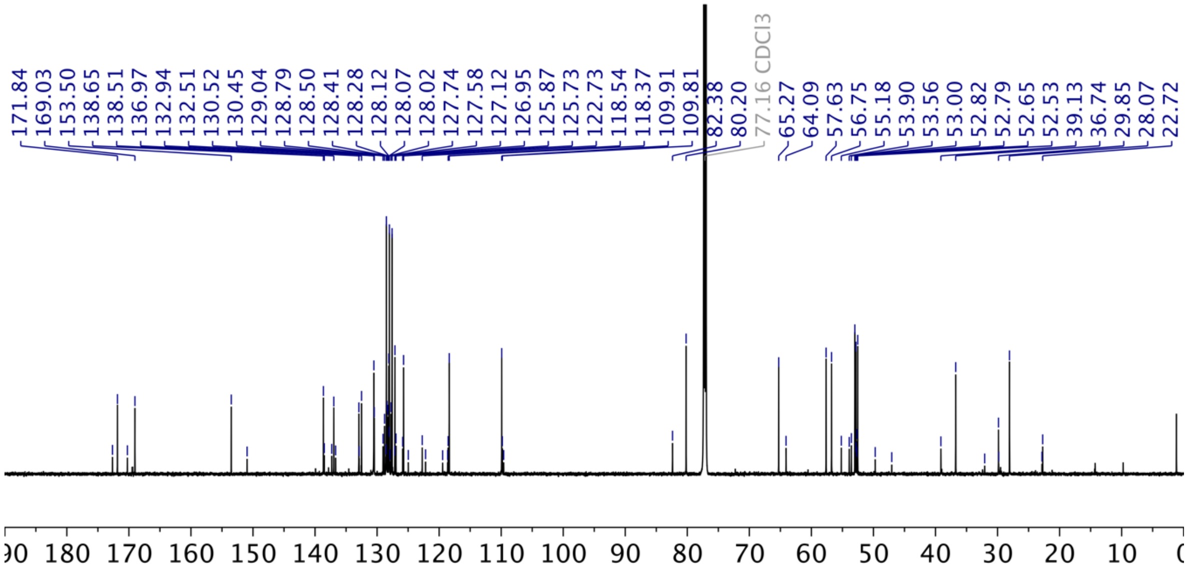
171.84 169.03 153.50 138.65 138.51 136.97 132.94 132.51 130.52 130.45 129.04 128.79 128.50 128.41 128.28 128.12 128.07 128.02 127.74 127.58 127.12 126.95 125.87 125.73 122.73 118.54 118.37 109.91 109.81 82.38 80.20 77.16 CDCl3 65.27 64.09 57.63 56.75 55.18 53.90 53.56 53.00 52.82 52.79 52.65 52.53 39.13 36.74 29.85 28.07 22.72
190 180 170 160 150 140 130 120 110 100 90 80 70 60 50 40 30 20 10 0

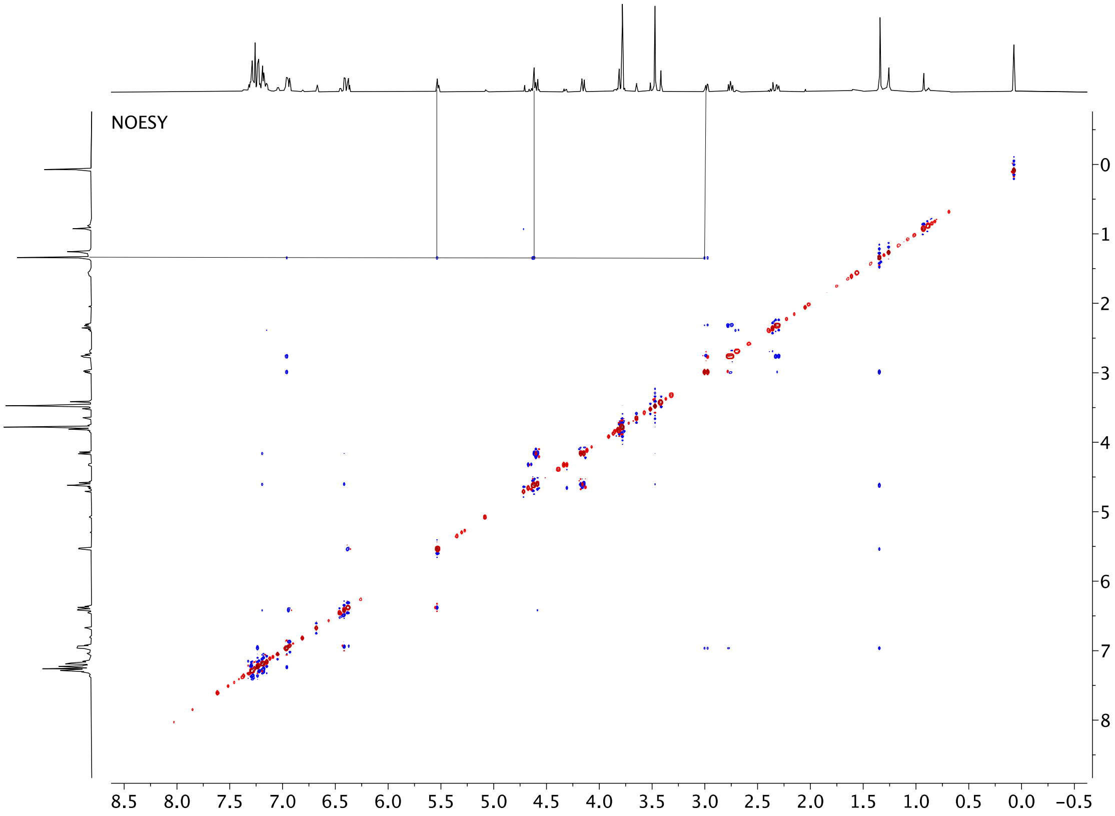
NOESY
8.5 8.0 7.5 7.0 6.5 6.0 5.5 5.0 4.5 4.0 3.5 3.0 2.5 2.0 1.5 1.0 0.5 0.0 −0.5
0 1 2 3 4 5 6 7 8

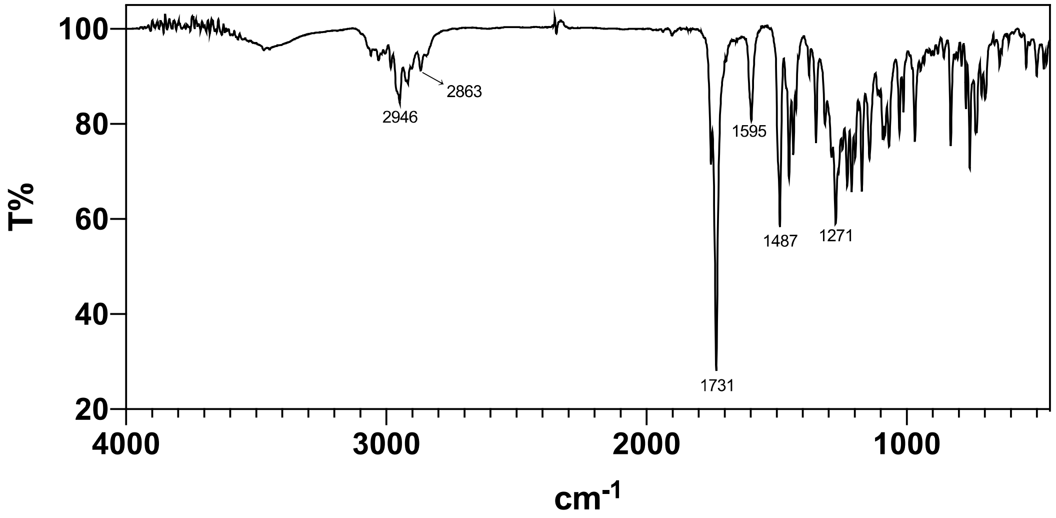
T%
100
80
60
40
20
4000 3000 2000 1000
cm-1
2946
2863
1731
1595
1487
1271

Acquisition Parameter

				Set Corrector Fill	74 V
Source Type	ESI	Ion Polarity	Positive	Set Pulsar Pull	799 V
n/a	n/a	Set Capillary Exit	80.0 V	Set Pulsar Push	799 V
Scan Begin	50 m/z	Set Hexapole RF	60.0 V	Set Reflector	1700 V
Scan End	3000 m/z	Set Skimmer 1	50.0 V	Set Flight Tube	8600 V
		Set Hexapole 1	24.3 V	Set Detector TOF	2311 V

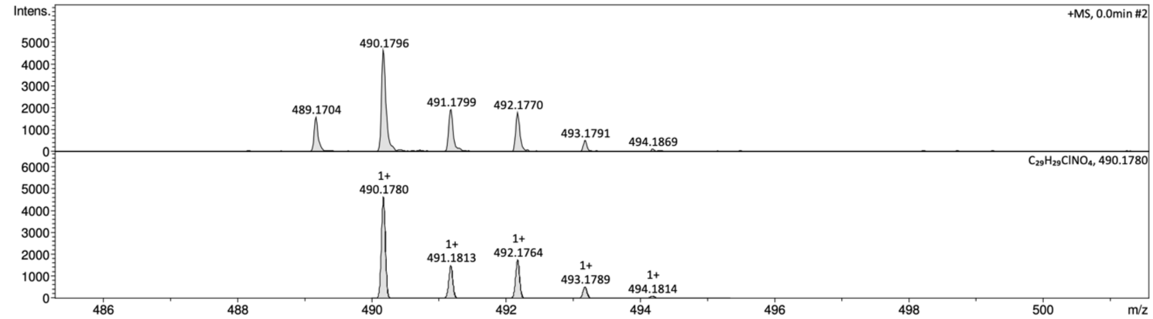

Meas. m/z	#	Ion Formula	m/z	err [ppm]	mSigma	# mSigma	Score	rdb	e⁻ Conf	N-Rule
490.1796	1	C29H29ClNO4	490.1780	-3.4	42.3	1	100.00	15.5	even	ok

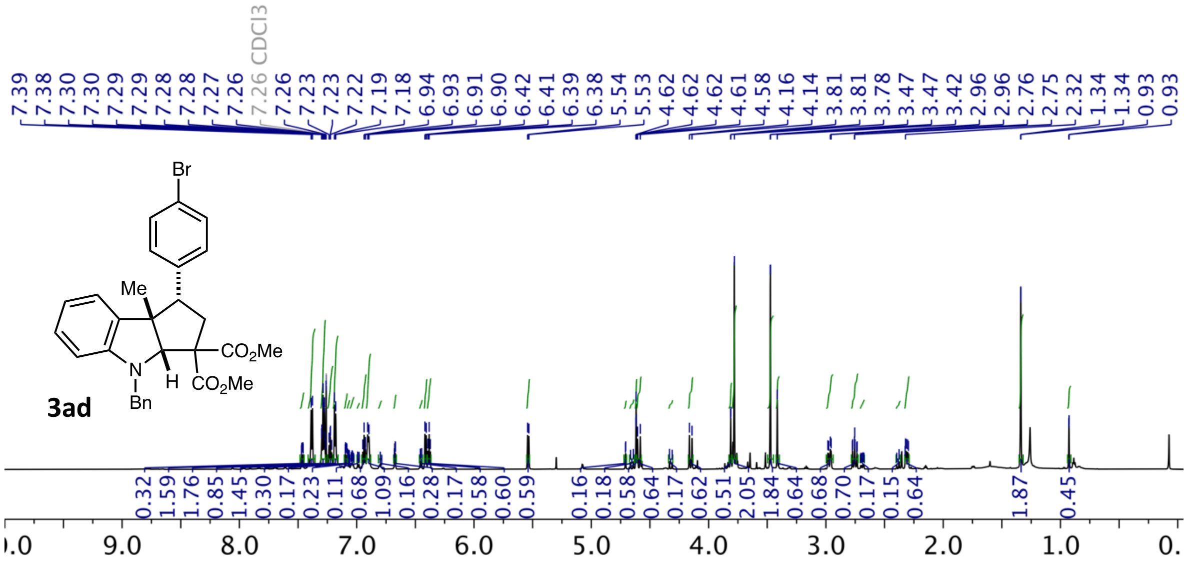
7.39 7.38 7.30 7.30 7.29 7.29 7.28 7.28 7.27 7.26 7.26 CDCl3 7.26 7.23 7.23 7.22 7.19 7.18 6.94 6.93 6.91 6.90 6.42 6.41 6.39 6.38 5.54 5.53 4.62 4.62 4.61 4.58 4.16 4.14 3.81 3.81 3.78 3.47 3.42 2.96 2.96 2.76 2.75 2.32 1.34 1.34 0.93 0.93
Br
Me
CO2Me
N
H
CO2Me
Bn
3ad
0.32 1.59 1.76 0.85 1.45 0.30 0.17 0.23 0.11 0.68 1.09 0.16 0.28 0.17 0.58 0.60 0.59 0.16 0.18 0.58 0.64 0.17 0.62 0.51 2.05 1.84 0.64 0.68 0.70 0.17 0.15 0.64 1.87 0.45
9.0 8.0 7.0 6.0 5.0 4.0 3.0 2.0 1.0

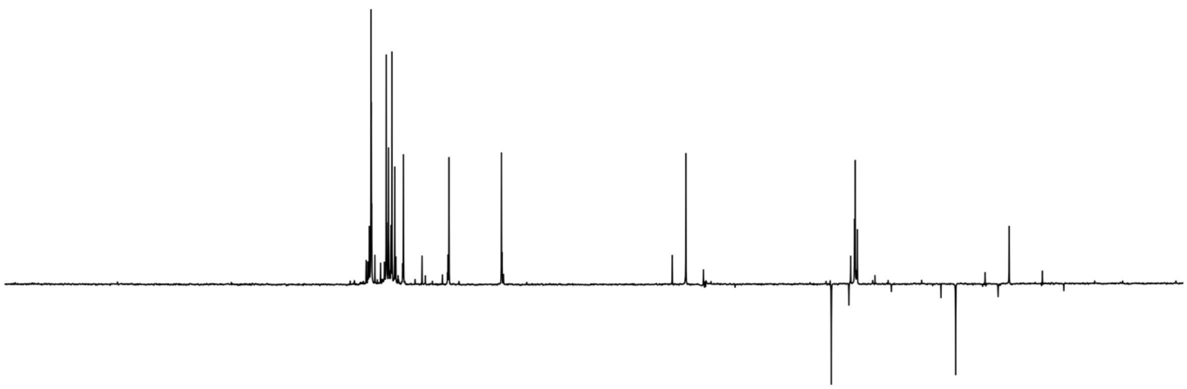

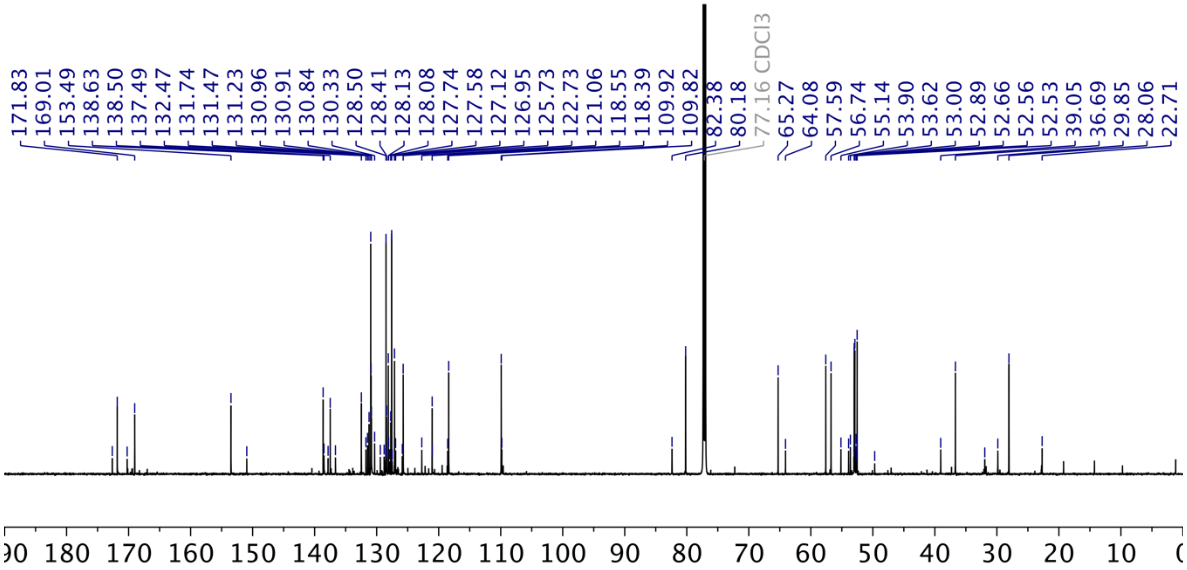
171.83 169.01 153.49 138.63 138.50 137.49 132.47 131.74 131.47 131.23 130.96 130.91 130.84 130.33 128.50 128.41 128.13 128.08 127.74 127.58 127.12 126.95 125.73 122.73 121.06 118.55 118.39 109.92 109.82 82.38 80.18 77.16 CDCl3 65.27 64.08 57.59 56.74 55.14 53.90 53.62 53.00 52.89 52.66 52.56 52.53 39.05 36.69 29.85 28.06 22.71
180 170 160 150 140 130 120 110 100 90 80 70 60 50 40 30 20 10

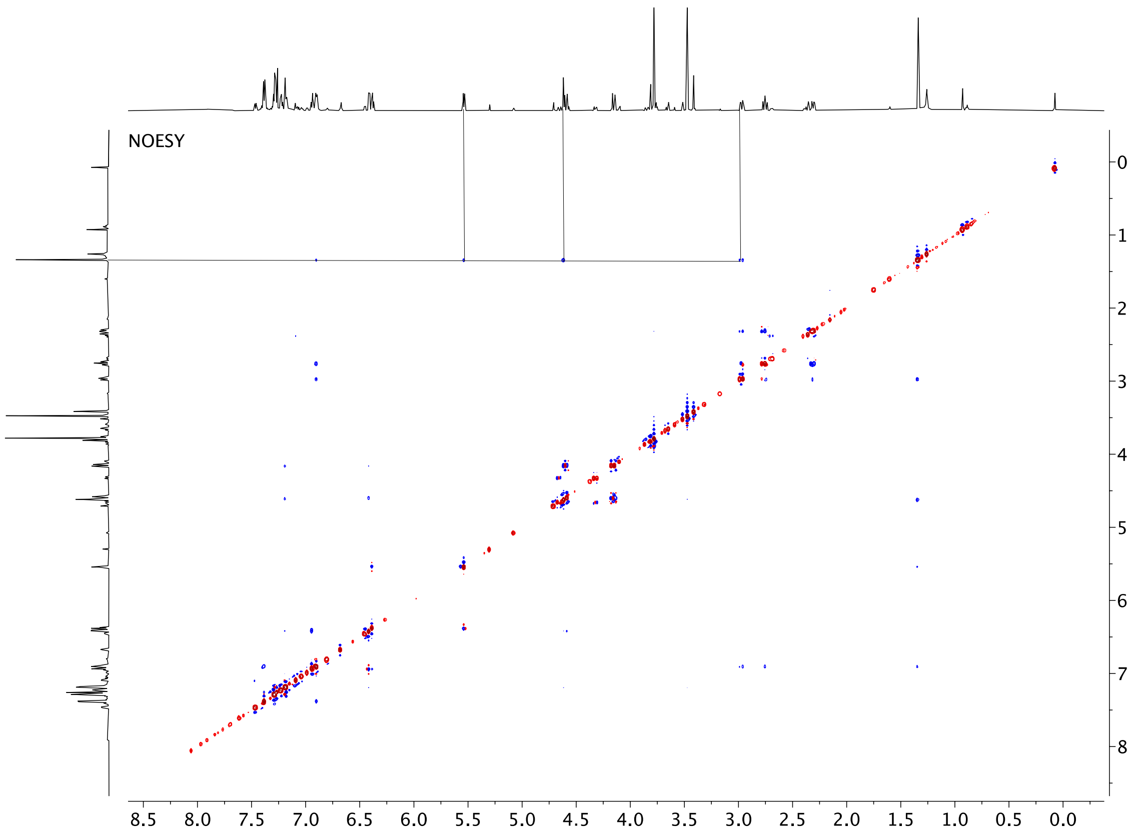
NOESY
8.5 8.0 7.5 7.0 6.5 6.0 5.5 5.0 4.5 4.0 3.5 3.0 2.5 2.0 1.5 1.0 0.5 0.0
0 1 2 3 4 5 6 7 8

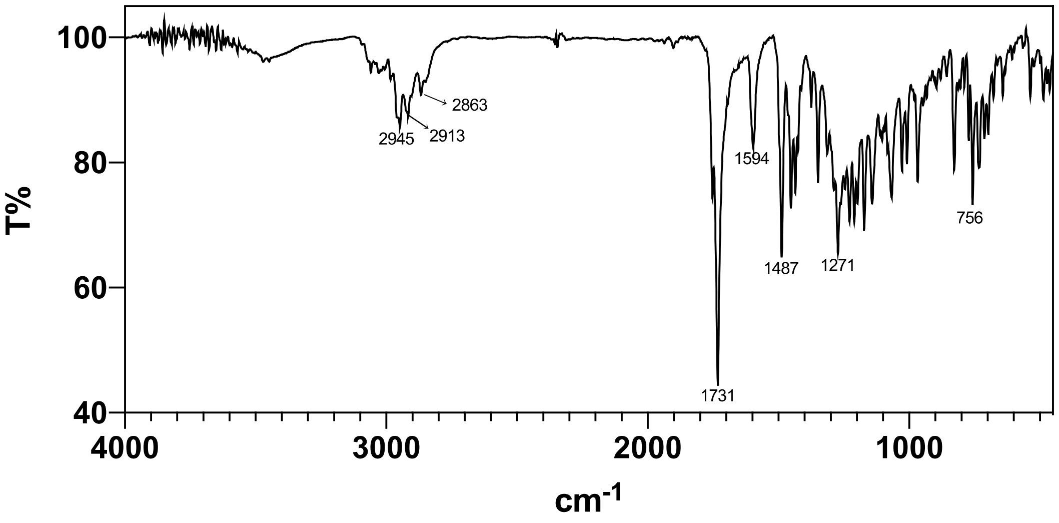
T%
100
80
60
40
4000 3000 2000 1000
cm-1
2945
2913
2863
1731
1594
1487
1271
756

Acquisition Parameter

Source Type	ESI	Ion Polarity	Positive	Set Corrector Fill	74 V
n/a	n/a	Set Capillary Exit	80.0 V	Set Pulsar Pull	799 V
Scan Begin	50 m/z	Set Hexapole RF	60.0 V	Set Pulsar Push	799 V
Scan End	3000 m/z	Set Skimmer 1	50.0 V	Set Reflector	1700 V
		Set Hexapole 1	24.3 V	Set Flight Tube	8600 V
				Set Detector TOF	2311 V

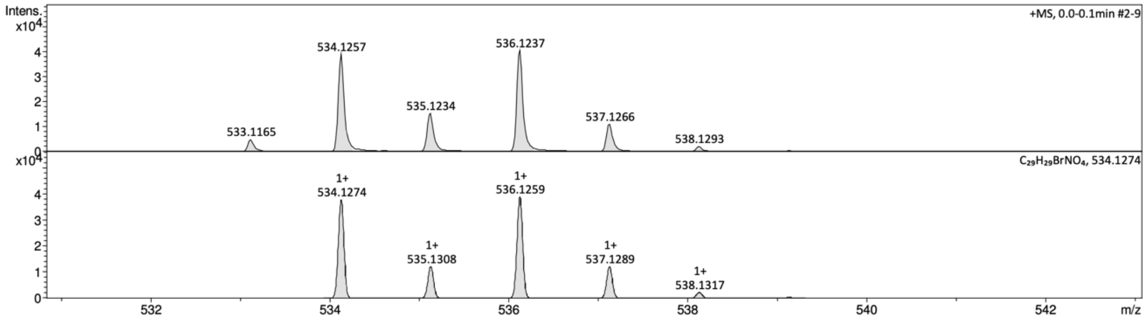

Meas. m/z	#	Ion Formula	m/z	err [ppm]	mSigma	# mSigma	Score	rdb	e⁻ Conf	N-Rule
534.1257	1	C29H29BrNO4	534.1274	3.2	35.3	1	100.00	15.5	even	ok

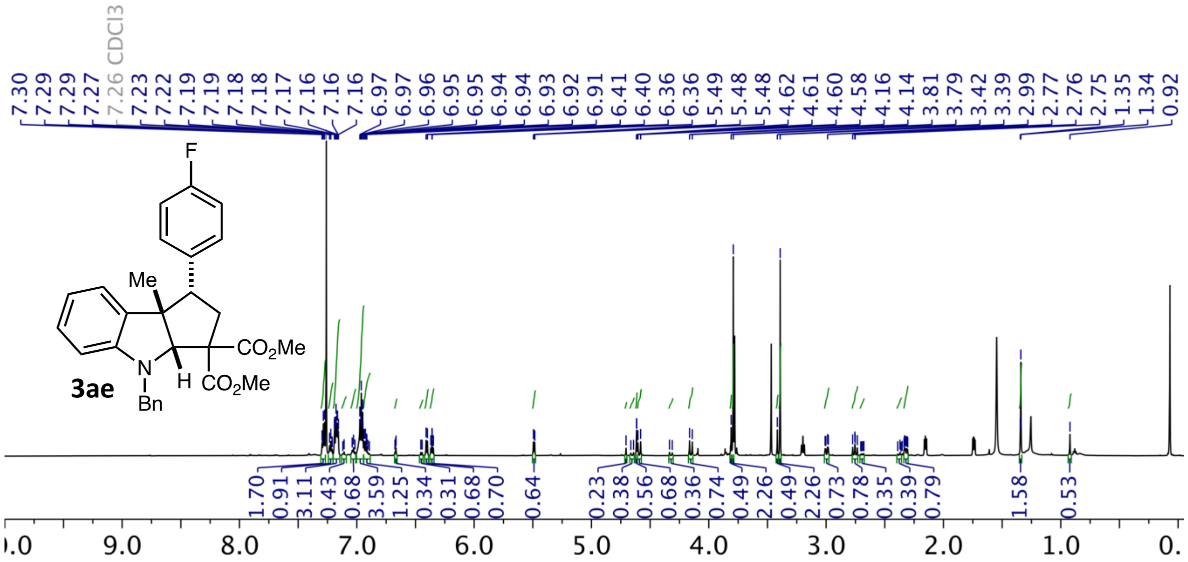
7.30 7.29 7.29 7.27 7.26 CDCl3 7.23 7.22 7.19 7.19 7.18 7.18 7.17 7.16 7.16 7.16 6.97 6.97 6.96 6.95 6.94 6.94 6.93 6.92 6.91 6.41 6.40 6.36 6.36 5.49 5.48 5.48 4.62 4.61 4.60 4.58 4.16 4.14 3.81 3.79 3.42 3.39 2.99 2.77 2.76 2.75 1.35 1.34 0.92
F
Me
CO_2Me
N
H
CO_2Me
Bn
3ae
1.70 0.91 3.11 0.43 0.68 3.59 1.25 0.34 0.31 0.68 0.70 0.64 0.23 0.38 0.56 0.68 0.36 0.74 0.49 2.26 0.49 2.26 0.73 0.78 0.35 0.39 0.79 1.58 0.53
9.0 8.0 7.0 6.0 5.0 4.0 3.0 2.0 1.0

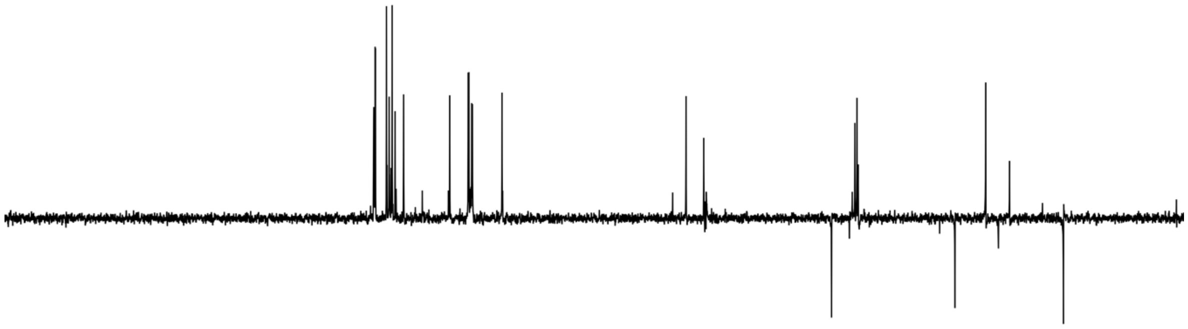

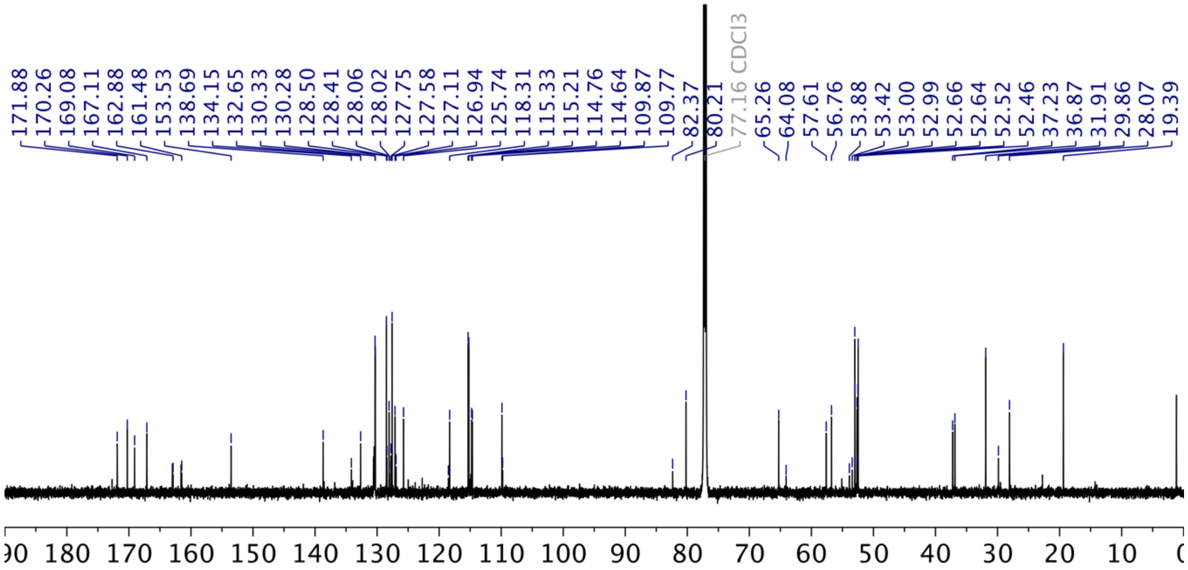
171.88 170.26 169.08 167.11 162.88 161.48 153.53 138.69 134.15 132.65 130.33 130.28 128.50 128.41 128.06 128.02 127.75 127.58 127.11 126.94 125.74 118.31 115.33 115.21 114.76 114.64 109.87 109.77 82.37 80.21 77.16 CDCl3 65.26 64.08 57.61 56.76 53.88 53.42 53.00 52.99 52.66 52.64 52.52 52.46 37.23 36.87 31.91 29.86 28.07 19.39
180 170 160 150 140 130 120 110 100 90 80 70 60 50 40 30 20 10

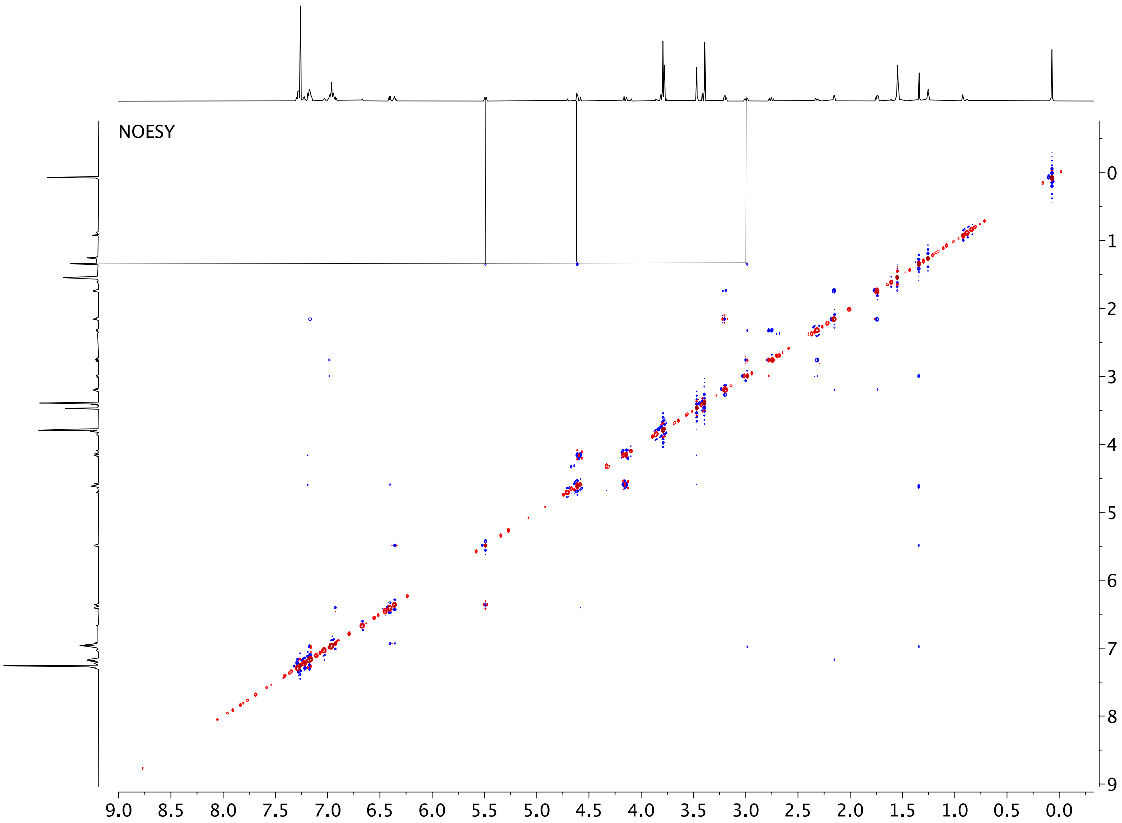
NOESY

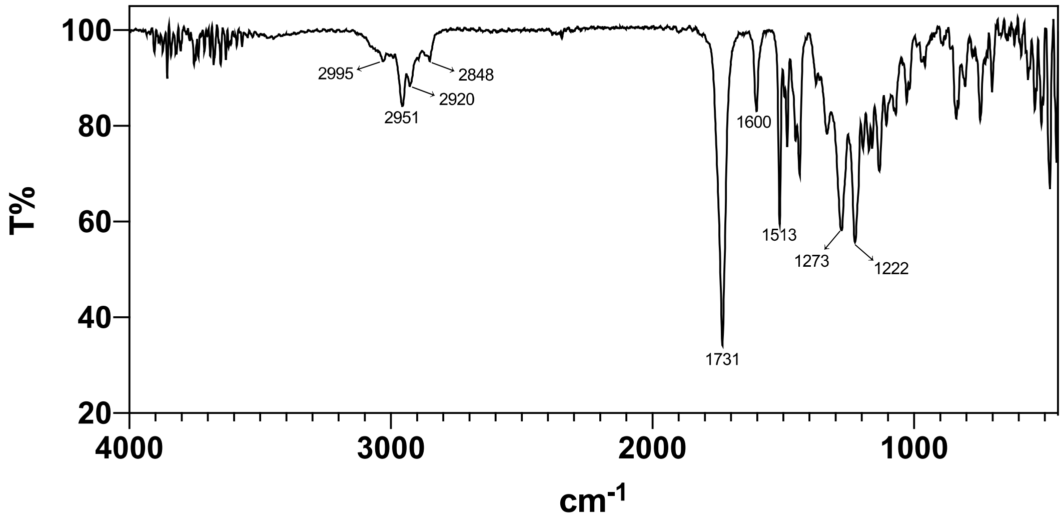
T%
cm-1
2995
2951
2920
2848
1731
1600
1513
1273
1222

Acquisition Parameter

Source Type	ESI	Ion Polarity	Positive	Set Corrector Fill	74 V
n/a	n/a	Set Capillary Exit	80.0 V	Set Pulsar Pull	799 V
Scan Begin	50 m/z	Set Hexapole RF	60.0 V	Set Pulsar Push	799 V
Scan End	3000 m/z	Set Skimmer 1	50.0 V	Set Reflector	1700 V
		Set Hexapole 1	24.3 V	Set Flight Tube	8600 V
				Set Detector TOF	2311 V

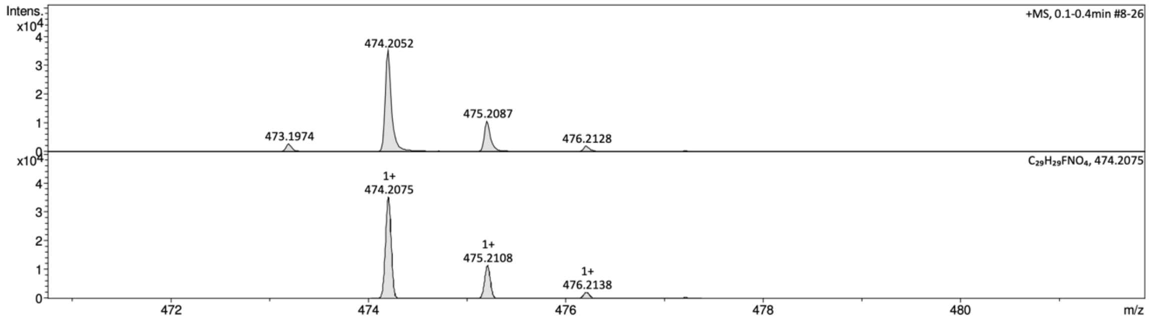

Meas. m/z	#	Ion Formula	m/z	err [ppm]	mSigma	# mSigma	Score	rdb	e⁻ Conf	N-Rule
474.2052	1	C29H29FNO4	474.2075	5.0	9.3	1	100.00	15.5	even	ok

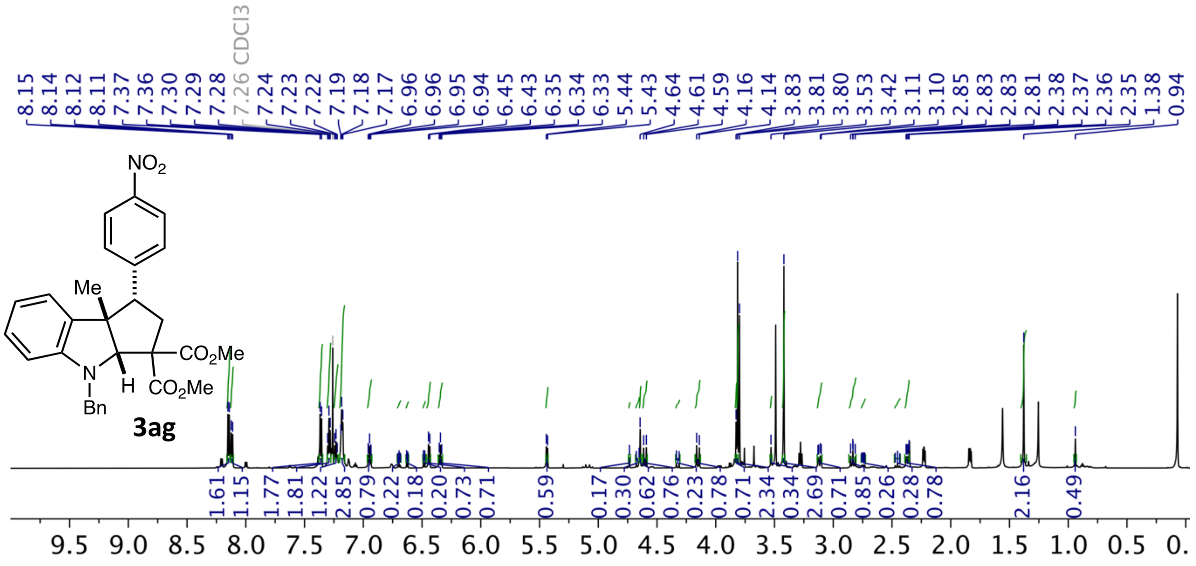
8.15 8.14 8.12 8.11 7.37 7.36 7.30 7.29 7.28 7.26 CDCl3 7.24 7.23 7.22 7.19 7.18 7.17 6.96 6.96 6.95 6.94 6.45 6.43 6.35 6.34 6.33 5.44 5.43 4.64 4.61 4.59 4.16 4.14 3.83 3.81 3.80 3.53 3.42 3.11 3.10 2.85 2.83 2.83 2.81 2.38 2.37 2.36 2.35 1.38 0.94
NO2
Me
CO2Me
CO2Me
N
H
Bn
3ag
1.61 1.15 1.77 1.81 1.22 2.85 0.79 0.22 0.18 0.20 0.73 0.71 0.59 0.17 0.30 0.62 0.76 0.23 0.78 0.71 2.34 0.34 2.69 0.71 0.85 0.26 0.28 0.78 2.16 0.49
9.5 9.0 8.5 8.0 7.5 7.0 6.5 6.0 5.5 5.0 4.5 4.0 3.5 3.0 2.5 2.0 1.5 1.0 0.5 0.

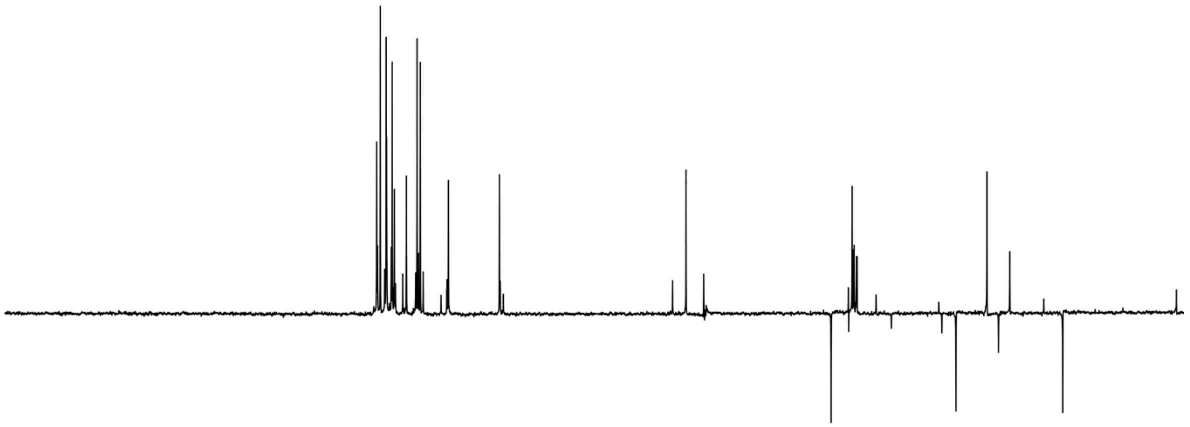

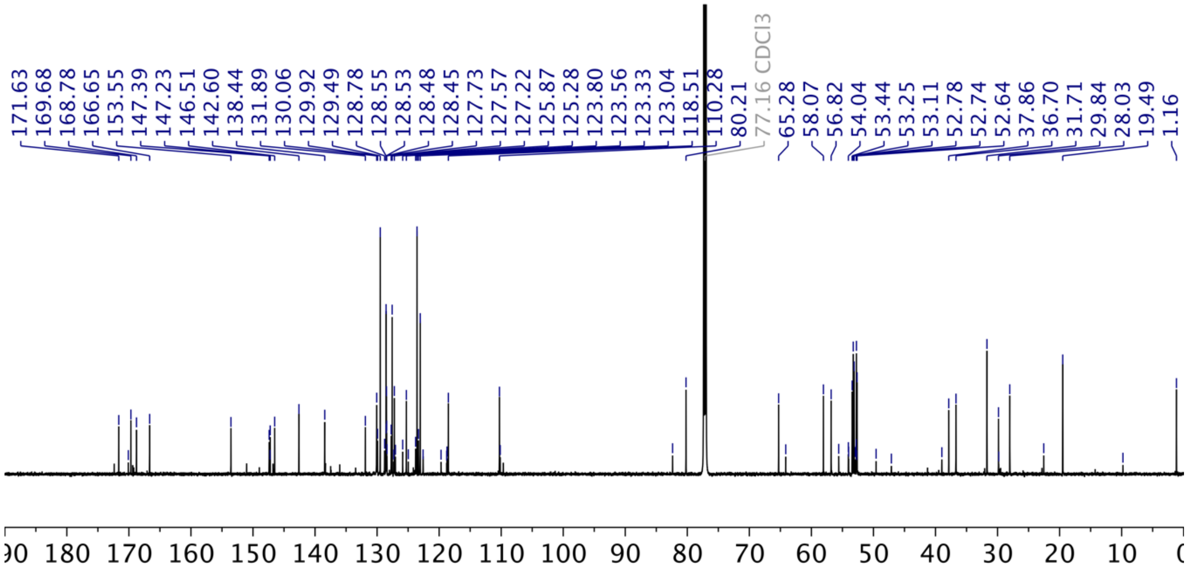
171.63 169.68 168.78 166.65 153.55 147.39 147.23 146.51 142.60 138.44 131.89 130.06 129.92 129.49 128.78 128.55 128.53 128.48 128.45 127.73 127.57 127.22 125.87 125.28 123.80 123.56 123.33 123.04 118.51 110.28 80.21 77.16 CDCl3 65.28 58.07 56.82 54.04 53.44 53.25 53.11 52.78 52.74 52.64 37.86 36.70 31.71 29.84 28.03 19.49 1.16
90 180 170 160 150 140 130 120 110 100 90 80 70 60 50 40 30 20 10 0

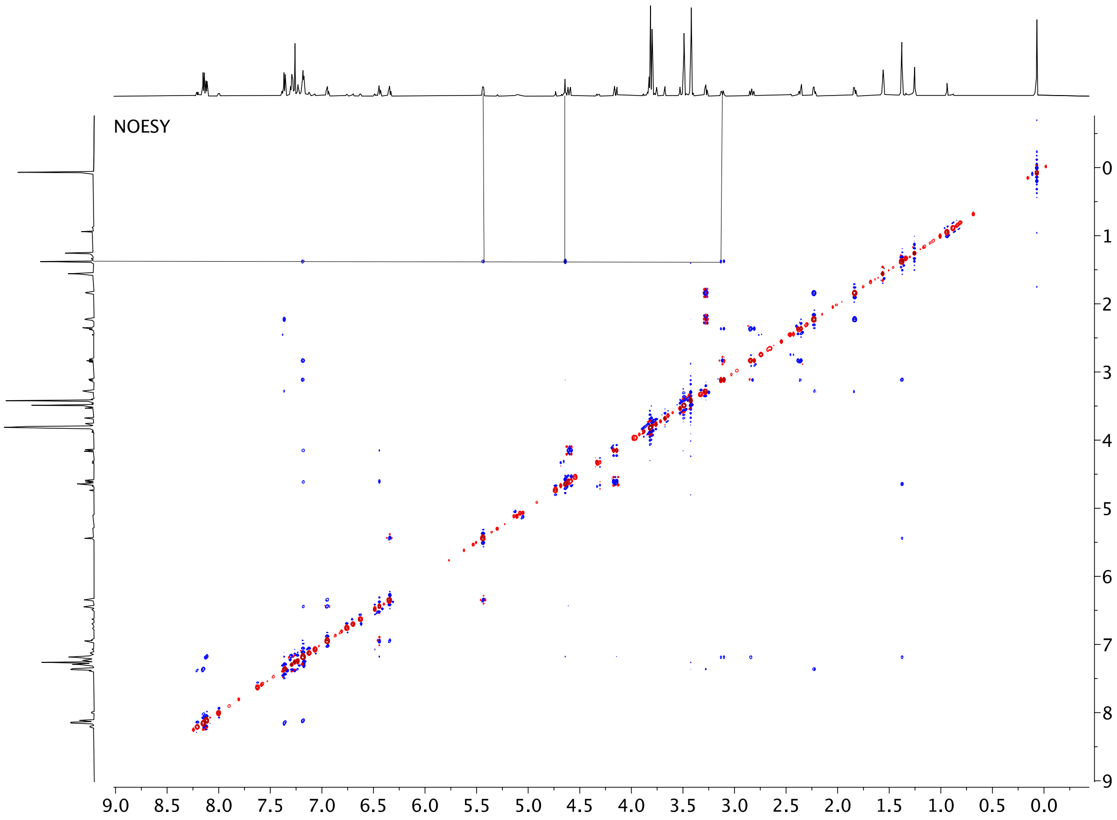
NOESY
9.0 8.5 8.0 7.5 7.0 6.5 6.0 5.5 5.0 4.5 4.0 3.5 3.0 2.5 2.0 1.5 1.0 0.5 0.0
0 1 2 3 4 5 6 7 8 9

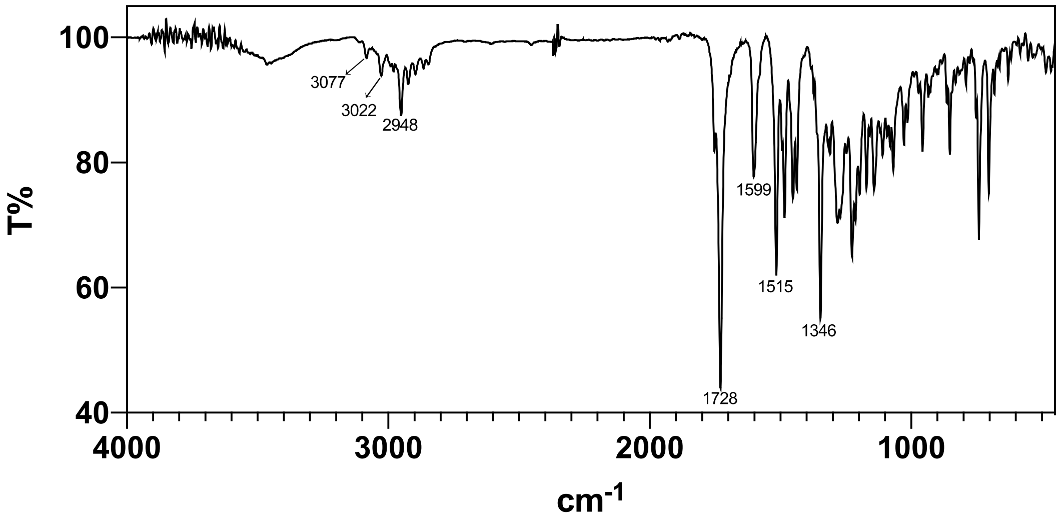
T%
100
80
60
40
3077
3022
2948
1599
1515
1346
1728
4000 3000 2000 1000
cm-1

Acquisition Parameter					
Source Type	ESI	Ion Polarity	Positive	Set Corrector Fill	74 V
n/a	n/a	Set Capillary Exit	80.0 V	Set Pulsar Pull	799 V
Scan Begin	50 m/z	Set Hexapole RF	60.0 V	Set Pulsar Push	799 V
Scan End	3000 m/z	Set Skimmer 1	50.0 V	Set Reflector	1700 V
		Set Hexapole 1	24.3 V	Set Flight Tube	8600 V
				Set Detector TOF	2311 V

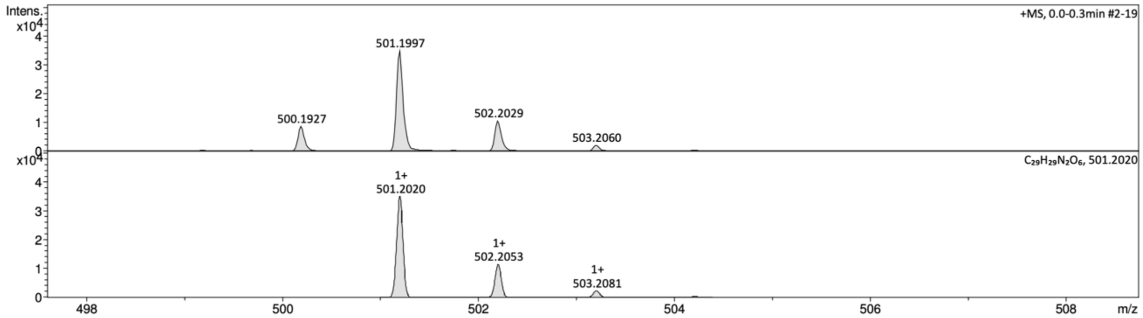

Meas. m/z	#	Ion Formula	m/z	err [ppm]	mSigma	# mSigma	Score	rdb	e⁻ Conf	N-Rule
501.1997	1	C29H29N2O6	501.2020	4.5	12.1	1	100.00	16.5	even	ok

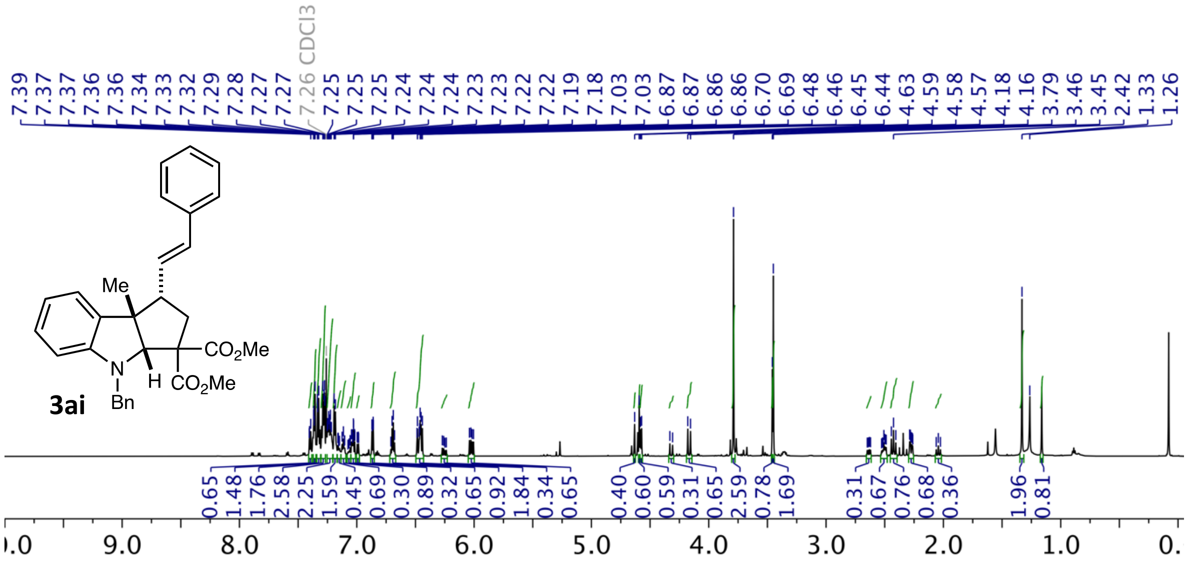
7.39 7.37 7.37 7.36 7.36 7.34 7.33 7.32 7.29 7.28 7.27 7.27 7.26 CDCl3 7.25 7.25 7.25 7.24 7.24 7.24 7.23 7.23 7.22 7.22 7.19 7.18 7.03 6.87 6.87 6.86 6.86 6.70 6.69 6.48 6.46 6.45 6.44 4.63 4.59 4.58 4.57 4.18 4.16 3.79 3.46 3.45 2.42 1.33 1.26
Me
CO_2Me
N
H
CO_2Me
Bn
3ai
0.65 1.48 1.76 2.58 2.25 1.59 0.45 0.69 0.30 0.89 0.32 0.65 0.92 1.84 0.34 0.65 0.40 0.60 0.59 0.31 0.65 2.59 0.78 1.69 0.31 0.67 0.76 0.68 0.36 1.96 0.81
9.0 8.0 7.0 6.0 5.0 4.0 3.0 2.0 1.0

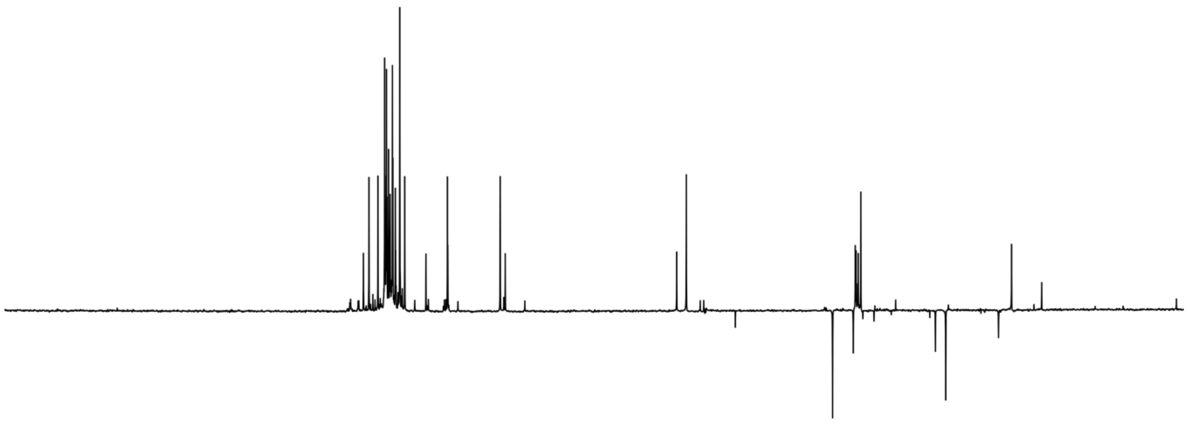

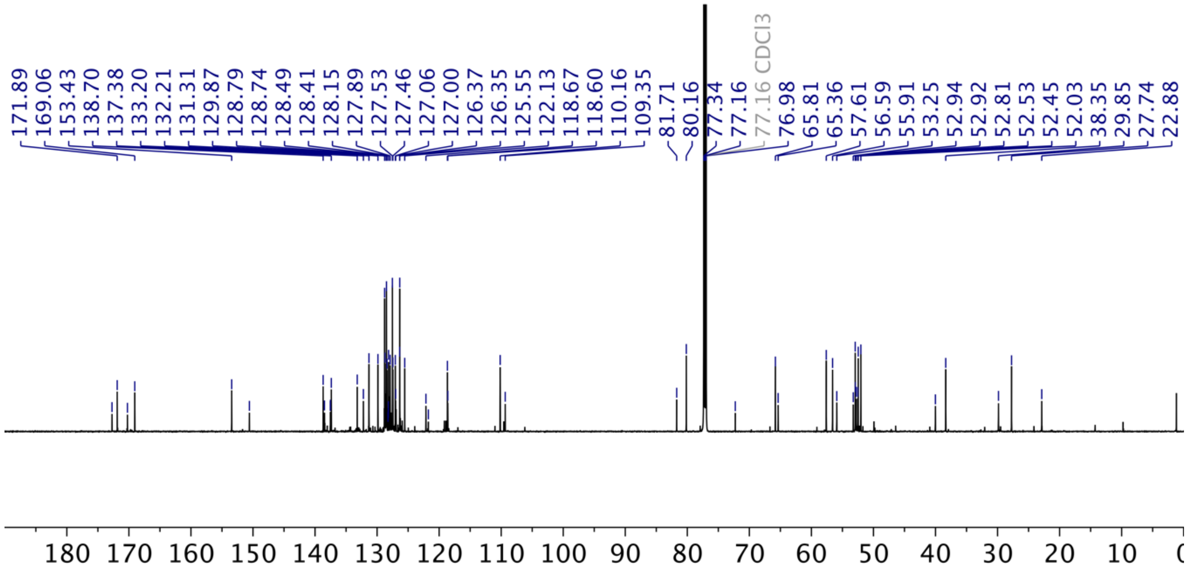
171.89 169.06 153.43 138.70 137.38 133.20 132.21 131.31 129.87 128.79 128.74 128.49 128.41 128.15 127.89 127.53 127.46 127.06 127.00 126.37 126.35 125.55 122.13 118.67 118.60 110.16 109.35 81.71 80.16 77.34 77.16 77.16 CDCl3 76.98 65.81 65.36 57.61 56.59 55.91 53.25 52.94 52.92 52.81 52.53 52.45 52.03 38.35 29.85 27.74 22.88
180 170 160 150 140 130 120 110 100 90 80 70 60 50 40 30 20 10

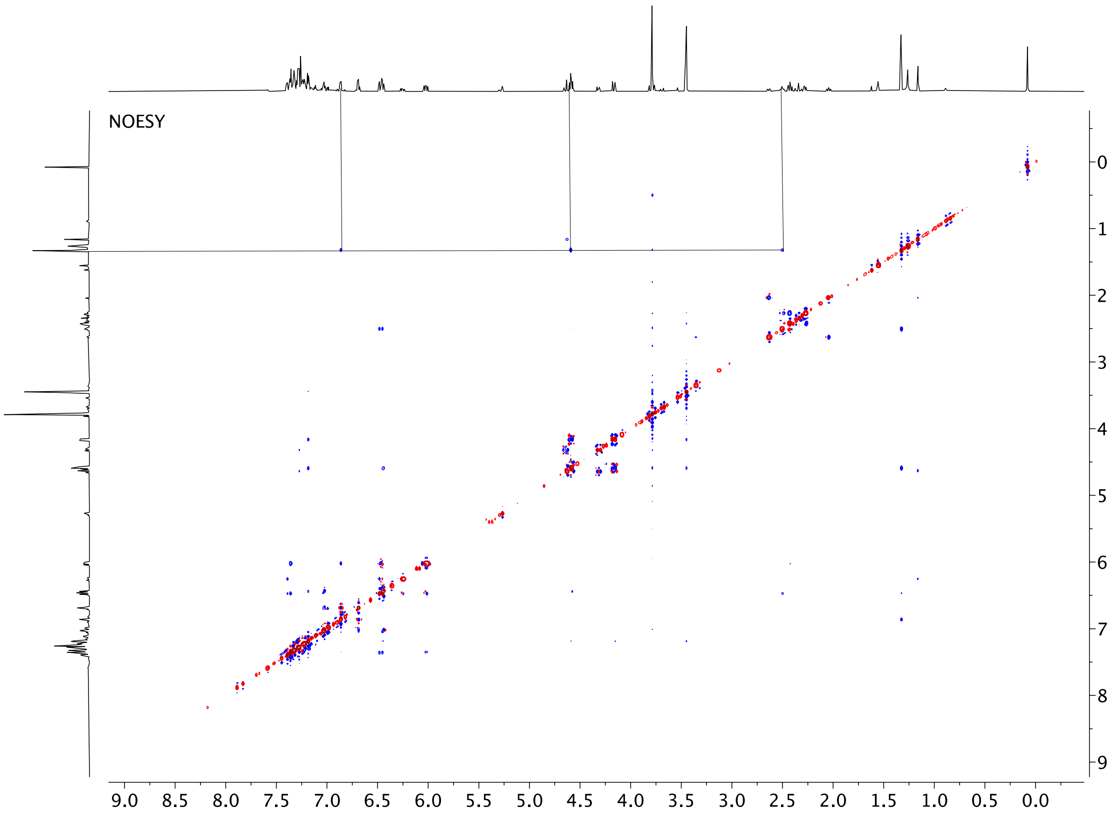
NOESY
9.0 8.5 8.0 7.5 7.0 6.5 6.0 5.5 5.0 4.5 4.0 3.5 3.0 2.5 2.0 1.5 1.0 0.5 0.0
0 1 2 3 4 5 6 7 8 9

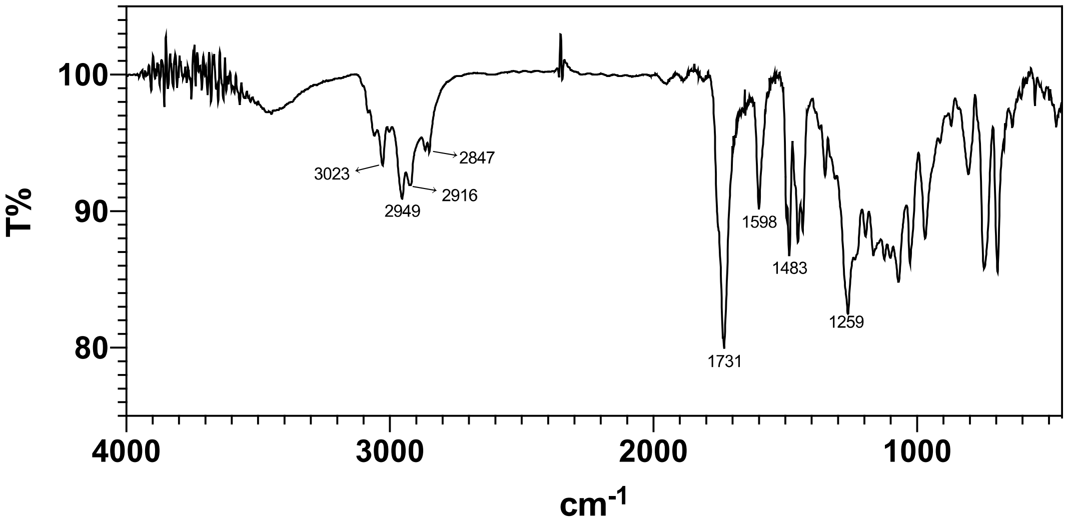
T%
100
90
80
3023
2949
2916
2847
1731
1598
1483
1259
4000
3000
2000
1000
cm-1

Acquisition Parameter

Source Type	ESI	Ion Polarity	Positive	Set Corrector Fill	74 V
n/a	n/a	Set Capillary Exit	80.0 V	Set Pulsar Pull	799 V
Scan Begin	50 m/z	Set Hexapole RF	60.0 V	Set Pulsar Push	799 V
Scan End	3000 m/z	Set Skimmer 1	50.0 V	Set Reflector	1700 V
		Set Hexapole 1	24.3 V	Set Flight Tube	8600 V
				Set Detector TOF	2311 V

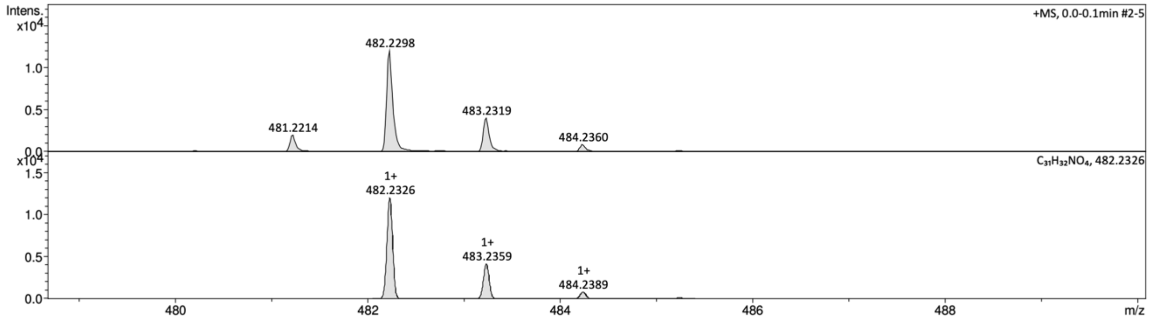

Meas. m/z	#	Ion Formula	m/z	err [ppm]	mSigma	# mSigma	Score	rdb	e⁻ Conf	N-Rule
482.2298	1	C31H32NO4	482.2326	5.7	6.4	1	100.00	16.5	even	ok

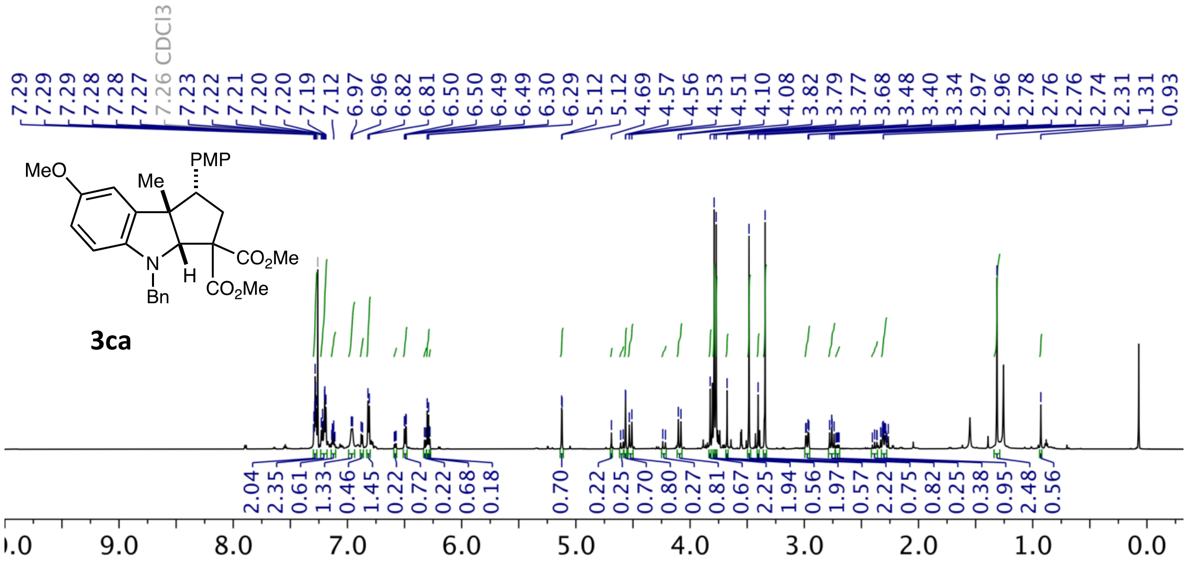
7.29 7.29 7.29 7.28 7.28 7.27 7.26 CDCl3 7.23 7.22 7.21 7.20 7.20 7.19 7.12 6.97 6.96 6.82 6.81 6.50 6.50 6.49 6.49 6.30 6.29 5.12 5.12 4.69 4.57 4.56 4.53 4.51 4.10 4.08 3.82 3.79 3.77 3.68 3.48 3.40 3.34 2.97 2.96 2.78 2.76 2.74 2.31 1.31 0.93
MeO
Me
PMP
CO2Me
N
H
CO2Me
Bn
3ca
2.04 2.35 0.61 1.33 0.46 1.45 0.22 0.72 0.22 0.68 0.18 0.70 0.22 0.25 0.70 0.80 0.27 0.81 0.67 2.25 1.94 0.56 1.97 0.57 2.22 0.75 0.82 0.25 0.38 0.95 2.48 0.56
9.0 8.0 7.0 6.0 5.0 4.0 3.0 2.0 1.0 0.0

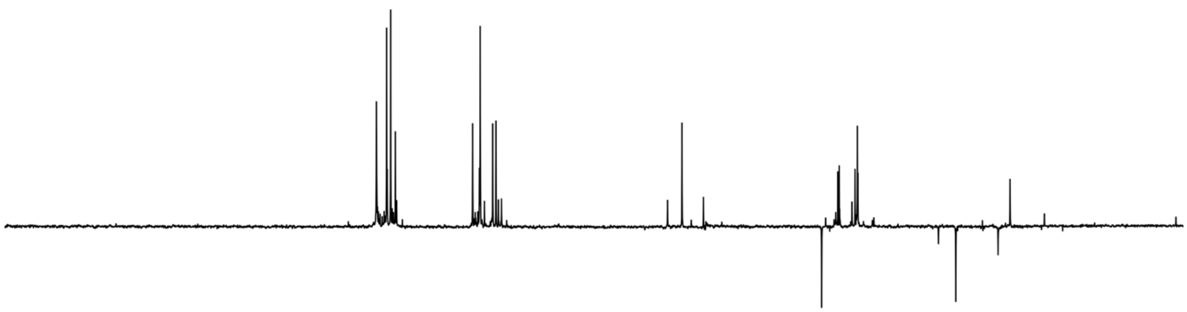

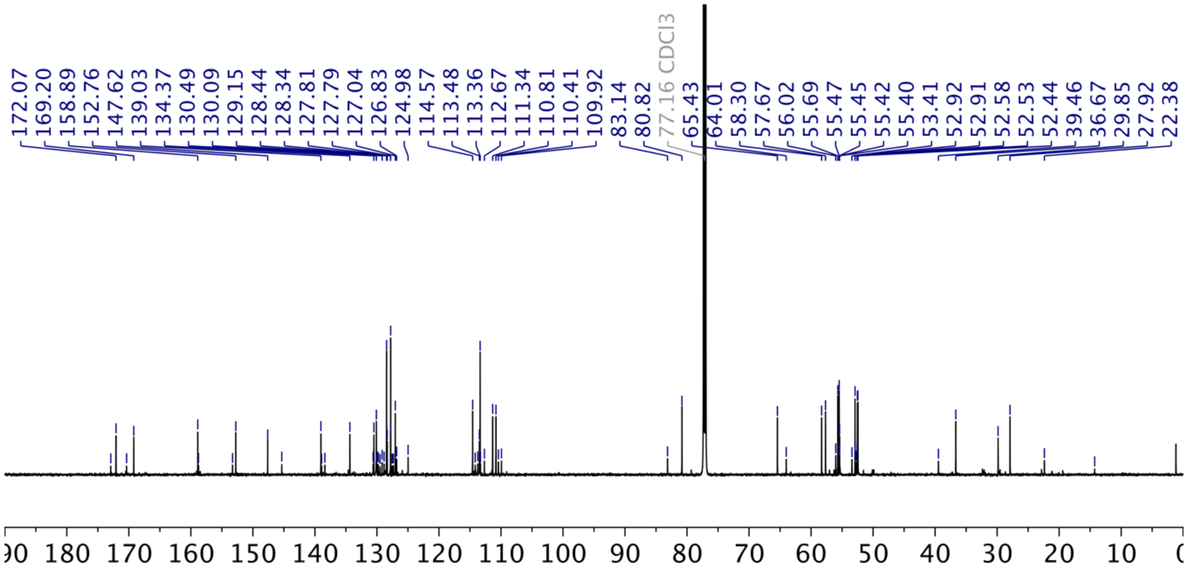
172.07 169.20 158.89 152.76 147.62 139.03 134.37 130.49 130.09 129.15 128.44 128.34 127.81 127.79 127.04 126.83 124.98 114.57 113.48 113.36 112.67 111.34 110.81 110.41 109.92 83.14 80.82 77.16 CDCl3 65.43 64.01 58.30 57.67 56.02 55.69 55.47 55.45 55.42 55.40 53.41 52.92 52.91 52.58 52.53 52.44 39.46 36.67 29.85 27.92 22.38
180 170 160 150 140 130 120 110 100 90 80 70 60 50 40 30 20 10

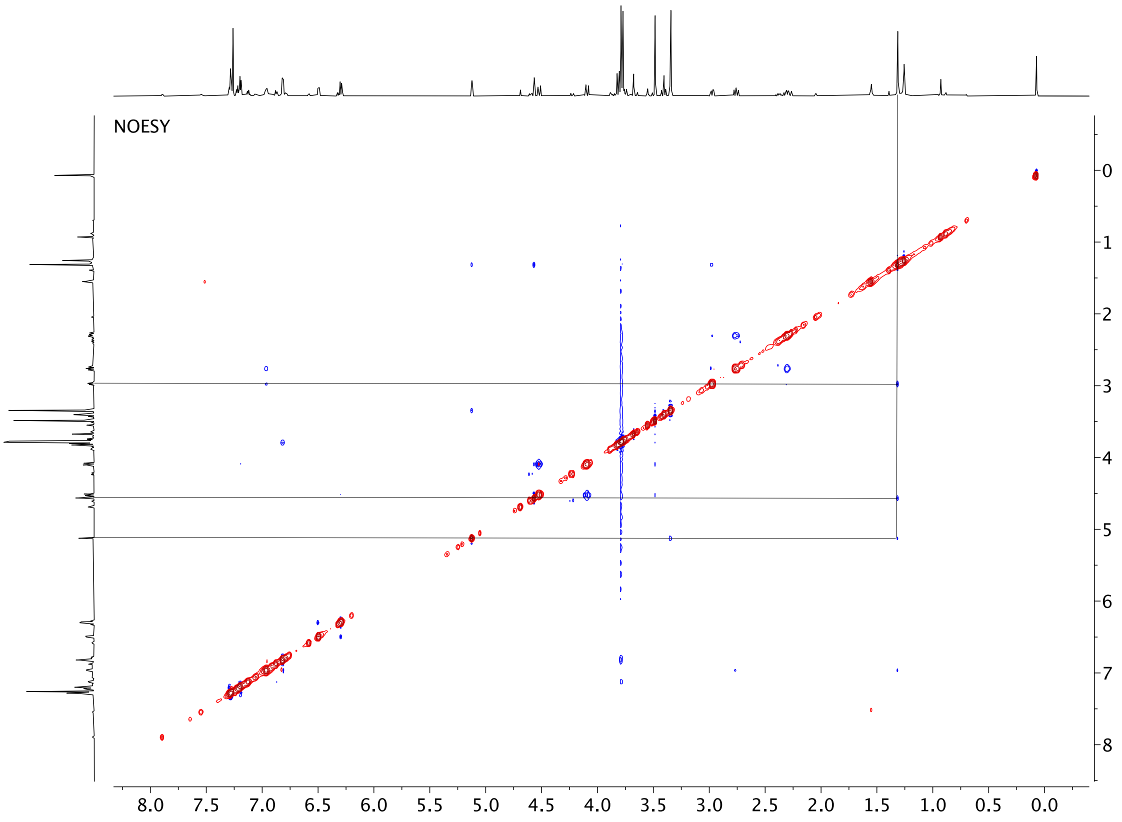
NOESY
8.0
7.5
7.0
6.5
6.0
5.5
5.0
4.5
4.0
3.5
3.0
2.5
2.0
1.5
1.0
0.5
0.0
0
1
2
3
4
5
6
7
8

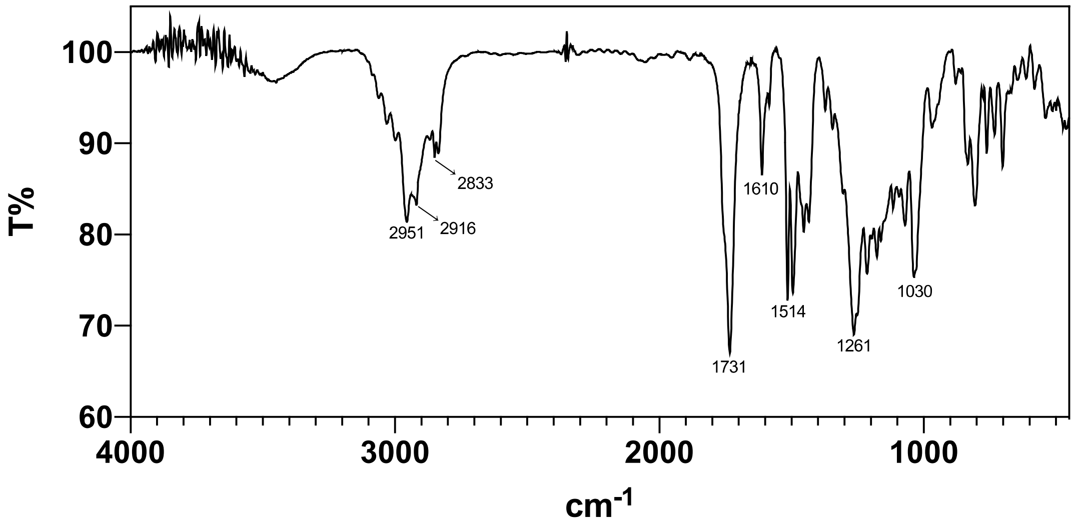
T%
100
90
80
70
60
4000
3000
2000
1000
cm-1
2833
2951
2916
1610
1514
1731
1261
1030

Acquisition Parameter					
				Set Corrector Fill	74 V
Source Type	ESI	Ion Polarity	Positive	Set Pulsar Pull	799 V
n/a	n/a	Set Capillary Exit	80.0 V	Set Pulsar Push	799 V
Scan Begin	50 m/z	Set Hexapole RF	60.0 V	Set Reflector	1700 V
Scan End	3000 m/z	Set Skimmer 1	50.0 V	Set Flight Tube	8600 V
		Set Hexapole 1	24.3 V	Set Detector TOF	2311 V

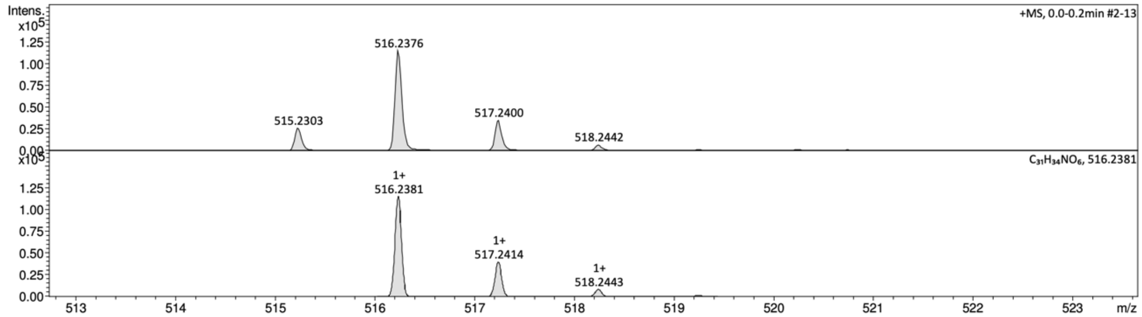

Meas. m/z	#	Ion Formula	m/z	err [ppm]	mSigma	# mSigma	Score	rdb	e⁻ Conf	N-Rule
516.2376	1	C31H34NO6	516.2381	0.8	21.9	1	100.00	15.5	even	ok

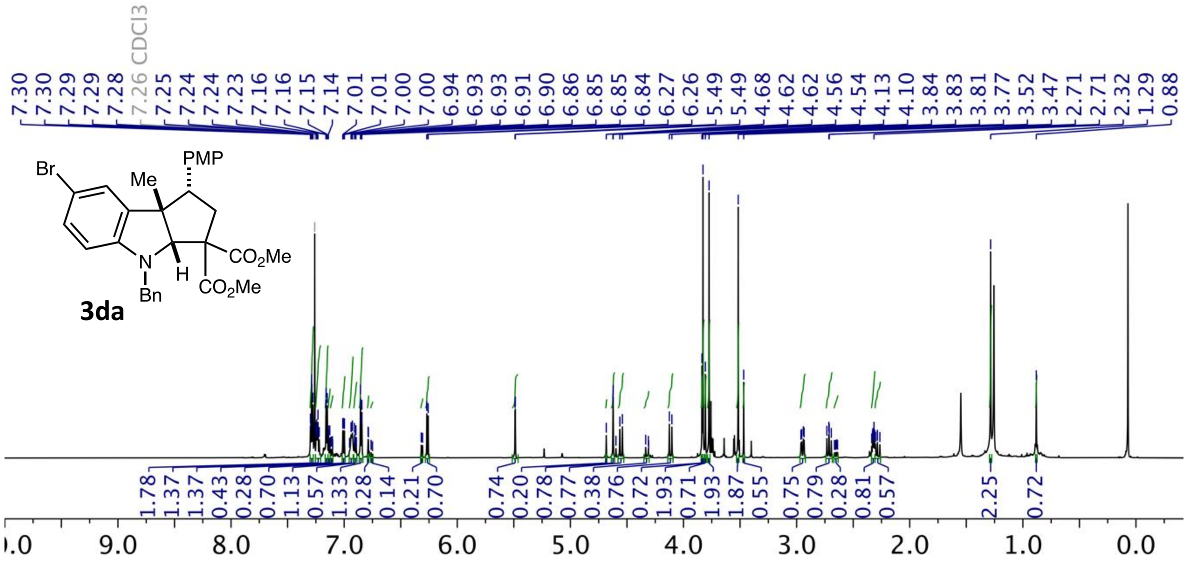
PMP
Br
Me
N
H
Bn
CO_2Me
CO_2Me
3da
7.26 CDCl3

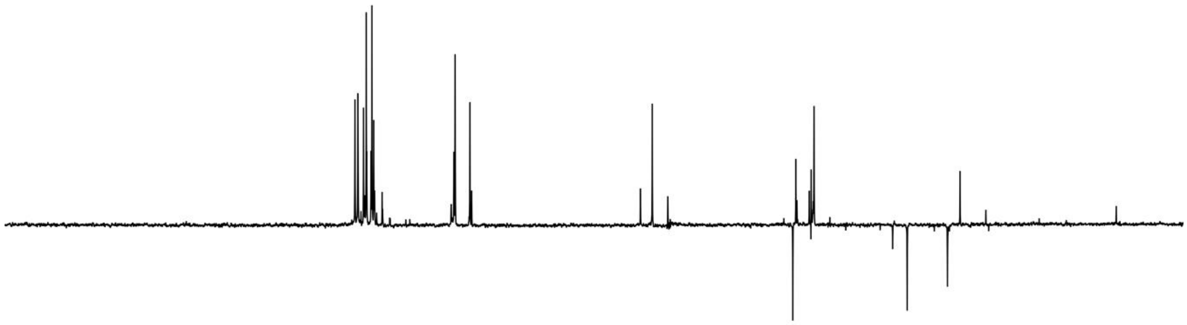

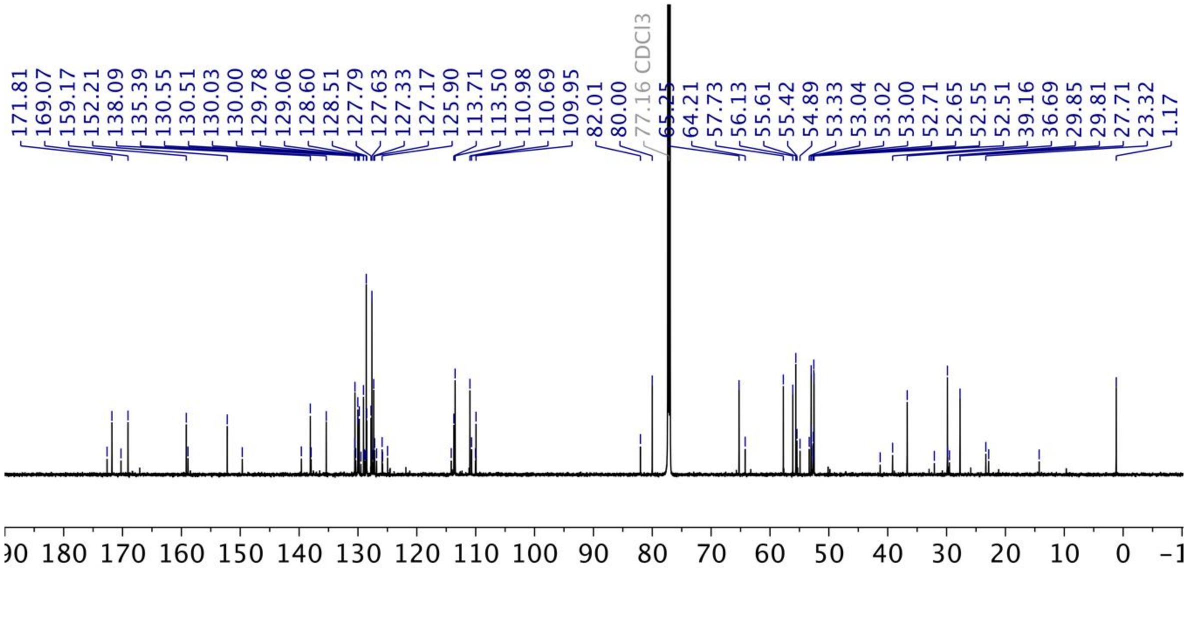
77.16 CDCl3

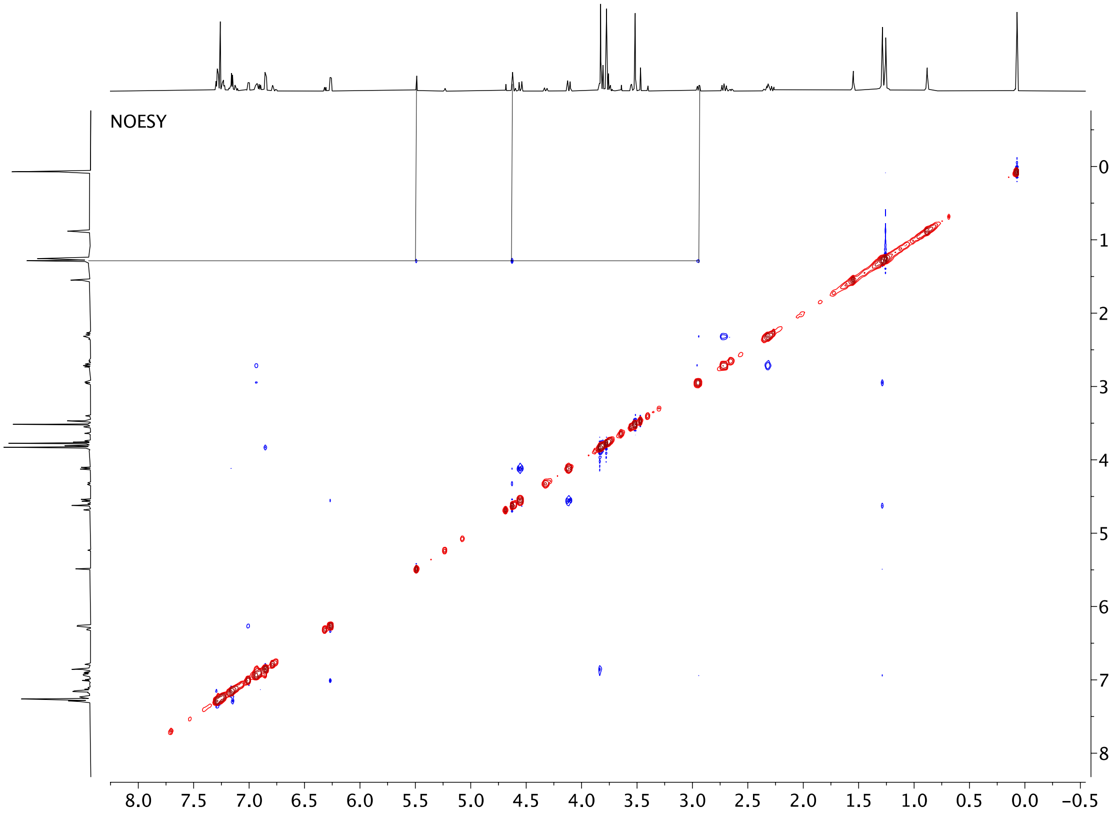
NOESY
8.0 7.5 7.0 6.5 6.0 5.5 5.0 4.5 4.0 3.5 3.0 2.5 2.0 1.5 1.0 0.5 0.0 −0.5
0 1 2 3 4 5 6 7 8

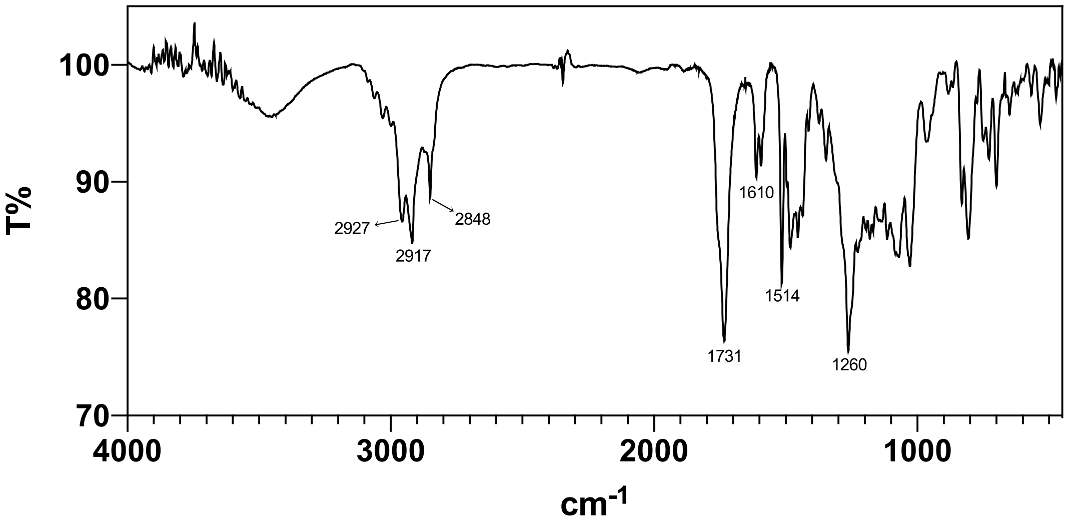
T%
100
90
80
70
4000 3000 2000 1000
cm-1
2927
2917
2848
1731
1610
1514
1260

Acquisition Parameter

Source Type	ESI	Ion Polarity	Positive	Set Corrector Fill	74 V
n/a	n/a	Set Capillary Exit	80.0 V	Set Pulsar Pull	799 V
Scan Begin	50 m/z	Set Hexapole RF	60.0 V	Set Pulsar Push	799 V
Scan End	3000 m/z	Set Skimmer 1	50.0 V	Set Reflector	1700 V
		Set Hexapole 1	24.3 V	Set Flight Tube	8600 V
				Set Detector TOF	2311 V

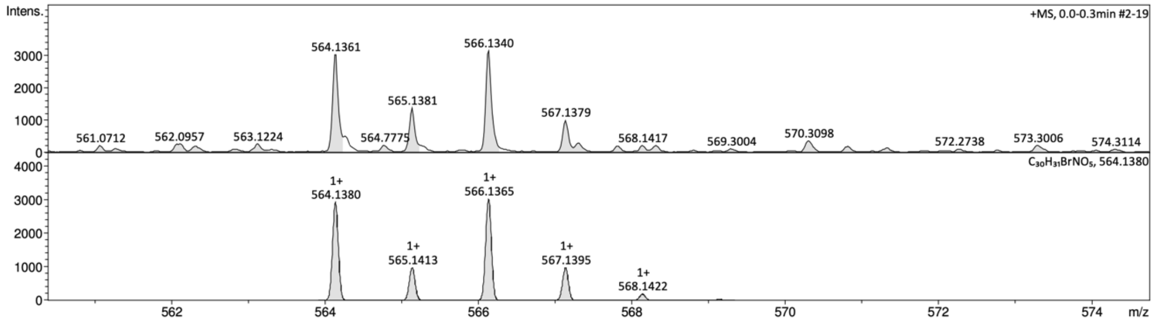

Meas. m/z	#	Ion Formula	m/z	err [ppm]	mSigma	# mSigma	Score	rdb	e⁻ Conf	N-Rule
564.1361	1	C30H31BrNO5	564.1380	3.3	51.6	1	100.00	15.5	even	ok

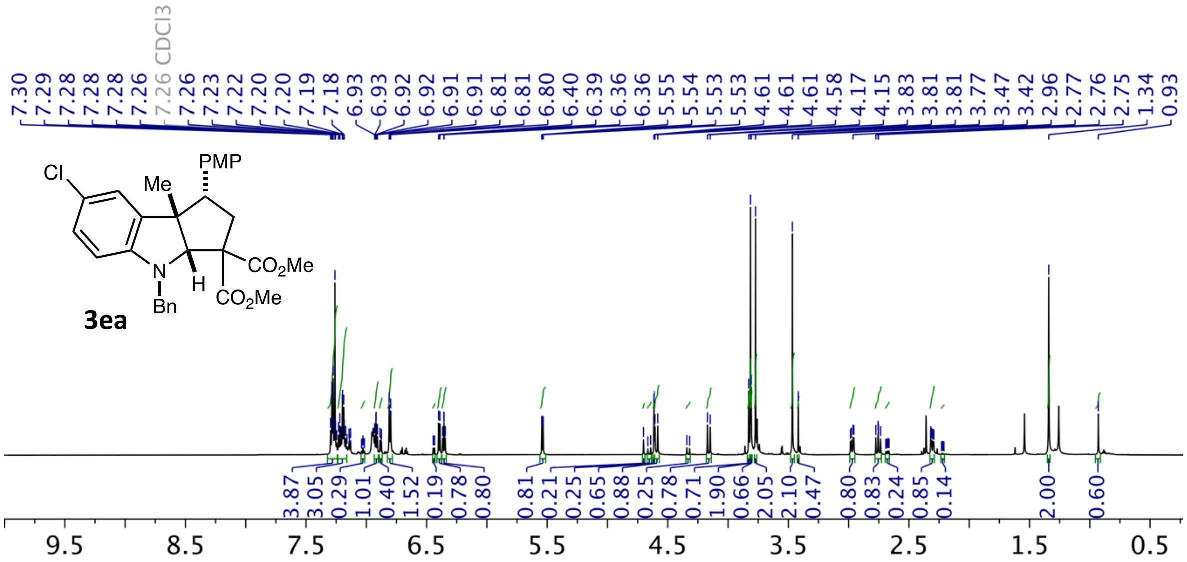
7.30 7.29 7.28 7.28 7.28 7.26 7.26 CDCl3 7.26 7.23 7.22 7.20 7.20 7.19 7.18 6.93 6.93 6.92 6.92 6.91 6.91 6.81 6.81 6.80 6.40 6.39 6.36 6.36 5.55 5.54 5.53 5.53 4.61 4.61 4.61 4.58 4.17 4.15 3.83 3.81 3.81 3.77 3.47 3.42 2.96 2.77 2.76 2.75 1.34 0.93
Cl
Me
PMP
CO2Me
CO2Me
N
H
Bn
3ea
3.87 3.05 0.29 1.01 0.40 1.52 0.19 0.78 0.80 0.81 0.21 0.25 0.65 0.88 0.25 0.78 0.71 1.90 0.66 2.05 2.10 0.47 0.80 0.83 0.24 0.85 0.14 2.00 0.60
9.5 8.5 7.5 6.5 5.5 4.5 3.5 2.5 1.5 0.5

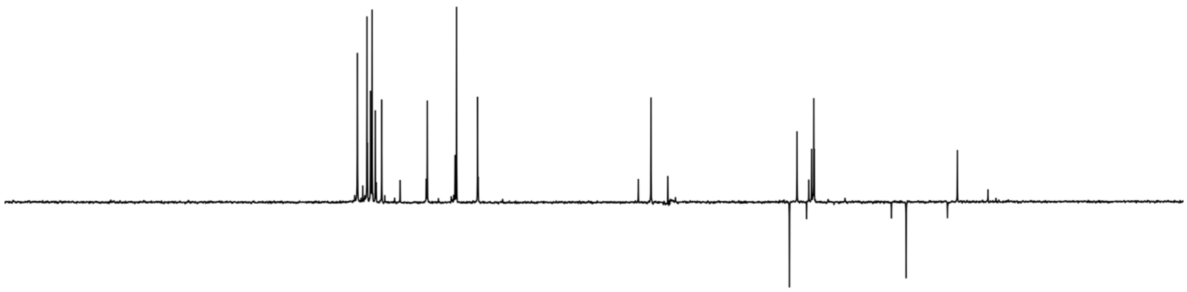

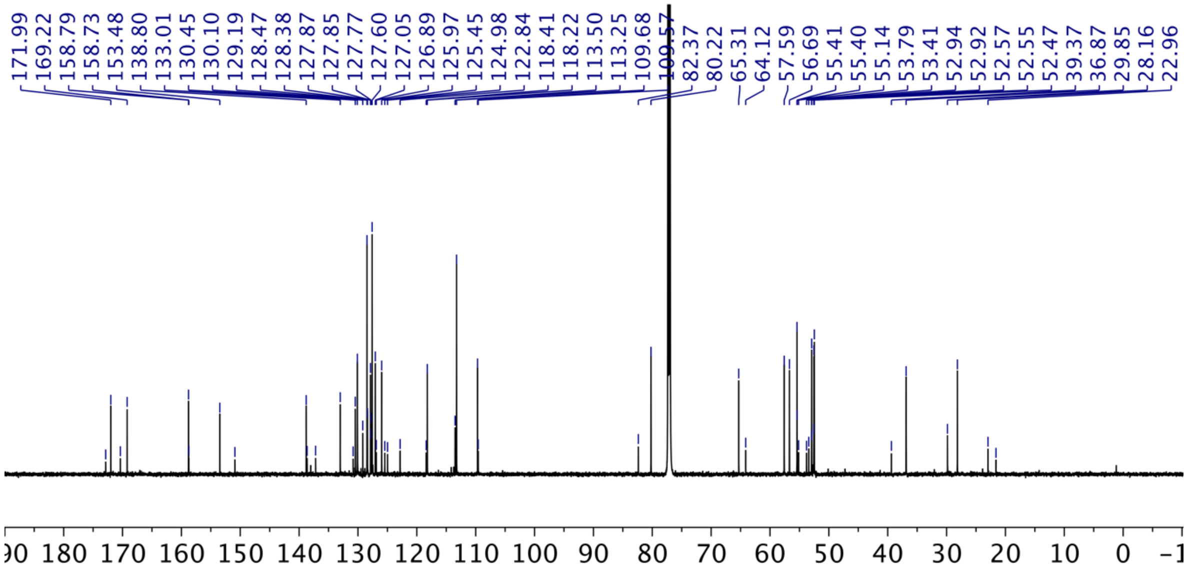
171.99 169.22 158.79 158.73 153.48 138.80 133.01 130.45 130.10 129.19 128.47 128.38 127.87 127.85 127.77 127.60 127.05 126.89 125.97 125.45 124.98 122.84 118.41 118.22 113.50 113.25 109.68 109.57 82.37 80.22 65.31 64.12 57.59 56.69 55.41 55.40 55.14 53.79 53.41 52.94 52.92 52.57 52.55 52.47 39.37 36.87 29.85 28.16 22.96
180 170 160 150 140 130 120 110 100 90 80 70 60 50 40 30 20 10 0

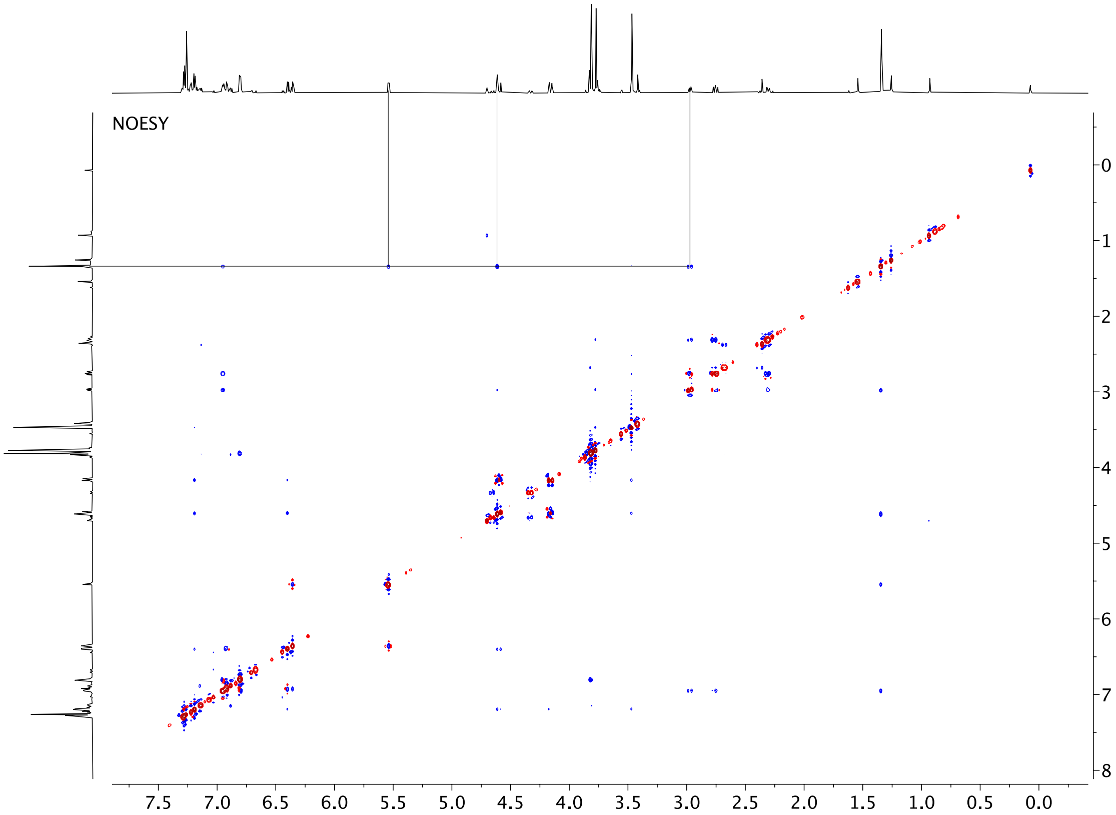
NOESY
7.5 7.0 6.5 6.0 5.5 5.0 4.5 4.0 3.5 3.0 2.5 2.0 1.5 1.0 0.5 0.0
0 1 2 3 4 5 6 7 8

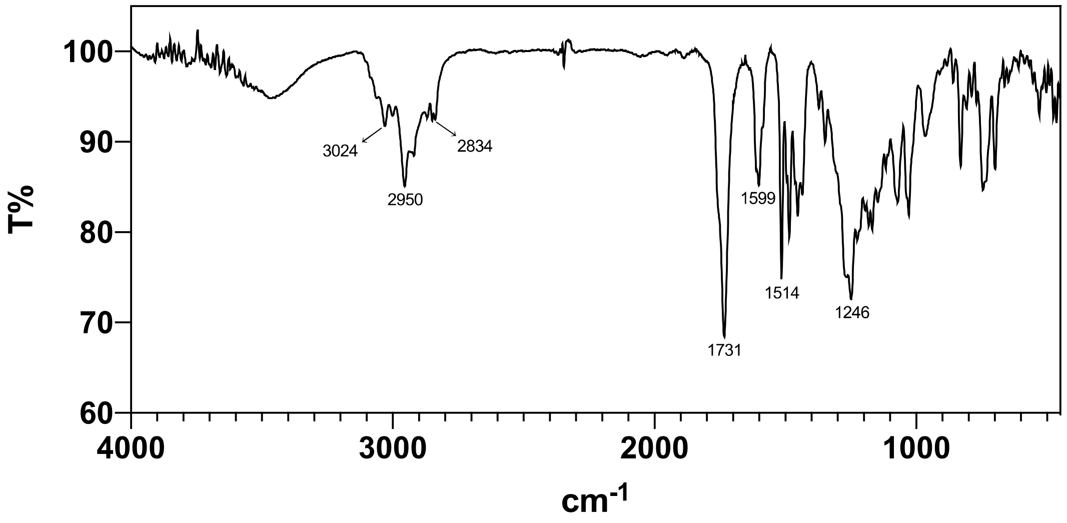
100
90
80
70
60
T%
4000 3000 2000 1000
cm-1
3024
2950
2834
1731
1599
1514
1246

Acquisition Parameter

Source Type	ESI	Ion Polarity	Positive	Set Corrector Fill	74 V
n/a	n/a	Set Capillary Exit	80.0 V	Set Pulsar Pull	799 V
Scan Begin	50 m/z	Set Hexapole RF	60.0 V	Set Pulsar Push	799 V
Scan End	3000 m/z	Set Skimmer 1	50.0 V	Set Reflector	1700 V
		Set Hexapole 1	24.3 V	Set Flight Tube	8600 V
				Set Detector TOF	2311 V

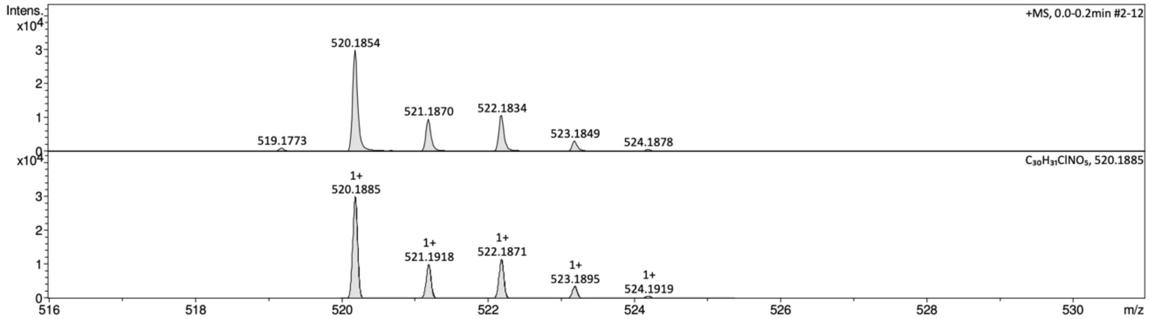

Meas. m/z	#	Ion Formula	m/z	err [ppm]	mSigma	# mSigma	Score	rdb	e⁻ Conf	N-Rule
520.1854	1	C30H31ClNO5	520.1885	5.9	16.8	1	100.00	15.5	even	ok

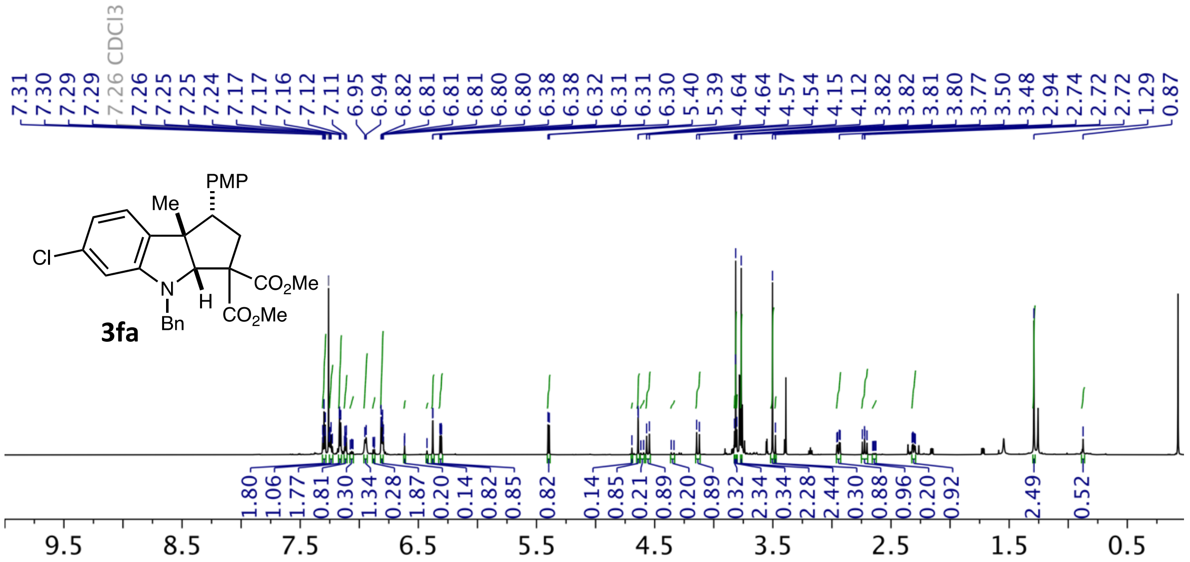
7.31 7.30 7.29 7.29 7.26 CDCl3 7.26 7.25 7.25 7.24 7.17 7.17 7.16 7.12 7.11 6.95 6.94 6.82 6.81 6.81 6.81 6.80 6.80 6.38 6.38 6.32 6.31 6.31 6.30 5.40 5.39 4.64 4.64 4.57 4.54 4.15 4.12 3.82 3.82 3.81 3.80 3.77 3.50 3.48 2.94 2.74 2.72 1.29 0.87
PMP
Me
Cl
N
H
CO2Me
CO2Me
Bn
3fa
1.80 1.06 1.77 0.81 0.30 1.34 0.28 1.87 0.20 0.14 0.82 0.85 0.82 0.14 0.85 0.21 0.89 0.20 0.89 0.32 2.34 0.34 2.28 2.44 0.30 0.88 0.96 0.20 0.92 2.49 0.52
9.5 8.5 7.5 6.5 5.5 4.5 3.5 2.5 1.5 0.5

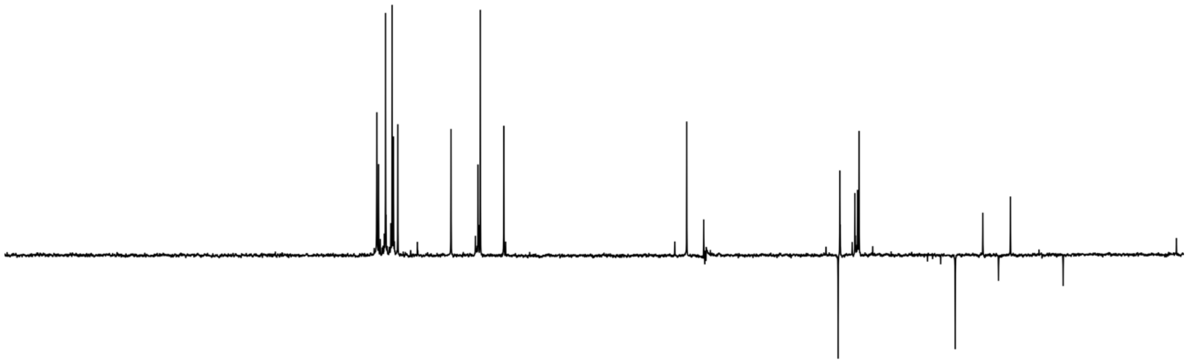

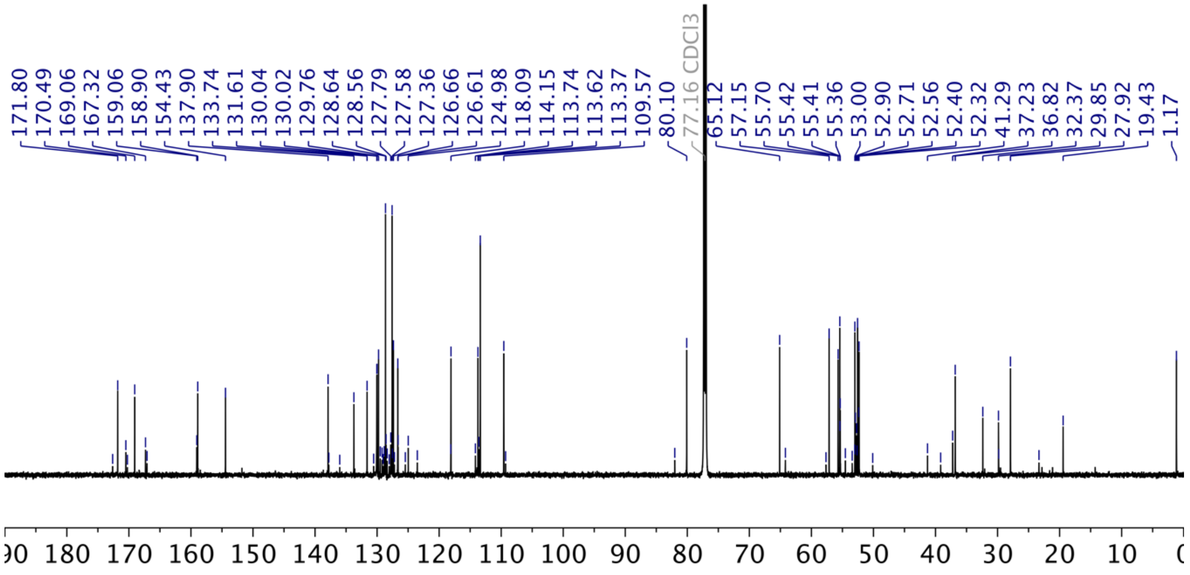
171.80 170.49 169.06 167.32 159.06 158.90 154.43 137.90 133.74 131.61 130.04 130.02 129.76 128.64 128.56 127.79 127.58 127.36 126.66 126.61 124.98 118.09 114.15 113.74 113.62 113.37 109.57 80.10 77.16 CDCl3 65.12 57.15 55.70 55.42 55.41 55.36 53.00 52.90 52.71 52.56 52.40 52.32 41.29 37.23 36.82 32.37 29.85 27.92 19.43 1.17
90 180 170 160 150 140 130 120 110 100 90 80 70 60 50 40 30 20 10 0

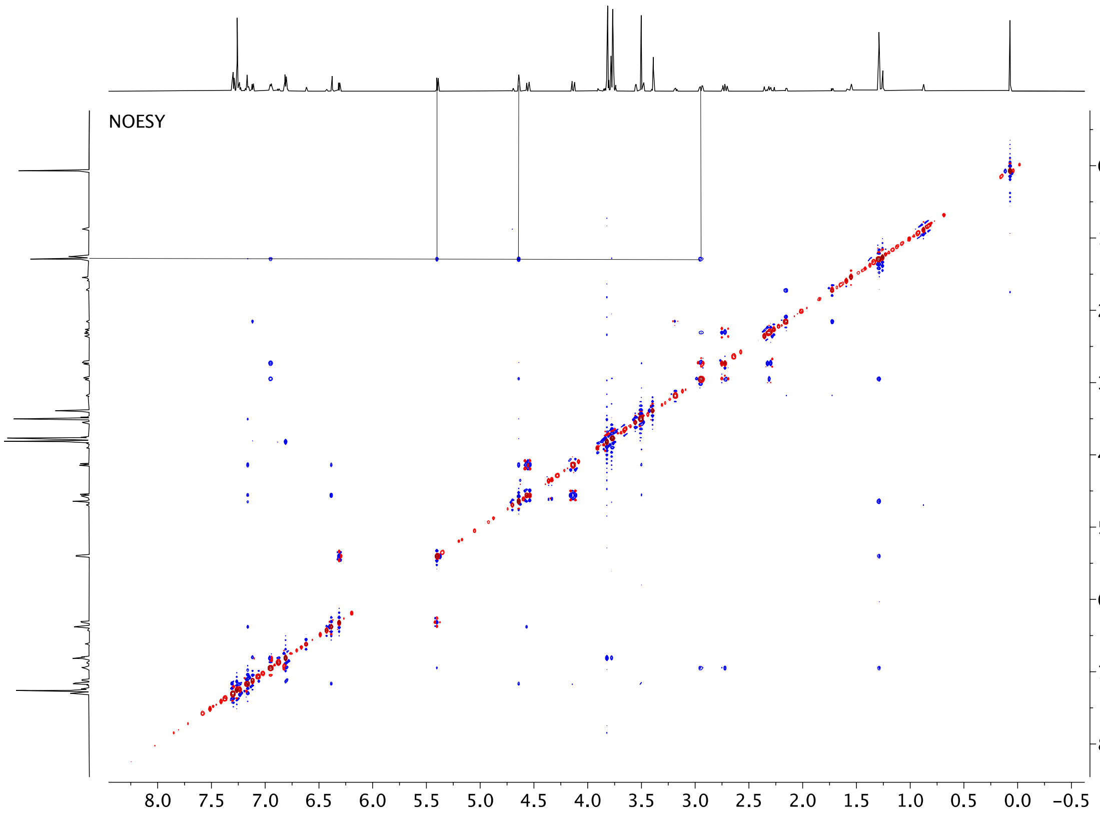
NOESY
8.0
7.5
7.0
6.5
6.0
5.5
5.0
4.5
4.0
3.5
3.0
2.5
2.0
1.5
1.0
0.5
0.0
−0.5
0
1
2
3
4
5
6
7
8

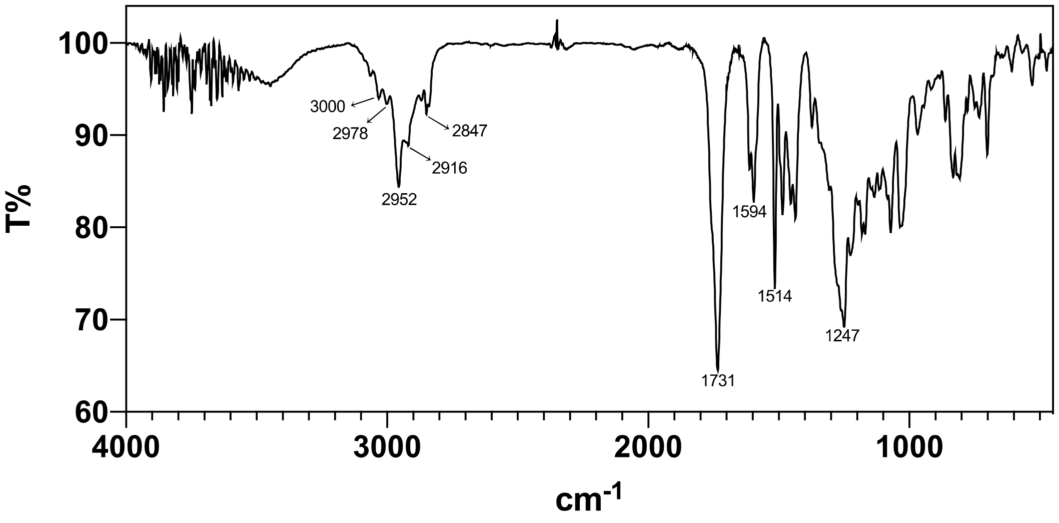
T%
100
90
80
70
60
4000
3000
2000
1000
cm-1
3000
2978
2952
2916
2847
1731
1594
1514
1247

6. Crystal data and structure refinement for 3ac.

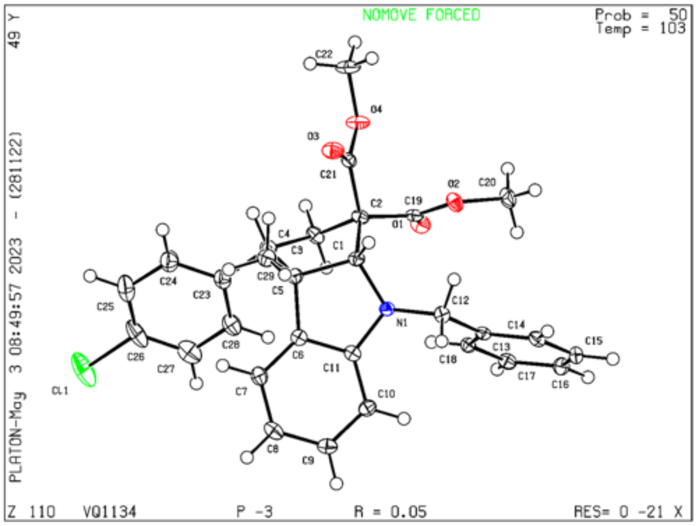

Crystal data

$C_{29}H_{28}ClNO_4$

M_r = 489.97

Trigonal, $P\bar{3}$

Hall symbol: -P 3

a = 24.3118 (8) Å

c = 7.4297 (4) Å

V = 3803.1 (3) Å^3

Z = 6

F(000) = 1548

D_x = 1.284 Mg m^{-3}

Mo $K\alpha$ radiation, λ = 0.71073 Å

Cell parameters from 9907 reflections

θ = 2.6–28.1°

μ = 0.19 mm^{-1}

T = 103 K

Plate, yellow

0.13 × 0.07 × 0.05 mm

Data collection

Bruker D8 VENTURE PHOTON III-14 diffractometer

Radiation source: INCOATEC microfocus sealed tube, Incoatec IμS 3.0

Incoatec multilayer mirror monochromator

Detector resolution: 7.3910 pixels mm^{-1}

ω and phi scans

Absorption correction: multi-scan BRUKER *SADABS2016*/2

T_{min} = 0.887, T_{max} = 0.938

98238 measured reflections

4794 independent reflections

3829 reflections with $I > 2\sigma(I)$

R_{int} = 0.101

θ_{max} = 25.7°, θ_{min} = 1.9°

h = −29→29

k = −29→29

l = −9→9

Refinement

Refinement on F^2

Least-squares matrix: full

$R[F^2 > 2\sigma(F^2)]$ = 0.047

$wR(F^2)$ = 0.123

S = 1.05

4794 reflections

319 parameters

0 restraints

0 constraints

Primary atom site location: dual

Secondary atom site location: dual

Hydrogen site location: inferred from neighbouring sites

H-atom parameters constrained

$w = 1/[\sigma^2(F_o^2) + (0.0479P)^2 + 4.2209P]$ where $P = (F_o^2 + 2F_c^2)/3$

$(\Delta/\sigma)_{max}$ < 0.001

$\Delta\rho_{max}$ = 1.05 e Å^{-3}

$\Delta\rho_{min}$ = −0.65 e Å^{-3}
